# Ligand-to-metal charge transfer facilitates photocatalytic oxygen atom transfer (OAT) with *cis*-dioxo molybdenum(vi)-Schiff base complexes†

**DOI:** 10.1039/d4sc02784a

**Published:** 2024-09-06

**Authors:** Thorsten Dreher, Lukas Geciauskas, Samuel Steinfeld, Barbara Procacci, Adrian C. Whitwood, Jason M. Lynam, Richard E. Douthwaite, Anne-K. Duhme-Klair

**Affiliations:** a Department of Chemistry, University of York Heslington YO10 5DD York UK richard.douthwaite@york.ac.uk anne.duhme-klair@york.ac.uk

## Abstract

Systems incorporating the *cis*-Mo(O)_2_ motif catalyse a range of important thermal homogeneous and heterogeneous oxygen atom transfer (OAT) reactions spanning biological oxidations to platform chemical synthesis. Analogous light-driven processes could offer a more sustainable approach. The *cis*-Mo(O)_2_ complexes reported here photocatalyse OAT under visible light irradiation, and operate *via* a non-emissive excited state with substantial ligand-to-metal charge-transfer (LMCT) character, in which a Mo=O π*-orbital is populated *via* transfer of electron density from a chromophoric salicylidene-aminophenol (SAP) ligand. SAP ligands can be prepared from affordable commercially-available precursors. The respective *cis*-Mo(O)_2_-SAP catalysts are air stable, function in the presence of water, and do not require additional photosensitisers or redox mediators. Benchmark OAT between phosphines and sulfoxides shows that electron withdrawing groups (*e.g.* C(O)OMe, CF_3_) are necessary for photocatalytic activity. The photocatalytic system described here is mechanistically distinct from both thermally catalysed OAT by the *cis*-Mo(O)_2_ motif, as well as typical photoredox systems that operate by outer sphere electron transfer mediated by long-lived emissive states. Both photoactivated and thermally activated OAT steps are coupled to establish a catalytic cycle, offering new opportunities for the development of photocatalytic atom transfer based on readily-available, high-valent metals, such as molybdenum.

## Abstract 2

Air-stable molybdenum(vi)-dioxo complexes with chromophoric salicylidene aminophenol ligands mediate photocatalytic oxygen atom transfer under visible light irradiation *via* a non-emissive photo-excited state with significant LMCT character.

## Graphical abstract

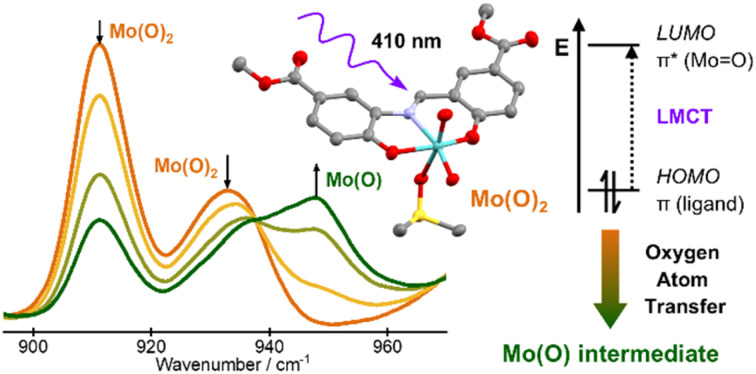

## Introduction

**Scheme 1:**
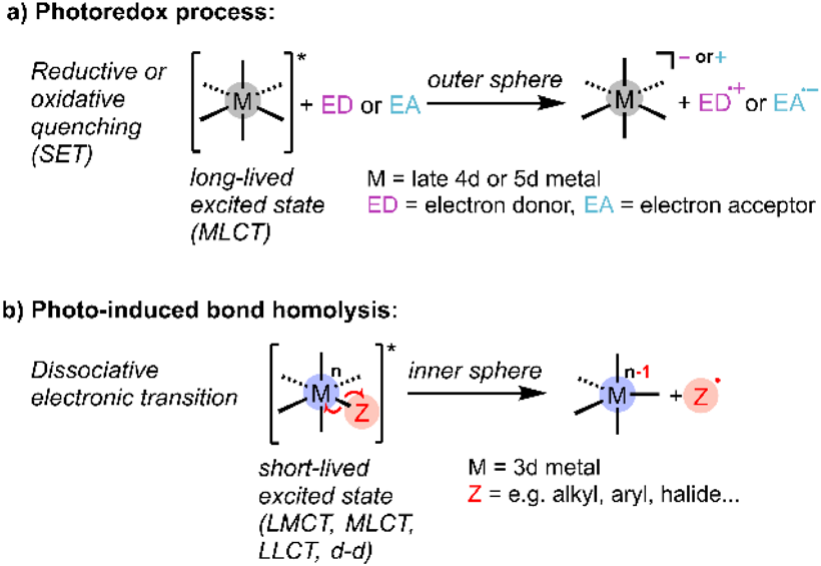
Schematic representation of conventional (a) photoredox and (b) bond homolysis processes used in homogeneous photocatalysis (SET: single-electron transfer; CT: charge transfer; M: metal, L: ligand).

It is well recognised that light could help meet energy needs for driving chemical reactions,^1^ and the last decade has seen intense interest in photocatalysis for synthesis, environmental remediation, and energy storage.^2^ In particular, metal-based photocatalysts have been heavily investigated because of their favourable photophysical properties and opportunities for tailored design.^3^ Mechanistically, most homogeneous photoredox chemistry can be characterised as arising from outer-sphere single-electron transfer (SET) between a photoexcited catalyst and substrate to generate radicals, which undergo subsequent reactions (Scheme 1a).^4–6^ These photocatalytic reactions are typically mediated by emissive metal-to-ligand charge transfer (MLCT) excited states with lifetimes in the μs regime, sufficient to support bimolecular reactivity, as exemplified by precious metal polypyridyl complexes.^7–10^ Other established metal-based photo-reactions suitable for incorporation into catalytic cycles are ligand dissociation and reductive elimination from short-lived (<ns) excited states of organometallic complexes.^11,12^

Recently there has been much interest in developing Earth-abundant metal-containing photocatalysts that operate *via* ligand-to-metal charge transfer (LMCT).^11,13,14^ These photocatalysts are typically high-valent complexes where LMCT induces radical reactivity *via* inner-sphere electron transfer, in some cases overcoming the thermodynamic limits of the more common outer-sphere SET *via* MLCT.^15,16^ Known reactivity of LMCT excited states include radical ligand dissociation, for example of halides (Scheme 1b), which can then exhibit catalytic reactivity,^17^ and atom abstraction, for example hydrogen atom transfer (HAT) from hydrocarbons observed for metal oxo complexes.^18^

**Scheme 2:**
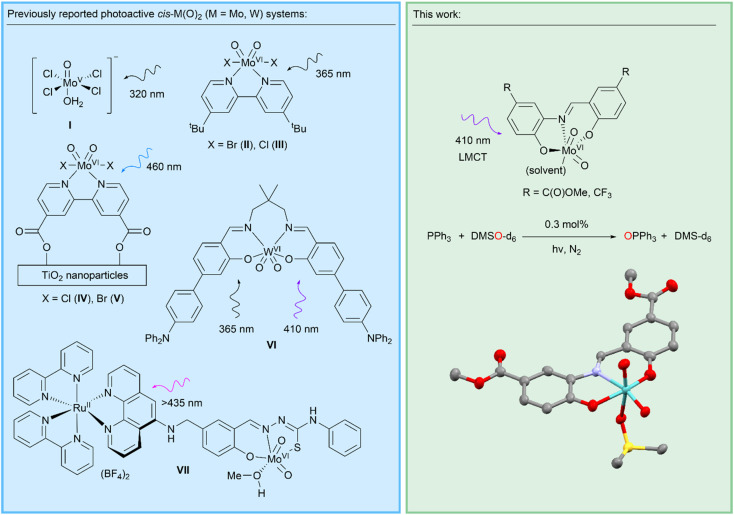
Previously-reported photoactive *cis*-Mo(O)_2_ and *cis*-W(O)_2_ complexes, and an exemplary photocatalytic system developed in this work, including a crystal structure of the [*cis*-Mo^VI^(O)_2_(κ^3^-SAP^C(O)OMe^)(Me_2_SO)] complex (3^C(O)OMe^_DMSO_) reported here (ORTEP plot (50% probability); H atoms omitted for clarity; C, grey; N, lavender; O, red; S, yellow; Mo, cyan).

The focus of the work presented here is the LMCT-induced reactivity of molybdenum oxo complexes to enable photocatalytic oxygen atom transfer (OAT) reactions. Molybdenum oxo and dioxo systems are found in molybdoenzymes, bio-inspired metal complexes, and metal molybdates,^19–22^ and are critical for supporting a wide range of thermally activated homogeneous and heterogeneous catalytic reactions found in biology, chemical synthesis, and industrial processes.^23–25^ These include a family of mononuclear molybdoenzymes containing an oxo ligand and pyranopterin dithiolene cofactor which acts as a non-innocent ligand, supporting OAT *via* two sequential one-electron transfer steps.^23,26–29^ Many classes of bio-inspired Mo-oxo and *cis*-Mo-dioxo complexes incorporating multidentate ligands have also been investigated for OAT reactions such as epoxidations.^30–34^ Heterogeneous metal molybdates catalyse a range of industrially-important heterogenous reactions such as propene oxidation, where surface Mo-oxo ligands are also implicated in concerted OAT-type reactivity.^30,35^ Mechanistically, all these reactions typically cycle through Mo(VI–IV) oxidation states *via* SET or concerted OAT.^21^ In addition to these thermally active Mo-catalysts, a number of photoactive systems incorporating the Mo(vi)-oxo motif are known (Scheme 2). Early work described [Mo(O)Cl_4_(H_2_O)]^−^ (I) and [*cis*-Mo(O)_2_Br_2_(*t*Bu-bipy)] (II) complexes for stoichiometric OAT to phosphines and alkenes under UV irradiation.^36,37^ More recently, a related [*cis*-Mo(O)_2_Cl_2_(*t*Bu-bipy)] (III) system was shown to stoichiometrically activate C–H bonds *via* HAT.^38,39^ A characteristic limiting feature of these systems is the formation of a [(O)Mo^V^–O–Mo^V^(O)] oxo-bridged complex^40–44^ that prevents catalytic turnover.^38^ Partly to overcome this limitation, the C(O)OH-substituted system [*cis*-Mo(O)_2_X_2_((C(O)OH)_2_-bipy)] (X = Cl(iv), Br(v)) was supported on UV photoactive TiO_2_ nanoparticles.^45–49^ These composite systems can be regenerated in a separate dark oxidation step, mimicking a catalytic cycle. Photoactivated OAT to phosphines has been achieved catalytically using a dyad (VII) comprising a [Ru^II^(bipy)_3_]^2+^ photosensitiser covalently-linked to a *cis*-Mo^VI^(O)_2_ cocatalyst which operates under visible light but does not incorporate direct photoactivation of the *cis*-Mo^VI^(O)_2_ moiety.^50^ This bio-inspired system requires methyl viologen as a redox mediator and significant catalyst degradation occurs. Of wider relevance, *cis*-W^VI^(O)_2_ complexes of highly-conjugated macrocyclic salen ligands (*e.g.*VI) have μs emissive states that mediate a number of photocatalytic transformations, including C–H activation and C–C coupling reactions, *via* SET-induced radical pathways.^51^ Collectively, these seminal reports suggest that there is an opportunity to exploit LMCT into a Mo=O π* orbital to support photocatalytic OAT that could expand the extensive thermal chemistry of this motif. We hypothesised that LMCT transitions that lead to the population of antibonding Mo=O orbitals can promote the labilisation of oxo ligands by decreasing their bond order, thereby facilitating photocatalytic OAT. We were motivated to explore d^0^ Mo-dioxo complexes that incorporate ligand systems that support charge-transfer transitions and enable catalytic turnover that is unrestricted by the formation of kinetically-inert [(O)Mo^V^–O–Mo^V^(O)] complexes under the chosen experimental conditions. Here we report a series of Mo-dioxo complexes of Schiff base ligands that are air stable, support LMCT under visible light irradiation and mediate photocatalytic OAT in the presence of water without photocatalyst degradation.

## Results and discussion

### Synthesis and characterisation of ligands and complexes

**Scheme 3:**
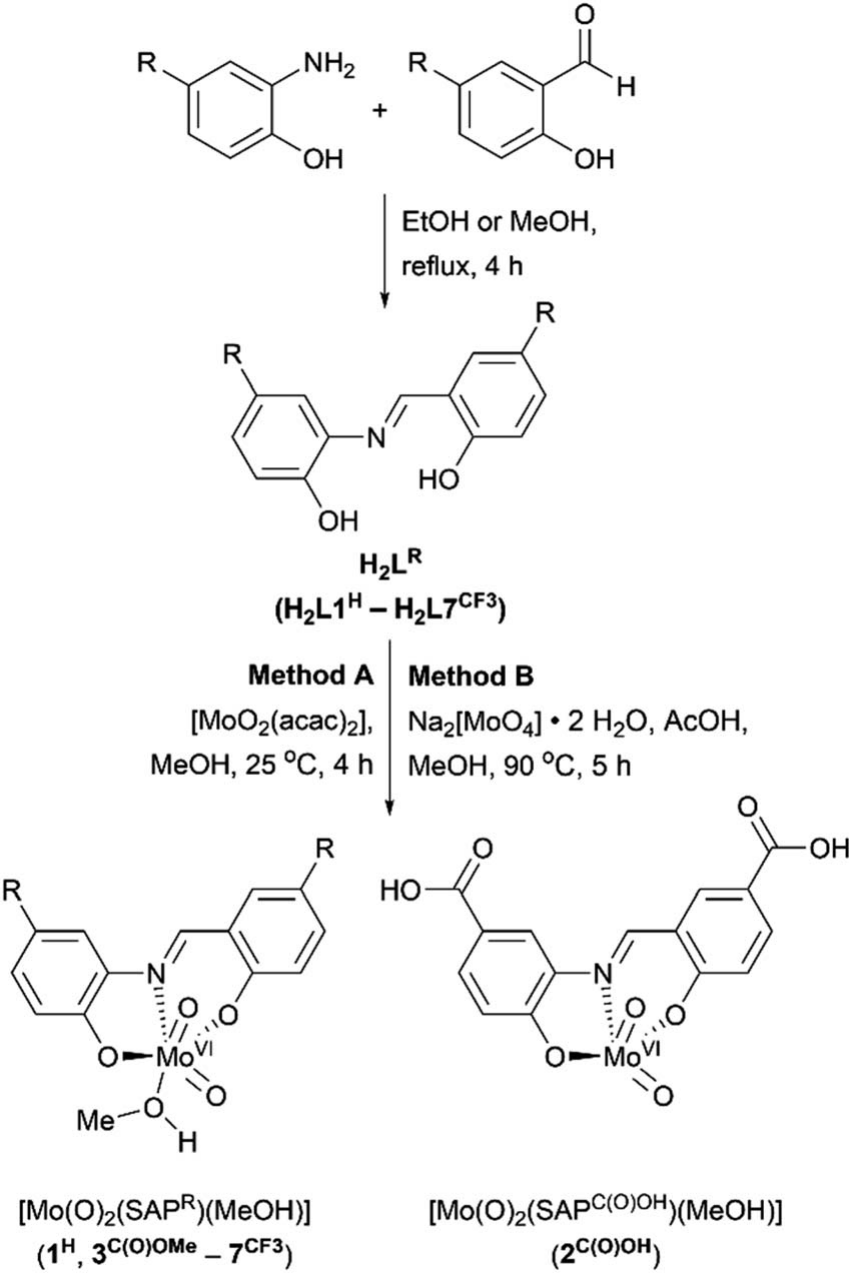
Synthesis of SAP-type ligands (H_2_L^R^) and *cis*-Mo^VI^(O)_2_ complexes (1^H^–7^CF3^). Where R = H (H_2_L1^H^ and 1^H^); C(O)OH (H_2_L2^C(O)OH^ and 2^C(O)OH^); C(O)OMe (H_2_L3^C(O)OMe^ and 3^C(O)OMe^); F (H_2_L4^F^ and 4^F^); *t*Bu (H_2_L5*^t^*^Bu^ and 5*^t^*^Bu^); OMe (H_2_L6^OMe^ and 6^OMe^); CF_3_ (H_2_L7^CF3^ and 7^CF3^).

The ligand system used in this work is based on a tridentate meridionally-coordinating salicylidene-aminophenol (SAP) motif, which is known to support visible-light induced LMCT transitions if coordinated to *cis*-Mo^VI^(O)_2_.^52,53^ Since SAP-type ligands can be prepared in a single step from commercially available precursors (Scheme 3), substituent effects could be used to vary their spectroscopic and electronic properties systematically and potentially limit oxo-bridged bimetallic complex formation.

Reactions between SAP ligands and [MoO_2_(acac)_2_] or Na_2_[MoO_4_]·2H_2_O in methanol give the target complexes 1^H^–7^CF3^, comprising meridional SAP coordination to a *cis*-Mo^VI^(O)_2_ moiety, with additional methanol coordination for most complexes giving [*cis*-Mo^VI^(O)_2_(κ^3^-SAP^R^)(MeOH)] with pseudo-octahedral geometry. The only exception was complex 2^C(O)OH^ which does not exhibit solvent coordination in the solid state. The proposed compositions were confirmed by ^1^H and ^13^C NMR, ATR-IR, and UV-vis spectroscopies, mass spectrometry, elemental analysis, and X-ray crystallography (see ESI†). Complexes 1^H^–7^CF3^ are pale yellow to dark red, and are air stable as solids and in solution, with good solubility in DMSO and DMF. All metrical data are consistent with similar *cis*-Mo^VI^(O)_2_ complexes of tridentate ligands.^44,54–59^

### Photocatalytic testing

Previously, *cis*-Mo^VI^(O)_2_-containing catalysts have been extensively studied for conventional thermal OAT between phosphines and sulfoxides as oxygen atom acceptors and donors, respectively, to mimic biological processes and investigate thermodynamic and kinetic features.^21,40,41^ In these studies, Hammett correlations are found for various ligand derivatives and substrates, and rates are reflective of the thermodynamic driving force between phosphine and sulfoxide.^41,42,50,60^ For comparison, we therefore initially examined the prototypical OAT reaction between PPh_3_ and DMSO-d_6_. Photoreactions were performed by irradiating at 410 nm, based on the UV-vis spectra of 1^H^–3^C(O)OMe^ (see Fig. S31, ESI†) which show very similar features, including a broad absorption band centered around 420 nm, assigned to a transition with substantial LMCT character based on TD-DFT simulations (see below).

Photoreactions with complexes 1^H^–3^C(O)OMe^ in excess DMSO-d_6_ and 300 equivalents of PPh_3_ over 3 hours showed that complex 1^H^ is essentially photoinactive. However, complexes 2^C(O)OH^ and 3^C(O)OMe^ exhibited 35% and 78% conversions of PPh_3_ to OPPh_3_, respectively, with essentially quantitative selectivity as determined by ^1^H and ^31^P NMR spectroscopy (Table 1, entries 1–3). In the absence of irradiation, complex 3^C(O)OMe^ is inactive (Table 1, entry 4). Irradiation causes heating of the reaction mixture to 45 °C, however in the absence of light, no reaction occurs on heating to 45 °C, confirming that OAT is induced by light (Table 1, entry 5). Further control experiments showed that no appreciable activity is observed in the absence of catalyst 3^C(O)OMe^ or using the SAP ligand precursor H_2_L3^C(O)OMe^ (Table 1, entries 6–7). The reaction was also monitored by ^1^H NMR over 3 hours, requiring periodic cessation of irradiation.

**Table 1:** OAT reactions between PPh_3_ and DMSO-d_6_^a^

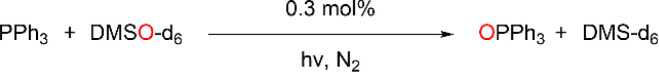				
Entry	Catalyst	PPh_3_ conversion/%^b^	TON	TOF/h^−1^
1	1^H^	3	9	3
2	2^C(O)OH^	35	105	35
3	3^C(O)OMe^	78	234	78
4^c^	3^C(O)OMe^	0	0	0
5^d^	3^C(O)OMe^	0	0	0
6	None	0	0	0
7	H_2_L3^C(O)OMe^	3	9	3
8^e^	3^C(O)OMe^	73	219	73
9^f^	3^C(O)OMe^	83	249	83
10	4^F^	2	6	2
11	5*^t^*^Bu^	0	0	0
12	6^OMe^	1	3	1
13	7^CF3^	97	291	97

a General conditions: catalyst (1 mM in DMSO-d_6_), 300 equivalents PPh_3_, 410 nm, 3 h, under N_2_.

b Conversion determined by ^1^H NMR spectroscopy.

c In the dark.

d In the dark and at 45 °C.

e Added water (300 mM).

f Under air.

**Fig. 1:**
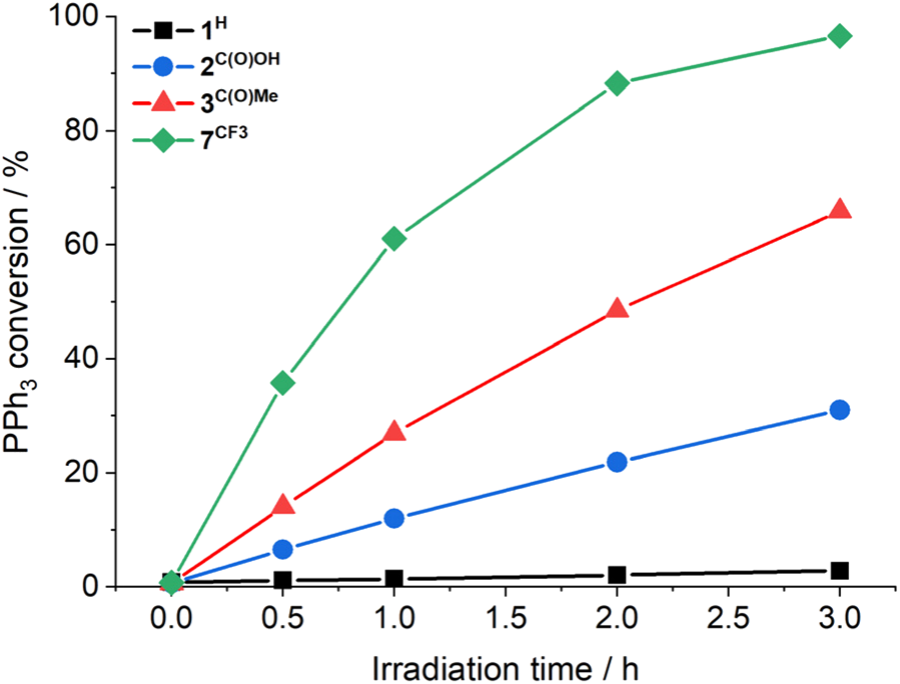
Photocatalytic OAT from DMSO-d_6_ to PPh_3_ as a function of time using 1^H^ (black squares), 2^C(O)OH^ (blue circles), 3^C(O)OMe^ (red triangles), and 7^CF3^ (green diamonds) (1 mM in DMSO-d_6_), 300 equivalents PPh_3_, 410 nm, 3 h, under N_2_; PPh_3_ conversion was determined by ^1^H NMR spectroscopy. Note: the lines are added to guide the eye, and do not represent data fitting.

There is no apparent loss in activity for 3^C(O)OMe^ over the monitored time (Fig. 1). The ^1^H NMR resonances of complexes 2^C(O)OH^ and 3^C(O)OMe^ remain unchanged after photoreaction, confirming photocatalyst stability for these complexes (see Fig. S45 and S46, ESI†). Furthermore, reactions using complex 3^C(O)OMe^ tolerate exposure to both air and water, again with no apparent catalyst degradation (Table 1, entries 8–9). The observation of the remarkable photocatalytic activity and stability for 2^C(O)OH^ and 3^C(O)OMe^ prompted the investigation of complexes 4^F^–7^CF3^ containing a range of electron withdrawing (EWG) and donating (EDG) groups, respectively (Table 1, entries 10–13). Hammett plots (see Fig. S48, ESI†) show that there is a threshold for photocatalytic activity: compounds with EDGs are mostly inactive whilst the EWGs studied lead to increased activity, with [Mo^VI^(O)_2_(SAP^CF3^)(MeOH)] (7^CF3^) approaching quantitative conversion after 3 hours of irradiation (Fig. 1). Conventional ‘thermal’ OAT catalysts typically show a linear Hammett correlation.^41,42,50,60^ For the active catalysts described here linearity is not observed indicating that the reaction rate is not driven by a single thermodynamic parameter.

Finally, we tested three different phosphines, using increasing equivalents (100, 200 and 400), in combination with catalysts 3^C(O)OMe^ and 7^CF3^ and in all cases, the reactions were found to be *pseudo*-first order in phosphine (see Table S4, Fig. S49 and S50, ESI†), as expected based on literature reports.^41,61–63^ PPh_3_ and (P(*p*-F-Ph)_3_ gave rise to similar *k*_obs_ values, in line with the respective Hammett parameters^64^ (*p*-F, *σ*_p_ = 0.06 and H, *σ*_p_ = 0). In comparison, P(*p*-CF_3_-Ph)_3_), the phosphine with the most electron-withdrawing substituent (*p*-CF_3,_*σ*_p_ = 0.53), gave rise to significantly lower *pseudo*-first-order rate constants. P(*p*-CF_3_-Ph)_3_ is not only the poorest nucleophile but also the most difficult phosphine to oxidise. Similar observations have previously been reported for thermally-activated catalysts.^65^

### TD-DFT calculations and electronic spectroscopy

**Fig. 2:**
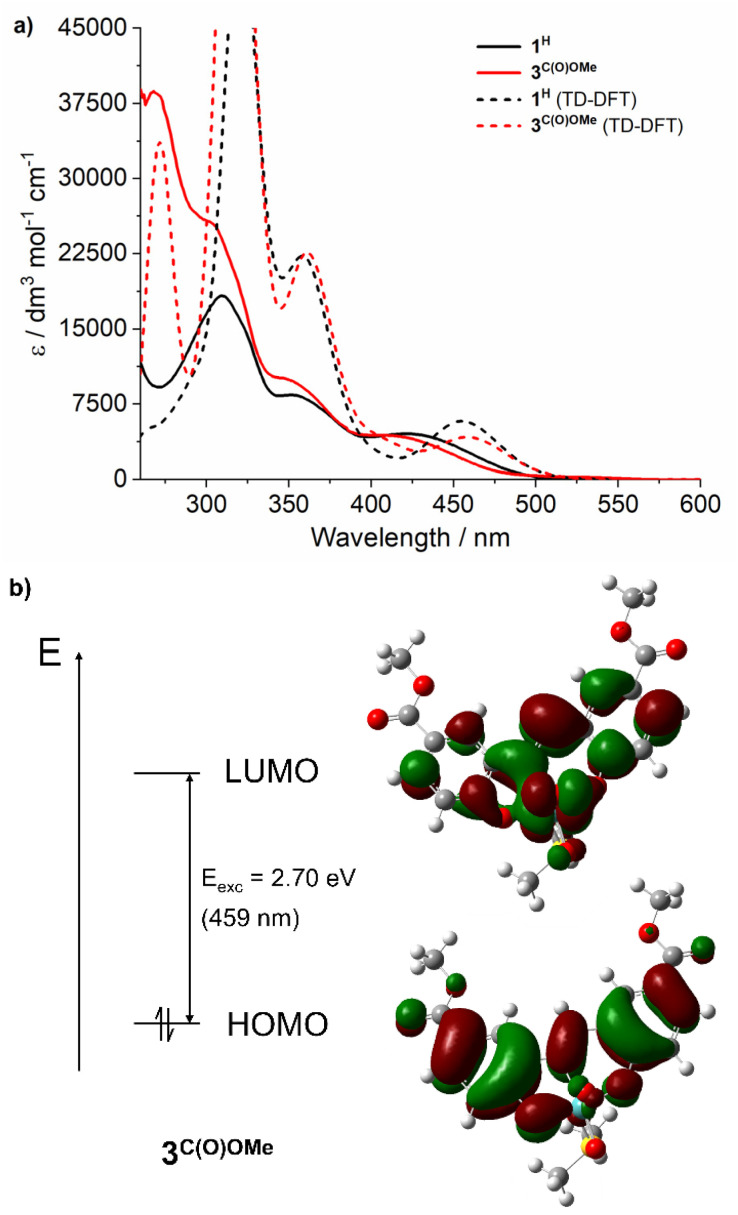
(a) Electronic absorption spectra of 1^H^ and 3^C(O)OMe^ at 0.05 mM in DMSO (solid lines), and spectra calculated at the (PBE0/DEF2-TZVPP//BP86/DEF2-SVPP; SCRF in DMSO) level of theory using TD-DFT simulations (dashed lines); Gaussian functions were fitted onto DFT determined transitions to obtain the calculated spectra. (b) Calculated ground state HOMO (iso-surface level 121) and LUMO (iso-surface level 122) orbitals for 3^C(O)OMe^ at the PBE0/DEF2-TZVPP level. The calculated S_0_ → S_1_ transition at 459 nm is shown and is 92% HOMO → LUMO.

To assign the optical transitions that support photocatalysis, TD-DFT calculations were performed on complexes 1^H^ and 3^C(O)OMe^, as representative photoinactive and photoactive complexes, respectively. The calculated transitions are in good agreement with the corresponding experimental UV-vis spectra (Fig. 2a) and show that the dominant orbital contribution to the lowest energy transition is HOMO → LUMO (>90% for both complexes). Examination of the orbital contributions to the HOMO demonstrate that it is localised on the SAP ligand, including the imine π-bond. The LUMO contains a ∼30% contribution from the Mo-based orbitals as well as oxo π*-orbital character (Fig. 2b). Therefore, the lowest energy excitation is probably best considered to be an LMCT §§A triplet state lying *ca* 75 kJ mol^−1^ lower in energy that the S_1_ state was also located and we cannot rule out a situation in which there is rapid intersystem crossing (<5 ns) and it is actually the triplet manifold that is responsible for the observed chemistry. Our calculations indicate that there is still significant Mo character in the highest occupied SOMO in a similar vein to the S_1_ state so is consistent with the proposed mechanism which has light-induced reduction of the metal centre as a key step..

**Fig. 3:**
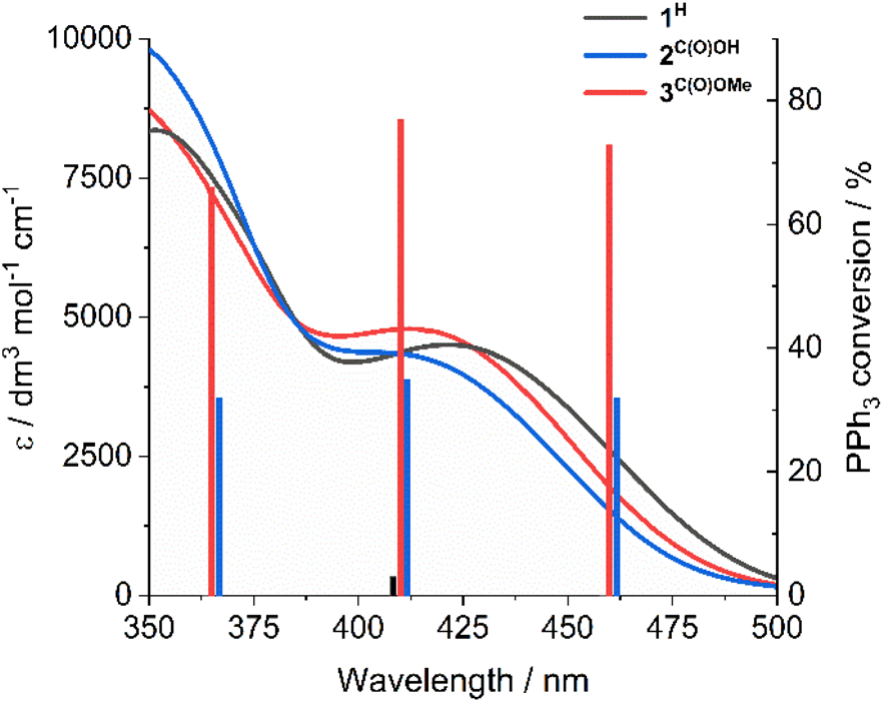
UV-vis spectra of 1^H^, 2^C(O)OH^, and 3^C(O)OMe^ and PPh_3_ conversion as a function of irradiation wavelength. Photocatalytic conditions: catalyst (1 mM in DMSO-d_6_), 300 equivalents PPh_3_, 3 h, under N_2_.

Notably, SAP ligand substituent orbitals contribute little to either the HOMO or LUMO of 1^H^ and 3^C(O)OMe^ and do not induce significant changes to the electron density of metal-imine and metal-oxo orbitals. Photocatalysis was also investigated at 365 and 460 nm for complexes 2^C(O)OH^ and 3^C(O)OMe^ and showed little variation between OPPh_3_ conversion and irradiation wavelength across the LMCT band (Fig. 3). This is in accordance with Kasha's rule,^66^ since the observed photoactivity of the complexes results from the lowest-lying excited state and is independent of the irradiation wavelength.

Steady-state and time-resolved fluorescence spectroscopies were used to compare the photoexcited states of 1^H^, 3^C(O)OMe^ and SAP ligands H_2_L1^H^ and H_2_L3^C(O)OMe^, respectively. The steady state emission spectra of the complexes and ligands both show a weak emission band at 525 nm with solutions of 1^H^ and 3^C(O)OMe^ exhibiting a lower emission by over an order of magnitude compared to H_2_L1^H^ and H_2_L3^C(O)OMe^, respectively (see Fig. S52, ESI†). Time-resolved photoluminescence showed these low intensity emission bands of complexes and ligands also have similar lifetimes of 3–5 ns (see Fig. S53 and Table S17, ESI†) which, for 1^H^ and 3^C(O)OMe^ are also unaffected by the addition of PPh_3_ (see Fig. S54, ESI†). We cannot exclude that for 1^H^ and 3^C(O)OMe^ emission could be due to residual dissociated ligand. Regardless, it is clear that 1^H^ and 3^C(O)OMe^ are essentially non-emissive and the excited state lifetimes of the emissive states are short relative to intermolecular reaction rates. Collectively, the TD-DFT, UV-vis, and fluorescence spectroscopies, and wavelength independence of photocatalysis, indicate that initial photon absorption does not account for the marked difference in reactivity of complexes 1^H^ and 3^C(O)OMe^ and that OAT is not attributable to an emissive excited state.

### Half-catalytic cycle OAT reactivity studies

Oxidation of PPh_3_ was also investigated under non-catalytic, stoichiometric conditions in the absence of DMSO-d_6_ as the oxygen atom donor. Reaction between 3^C(O)OMe^ and 5 equivalents of PPh_3_ in DMF-d_7_ (a solvent unreactive under these conditions) was monitored using ^1^H and ^31^P NMR spectroscopy. The addition of PPh_3_ to 3^C(O)OMe^ does not affect ^1^H and ^31^P NMR signals of either 3^C(O)OMe^ or PPh_3_, indicating that no significant interaction is detectable in the ground state before irradiation and was further supported by ^1^H and ^31^P NMR spectra of 3^C(O)OMe^ obtained in the presence of increasing equivalents of PPh_3_ (see Fig. S55, ESI†). On irradiation, the samples change colour from yellow to dark green within minutes (see Fig. S58, ESI†), with gradual loss of ^1^H NMR signals associated with PPh_3_ and 3^C(O)OMe^, and growth of signals due to OPPh_3_ and broader peaks upfield of 3^C(O)OMe^ associated with a new SAP-containing species (see Fig. S59b, ESI†). No signals indicating coordination of PPh_3_ or OPPh_3_ to the Mo-complex are observed. In comparison, catalytic reactions in DMSO-d_6_ as the solvent (Table 1), remain yellow throughout the course of the reaction, presumably because the large excess of DMSO-d_6_ leads to fast re-oxidation of the dark green SAP-containing intermediate, thereby preventing its accumulation.

Closure of the catalytic cycle, requiring reoxidation of the catalyst by DMSO-d_6_, was subsequently investigated by first irradiating a mixture of 3^C(O)OMe^ and 5 equivalents of PPh_3_ in DMF-d_7_ for 21 h, and then adding 10 equivalents of DMSO-d_6_ in the dark at 45 °C which is the reaction temperature during irradiation. After 2 h at 45 °C, *ca* 75% of 3^C(O)OMe^ was regenerated indicating that the reoxidation of the catalytic intermediate is a thermally driven process (see Fig. S60b, ESI†). An analogous experiment performed under irradiation gave 50% regeneration of 3^C(O)OMe^ (see Fig. S59b, ESI†) which is lower due to continued photocatalytic turnover in the presence of excess PPh_3_.

**Fig. 4:**
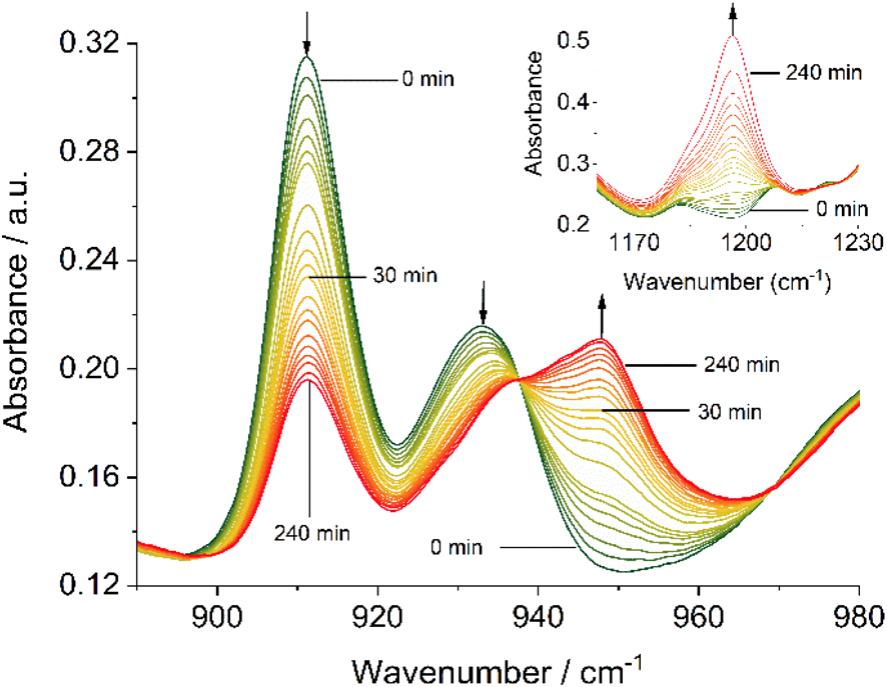
Solution-IR spectra in the region of the Mo=O stretching bands (890–900 cm^−1^) of 3^C(O)OMe^ (50 mM in DMF), 10 equivalents PPh_3_ and 50 equivalents DMSO-d_6_ in DMF irradiated at 410 nm as a function of time; insert–O=P stretching band (1160–1230 cm^−1^) of the same sample.

Complementary IR spectroscopic studies with 1^H^ and 3^C(O)OMe^ in solution with 10 equivalents of PPh_3_ and 50 equivalents of DMSO-d_6_ in DMF allowed monitoring of the characteristic symmetric and asymmetric stretch vibrations at *ca* 910 and 930 cm^−1^, attributable to *cis*-Mo(O)_2_,^57,60^ and a band at 1196 cm^−1^ assigned to O=P stretch vibration of the free OPPh_3_ product,^67,68^ respectively. On irradiation at 410 nm, the generation of a new species occurs with a single band at 948 cm^−1^ characteristic of a Mo(O) species,^69^ with concomitant generation of OPPh_3_ (Fig. 4), confirming OAT has occurred. On standing in the dark for 20 hours under a constant flow of nitrogen, significant regeneration of 3^C(O)OMe^ is observed (see Fig. S65, ESI†).

**Scheme 4:**
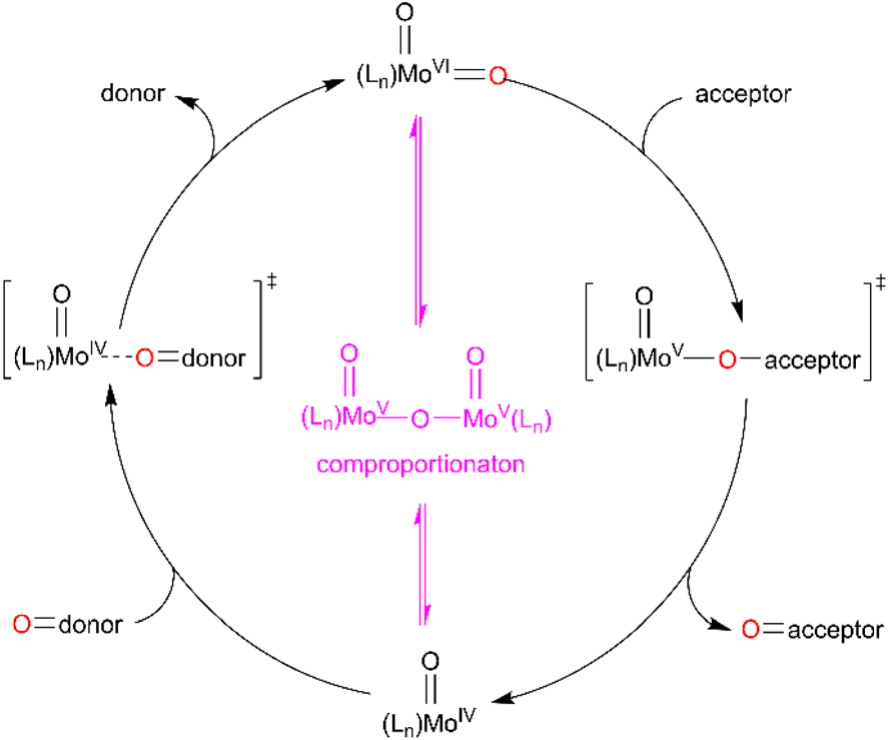
General OAT mechanism between an oxygen-atom acceptor and an oxygen-atom donor *via* Mo^VI^/Mo^V^/Mo^IV^ redox cycling.

Both, the formation of the Mo(O) intermediate and the regeneration of *cis*-Mo^VI^(O)_2_ (3^C(O)OMe^) give rise to clear isosbestic points. For conventional ‘thermal’ OAT catalysed by *cis*-Mo^VI^(O)_2_ complexes it has been reported previously that the putative Mo^IV^(O) intermediates can comproportionate with remaining *cis*-Mo^VI^(O)_2_ complexes to give stable, and often catalytically inert [(O)Mo^V^–O–Mo^V^(O)] oxo-bridged complexes.^40–44,70^ Here, after photo-activation of 3^C(O)OMe^, the presence of a single band at 948 cm^−1^ and isosbestic points are consistent with formation of a monomeric Mo^IV^(O) intermediate or rapid comproportionation to a [(O)Mo^V^–O–Mo^V^(O)] complex in equilibrium with Mo^IV^(O), as illustrated in Scheme 4, that reacts too rapidly to be observed.

The IR-solution spectrum of 1^H^ showed no change on irradiation, confirming that OAT from 1^H^ to PPh_3_ does not occur (see Fig. S67 and S68, ESI†). Collectively, the ^1^H, ^31^P and IR-solution data show that OAT occurs under irradiation to give a Mo(O) complex intermediate and subsequent regeneration of *cis*-Mo(O)_2_ occurs thermally. Furthermore, the lack of catalytic turnover for 1^H^ is not due to stoichiometric OAT and subsequent formation of a catalytically inactive [(O)Mo^V^–O–Mo^V^(O)] oxo-bridged bimetallic complex.^71^

### Phosphine and sulfoxide scope and equimolar catalytic reactions

There is a clear opportunity to develop synthetically useful OAT chemistry using these systems and, as an example, the deoxygenation of sulfoxides is considered of synthetic utility for sulfide formation.^72–74^ For this purpose, the reaction solvent was changed from DMSO, a reactive solvent, to acetone, which is unreactive under these conditions. Photocatalytic OAT also occurs between equimolar ratios of a range of other phosphines and sulfoxides (Tables 2, S18 and S19, ESI†). Aryl, alkyl and substituted derivatives are tolerated, and the studied sulfoxides and phosphines all exhibited OAT with generation of the respective phosphine oxide and sulfide, showing complete selectivity for the OAT reaction. For example, using 3^C(O)OMe^ and 300 equivalents of PPh_3_ and 300 equivalents of *p*-tolyl sulfoxide in acetone gives 97% conversion in 48 hours.

**Table 2:** Substrate conversions for selected sulfoxides and phosphines under benchmark conditions^a^

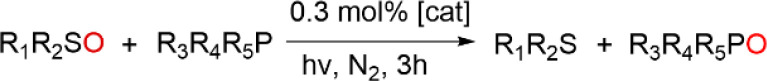			
Entry	Sulfoxide	Phosphine	Conversion^b^ %^c^, (%)^d^
1	R_1_ = R_2_ = Me	R_3_ = R_4_ = R_5_ = Ph	14, (20)
2	R_1_ = R_2_ = CH_2_Ph	R_3_ = R_4_ = R_5_ = Ph	21^d^
3	R_1_ = R_2_ = 4-Me-Ph	R_3_ = R_4_ = R_5_ = Ph	46, (46)
4	R_1_ = Me; R_2_ = Ph	R_3_ = R_4_ = R_5_ = Ph	35^e^
5	R_1_ = Me; R_2_ = 4-Me-Ph	R_3_ = R_4_ = R_5_ = Ph	44, (42)
6	R_1_ = Me; R_2_ = 4-Br-Ph	R_3_ = R_4_ = R_5_ = Ph	36, (39)
7	R_1_ = Me; R_2_ = 4-MeO-Ph	R_3_ = R_4_ = R_5_ = Ph	46, (45)
8	R_1_ = R_2_ = Me	R_3_ = R_4_ = Me; R_5_ = Ph	30, (36)
9	R_1_ = R_2_ = Me	R_3_ = Me; R_4_ = R_5_ = Ph	35, (42)
10	R_1_ = Me; R_2_ = 4-Me-Ph	R_3_ = R_4_ = R_5_ = 4-F-Ph	14^e^
11	R_1_ = Me; R_2_ = 4-Me-Ph	R_3_ = R_4_ = R_5_ = 4-MeO-Ph	32, (47)
12	R_1_ = Me; R_2_ = 4-Me-Ph	R_3_ = R_4_ = R_5_ = 4-CF_3_-Ph	40^e^

a General condition: 1 mM of 3^C(O)OMe^ in acetone-d_6_, 300 mM sulfoxide and 300 mM phosphine, 410 nm, 3 h, under N_2_.

b Conversions determined by ^1^H NMR spectroscopy.

c Conversion of sulfoxide to sulfide.

d Conversion of phosphine to phosphine oxide in brackets.

e Determination of phosphine conversion prevented by overlapping signals.

### Mechanism of the photocatalytic OAT reaction

The foregoing observations can be used to propose a catalytic mechanism and identify some notable features. Conventional thermal OAT using bio-inspired *cis*-Mo(O)_2_ complexes^75^ generally occurs *via* the nucleophilic attack of oxygen atom acceptor on a Mo-oxo π* orbital (Scheme 4), which results in concomitant weakening of the Mo-oxo and strengthening of the acceptor-oxo bond. In these reported systems Hammett relationships for catalyst ligands indicate that EWGs reduce localisation of negative charge in the transition state, thereby increasing the rate of the reaction.^41,42,50,60^ In comparison, complexes 1^H^–7^CF3^ show similar, but non-linear trends (Table 1, Fig. S48†) with H, F, OMe, and *t*Bu ligand substitution giving inactive catalysts, whereas the EWGs, C(O)OMe, C(O)OH and CF_3_ support a broad range of OAT activity.

The similarity of the electronic absorption spectra (see Fig. S31, ESI†) and absorption coefficients at 410 nm for 1^H^–7^CF3^ indicate that initial photon absorption does not account for the differences in the observed activity. In addition, similar emission data for ligands and complexes in the presence and absence of substrate (see Table S17, Fig. S52–S54, ESI†) suggest that the emissive excited states that are detectable do not mediate OAT. Nevertheless, ^1^H, ^31^P NMR and IR spectroscopies clearly show that, for inactive complexes, catalytic OAT fails because initial OAT to phosphine does not occur. There is no evidence for an inert intermediate after initial OAT that prevents catalytic turn over. Indeed, IR-solution spectroscopy clearly shows that SAP ligands with sufficiently electron-withdrawing substituents support the generation of a Mo(O) intermediate under irradiation which undergoes thermal regenerative OAT.

**Scheme 5:**
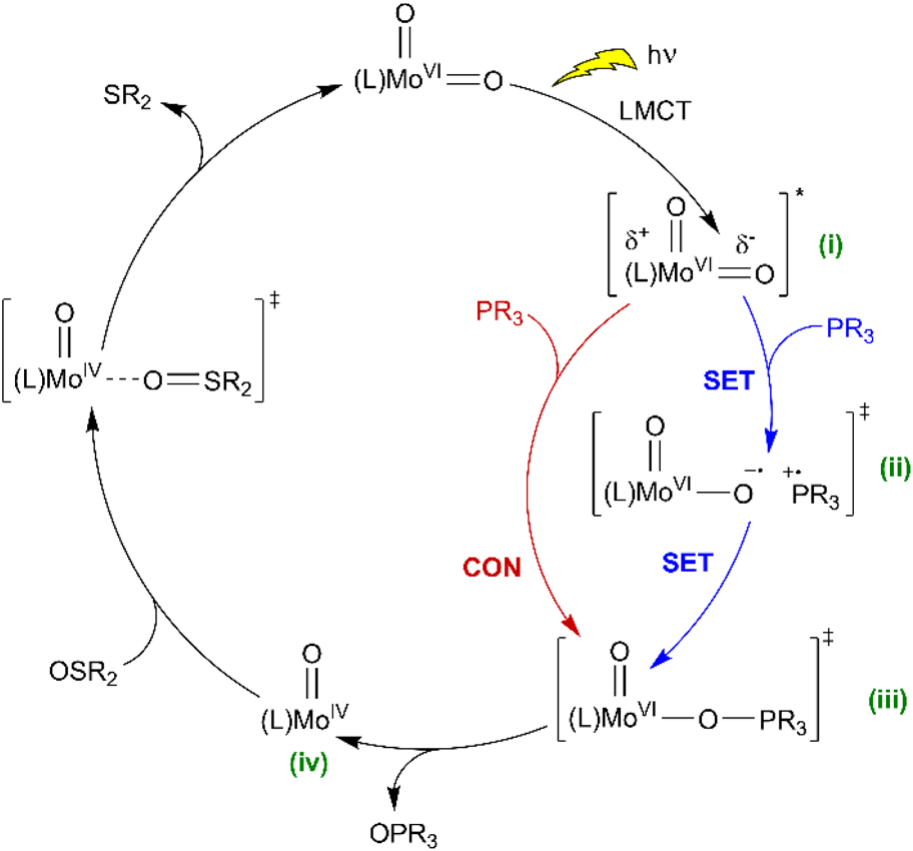
Proposed catalytic mechanisms for light-activated OAT between phosphine and sulfoxide substrates, catalysed by photoactive *cis*-Mo^VI^(O)_2_ complexes (SET = single electron transfer, CON = concerted reaction, comproportionation as shown in Scheme 4 also conceivable).

Based on the above observations, a mechanism is proposed (Scheme 5) where the initial photon absorption leads to the generation of a non-emissive excited state (i). Subsequently OAT occurs *via* either (ii) SET followed by a radical rebound pathway *via* caged-radicals, as proposed for some metal-oxyl systems,^76–78^ or alternatively, a concerted reaction to give (iii) *via* the nucleophilic addition of phosphine. Both pathways would be promoted by EWGs on the SAP ligands, with the SET pathway potentially less sensitive to phosphine steric features. Subsequently, elimination of OPPh_3_ from (iii) gives Mo(O) (iv) which undergoes thermal regenerative OAT from a sulfoxide donor. In comparison, recently reported photoactive W-dioxo complexes are proposed to photocatalyse *via* photo-induced SET pathways from an emissive state,^51^ similar to the well-established Ru- and Ir-bipyridyl photoredox catalysts,^8,72^ where substrate binding to the metal centre is not required, and radical-based chemical reactions are initiated *via* outer sphere electron transfer. Irradiation of 3^C(O)OMe^ in DMSO in the presence of potential sacrificial electron donors,^79^ such as triethylamine (TEA), *N*,*N*-dimethylaniline (DMA), and 1, 3-methyl-2-phenylbenzimidazoline (BIH) showed a decrease in the amount of catalyst and a complex array of products (Fig. S86†). These observations are indicative of electron transfer and suggest that complex photoredox chemistry is occurring that leads to the decomposition of the catalyst. To try and differentiate between concerted and SET pathways, a radical scavenging experiment was undertaken. Since TEMPO is unsuitable as a radical scavenger because it reacts directly with catalyst 3^C(O)Me^ under irradiation (see Fig. S87 and S88, ESI†), we selected 2,4,6-tri-*tert*-butylphenol (TTBP)^80,81^ as an alternative. A control experiment showed that on irradiation of a mixture of 3^C(O)Me^ and TTBP in DMSO no change was observed in the ^1^H NMR spectrum (see Fig. S89, ESI†). On addition of PPh_3_ catalysis occurs giving 57% conversion compared to 69% for a parallel control reaction in the absence of TTBP (see Fig. S90, ESI†). The addition of electrolyte (250 mM [*n*Bu_4_N][PF_6_]) to facilitate cage escape of any radicals formed *via* SET decreased the conversion further to 53% (see Fig. S96 and S97, ESI†). These observations could be suggestive of a SET mechanism in which the putative [PR_3_]˙^+^ radical does not significantly dissociate from the catalyst. The observed decrease in conversion, however, is not pronounced enough to allow a conclusive differentiation between the concerted and radical-rebound (caged-radical) pathways shown in Scheme 5. Similarly, catalysis in the presence of styrene and cyclohexene as putative radical traps also did not affect conversion of PPh_3_ to OPPh_3_ (see Fig. S98–S101†). In addition, we note the insensitivity of catalysis to dioxygen (Table 1, entry 9) suggesting that free [PR_3_]˙^+^ is not present.

Conceivably, prior coordination of PR_3_ could be followed by intramolecular migration of PR_3_*cis* to Mo^V^(O) which has been proposed for related stoichiometric reactions^37^ although in these cases spectral changes are observed on mixing PR_3_ with Mo^V^ which we do not observe here. Collectively, these observations suggest that the putative [PR_3_]˙^+^ radical does not significantly dissociate from (ii) but the data do not conclusively differentiate between the concerted and radical rebound (caged-radical) pathways shown in Scheme 5.

Of perhaps more direct relevance are recent hydrogen atom transfer (HAT) reactions to [*cis*-Mo(O)_2_X_2_(*t*Bu-bipy)] (where X = F, Cl, Br) complexes where a LMCT excited state is proposed to mediate stoichiometric HAT *from* a hydrocarbon substrate *to* an oxo ligand *via* a step-wise SET-proton transfer or concerted pathways.^38^ In contrast LMCT here promotes bimolecular radical oxo-ligand transfer *to* a phosphine substrate. This reactivity is analogous to unimolecular radical dissociation of ligands with a single M–L bond such as halides *via* inner sphere electron transfer (Scheme 1).^17^

## Conclusions

A novel class of photoactive Mo-based OAT catalysts was synthesised in two steps from inexpensive, commercially available precursors. The catalysts are tolerant to both water and oxygen. Compared to the established Mo-oxo catalytic chemistry of biological, bio-inspired, and heterogeneous systems, there are clear mechanistic differences for the photocatalytic cycle proposed here, which arise from photoactivation *via* LMCT. In contrast to most homogeneous photocatalytic redox systems, photo-induced reactivity does not arise from a long-lived emissive state, but rather from a non-emissive LMCT excited state. In common with thermal OAT chemistry mediated with *cis*-Mo(O)_2_ complexes, EWGs promote activity indicating the accumulation of negative charge in the rate-limiting transition state. A further important feature of the SAP complexes studied here is that under the chosen experimental conditions the formation of inert [(O)Mo^V^–O–Mo^V^(O)] complexes is not restricting catalytic turnover, which is sustained with remarkable catalyst stability, allowing equimolar OAT reactions with very low catalyst loading compared to many photoredox reactions. A unique feature of the work described here is the coupling of photoactivated and thermally activated OAT steps to establish a catalytic cycle. There is considerable scope to further understand mechanistic details of these LMCT-induced reactions that could guide future atom transfer catalyst developments to expand the reactivity beyond that described here, which is the focus of current studies.

## Author contributions

R. E. D. and A.-K. D.-K. directed the project. T. D., L. G., and S. S. carried out compound synthesis, characterisation, and respective data analysis. A. C. W. collected, solved, and refined the crystal data. T. D. and L. G. carried out photocatalytic testing of the reported complexes. L. G. and J. M. L. carried out computational calculations. L. G. and B. P. carried out FTIR-monitoring of non-catalytic reactions and analysed the respective data. T. D. tested the substrate scope. L. G. carried out sacrificial electron donor and radical scavenging experiments, catalysis with electrolyte and kinetic experiments with different phosphines. T. D., L. G., R. E. D. and A.-K. D.-K. co-wrote the original draft. All authors contributed to the editing of the manuscript. R. E. D. and A.-K. D.-K. were responsible for funding acquisition and project administration.

## Conflicts of interest

The authors declare no conflict of interest.

## Supplementary Material

SC-015-D4SC02784A-s001

SC-015-D4SC02784A-s002

## Data Availability

All supporting data has been uploaded as part of the ESI.†§
