# Supplementary material for: Ligand-to-metal charge transfer facilitates photocatalytic oxygen atom transfer (OAT) with cis-dioxo molybdenum(vi)-Schiff base complexes
Source: Chem Sci. 2024 Sep 6;15(39):16186–95. doi: 10.1039/d4sc02784a (PMC11396016; doi:10.1039/d4sc02784a)
Supplement: SC-015-D4SC02784A-s001 [file SC-015-D4SC02784A-s001.pdf]

Supplementary Information (SI) for Chemical Science.
This journal is © The Royal Society of Chemistry 2024

Supporting Information

Ligand-to-metal charge transfer facilitates photocatalytic oxygen atom transfer (OAT) with *cis*-dioxo molybdenum (VI)-Schiff base complexes

Thorsten Dreher,[a‡] Lukas Geciauskas,[a‡] Samuel Steinfeld,[a] Barbara Procacci,[a] Adrian C. Whitwood,[a] Jason M. Lynam,[a] Richard E. Douthwaite,*[a] Anne-K. Duhme-Klair*[a]

[a] Department of Chemistry, University of York, Heslington, YO10 5DD, York, United Kingdom.

‡ These authors have contributed equally to this work.

* Corresponding author e-mail: richard.douthwaite@york.ac.uk; anne.duhme-klair@york.ac.uk.

Contents

Experimental Procedures 3
General 3
Synthesis procedures 7
Synthesis of precursor compounds 7
General method for SAP ligand synthesis 9
General methods for complex synthesis 13
^{1}H and $^{13}C\{^{1}H\}$ NMR spectra of ligands and complexes 19
UV-visible spectra (in DMSO) of complexes 1^{H} – 7^{CF3} 33
Crystal structures of complexes 1^{H}_{DMSO}, $3^{C(O)OMe}_{DMSO}$, 4^{F}_{THF}, 6^{OMe}, and 7^{CF3}_{H2O} 34
Photocatalytic testing of complexes 1^{H} – 7^{CF3} 39
^{1}H NMR spectra of photocatalytic reactions 39
Photocatalytic testing of complexes 1^{H}, $2^{C(O)OH}$, $3^{C(O)OMe}$, 7^{CF3} as a function of time. 46
Photocatalytic control reactions 50
Hammett analysis of substituent effects on substrate conversion for complexes 1^{H} – 7^{CF3} 51
Kinetics of reactions between $3^{C(O)OMe}$ or 7^{CF3} and PPh_3, $P(p\text{-}F\text{-}Ph)_3$, and $P(p\text{-}CF_3\text{-}Ph)_3$ in DMSO-d_6 52
Computational calculations 55
Geometry optimization of complexes 1^{H} and $3^{C(O)OMe}$ 55
Time-dependent density functional theory (TD-DFT) calculations of complexes 1^{H} and $3^{C(O)OMe}$. 61
Photocatalysis at different wavelengths 65
Steady state emission spectroscopy 65
Time-resolved photoluminescence spectroscopy 66
Ground state interactions between $3^{C(O)OMe}$ or 7^{CF3} and PPh_3 – ^{1}H and ^{31}P NMR spectra 68
Non-catalytic reactions – Mo(O)-intermediate generation 71
Detection by ^{1}H NMR spectroscopy 71
In operando solution FTIR spectroscopy. 74
Phosphine and sulfoxide substrate scope 79
Reactions between $3^{C(O)Me}$ and sacrificial electron donors TEA, DMA and BIH 97
Radical scavenging experiments with TEMPO 98
Reactions between PPh_3 and DMSO-d_6 catalysed by $3^{C(O)OMe}$ in the presence of TTBP and $[nBu_4N][PF_6]$ 100
Reactions between PPh_3 and DMSO-d_6 catalysed by $3^{C(O)OMe}$ in the presence of alkenes 110

References 114

Experimental Procedures

General

Materials. Chemicals and solvents were obtained from commercial suppliers (Acros Organics, Fluka, Sigma-Aldrich, Fluorochem, Alfa Aesar, Fischer Scientific, Apollo Scientific, Tokyo Chemical Industry) and used without further purification unless stated otherwise.

NMR Spectroscopy. ^{1}H, ^{13}C {^{1}H} and ^{31}P {^{1}H} NMR spectra were recorded on a Jeol ECS400D 400 MHz spectrometer (^{1}H NMR 400 MHz, ^{13}C NMR 100.6 MHz ^{31}P NMR 161.9 MHz), a Bruker AV500b 500 MHz spectrometer (^{1}H NMR 500 MHz, ^{13}C NMR 125.75, ^{31}P NMR 202.4 MHz) and a Bruker AV600 600 MHz spectrometer (^{1}H NMR 600 MHz, ^{13}C NMR 150.9 MHz, ^{31}P NMR 242.9 MHz). The assignment of the spectra was aided by DEPT135, COSY, HMBC and HSQC experiments. TopSpin and MestReNova software packages were used for processing and analysis of spectra. Chemical shifts are quoted as ppm referenced to residual solvent. Coupling constants are rounded to the nearest 0.5 Hz. The following abbreviations for multiplicity are used: s for singlet, d for doublet, t for triplet, q for quartet, dd for doublet of doublet, td triplet of doublet, ddd for doublet of doublet of doublets, m for multiplet, br for broad.

Chromatography. Analytical thin-layer chromatography (TLC) was performed using Merck silica gel 60 aluminium-supported plates with specified solvent systems and visualised under UV light. Flash column chromatography was carried out using Fluka Silica gel, pore size 60 Å, 220–440 mesh, 35–75 µm.

ATR-FTIR Spectroscopy. Were recorded of solid samples on a Perkin Elmer Spectrum Two in the region of 400-4000 cm^{-1}.

Elemental Analysis. Was carried out by Dr S. Hicks, L. Duff, Dr G. McAllister on an Exeter Analytical Inc. CE-440 analyser.

Mass Spectrometry. was carried out by K. Heaton, Dr. R. Cercola and A. Lopez on Waters GCT Premier mass spectrometer (LIFDI) and Bruker compact® time of flight mass spectrometer (ESI). Masses for molybdenum were calculated based on ^{98}Mo.

Melting Points. Were recorded on a Stewart Scientific SMP3 melting point apparatus.

UV-vis Spectroscopy. Were recorded on a Shimadzu 1800 in the range of 270 to 700 nm (DMSO), using quartz cuvettes of 1 cm pathlength.

Steady State Emission Spectroscopy. Were recorded on a Hitachi F-4500 spectrometer in acetone or DMSO and under nitrogen with the following parameters: excitation at 410 nm, excitation slit width of 10 nm, emission slit width of 20 nm, 950 V photomultiplier tube (PMT) voltage, scan speed of 240 nm/min, pathlength 1 cm; spectra were corrected for instrument response. All measurements in a set of experiments were performed at an equal absorbance of 0.1 at 410 nm or at equal concentration.

Time-Resolved Photoluminescence Spectroscopy. Were recorded on an Edinburgh Instruments FLS980 spectrometer in acetone or DMSO and under nitrogen with the following parameters: excitation at 472.8 nm with a EPL-470 laser source, excitation slit width 0.03 nm, emission slit width 10 nm, range 0 - 200 ns. All measurements in a set of experiments were performed at an equal absorbance of 0.1 at 473 nm or at equal concentration. Data analysis and fitting were performed with the FLS980 software. Samples were prepared and analysed under N_2 atmosphere.

Single Crystal X-ray Diffraction. Was performed by Dr. A. C. Whitwood. Diffraction data were collected at 110 K on an Oxford Diffraction SuperNova diffractometer with Cu-K_{α} radiation (λ = 1.54184 Å) using a HyPix-6000HE detector. The crystal was cooled with an Oxford Instruments Cryojet. Diffractometer control, data collection, initial unit cell determination, frame integration and unit-cell refinement were carried out with

CrysAlisPro.[1] Face-indexed absorption corrections were applied using spherical harmonics, implemented in SCALE3 ABSPACK[2] scaling algorithm within CrysAlisPro. OLEX2[3] was used for overall structure solution, refinement and preparation of computer graphics and publication data. Within OLEX2, the algorithms used for structure solution were "ShelXT dual-space".[4] Refinement by full-matrix least-squares used the SHELXL[5] algorithm within OLEX2.[3]

Computational calculations. Ground state (S_0) and lowest triplet state (T_1) geometries of complexes $\mathbf{1}^{\mathbf{H}}$ and $\mathbf{3}^{\mathbf{C(O)OMe}}$ were optimised using the BP86 functional (for T_1 – unrestricted BP86),[6,7] DEF2SVPP hybrid basis set,[8,9] and self-consistent reaction field (SCRF)[10] description for DMSO solvent environment, as implemented in the Gaussian 16W software package.[11] All geometry optimizations were performed in C1 symmetry. Subsequent vibrational frequency analysis was used to confirm that each stationary point was a minimum on the potential energy surface. The vertical transition energies of complexes $\mathbf{1}^{\mathbf{H}}$ and $\mathbf{3}^{\mathbf{C(O)OMe}}$ were computed employing TD-DFT as implemented in the Gaussian 16W software package.[11] To explore the best parameters for model fit with the experimental data, the TD-DFT calculations were performed using four different hybrid functionals (PBE0; CAM-B3LYP; M062X; wB97XD).[12–15] Tam-Dancoff approximation was used for the calculations, alongside DEF2TZVPP hybrid basis set,[8,9] self-consistent reaction field (SCRF)[10] description for DMSO solvent environment, and optimised geometry input from optimisation calculations. To compare the relative energies of S_0, T_1 and S_1 states of $\mathbf{1}^{\mathbf{H}}$ and $\mathbf{3}^{\mathbf{C(O)OMe}}$, as well as to determine the molecular orbital compositions for S_0 and T_1 states, single point energy calculations and population analysis calculations were carried out using PBE0 functional (for T_1 – unrestricted PBE0), DEF2TZVPP hybrid basis set,[8,9] self-consistent reaction field (SCRF)[10] description for DMSO solvent environment, and optimised geometry input from optimisation calculations.

Photolysis Apparatus. Reactions were performed in silica NMR tubes sealed under nitrogen with a Young's Teflon stopcock. The photoreactor (Fig. S1) holds three NMR tubes, positioned equidistantly from the LED light source. Each tube is inserted by 8 cm ensuring the sample is contained within the reactor and the housing is coated internally with reflective material. LEDs (LED Engin, California, USA) were powered by a constant current source of 700 mA per LED. Each LED showed similar irradiance of around 20 mW cm^{-2}, which was measured using a power meter at 4 cm.

Photocatalytic Reactions. Reactions were prepared in a glove box under N_2 atmosphere, solvents were dried over 3 Å molecular sieves and degassed via 3 freeze-pump-thaw cycles. Solutions of the samples in DMSO-d_6, acetone-d_6, and DMF-d_7 were prepared containing appropriate amounts of phosphine, catalyst, and any additional compounds, and 700 µL – 1000 µL portions were then transferred using a syringe into a Youngs NMR tube (borosilicate glass). The NMR tubes were inserted into the photoreactor (Fig. S1) of selected wavelength, and irradiated for 3 hours (or selected time intervals). The temperature upon irradiation was determined by immersing a temperature probe inside an NMR tube which was then irradiated inside the photoreactor used for the catalytic tests over 1 h. During this time the temperature stabilised at 43-45 °C. Reactions were monitored by ^{1}H and ^{31}P NMR spectroscopy and conversion/yield was measured by integrating appropriate ^{1}H NMR signals of phosphine/phosphine oxide or sulfoxide/sulfide:

$$\%\,Conversion = \frac{i(product)}{i(product) + i(starting\ material)} \times 100$$

Where i(x) is the integrated NMR signal corrected for the number of protons represented.

In Operando FTIR Spectroscopy. Sample preparation and cell assembly were carried out in a glove box under an N_2 atmosphere. DMF and DMSO-d_6 were degassed *via* 3 cycles of freeze-pump-thaw, and stored in a glove box over 3 Å molecular sieves. Solutions of samples containing appropriate amounts of catalyst, PPh_3, and DMSO-d_6 were prepared in DMF. On dissolution of all components, a 50 µL aliquot was added to the fluid FTIR cell (Harrick flow cell), and irradiated inside the IR instrument chamber (Fig. S2) with a blue LED (λ = 410 nm) from the base of the photoreactor (Fig. S1(f)). The reaction was monitored by comparing

absorbances at characteristic intensities for the starting catalysts (900-1000 cm^{-1} region) and $OPPh_3$ (1196 cm^{-1}). FTIR spectra were recorded using Bruker Vertex 70v FT-IR spectrometer, (2000–400 cm^{-1}) using N_2 purge, 25 µm spacer, BaF_2 windows, air background, 3.5 mm beam slits, 4 scans per spectrum, at 1 cm^{-1} resolution.

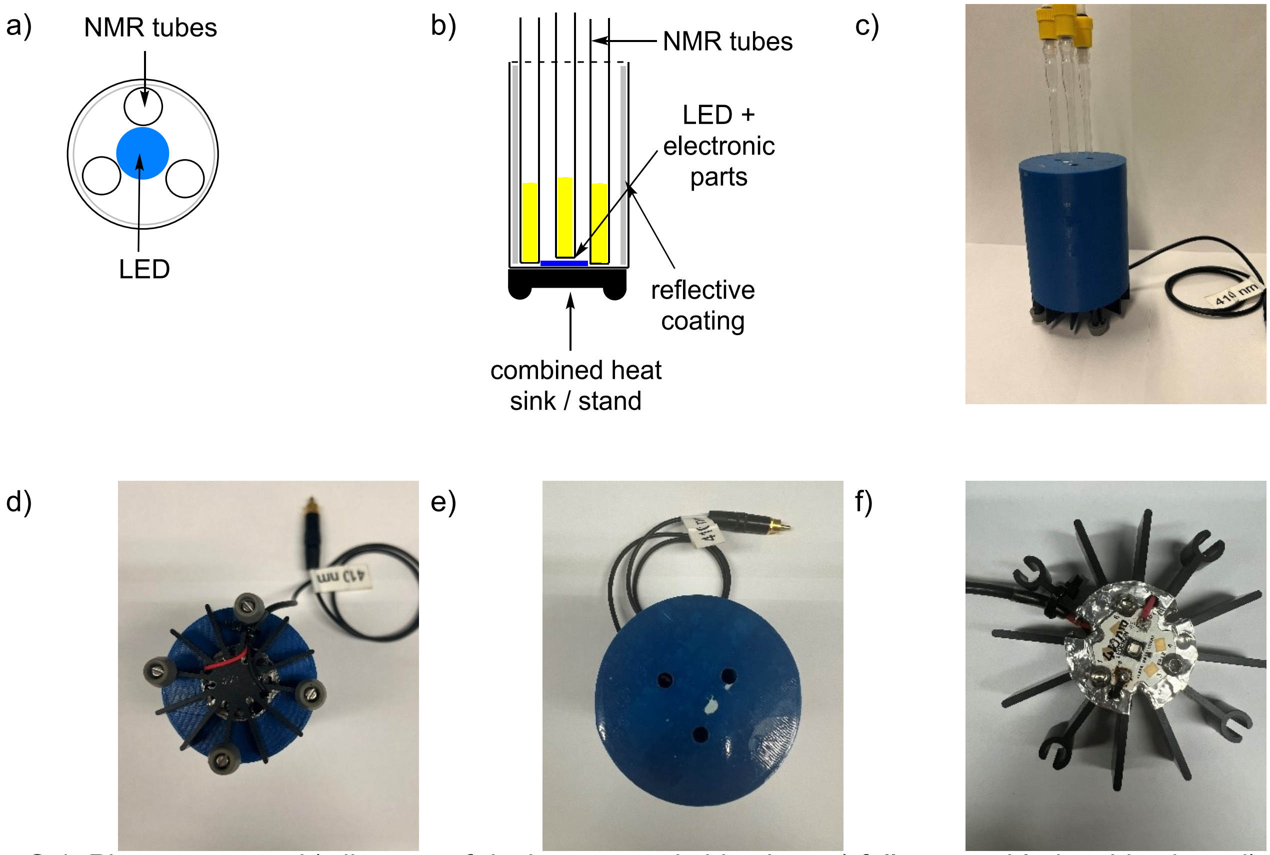

Figure S 1: Photoreactor; a-b) diagram of design, top and side view; c) fully assembled – side view; d) fully assembled – bottom view; e) fully assembled – top view; f) LED and electronic parts from the base of the photoreactor after removal from the blue casing.

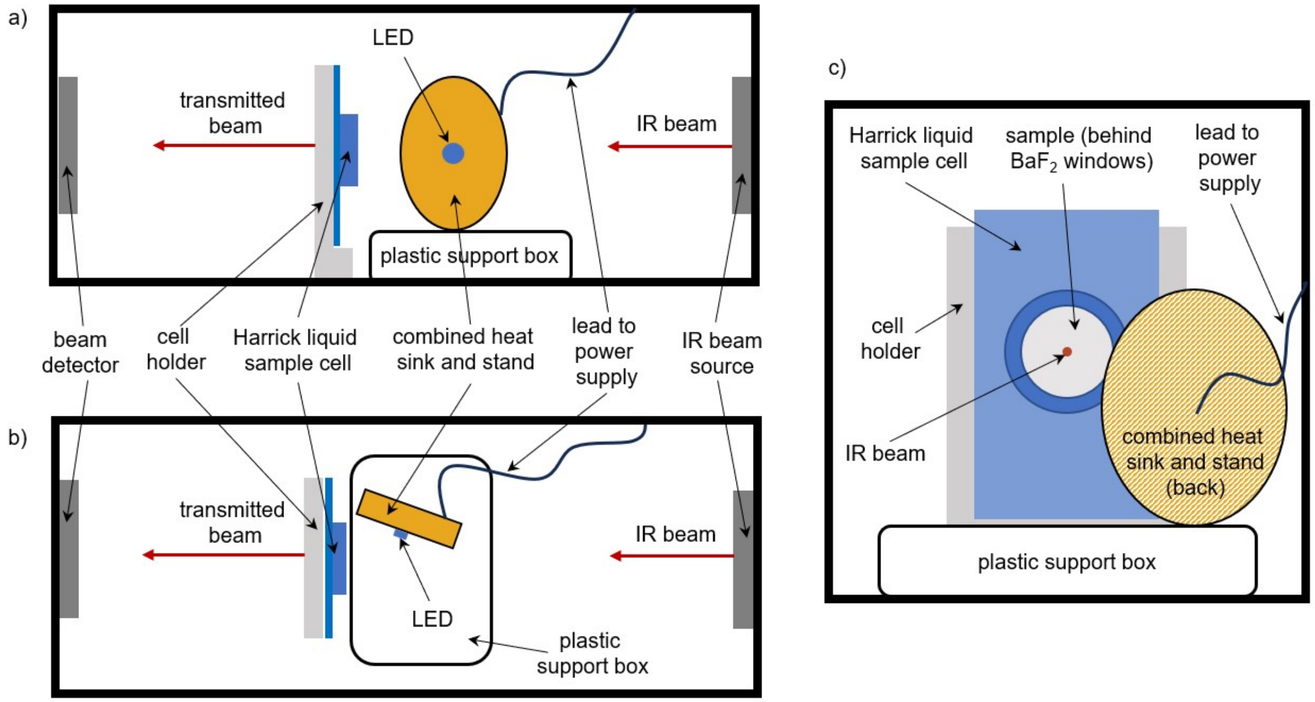

Figure S 2: Diagram of apparatusfor solution FTIR sample under irradiation; a) side view; b) top view; c) beam side view.

Synthesis procedures

Synthesis of precursor compounds

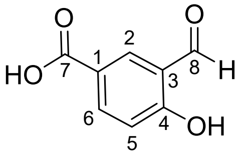

$C_8H_6O_4$, 166.13 g/mol

Formyl-4-hydroxybenzoic acid. Was prepared according to a literature procedure.[16,17] HMTA (3.06 g, 21.7 mmol) was dissolved in trifluoroacetic acid (9 mL) under nitrogen and added drop wise to a suspension of 4-hydroxybenzoic acid (3 g, 21.7 mmol) in trifluoroacetic acid (8 mL) under nitrogen. The mixture was heated to 90 °C for 4 h with stirring. After cooling to RT, the yellow mixture was poured into 4 M HCl (60 mL) and stirred for 22 h. The precipitate was isolated by filtration, washed with water (80 mL), and dried under vacuum to give a pale-yellow solid. Yield = 40% (1.206 g, 7.26 mmol). Analytical data are consistent with those reported in the literature.[17,18]

Mp.: 230°C

^{1}H NMR (400 MHz, DMSO-d$_6$, 25 °C): δ = 12.82 (1H, COOH), 11.49 (1H, OH), 10.29 (1H, H8), 8.23 (d, $^4J_{H,H}$ = 2.5 Hz, 1H, H2) 8.04 (dd, $^3J_{H,H}$ = 8.5 Hz, $^4J_{H,H}$ =2.5 Hz, 1H, H6), 7.08 (d, $^3J_{H,H}$ = 8.5 Hz, 1H, H5) ppm.

^{13}C{^{1}H} NMR (101 MHz, DMSO-d$_6$, 25 °C): δ = 190.6, 166.5, 164.1, 136.9, 130.7, 122.1, 122.0, 117.6 ppm.

HRMS (ESI): m/z calcd for $(C_8H_5O_4)^-$: 165.0193, found 165.0189.

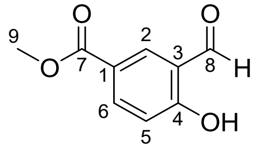

$C_9H_8O_4$, 180.16 g/mol

3-Formyl-4-hydroxybenzoic acid methyl ester. Was prepared according to a literature procedure.[19] Formyl-4-hydroxybenzoic acid (1 g, 6 mmol) was suspended in MeOH (10 mL). Whilst stirring, sulfuric acid (0.66 mL, 10.9 mmol) was added, and the mixture heated to reflux for 22 h. The resulting pale pink solution was cooled to RT, the volatiles removed under reduced pressure and the solid dissolved in chloroform (30 mL). After washing with saturated $NaHCO_3$ solution (20 mL) and drying over $MgSO_4$ the solvent was removed and the product dried *in vacuo*. Yield = 46% (500 mg, 2.78 mmol). Analytical data are consistent with those reported in the literature.[18]

Mp.: 76°C

^{1}H NMR (600 MHz, $CDCl_3$, 25 °C): δ = 11.22 (br s,1H, OH), 9.87 (s, 1H, H8) 8.22 (d, $^4J_{H,H}$ = 2.5 Hz, 1H, H2), 8.08 (dd, $^3J_{H,H}$ = 8.5 Hz, $^4J_{H,H}$ = 2.5 Hz, 1H, H6), 6.93 (d, $^3J_{H,H}$ = 8.5 Hz, 1H, H5), 3.85 (s, 3H, H9) ppm.

^{13}C{^{1}H} NMR (151 MHz , $CDCl_3$, 25 °C): δ = 196.3, 165.4, 164.9, 137.7, 136.0, 122.2, 120.0, 117.8, 52.2 ppm.

HRMS (ESI): m/z calcd for $[C_9H_7O_4]^-$: 179.0350, found 179.0347.

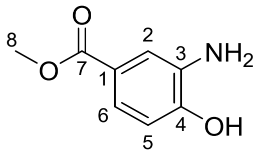

$C_8H_9NO_3$, 167.16 g/mol

3-Amino-4-hydroxybenzoic acid methyl ester. Was prepared according to a literature procedure.[20] 3-Amino-4-hydroxybenzoic acid (500 mg, 3.3 mmol) was dissolved in dry MeOH (20 mL) and sulfuric acid (0.4 mL, 6.61 mmol) added whilst stirring. The mixture was heated to reflux for 22 h and after cooling to RT, the volatiles were removed under reduced pressure and the remaining oil was dissolved in ethyl acetate (20 mL) and saturated $NaHCO_3$ solution (20 mL). The phases were separated and the aqueous phase extracted with ethyl acetate two more times (10 mL each). The combined organic phases were washed with water (10 mL), dried over $MgSO_4$ and the volatiles removed under reduced pressure. The product was obtained as a light brown solid. Yield = 78% (425 mg, 2.54 mmol). Analytical data are consistent with those reported in the literature.[20]

Mp.: 111-113°C

^{1}H NMR (600 MHz, $CDCl_3$, 25 °C) δ = 7.46 (d, $^4J_{H,H}$ = 1.5 Hz, 1H, H2), 7.42 (dd, $^3J_{H,H}$ = 8.0 Hz, $^4J_{H,H}$ =1.5 Hz, 1H, H6), 6.75 (d, $^3J_{H,H}$ = 8.0 Hz, 1H, H5), 3.86 (s, 3H, H8) ppm.

^{13}C{^{1}H} NMR (151 MHz, $CDCl_3$, 25 °C) δ = 167.3, 148.5, 131.5, 123.4, 122.2, 118.4, 114.6, 52.1 ppm.

HRMS (ESI): m/z calcd for $[C_8H_{10}NO_3]^+$: 168.0661, found 168.0655.

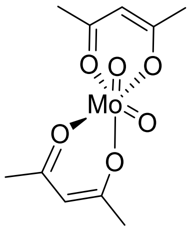

$C_{10}H_{14}MoO_6$, 326.18 g/mol

[Bis(acetylacetonato)dioxomolybdenum(VI)], MoO_2(acac)$_2$. Was prepared using an adapted literature method.[21] Sodium molybdate dihydrate (370 mg, 1.5 mmol) was dissolved in water (5.5 mL) and the solution acidified to pH 1 with 6 M HCl. While stirring, acetylacetone (470 µL, 4.5 mmol) was added and the mixture kept in the dark. After 4 h, a yellow solid precipitated which was isolated by filtration and washed with ice cold water. The crude yellow product (680 mg) was dried *in vacuo*. Yield = 81% (397 mg, 1.22 mmol). Analytical data consistent with the those reported in the literature.[22,23]

Mp.: 179-181°C

^{1}H NMR (400 MHz, $CDCl_3$, 25°C) δ = 5.7 (1H, HC=C, enol form), 3.7 (2H, CH_2, keto form), 2.2 (6H, CH_3, keto form), 2.1(6H, CH_3, enol form) ppm.

^{13}C{^{1}H} NMR (101 MHz, DMSO, 25°C) δ = 196.5, 184.2, 104.7, 27.6, 25.0 ppm.

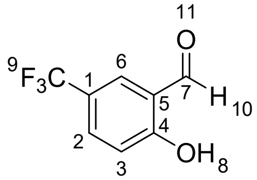

$C_8H_5O_2F_3$, 190.12 g/mol

2-Hydroxy-5-(trifluoromethyl)benzaldehyde. Was prepared according to a modified literature procedure.[17] HMTA (1.73 g, 12.3 mmol) in TFA (10 mL) was added dropwise to a stirred suspension of 4-(trifluoromethyl)phenol (2.00 g, 12.3 mmol) in TFA (5 mL). The mixture was heated to 100 °C and stirred for 4 h. After cooling down, HCl (30 mL, 4 M) was added and the mixture was stirred overnight at RT. The solution was extracted with $CHCl_3$ (4 × 50 mL), dried over $MgSO_4$, filtered and concentrated under reduced pressure. The crude product was purified by silica column chromatography (10:1 PE:EtOAc) to give the product as a pale solid. Yield = 27 % (642 mg, 3.3 mmol). Analytical data are consistent with those reported in the literature.[24]

R_f = 0.52 (SiO_2, 10:1 PE:EtOAc).

Mp.: 56–61 °C.

^{1}H NMR (400 MHz, DMSO-d$_6$): δ = 11.58 (s, 1H, H8), 10.30 (s, 1H, H10), 7.91 (d, $^4J_{H,H}$ = 3.0 Hz, 1H, H6), 7.84 (dd, $^3J_{H,H}$ = 9.0, $^4J_{H,H}$ 3.9 Hz, 1H, H2), 7.18 (d, $^3J_{H,H}$ = 8.5 Hz, 1H, H3) ppm.

^{13}C{^{1}H} NMR (101 MHz, DMSO-d$_6$): δ = 189.8 (C7), 163.4 (C4), 132.5 (d, J = 3.0 Hz, C2), 125.8 (d, $^3J_{C,F}$ = 4.0 Hz, C6), 124.2 (d, $^1J_{C,F}$ = 272.0 Hz, C9), 122.3 (C5), 120.1 (d, $^2J_{C,F}$ = 33.0 Hz, C1), 118.5 (C3) ppm.

^{19}F NMR (376 MHz, DMSO-d$_6$): δ = -60.30 (s, 3F, F9) ppm.

HRMS (ESI): m/z calcd for $[C_8H_4O_2F_3]^-$: 189.0169, found 189.0167.

General method for SAP ligand synthesis

Ligands were synthesised using an adapted literature procedure.[25] In a typical reaction, ca. 150 mg of appropriate aldehyde and an equimolar amount of the appropriate amine were suspended in MeOH or EtOH (dried over 3 Å molecular sieves) and heated to reflux with stirring for 4 h. After cooling to room temperature, the product was isolated by: method 1 – filtration, washing with MeOH and Et_2O and drying in vacuo; method 2 – by concentration of the reaction mixture to dryness in vacuo.

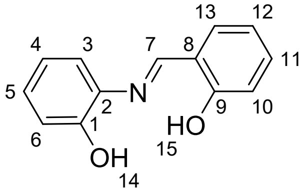

$C_{13}H_{11}NO_2$, 213.24 g/mol.

2-[(E)-(2-Hydroxybenzylidene)amino]phenol, H_2L1^H. From reaction between 2-aminophenol (270 mg, 2.45 mmol) and salicylic aldehyde (300 mg, 2.45 mmol) in EtOH (10 mL), the product was isolated by method 1, and obtained as an orange-red crystalline solid. Yield = 76 % (399 mg, 1.87 mmol). Analytical data are consistent with those reported in the literature.[26,27]

Mp.: 185-187°C.

^{1}H NMR (600 MHz, DMSO-d_6, 25 °C) δ = 13.79 (s, 1H, H15), 9.74 (s, 1H, H14), 8.96 (s, 1H, H7), 7.61 (dd, $^3J_{H,H}$ = 7.5 Hz, $^4J_{H,H}$ = 1.0 Hz, 1H, H13), 7.38 (ddd, $^3J_{H,H}$ = 8.0 Hz, $^3J_{H,H}$ = 8.0 Hz, $^4J_{H,H}$ = 1.5 Hz, 1H, H11), 7.36 (dd, $^3J_{H,H}$ = 8.0 Hz, $^4J_{H,H}$ = 1.0 Hz, 1H, H3), 7.13 (ddd, $^3J_{H,H}$ = 7.5 Hz, $^3J_{H,H}$ = 7.5 Hz, $^4J_{H,H}$ = 1.0 Hz, 1H, H5), 6.97 (dd, $^3J_{H,H}$ = 8.0 Hz, $^4J_{H,H}$ = 1.0 Hz, 1H, H6), 6.94 (m, 2H, H10/12), 6.88 (ddd, $^3J_{H,H}$ = 7.5 Hz, $^3J_{H,H}$ = 7.5 Hz, $^4J_{H,H}$ = 1.0 Hz, 1H, H4) ppm.

^{13}C{^{1}H} NMR (151 MHz, DMSO-d$_6$, 25 °C) δ = 161.7 C7, 160.7 C9, 151.1 C1, 135.0 C2, 132.8 C11, 132.3 C13, 128.1 C5, 119.6 C3+4, 119.5 C8, 118.7 C10/12, 116.7 C10/12, 116.5 C6 ppm.

IR (ATR), ν (cm^{-1}): 2557-3048 (m), 1627 (s).

HRMS (ESI): m/z calcd for $[C_{13}H_{12}NO_2]^+$: 214.0863, measured: 214.0861.

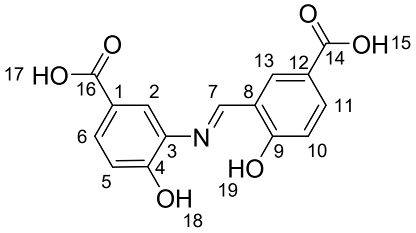

$C_{15}H_{11}NO_6$, 301.25 g/mol

4-Hydroxy-3-[(E)-(5-carboxy-2-hydroxybenzylidene)amino]benzoic acid, **$H_2L2^{C(O)OH}$**. From reaction between 3-amino-4-hydroxybenzoic acid (1087 mg, 6.54 mmol) and 3-formyl-4-hydroxybenzoic acid (1000 mg, 6.53 mmol) in EtOH (30 mL), the product was isolated by method 1 and obtained as a yellow solid. Yield = 70% (1375 mg, 4.56 mmol).

Mp.: decomposition > 300°C.

^{1}H NMR (400 MHz, DMSO, 25 °C): δ = 14.52 (br s, 1H, H19), 12.58 (br s, 2H, H15/17), 10.74 (br s, 1H, H18), 9.20 (s, 1H, H7), 8.35 (d, $^4J_{H,H}$ = 2.0 Hz, 1H, H13), 8.02 (d, $^4J_{H,H}$ = 2.0 Hz, 1H, H2), 7.94 (dd, $^3J_{H,H}$ = 8.7 Hz, $^4J_{H,H}$ = 2.1 Hz, 1H, H11), 7.77 (dd, $^3J_{H,H}$ = 8.5 Hz, $^4J_{H,H}$ = 2.0 Hz, 1H, H6), 7.05 (d, $^3J_{H,H}$ = 8.5 Hz, 1H, H5), 7.00 (d, $^3J_{H,H}$ = 8.7 Hz, 1H, H10) ppm.

^{13}C{^{1}H} NMR (101 MHz, DMSO, 25 °C): δ = 167.0, 166.8, 165.5, 162.1, 155.4, 134.9, 134.2, 133.9, 130.0, 122.2, 121.1, 120.8, 118.9, 117.5, 116.3 ppm.

IR (ATR), ν (cm^{-1}): 2789-3068 (m), 1725(s), 1657 (s), 1630 (s).

HRMS (ESI): m/z calcd for $[C_{15}H_{11}NO_6]^-$: 300.0514, found: 300.0512

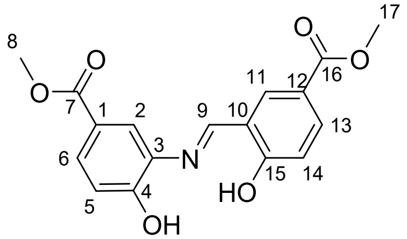

$C_{17}H_{15}NO_6$, 329.09 g/mol

4-Hydroxy-3-[(E)-(4-methyl-oxycarbonyl-2-hydroxybenzylidene)amino]benzoic acid methyl ester, $H_2L3^{C(O)OMe}$. From reaction between 3-amino-4-hydroxybenzoic acid methyl ester (139 mg, 0.83 mmol) and 3-formyl-4-hydroxybenzoic acid methyl ester (150 mg, 0.83 mmol) in MeOH (5 mL), the product was isolated by method 1, and obtained as a yellow solid. Yield = 70% (190 mg, 0.58 mmol).

Mp.: 201-204°C.

^{1}H NMR (400 MHz, DMSO-d$_6$, 25 °C) δ = 14.53 (s, 1 H, OH), 10.90 (s, 1 H, OH), 9.23 (s, 1H, H9) 8.38 (d, $^4J_{H,H}$ = 2.0 Hz, 1H, H11), 8.02 (d, $^4J_{H,H}$ = 2.0 Hz, 1H, H2), 7.95 (dd, $^3J_{H,H}$ = 8.5 Hz, $^4J_{H,H}$ = 1.5 Hz, 1H, H13), 7.79 (dd, $^3J_{H,H}$ = 8.5 Hz, $^4J_{H,H}$ = 2.0 Hz, 1H, H6), 7.07 (d, $^3J_{H,H}$ = 8.5 Hz, 1H, H5), 7.01 (d, $^3J_{H,H}$ = 8.5 Hz, 1H, H14), 3.84 (s, 3H, H8/17), 3.83 (s, 3H, H8/17) ppm.

^{13}C{^{1}H} NMR (151 MHz, DMSO-d$_6$, 25 °C) δ = 165.8 C7, 165.7 C15, 165.6 C16, 162.1 C9, 155.6 C4, 134.7 C11, 133.9 C10/13, 133.9 C10/13, 129.8 C6, 120.9 C1, 120.6 C2, 119.9 C12, 118.8 C10, 117.6 C14, 116.4 C5, 51.9 C8/C17 ppm.

IR (ATR), ν (cm^{-1}): 2789-3068 (m), 1716 (s), 1677 (s), 1618 (s).

HRMS (ESI): *m*/*z* calcd for $[C_{17}H_{15}NO_6]^+$: 330.0972, found 330.0970.

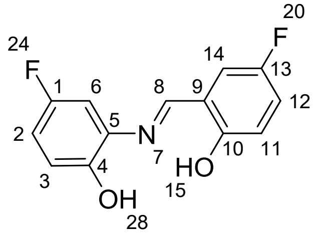

$C_{13}H_9F_2NO_2$, 249.21 g/mmol

4-Fluoro-2-{(*E*)-[(5-fluoro-2-hydroxyphenyl)imino]methyl}phenol, H_2L4^F. From reaction between 5-fluoro-2-hydroxybenzaldehyde (210 mg, 1.50 mmol) and 2-amino-4-fluorophenol (191 mg, 1.50 mmol) in MeOH (6 mL), the product was isolated by method 1, and the product obtained as an orange solid. Yield = 61% (228 mg, 0.91 mmol).

Mp.: 189–194 °C.

^{1}H NMR (400 MHz, DMSO-d$_6$, 25 °C): δ = 13.09 (s, 1H, H15/H28), 9.73 (s, 1H, H15/H28), 8.95 (s, 1H, H8), 7.50 (dd, $^3J_{F,H}$ = 9.0 Hz, $^4J_{H,H}$ = 3.2 Hz, 1H, H6/H14), 7.27 (m, 2H, H2/H6/H12/H14), 7.04–6.89 (m, 3H, H2/H3/H11/H12) ppm.

^{13}C{^{1}H} NMR (101 MHz, DMSO-d$_6$, 25 °C): δ = 161.3 (d, $^4J_{C,F}$ = 2.9 Hz, C8), 156.8 (d, $^4J_{C,F}$ = 1.9 Hz, C4/C10), 155.7 (d, $^1J_{C,F}$ = 236.2 Hz, C1/C13), 154.9 (d, $^1J_{C,F}$ = 234.2 Hz, C1/C13), 147.8 (d, $^4J_{C,F}$ = 1.9 Hz, C4/C10), 135.4 (d, $^3J_{C,F}$ = 7.6 Hz, C5), 120.2 (d, $^2J_{C,F}$ = 23.1 Hz, C2/C6/C12/C14), 119.7 (d, $^3J_{C,F}$ = 7.7 Hz, C9), 118.1 (d, $^3J_{C,F}$ = 7.7 Hz, C3/C11), 117.2 (d, $^3J_{C,F}$ = 8.6 Hz, C3/C11), 116.9 (d, $^2J_{C,F}$ = 23.1 Hz, C6/C14), 114.4 (d, $^2J_{C,F}$ = 23.1 Hz, C2/C12), 106.1 (d, $^2J_{C,F}$ = 24.1 Hz, C2/C6/C12/C14) ppm.

^{19}F NMR (376 MHz, DMSO-d$_6$): δ = -124.04 – -124.10 (m, 1F, F20/F24), -125.25 – -125.31 (m, 1F, F20/F24) ppm.

IR (ATR), ν (cm^{-1}): 2881 (m), 1634 (s), 1119 (s).

HRMS (ESI): *m*/*z* calcd for $[C_{13}H_{10}F_2NO_2]^+$: 250.0674, found: 250.0674.

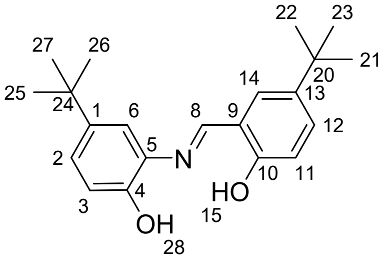

$C_{21}H_{27}NO_2$, 325.45 g/mol

4-Tert-butyl-2-{(E)-[(5-tert-butyl-2-hydroxyphenyl)imino]methyl}phenol, H_2L5^{tBu}. From reaction between 5-(tert-butyl)-2-hydroxybenzaldehyde (257 µL, 1.50 mmol) and 2-amino-4-(tert-butyl)phenol (248 mg, 1.50 mmol) in MeOH (6 mL), the product was isolated by method 2, and obtained as a red solid. Yield = 91% (442 mg, 1.36 mmol).

Mp.: 190–195°C.

^{1}H NMR (400 MHz, DMSO-d$_6$, 25 °C): δ = 13.69 (s, 1H, H15/H28), 9.48 (s, 1H, H15/H28), 9.01 (s, 1H, H8), 7.64 (d, $^4J_{H,H}$ = 3.0 Hz, 1H, H6/H14), 7.42 (dd, $^3J_{H,H}$ = 8.5 Hz, $^4J_{H,H}$ = 2.5 Hz, 1H, H2/H12), 7.35 (d, $^3J_{H,H}$ = 2.4 Hz, 1H, H6/H14), 7.13 (dd, $^3J_{H,H}$ = 8.5 Hz, $^4J_{H,H}$ = 2.5 Hz, 1H, H2/H12), 6.88 (d, $^3J_{H,H}$ = 4.5 Hz, 1H, H3/H11), 6.86 (d, $^3J_{H,H}$ = 5.0 Hz, 1H, H3/H11), 1.30 – 1.29 (2×s, 18H, H21-H27) ppm.

^{13}C{^{1}H} NMR (101 MHz, DMSO-d$_6$, 25 °C): δ = 161.8 (C8), 158.6 (C4/C10), 148.7 (C4/C10), 142.0 (C1/C13), 140.8 (C1/C13), 134.2 (C5), 130.0 (C2/C12), 128.6 (C6/C14), 124.6 (C2/C12), 118.8 (C9), 116.2 (C3/C11), 115.9 (C6/C14), 34.0 (C20/C24), 33.8 (C20/C24), 31.4 (C21-C27), 31.3 (C21-C27) ppm.

IR (ATR), ν (cm^{-1}): 2964 (m), 2902 (m), 2865 (m), 1622 (s).

HRMS (ESI) m/z calcd for $C_{21}H_{28}NO_2^+$: 326.2115, found 326.2121.

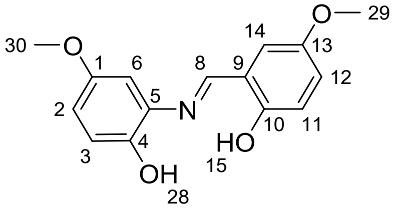

$C_{15}H_{15}NO_4$, 273.29 g/mol

2-{(E)-[(2-hydroxy-5-methoxyphenyl)imino]methyl}-4-methoxyphenol, H_2L6^{OMe}. From reaction between 2-hydroxy-5-methoxybenzaldehyde (187 µL, 1.50 mmol) and 2-amino-4-methoxyphenol (209 mg, 1.50 mmol) in MeOH (6 mL), the product was isolated by method 2, and the product obtained as a dark red solid. Yield = 98% (404 mg, 1.48 mmol).

Mp.: 180–185 °C.

^{1}H NMR (400 MHz, DMSO-d$_6$, 25 °C): δ = 13.11 (s, 1H, H15/H28), 9.24 (s, 1H, H15/H28), 8.95 (s, 1H, H8), 7.21 (d, $^4J_{H,H}$ = 3.4 Hz, 1H, H6/H14), 7.01 (dd, $^3J_{H,H}$ = 8.8 Hz, $^4J_{H,H}$ = 2.9 Hz, 1H, H2/H12), 6.97 (d, $^4J_{H,H}$ = 2.9 Hz, 1H, H6/H14), 6.87 (d, $^3J_{H,H}$ = 9.8 Hz, 2H, H3/H11), 6.73 (dd, $^3J_{H,H}$ = 8.8 Hz, $^4J_{H,H}$ = 2.9 Hz, 1H, H2/H12), 3.75 (s, 3H, H29/H30), 3.73 (s, 3H, H29/H30), ppm.

$^{13}C\{^1H\}$ NMR (101 MHz, DMSO-d$_6$, 25 °C): δ = 161.3 (C8), 154.8 (C1/C13), 152.7 (C1/C13), 151.6 (C4/C10), 145.2 (C4/C10), 135.2 (C5), 120.2 (C2/C12), 119.3 (C9), 117.5 (C3/C11), 117.1 (C3/C11), 115.0 (C6/C14), 113.9 (C2/C12), 104.4 (C6/C14), 55.5 (C29/C30) ppm.

IR (ATR), ν (cm^{-1}): 2935 (m), 2832 (s), 1608 (s), 1147 (m).

HRMS (ESI): *m/z* calcd for $[C_{15}H_{16}NO_4]^+$: 274.1074, found: 274.1076.

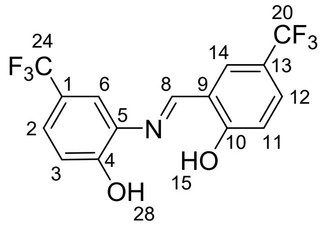

$C_{15}H_9F_6NO_2$, 349,2324 g/mol

2-[(*E*)-{[2-hydroxy-5-(trifluoromethyl)phenyl]imino}methyl]-4-(trifluoromethyl)-phenol , H_2L7^{CF3}. From reaction between 2-amino-4-(trifluoromethyl)phenol (140 mg, 0.8 mmol) and 2-hydroxy-5-(trifluoromethyl)benzaldehyde (150 mg, 0.8 mmol) in MeOH (6 mL), the product was isolated by method 2, and the product obtained as a dark red solid. Yield = 97 % (267 mg, 0.78 mmol).

Mp.: 216–221 °C.

^1H NMR (400 MHz, DMSO-d$_6$, 25 °C): δ = 14.07 (s, 1H, H4/H10), 10.75 (s, 1H, H4/H10), 9.19 (s, 1H, H8), 8.10 (d, $^4J_{H,H}$ = 3.0 Hz, 1H, H6/14), 7.77 (d, $^4J_{H,H}$ = 3.0 Hz, 1H, H6/H14), 7.73 (dd, $^3J_{H,H}$ = 9.0 Hz, $^4J_{H,H}$ = 3.0 Hz, 1H, H2/12), 7.51 (dd, $^3J_{H,H}$ = 8.5 Hz, $^4J_{H,H}$ = 2.5 Hz, 1H, H2/12), 7.15 – 7.12 (m, 2H, H3/H11) ppm.

$^{13}C\{^1H\}$ NMR (101 MHz, DMSO-d$_6$, 25 °C): δ = 163.8, 162.4, 154.5, 134.8, 129.7 (m), 125.8 (m) 125.3 (m), 123.1 (m), 120.4, 120.1, 119.6, 119.3, 119.2, 118.0 , 116.9 (m) (signals could not be unambiguously assigned due to low intensity of some peaks, and multiple $J_{C,F}$ couplings).

^{19}F NMR (376 MHz, DMSO-d$_6$, 25 °C): δ = -59.54 (s, 3F, F20/F24), -59.91 (s, 3F, F20/F24) ppm.

IR (ATR), ν (cm^{-1}): 2982 (m), 1624(s), 1121 (s).

HRMS (ESI): *m/z* calcd for $[C_{15}H_{10}F_6NO_2]^+$: 350.0610, found: 350.0610.

General methods for complex synthesis

Method A. In a typical reaction, 100 mg of ligand and an equimolar amount of $MoO_2(acac)_2$ were suspended in MeOH (2 mL, dried over 3 Å molecular sieves) and stirred for 4 h. The precipitate was isolated by: method 1 – filtration, washed with a small quantity MeOH and Et_2O (approximately 1 mL) and dried *in vacuo*; method 2 – by concentration of the reaction mixture to dryness in vacuo.

Method B. In a typical reaction, 100 mg of ligand was dissolved in hot (90°C) MeOH (10 mL), to which an equimolar amount of $Na_2MoO_4•2H_2O$ and 2 molar equivalents of glacial acetic acid (89.0 µL, 1.51 mmol) were added. The resultant mixture was refluxed at 90 °C for 5 h. After cooling down, the product was isolated by filtration, washed with small amount of MeOH (approximately 2 mL), and dried in vacuo.

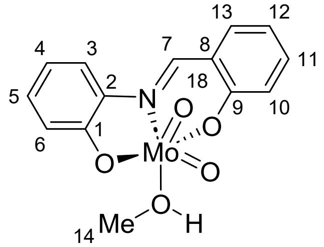

$C_{14}H_{13}MoNO_5$, 371.22 g/mol

Complex 1^{H}. **1^{H}** was prepared by method A; from the reaction between **H_2L1^{H}** (69 mg, 0.32 mmol) and $MoO_2(acac)_2$ (105 mg, 0.32 mmol) in MeOH (8 mL), the product was isolated by method 1, and obtained as a yellow solid. Yield = 81% (97 mg, 0.26 mmol).

Mp.: loss of MeOH > 120 °C, decomposition > 300 °C.

^{1}H NMR (400 MHz, DMSO-d$_6$, 25 °C) δ = 9.28 (1H, H7), 7.82 (dd, $^{3}J_{H,H}$ = 8.0 Hz, $^{4}J_{H,H}$ = 1.0 Hz, 1H, H3), 7.77 (dd, $^{3}J_{H,H}$ = 8.0 Hz, $^{4}J_{H,H}$ = 1.5 Hz, 1H, H13), 7.54 (ddd, $^{3}J_{H,H}$ = 7.5 Hz, $^{3}J_{H,H}$ = 7.5 Hz, $^{4}J_{H,H}$ = 1.5 Hz, 1H, H11), 7.23 (ddd, $^{3}J_{H,H}$ = 7.5 Hz, $^{3}J_{H,H}$ = 7.5 Hz, $^{4}J_{H,H}$ = 1.0 Hz, 1H, H5), 7.06 (ddd, $^{3}J_{H,H}$ = 7.5 Hz, $^{3}J_{H,H}$ = 7.5 Hz, $^{4}J_{H,H}$ = 1.0 Hz, 1H, H12), 6.99 – 6.90 (m, 2H, H10/H4), 6.85 (dd, $^{3}J_{H,H}$ = 8.0 Hz, $^{4}J_{H,H}$ = 1.0 Hz, 1H, H6), 4.12 (q, $^{3}J_{H,H}$ = 5.5 Hz, 1H, MeO*H*), 3.17 (d, $^{3}J_{H,H}$ = 5.5 Hz, 3H, H14).

^{13}C{^{1}H} NMR (101 MHz, DMSO, 25 °C) δ = 161.2 C9, 160.1 C1, 156.7 C7, 135.6 C2/C11, 135.2 C13, 130.1 C5, 122.0 C8, 120.9 C12, 120.4 C10, 118.9 C4/C10, 117.4 C4/C10, 116.5 C3, 48.7 C14 ppm.

IR (ATR), ν (cm^{-1}): 1611 (s), 908 (br), 931 (s).

UV/Vis (*DMSO*): λ_{max} (ε) = 422 (4500), 352 nm (7800 mol^{-1} dm^{3} cm^{-1})

LIFDI-MS: m/z calcd for $[C_{13}H_9MoNO_4]^+$: 340.9586, found: 340.9585.

Elemental analysis calcd (%) for $C_{13}H_9MoNO_4$: C 45.3, H 3.5, N 3.8, found: C 45.05, H 3.3, N 3.6.

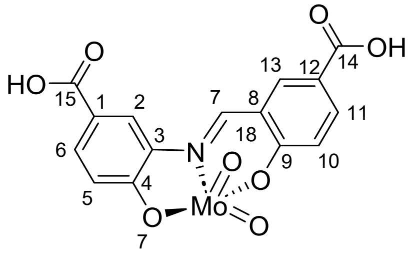

$C_{15}H_9MoNO_8$, 427.20 g/mol

Complex 2$^{C(O)OH}$. **2$^{C(O)OH}$** was prepared by method A; from reaction between **H_2L2$^{C(O)OH}$** (196 mg, 0.65 mmol) and $MoO_2(acac)_2$ (210 mg, 0.64 mmol) in MeOH (6 mL), the product was isolated by method 1, and obtained as a brown solid. Yield = 78% (231 mg, 0.54 mmol).

Mp.: decomposition > 300 °C.

^{1}H NMR (400 MHz, DMSO, 25 °C): δ = 13.00 (v br, 2H , COO*H*), 9.62 (1H, H7), 8.59 (d, $^{4}J_{H,H}$ = 2.0 Hz, 1H, H13), 8.49 (d, $^{4}J_{H,H}$ = 1.5 Hz, 1H, H2), 8.06 (dd, $^{3}J_{H,H}$ = 8.5 Hz, $^{4}J_{H,H}$ = 2.0 Hz, 1H, H11), 7.84 (dd, $^{3}J_{H,H}$ = 8.5 Hz, $^{4}J_{H,H}$ = 1.5 Hz, 1H, H6), 7.02 (d, $^{3}J_{H,H}$ = 8.5 Hz, 1H, H10), 6.94 (d, $^{3}J_{H,H}$ = 8.5 Hz, 1H, H5) ppm.

$^{13}C\{^1H\}$ NMR (101 MHz, DMSO, 25 °C) δ = 166.9 C15, 166.4 C14, 164.1 C9, 163.8 C4, 158.4 C7, 138.0 C13, 136.4 C11, 136.7 C3, 131.8 C6, 123.4 C12, 123.1 C1, 121.8 C8, 119.5 C10, 118.6 C2, 117.4 C5 ppm.

IR (ATR), ν (cm^{-1}): 1722 (s br), 1668 (s), 1607 (s), 946 (s), 808 (s).

UV/Vis (*DMSO*): λ_{max} (ε) = 406 (4200), 352 nm (2500 $mol^{-1}\,dm^3\,cm^{-1}$)

LIFDI-MS: m/z calcd for $[C_{13}H_9MoNO_4]^+$: 428.9395, found: 428.9382.

Elemental analysis calcd (%) for $C_{30}H_{18}Mo_2N_2O_{16}$ • 1.8 H_2O: C 40.6, H 2.5, N 3.2, found: C 40.6, H 2.4, N 3.1.

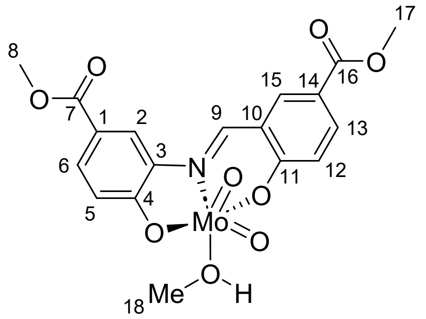

$C_{18}H_{17}MoNO_9$, 487.29

Complex $3^{C(O)OMe}$. $3^{C(O)OMe}$ was prepared by method A; from reaction between **$H_2L3^{C(O)OMe}$** (100 mg, 0.30 mmol) and $MoO_2(acac)_2$ (100 mg, 0.30 mmol) in MeOH (1.5 mL), the product was isolated by method 1, and obtained as a yellow solid. Yield = 82% (120 mg, 0.25 mmol).

Mp.: loss of MeOH > 180 °C, decomposition > 300 °C.

^1H NMR (400 MHz, DMSO, 25 °C): δ = 9.66 (s, 1H, H9), 8.62 (s, 1H, H15), 8.51 (s, 1H, H2), 8.08 (dd, $^3J_{H,H}$ = 9.0 Hz, $^3J_{H,H}$ = 1.5 Hz, 1H, H13), 7.87 (d, $^3J_{H,H}$ = 9.0 Hz, 1H, H6), 7.05 (d, $^3J_{H,H}$ = 9.0 Hz, 1H, H12), 6.97 (d, $^3J_{H,H}$ = 8.5 Hz, 1H, H5), 4.13 (q, $^3J_{H,H}$ = 5.0 Hz, 1H, MeO*H*), 3.87 (s, 6H, H8/H17), 3.16 (d, $^3J_{H,H}$ = 5.0 Hz, 3H, H18) ppm.

$^{13}C\{^1H\}$ NMR (101 MHz, DMSO, 25 °C) δ = 165.8 C7, 165.2 C16, 164.3 C11, 164.0 C4, 158.5 C9, 137.9 C15, 136.1 C13, 135.8 C3, 131.6 C6, 122.2 C10, 121.9 C1, 121.9 C14, 119.7 12, 118.5 C2, 117.6 C5, 52.3 C8/17, 52.2 C8/17, 48.7 C18 ppm.

IR (ATR), ν (cm^{-1}): 1689 (s br), 1608 (s), 936 (s), 908 (s).

UV/Vis (*DMSO*): λ_{max} (ε) = 409 (4500), 347 nm (10000 $mol^{-1}\,dm^3\,cm^{-1}$)

Fluorescence (DMSO): λ_{ex}=410 nm; λ_{em}=525 nm

LIFDI-MS: m/z calcd for $[C_{18}H_{17}MoNO_9]^+$: 456.96898, found: 456.97027.

Elemental analysis calcd (%) for $C_{18}H_{17}MoNO_9$ • 0.30 H_2O: C 43.9, H 3.6, N 2.8, found: C 43.5, H 3.2, N 2.5.

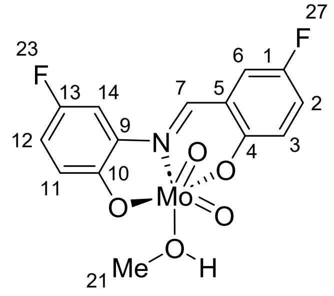

$C_{14}H_{11}F_2MoNO_5$, 407.19 g/mol

Complex 4^F. **4^F** was prepared by method B; from reaction between **H_2L4^F** (74.5 mg, 0.30 mmol), Na_2MoO_4 (72.3 mg, 0.30 mmol) and glacial acetic acid (35.0 µL, 0.60 µmol) in MeOH (5 mL), the product was obtained as an orange solid. Yield = 68% (82 mg, 0.20 mmol).

M.p.: >300 °C.

^{1}H NMR (400 MHz, DMSO-d$_6$, 25 °C): δ = 9.23 (s, 1H, H7), 7.68 (dd, $^3J_{F,H}$ = 10.0 Hz, $^4J_{H,H}$ = 3.0 Hz, 1H, H6/H14), 7.53 (dd, $^3J_{F,H}$ = 9.0 Hz, $^4J_{H,H}$ = 3.5 Hz, 1H, H6/H14), 7.44 (ddd, $^3J_{H,H}$ = 8.5 Hz, $^3J_{F,H}$ = 8.5 Hz, $^4J_{H,H}$ = 3.5 Hz, 1H, H2/H12), 7.13 ($^3J_{H,H}$ = 9.0 Hz, $^3J_{H,H}$ = 9.0 Hz, $^4J_{H,H}$ = 3.0 Hz, 1H, H2/H12), 6.98 (dd, $^3J_{H,H}$ = 9.0 Hz, $^4J_{F,H}$ = 4.5 Hz, 1H, H3/H11), 6.88 (dd, $^3J_{H,H}$ = 9.0 Hz, $^4J_{F,H}$ = 5.0 Hz, 1H, H3/H11) ppm.

^{13}C{^{1}H} NMR (101 MHz, DMSO-d$_6$, 25 °C): δ = 158.0 (d, $^4J_{C,F}$ = 2.0 Hz, C4/C10), 157.3 (d, $^4J_{C,F}$ = 3.0 Hz, C7), 157.2 (d, $^1J_{C,F}$ = 84.0 Hz, C1/C13), 156.7 (d, $^4J_{C,F}$ = 2.0 Hz, C4/C10), 154.8 (d, $^1J_{C,F}$ = 83.0 Hz, C1/C13), 135.5 (d, $^3J_{C,F}$ = 10.0 Hz, C9), 123.0 (d, $^2J_{C,F}$ = 23.5 Hz, C2/C12), 121.9 (d, $^3J_{C,F}$ = 8.0 Hz, C5), 120.6 (d, $^3J_{C,F}$ = 8.0 Hz, C3/C11), 119.3 (d, $^2J_{C,F}$ = 24.0 Hz, C6/C14), 117.9 (d, $^3J_{C,F}$ = 8.5 Hz, C3/C11), 117.0 (d, $^2J_{C,F}$ = 24.0 Hz, C2/C12), 103.6 (d, $^2J_{C,F}$ = 26.0 Hz, C6/C14) ppm.

^{19}F NMR (376 MHz, DMSO-d$_6$, 25 °C): δ = -122.38 – -122.45 (m, 1F, F23/F27), -123.22 – -123.28 (m, 1F, F23/F27) ppm.

IR (ATR), ν (cm^{-1}): 3239 (s), 1617 (s), 1136 (s), 929 (s), 904 (s).

UV/vis (DMSO): λ_{max} (ε) = 438 (4500), 360 (7700), 306 nm (15200 mol^{-1} dm^3 cm^{-1}).

LIFDI-MS: m/z calcd for $[C_{13}H_7NO_4F_2Mo]^+$: 376.9392, found: 376.9381.

Elemental analysis calcd (%) for $C_{14}H_{11}F_2MoNO_5$: C 41.3, H 2.7, N 3.4, found: C 41.9, H 3.3, N 3.6.

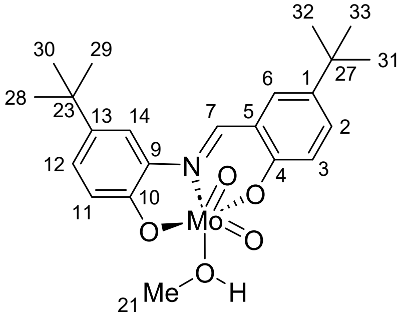

$C_{22}H_{29}MoNO_5$, 483.43 g/mol

Complex 5^{tBu}. 5^{tBu} was prepared using method A; from reaction between **H_2L5^{tBu}** (200 mg, 0.62 mmol) and $MoO_2(acac)_2$ (201 mg, 0.62 mmol) in MeOH (10 mL), the product was isolated by method 2, recrystallised by vapor diffusion from THF and heptane, and obtained as orange needles. Yield = 22% (64 mg, 0.13 mmol).

M.p.: >300 °C.

^{1}H NMR (400 MHz, DMSO-d$_6$, 25 °C): δ = 9.33 (s, 1H, H7), 7.82 (d, $^4J_{H,H}$ = 2.5 Hz, 1H, H6/H14), 7.81 (d, $^4J_{H,H}$ = 2.0 Hz, 1H, H6/H14), 7.58 (dd, $^3J_{H,H}$ = 8.5 Hz, $^4J_{H,H}$ = 2.5 Hz, 1H, H2/H12), 7.25 (dd, $^3J_{H,H}$ = 8.5 Hz, $^4J_{H,H}$ = 2.0 Hz, 1H, H2/H12), 6.85 (d, $^3J_{H,H}$ = 8.5 Hz, 1H, H3/H11), 6.75 (d, $^3J_{H,H}$ = 8.5 Hz, 1H, H3/H11), 1.33 (2×s, 18H, H28-H33) ppm.

^{13}C{^{1}H} NMR (101 MHz, DMSO-d$_6$, 25 °C): δ = 159.1 (C4/C10), 158.0 (C4/C10), 156.5 (C7), 143.2 (C1/C13), 143.0 (C1/C13), 135.0 (C9), 132.8 (C2/C12), 131.3 (C6/C14), 126.9 (C2/C12), 121.4 (C5), 118.4 (C3/C11), 116.4 (C3/C11), 112.9 (C6/C14), 34.4 (C23/C27), 34.0 (C23/C27), 31.4 (C28/C29/C30/C31/C32/C33), 31.1 (C28/C29/C30/C31/C32/C33) ppm.

IR (ATR), ν (cm^{-1}): 2956 (s), 1612 (s), 932 (s), 911 (s).

UV/vis (DMSO): λ_{max} (ε) = 434 (3900), 359 (7800), 315 nm (17800 mol^{-1} dm^3 cm^{-1}).

LIFDI-MS: m/z calcd for $[C_{21}H_{25}NO_4Mo]^+$: 453.0832, found: 453.0819.

Elemental analysis calcd (%) for $C_{21}H_{25}NO_4Mo$: C 54.7, H 6.1, N 2.9, found: C 56.0, H 5.8, N 2.5.

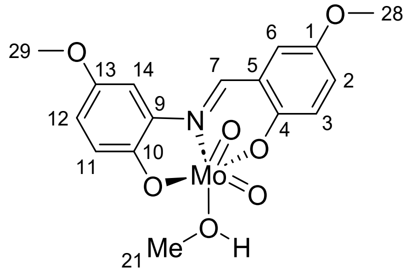

$C_{16}H_{17}MoNO_7$, 431.26 g/mol

Complex 6^{OMe}. 6^{OMe} was prepared by method B; from reaction between **H_2L6^{OMe}** (206 mg, 0.76 mmol), Na_2MoO_4 (183 mg, 0.76 mmol) and glacial acetic acid (35.0 µL, 1.51 mmol) in MeOH (10 ml), the product was obtained as a red solid. Yield = 78% (255 mg, 0.20 mmol).

M.p.: >300 °C.

^{1}H NMR (400 MHz, DMSO-d$_6$, 25 °C): δ = 9.23 (s, 1H, H7), 7.39 (d, $^4J_{H,H}$ = 3.0 Hz, 1H, H6/H14), 7.31 (d, $^4J_{H,H}$ = 3.5 Hz, 1H, H6/H14), 7.14 (dd, $^3J_{H,H}$ = 9.0 Hz, $^4J_{H,H}$ = 3.0 Hz, 1H, H2/H12), 6.88–6.82 (m, 2H, H2/H3/H11/H12), 6.76 (d, $^3J_{H,H}$ = 9.0 Hz, 1H, H3/H11), 3.79 (s, 6H, H28/H29) ppm.

^{13}C{^{1}H} NMR (101 MHz, DMSO-d$_6$, 25 °C): δ = 156.4 (C7), 155.9 (C1/C13), 154.6 (C1/C13), 153.6 (C4/C10), 152.9 (C4/C10), 135.4 (C9), 122.8 (C2/C12), 121.8 (C5), 119.7 (C3/C11), 117.5 (C3/C11), 117.0 (C2/C12), 116.7 (C6/C14), 100.7 (C6/C14), 55.8 (C28/C29), 55.6 (C28/C29) ppm.
IR (ATR), ν (cm^{-1}): 3384 (s), 1620 (s), 1150 (s), 926 (s), 904 (s).

UV/vis (DMSO): λ_{max} (ε) = 467 (4000), 377 (7900), 314 nm (1690 mol^{-1} dm^3 cm^{-1}).

LIFDI-MS: m/z calcd for $[C_{15}H_{13}NO_6Mo]^+$: 400.9792, found 400.9792.

Elemental analysis calcd (%) for $C_{16}H_{17}NO_7Mo$: C 44.6, H 4.0, N 3.3, found: C 41.7, H 3.4, N 2.3.

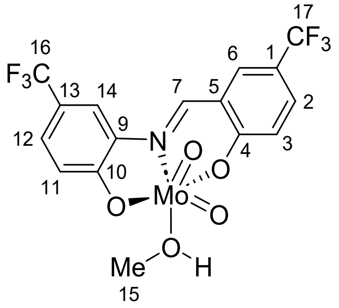

$C_{16}H_{11}F_6MoNO_5$, 507.21 g/mol

Complex 7^{CF3}. 7^{CF3} was prepared by method A, from reaction of **H_2L7^{CF3}** (100 mg, 0.3 mmol) with $[MoO_2(acac)_2]$ (93.2 mg, 0.3 mmol) in MeOH (5 mL). The product was isolated by method 2 and obtained as a dark red solid. Yield = 93 % (135 mg, 0.3 mmol).

M.p.: >300 °C.

^{1}H NMR (400 MHz, DMSO-d$_6$, 25°C): δ = 9.60 (s, 1H, H7), 8.23 (m, 2H, H6/14), 7.89 (dd, $^3J_{H,H}$ = 9.0 Hz, $^4J_{H,H}$ = 2.5 Hz, 1H, H2/H12), 7.61 (dd, $^3J_{H,H}$ = 9.0 Hz, $^4J_{H,H}$ = 2.5 Hz, 1H, H2/H12), 7.16 (d, $^4J_{H,H}$ = 8.5 Hz, 1H, H3/H11), 7.07 (d, $^4J_{H,H}$ = 8.5 Hz, 1H, H3/H11) ppm.

^{13}C{^{1}H} NMR (101 MHz, DMSO-d$_6$, 25°C): δ = 163.1, 162.8, 158.6, 135.6, 133.0 (m), 132.2 (m), 127.2 (m), 125.6, 123.0, 122.6, 121.6, 121.1 (m), 120.5, 118.3, 114.7 (m) ppm (could not be confidently assigned due to low intensity of some peaks, and multiple $J_{C,F}$ couplings).

^{19}F NMR (376 MHz, DMSO-d$_6$, 25°C): δ = -59.56 (s, 3F, F16/F17), -60.35 (s, 3F, F16/F17) ppm.
IR (ATR), ν (cm^{-1}): 1623 (s), 1152 (s), 923 (s), 898 (s).

UV/vis (DMSO): λ_{max} (ε) = 409 (4300), 342 (8300), 299 nm (15900 mol^{-1} dm^3 cm^{-1}) .

LIFDI-MS: m/z calcd for $[C_{15}H_7NO_4F_6Mo]^+$: 476.9328, found 476.9313.

Elemental analysis calcd (%) for $C_{16}H_{11}NO_5F_6Mo$: C 37.9, H 2.2, 2.8, found: C 39.3, H 1.7, N 2.8.

^{1}H and ^{13}C{^{1}H} NMR spectra of ligands and complexes

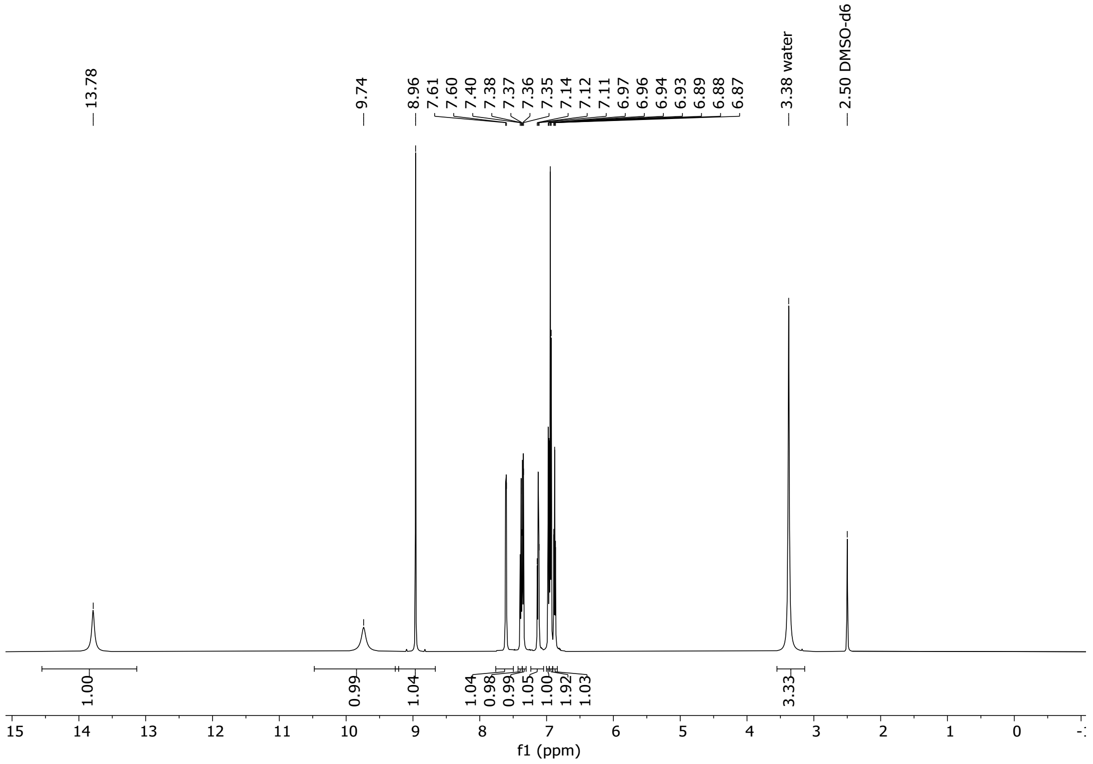

Figure S 3: **H_2L1^H** ^{1}H NMR spectrum (600 MHz, DMSO-d_6, 25 °C).

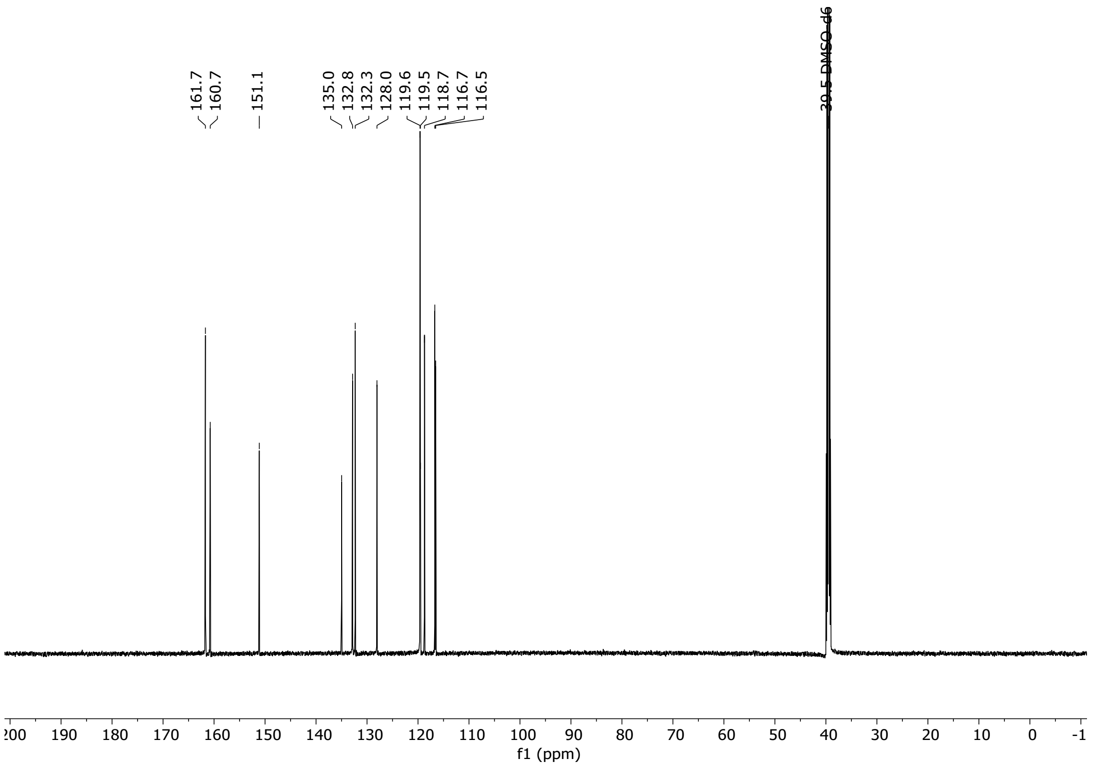

Figure S 4: **H_2L1^H** ^{13}C{^{1}H} NMR spectrum (151 MHz, DMSO-d_6, 25 °C).

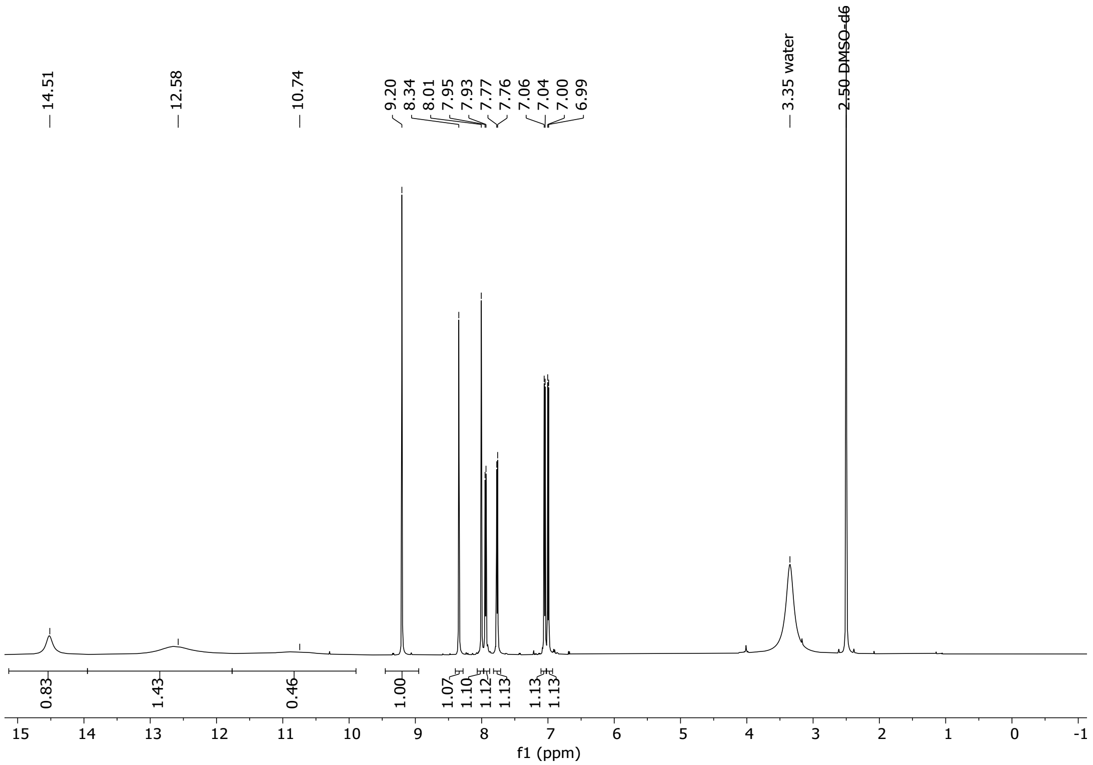

Figure S 5: **$H_2L2^{C(O)OH}$** ^{1}H NMRs spectrum (600 MHz, DMSO-d_6, 25 °C).

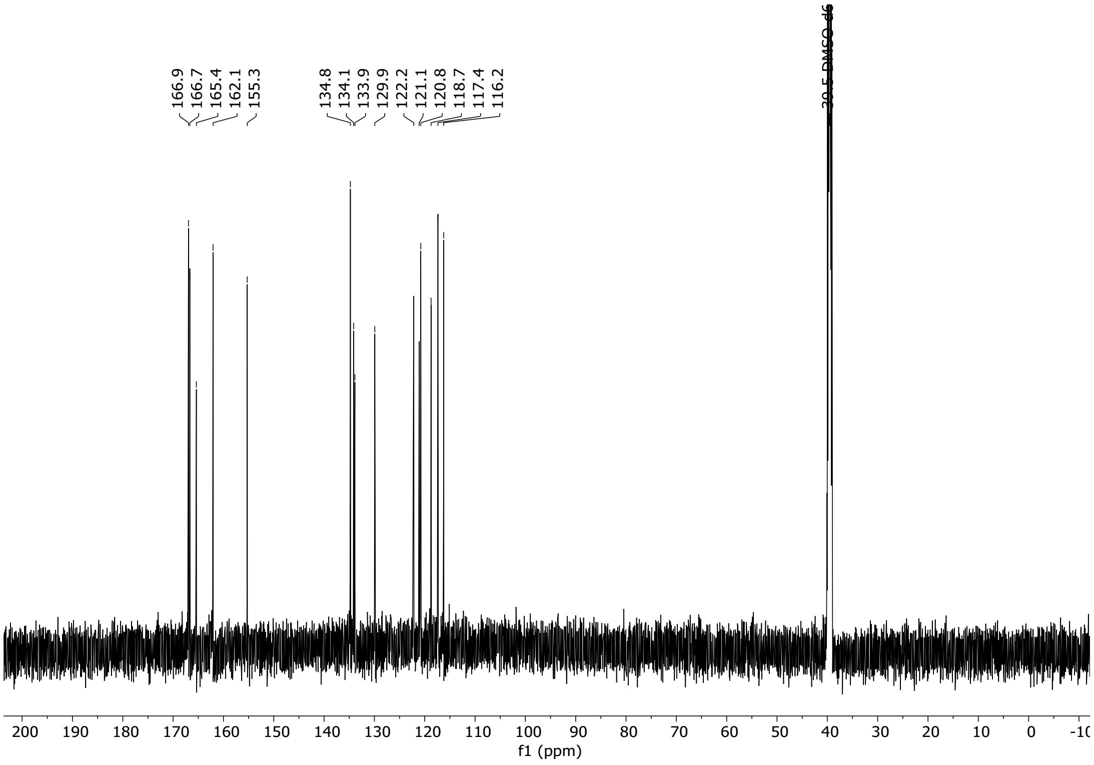

Figure S 6: **$H_2L2^{C(O)OH}$** ^{13}C {^{1}H} NMR spectrum (151 MHz, DMSO-d_6, 25 °C).

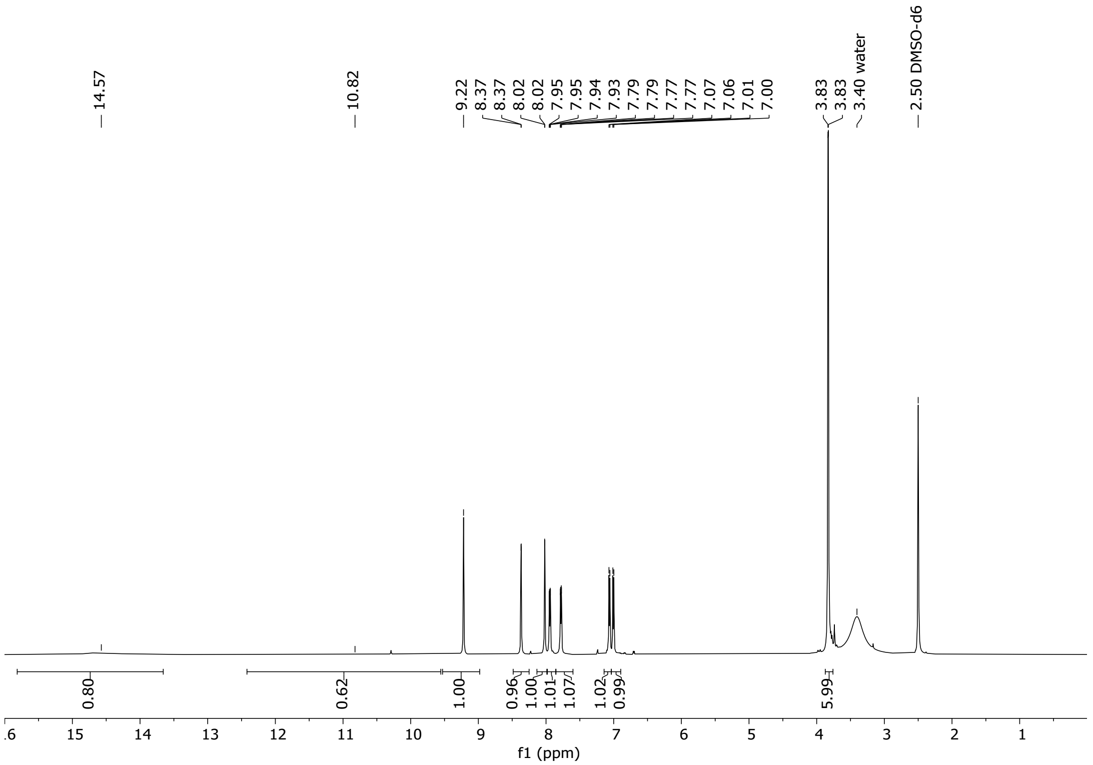

Figure S 7: **$H_2L3^{C(O)OMe}$** ^{1}H NMR spectrum (600 MHz, DMSO-d_6, 25 °C).

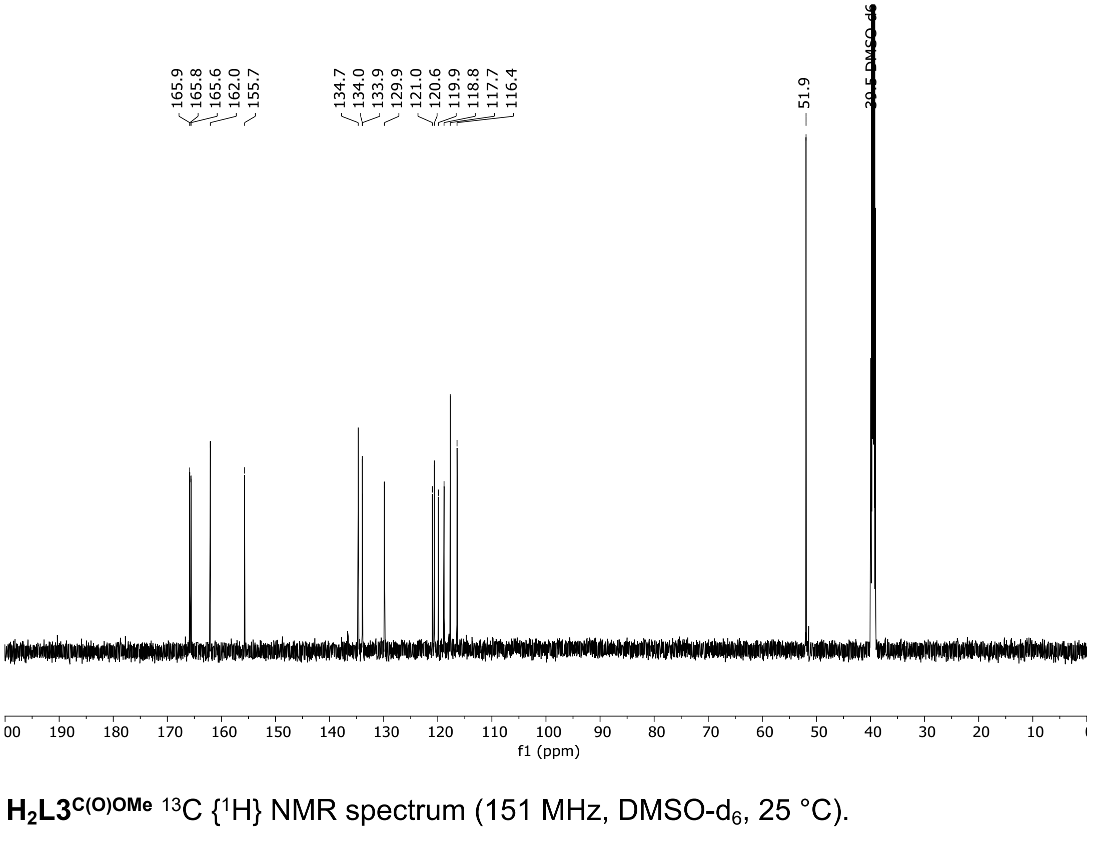

Figure S 8: **$H_2L3^{C(O)OMe}$** ^{13}C {^{1}H} NMR spectrum (151 MHz, DMSO-d_6, 25 °C).

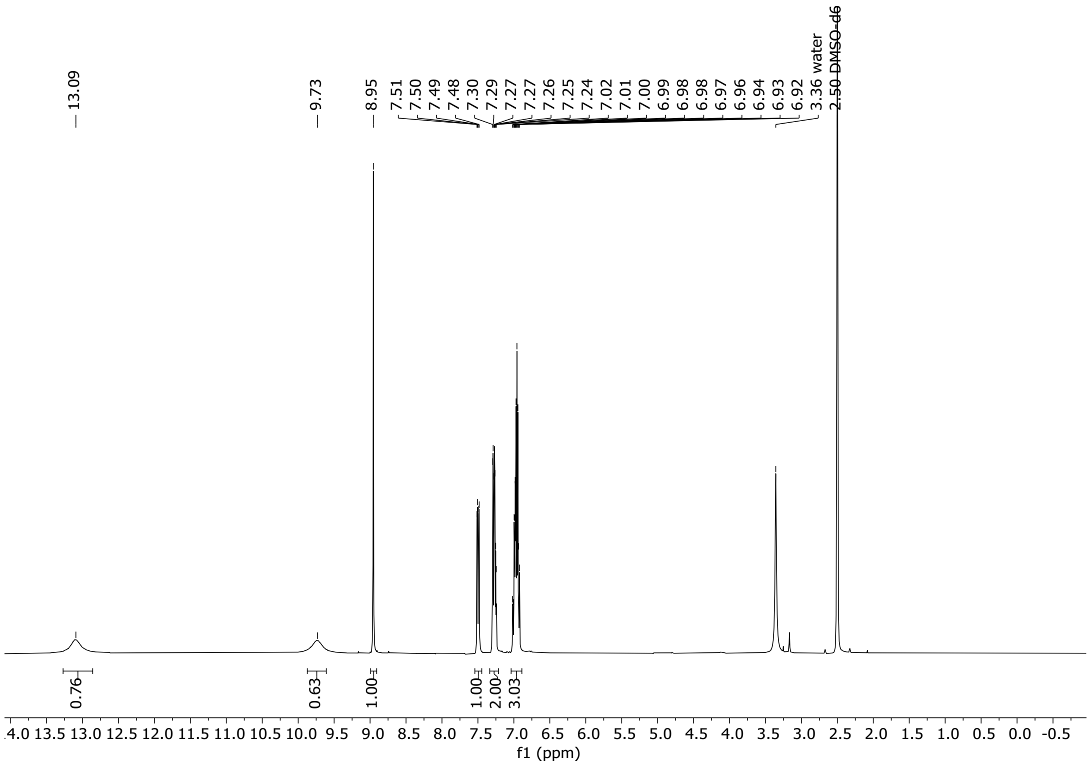

Figure S 9: **H_2L4^F** ^{1}H NMR spectrum (400 MHz, DMSO-d_6, 25 °C).

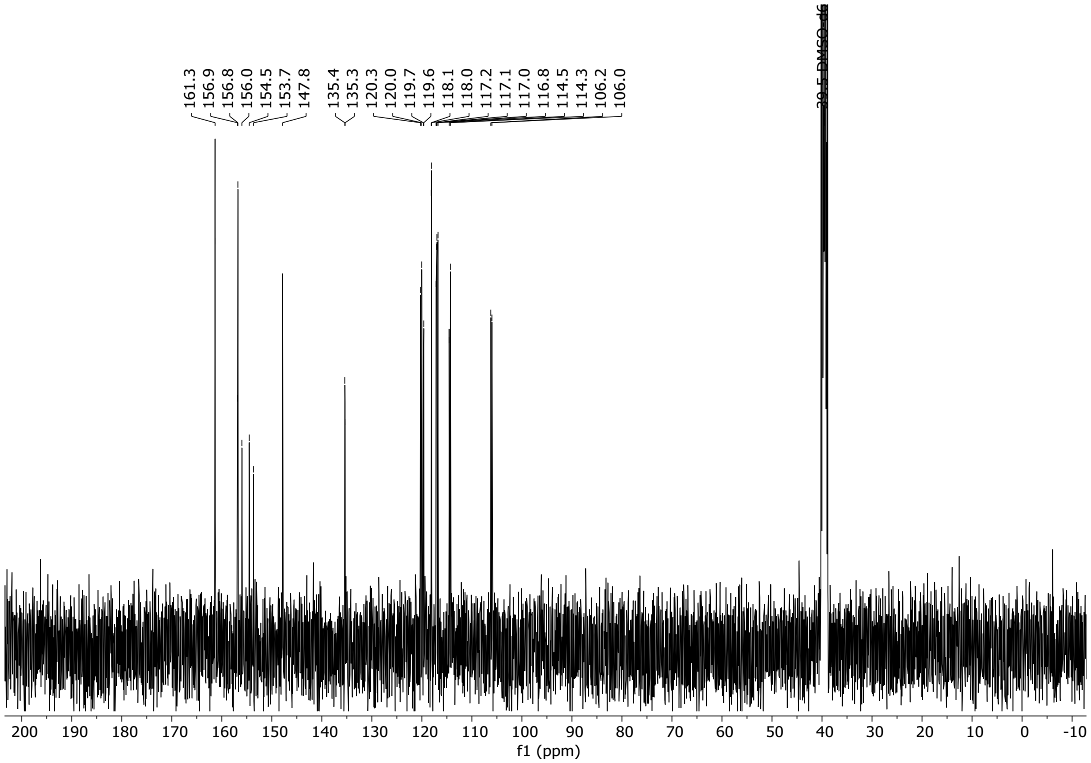

Figure S 10: **H_2L4^F** ^{13}C {^{1}H} NMR spectrum (101 MHz, DMSO-d_6, 25 °C).

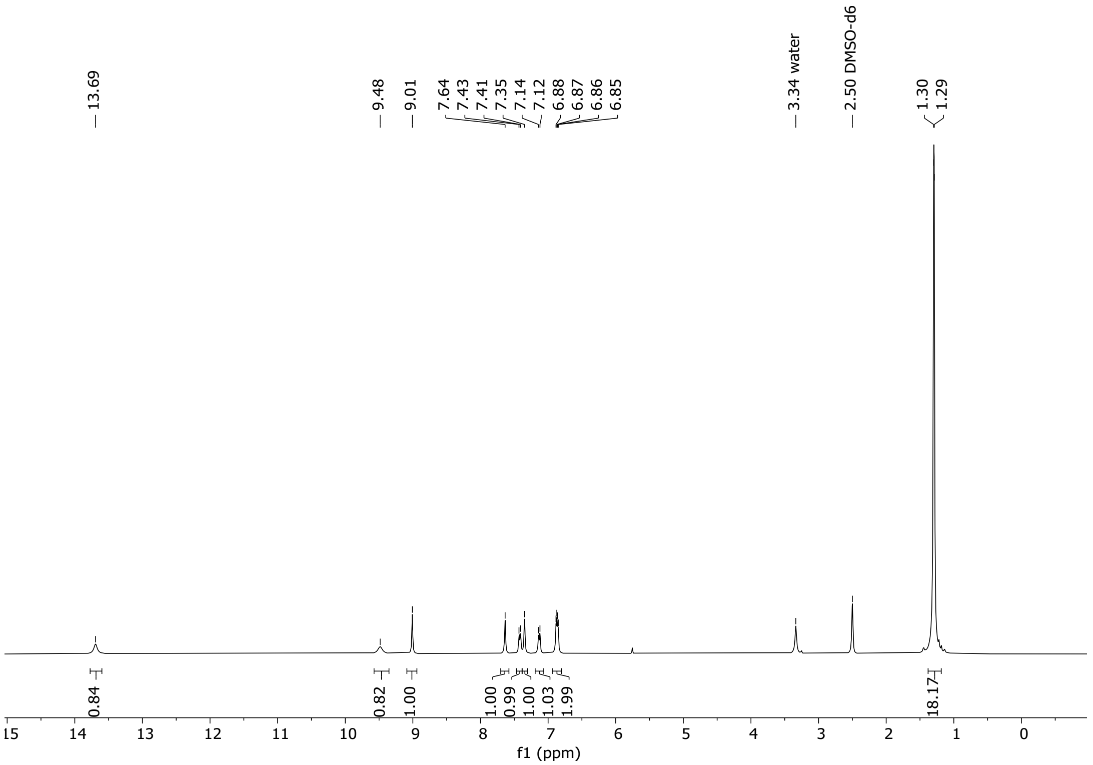

Figure S 11: **H_2L5^{tBu}** ^{1}H NMR spectrum (400 MHz, DMSO-d_6, 25 °C).

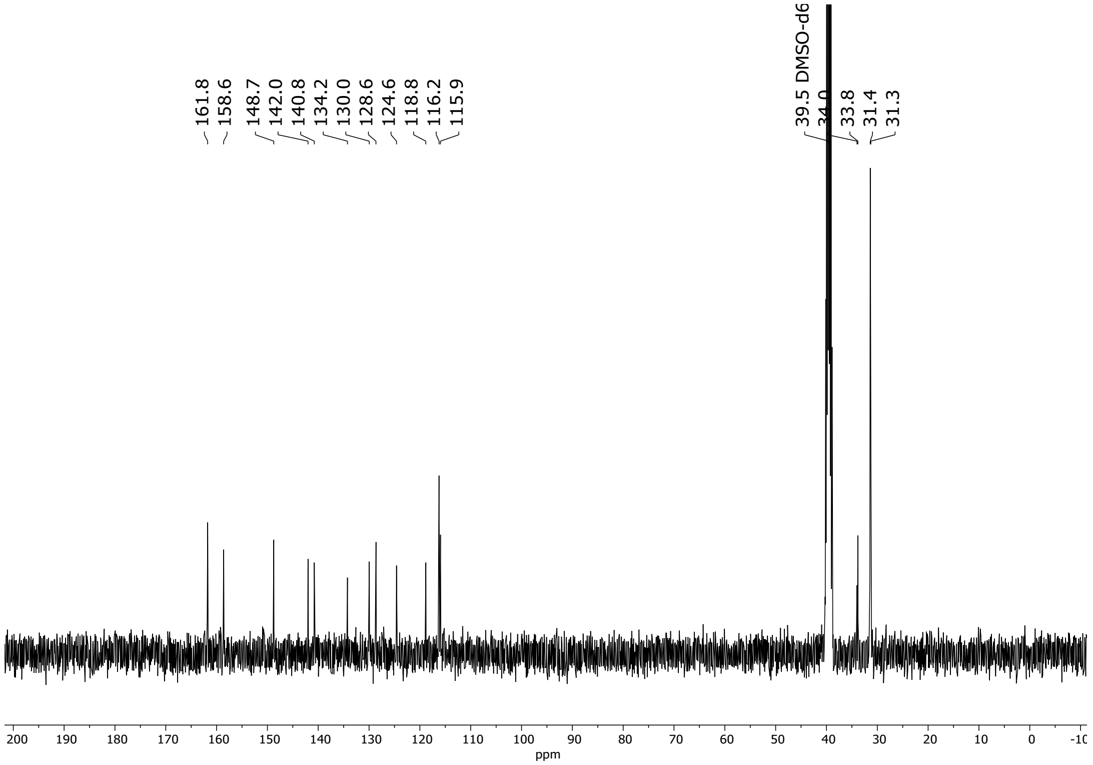

Figure S 12: **H_2L5^{tBu}** ^{13}C {^{1}H} NMR spectrum (101 MHz, DMSO-d_6, 25 °C).

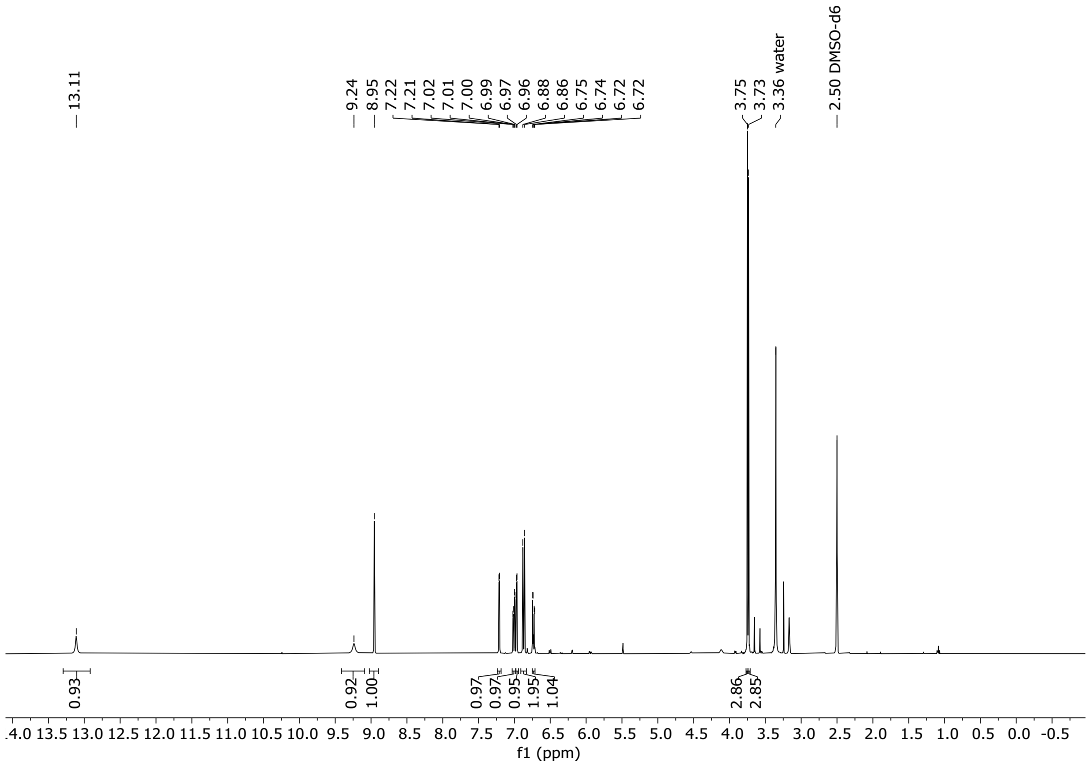

Figure S 13: **H_2L6^{OMe}** ^{1}H NMR spectrum (400 MHz, DMSO-d$_6$, 25 °C).

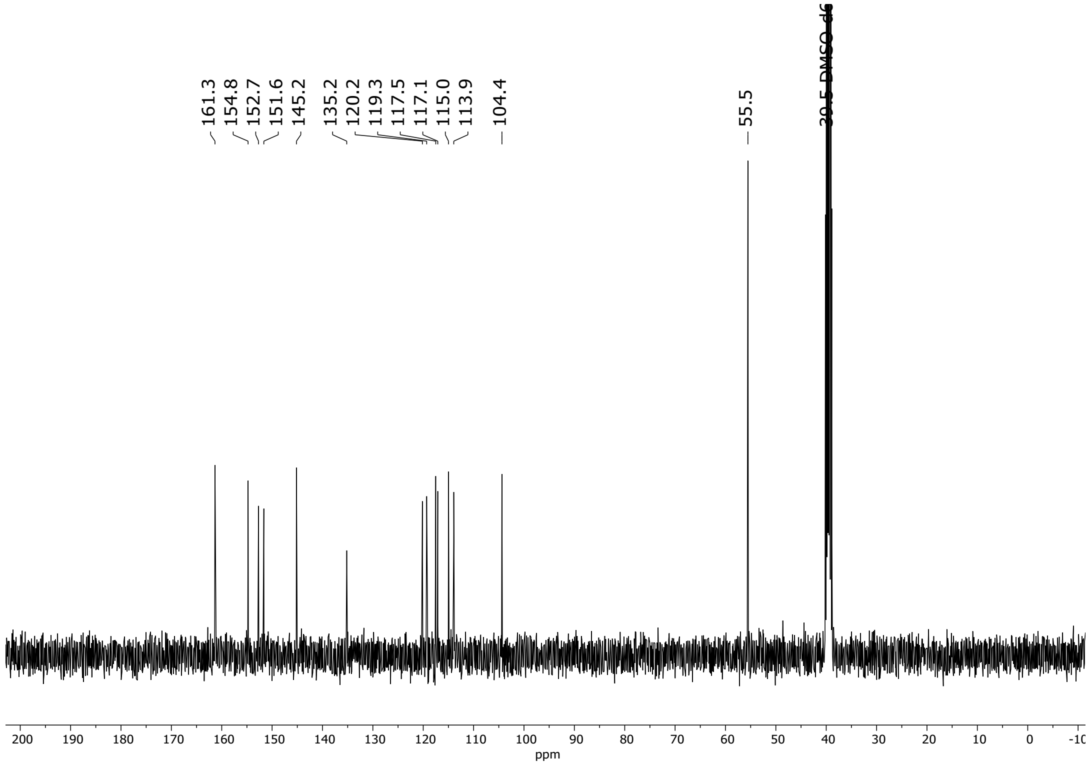

Figure S 14: **H_2L6^{OMe}** ^{13}C {^{1}H} NMR spectrum (101 MHz, DMSO-d$_6$, 25 °C).

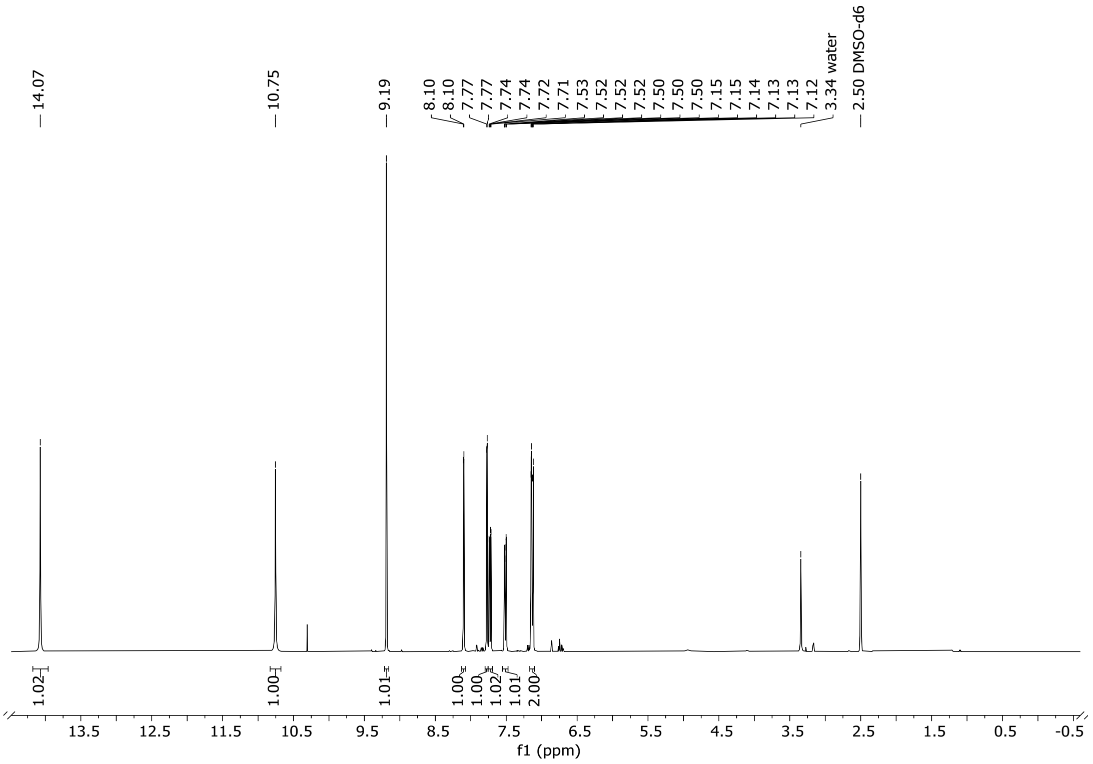

Figure S 15. **H_2L7^{CF3}** ^1H NMR spectrum (400 MHz, DMSO-d_6, 25 °C).

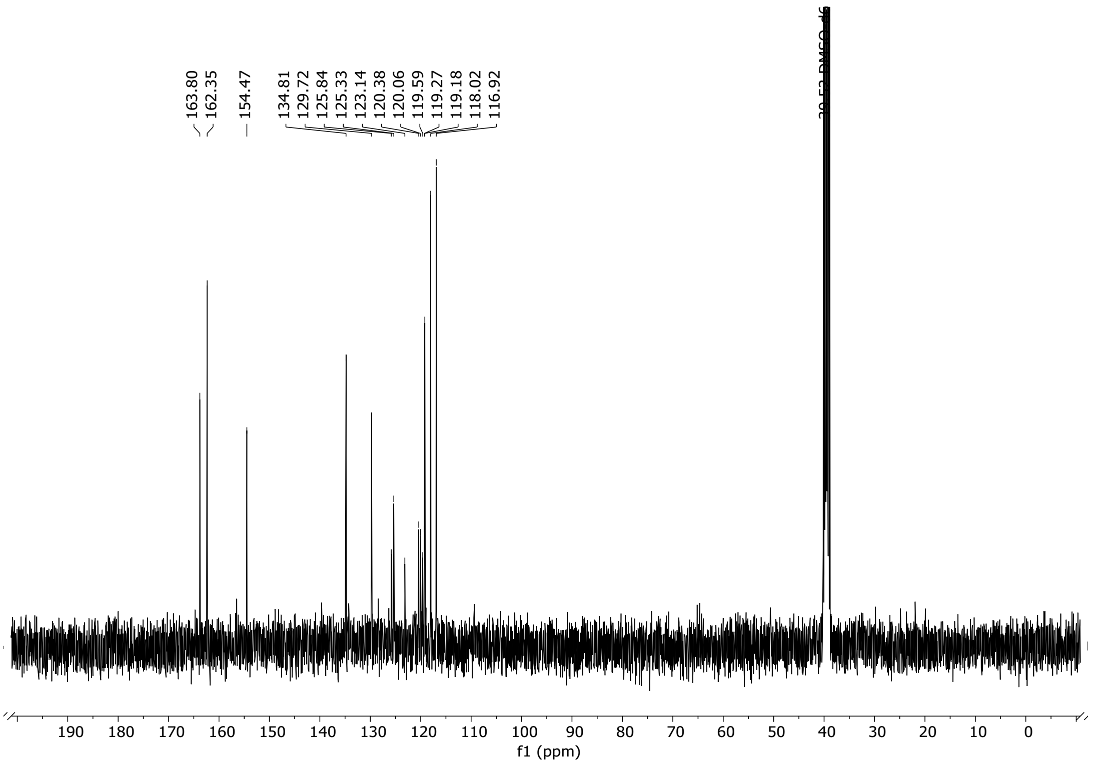

Figure S 16. **H_2L7^{CF3}** ^{13}C {^1H} NMR spectrum (101 MHz, DMSO-d_6, 25 °C).

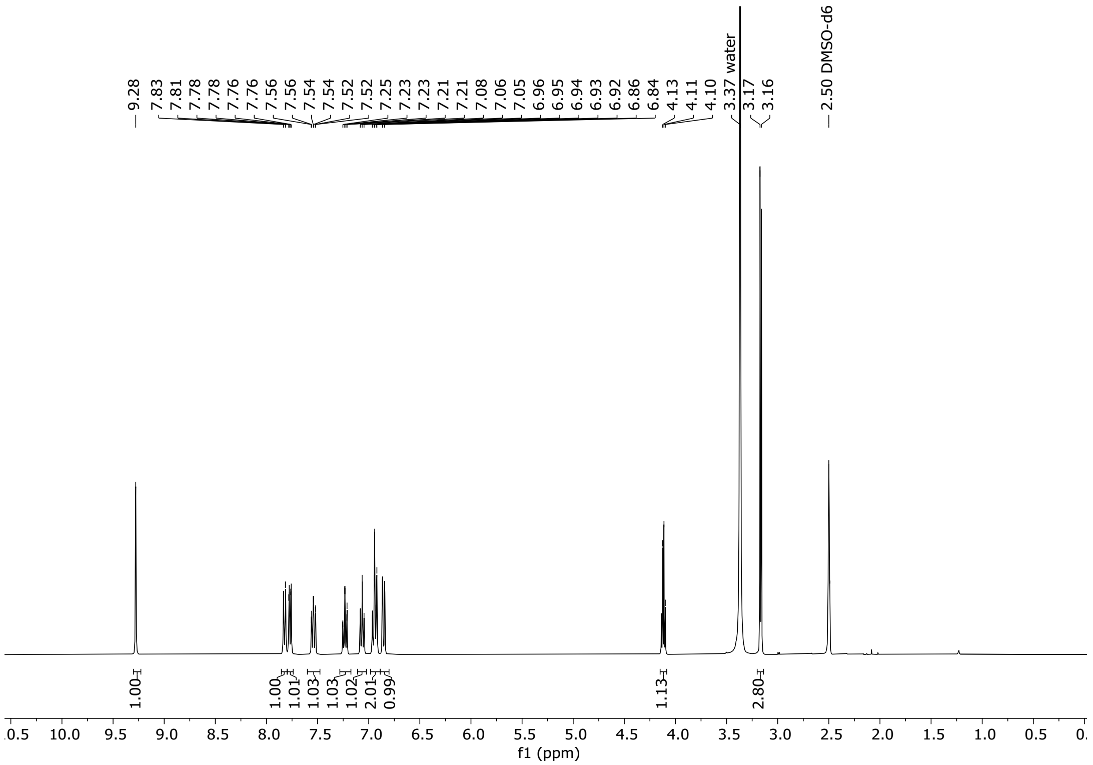

Figure S 17: Complex $\mathbf{1^H}$ ^{1}H NMR spectrum (400 MHz, DMSO-d_6, 25 °C).

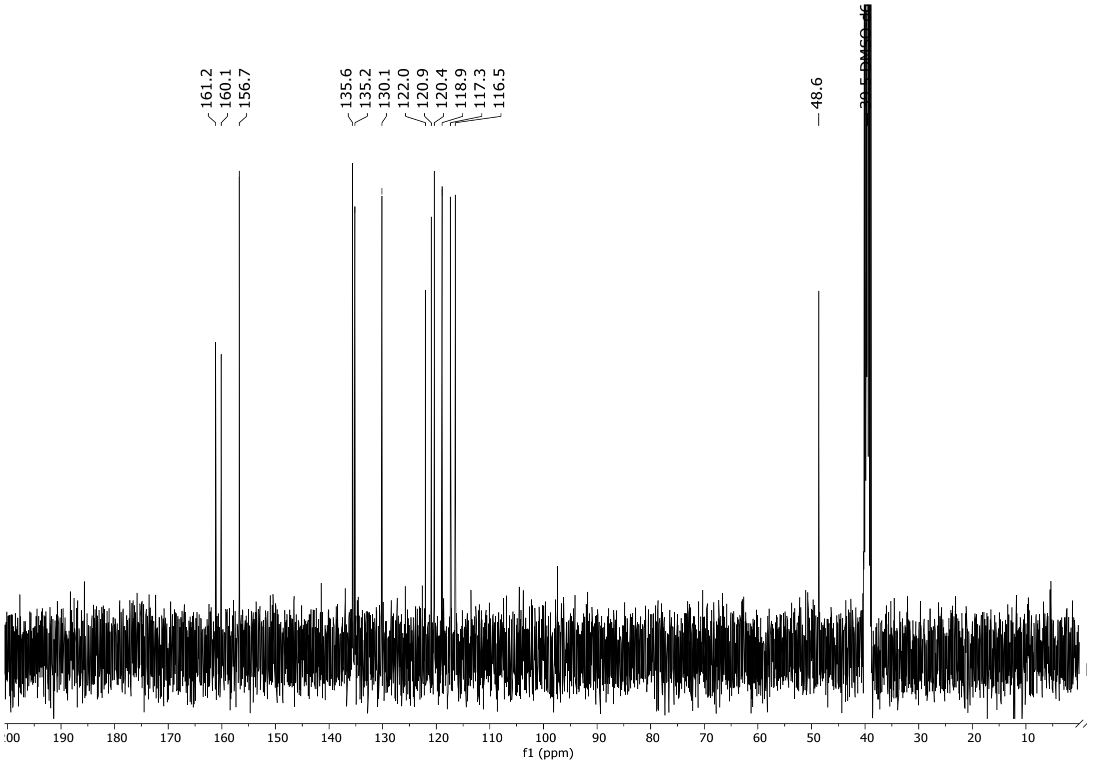

Figure S 18: Complex $\mathbf{1^H}$ ^{13}C {^{1}H} NMR spectrum (101 MHz, DMSO-d_6, 25 °C).

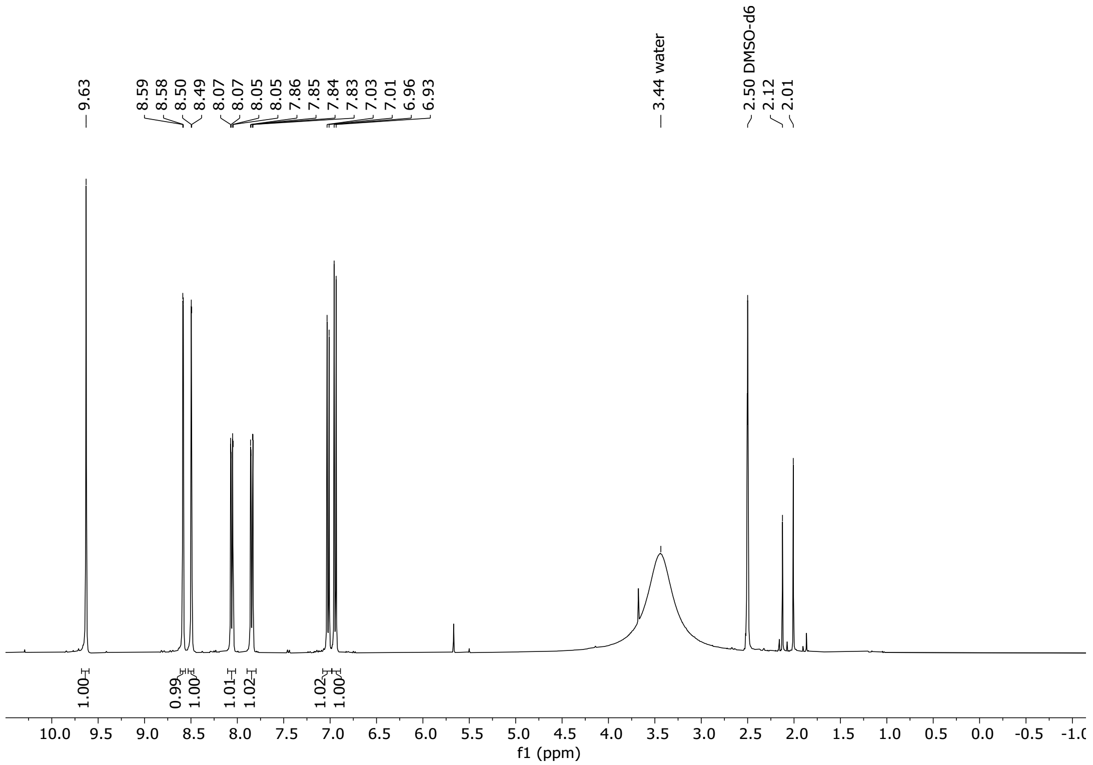

Figure S 19: Complex **2**$^{C(O)OH}$ ^{1}H NMR spectrum (400 MHz, DMSO-d_6, 25 °C).

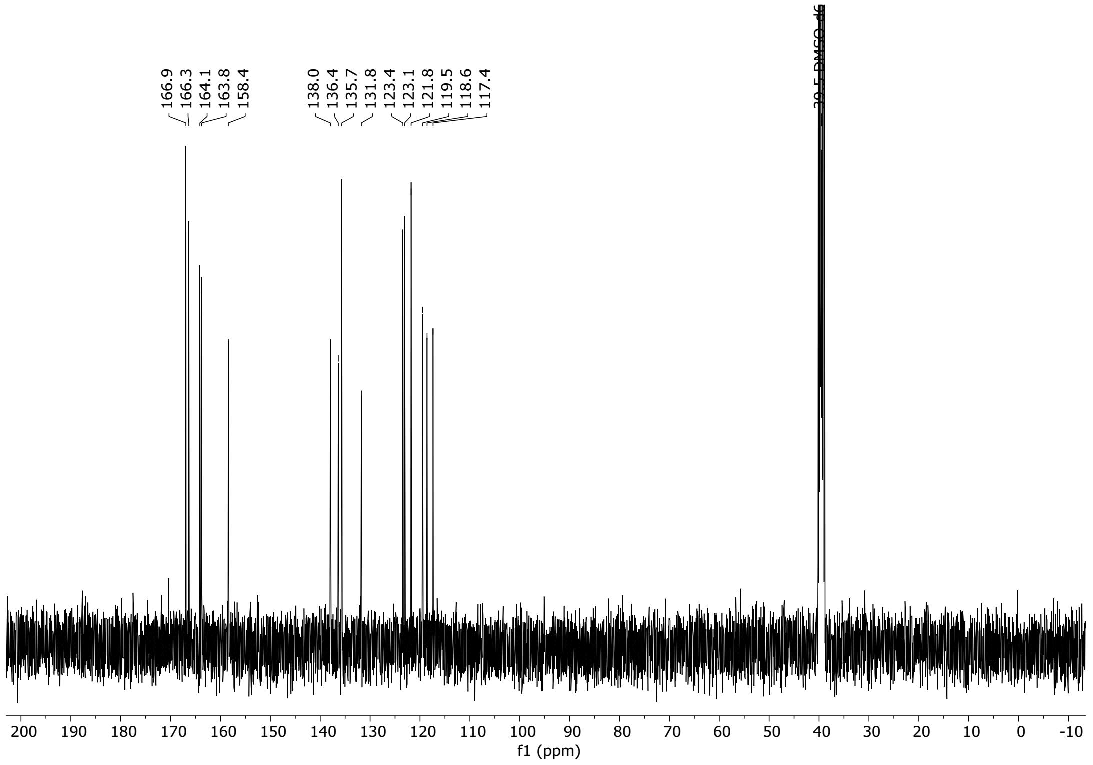

Figure S 20: Complex **2**$^{C(O)OH}$ ^{13}C {^{1}H} NMR spectrum (101 MHz, DMSO-d_6, 25 °C).

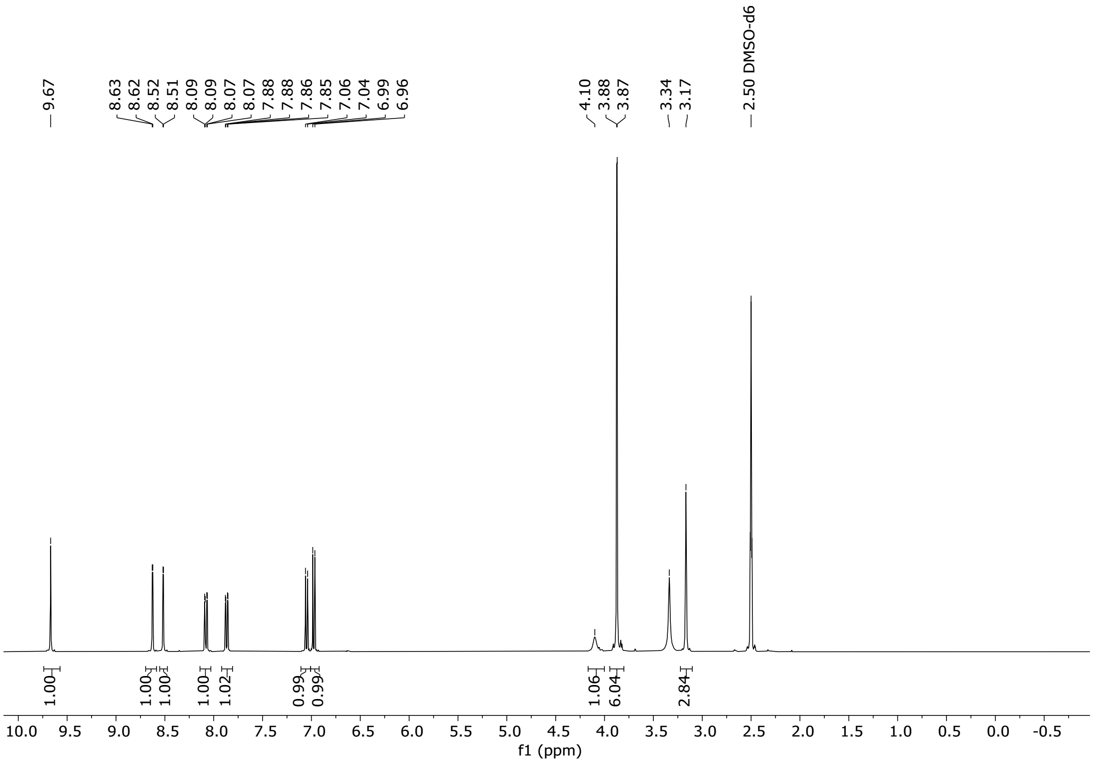

Figure S 21: Complex **3**$^{\mathbf{C(O)OMe}}$ ^{1}H NMR spectrum (400 MHz, DMSO-d_6, 25 °C).

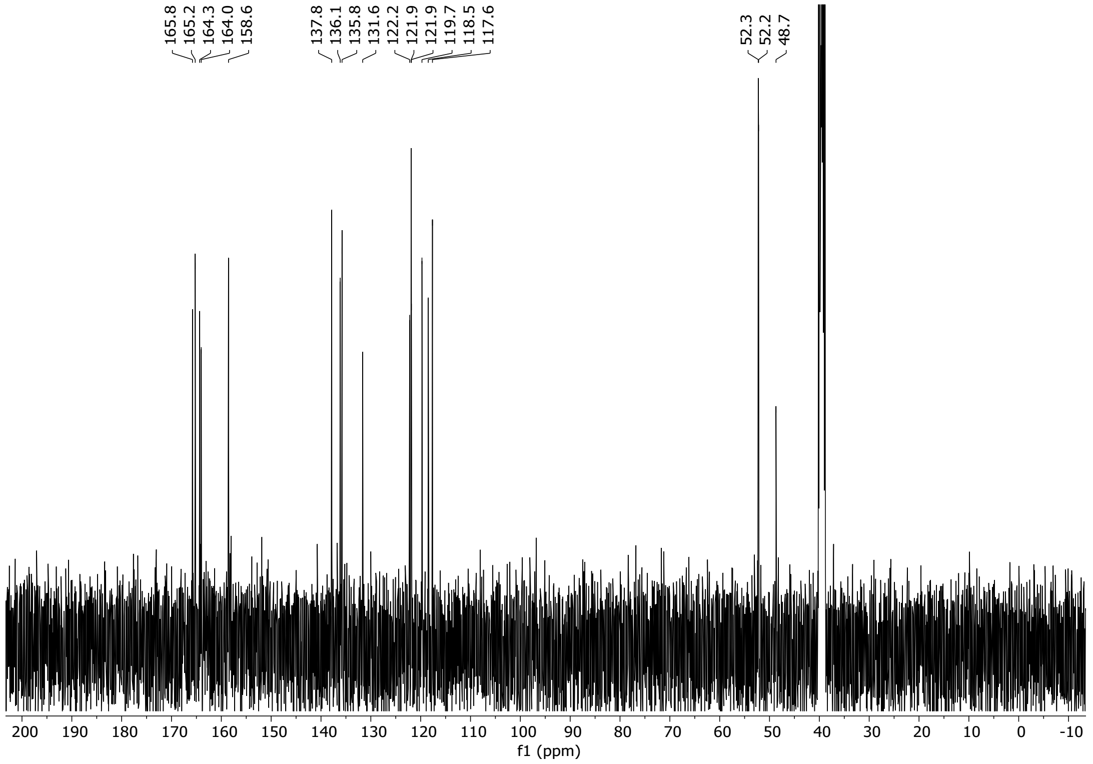

Figure S 22: Complex **3**$^{\mathbf{C(O)OMe}}$ ^{13}C {^{1}H} NMR spectrum (101 MHz, DMSO-d_6, 25 °C).

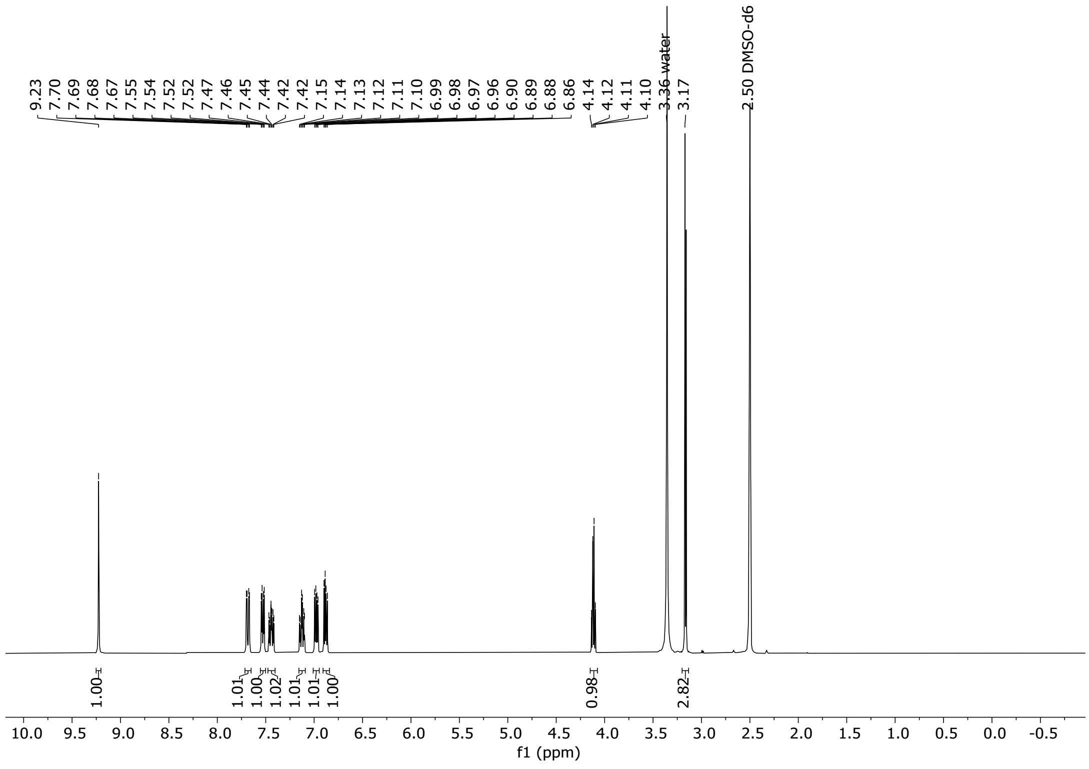

Figure S 23: Complex **4^F** ^{1}H NMR spectrum (400 MHz, DMSO-d_6, 25 °C).

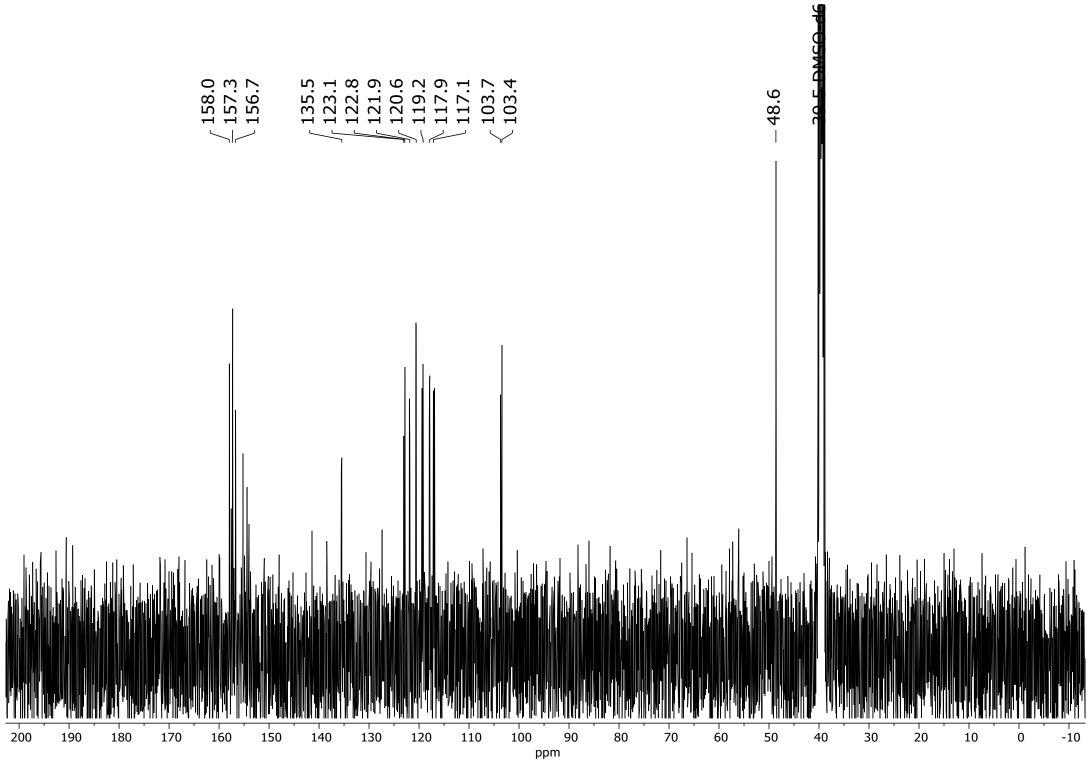

Figure S 24: Complex **4^F** ^{13}C {^{1}H} NMR spectrum (101 MHz, DMSO-d_6, 25 °C).

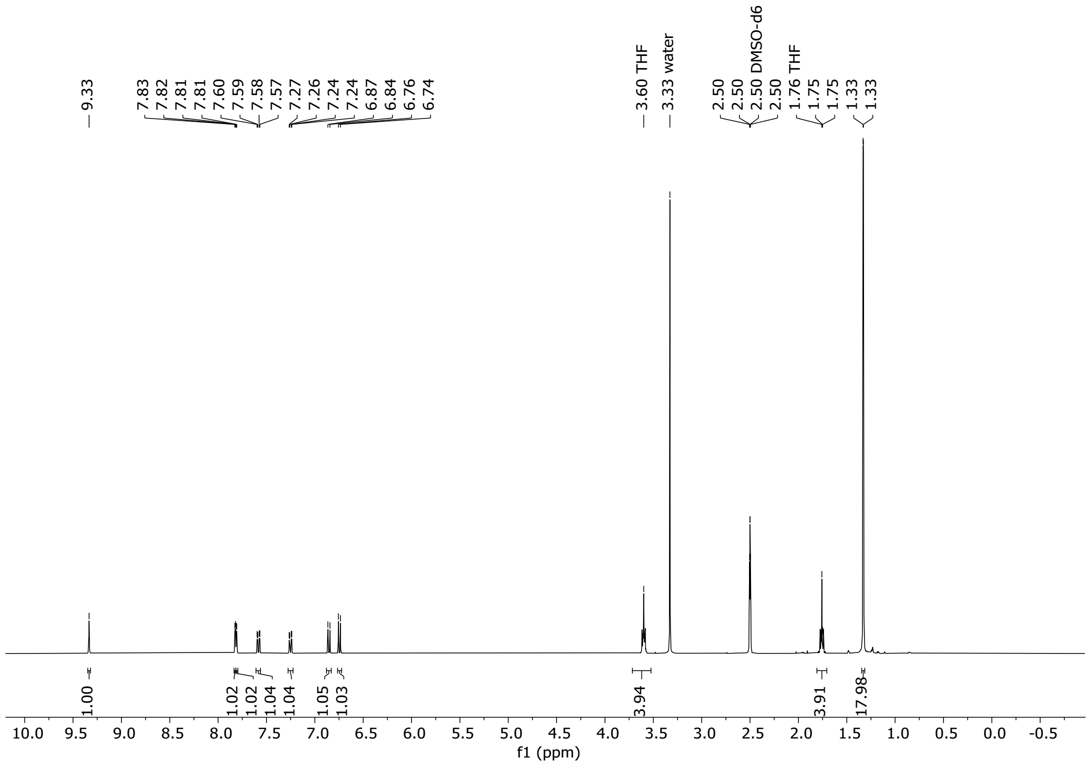

Figure S 25: Complex $\mathbf{5}^{tBu}$ ^{1}H NMR spectrum (400 MHz, DMSO-d_6, 25 °C).

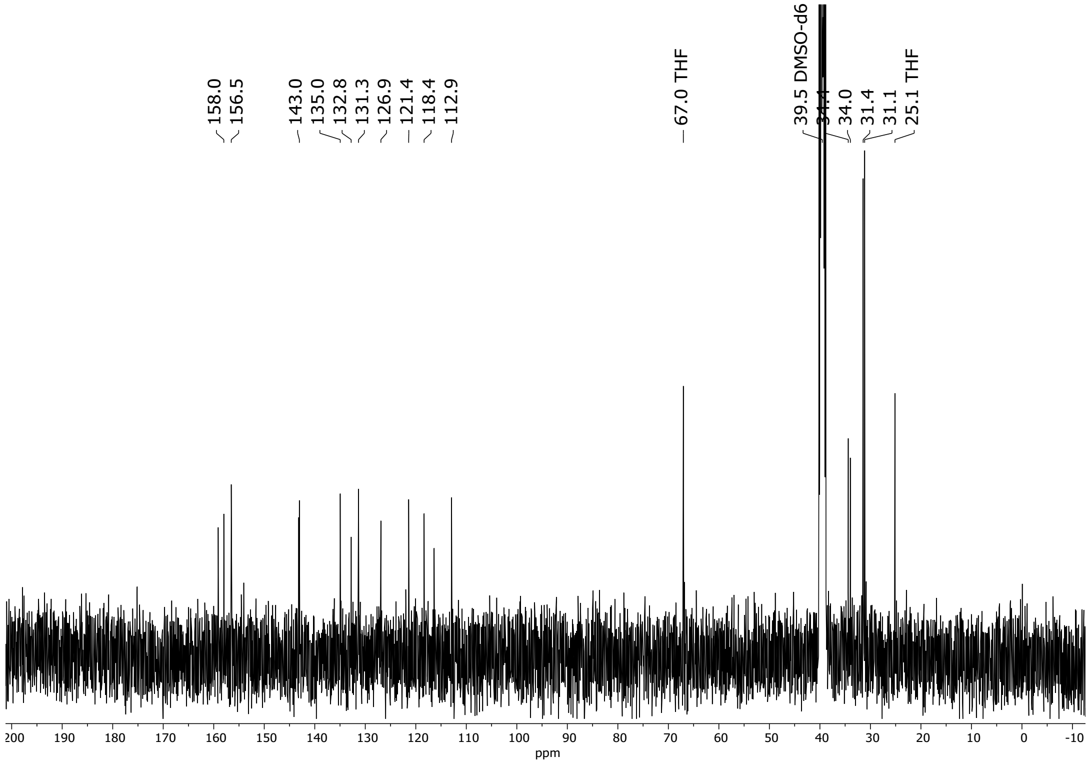

Figure S 26: Complex $\mathbf{5}^{tBu}$ ^{13}C {^{1}H} NMR spectrum (101 MHz, DMSO-d_6, 25 °C).

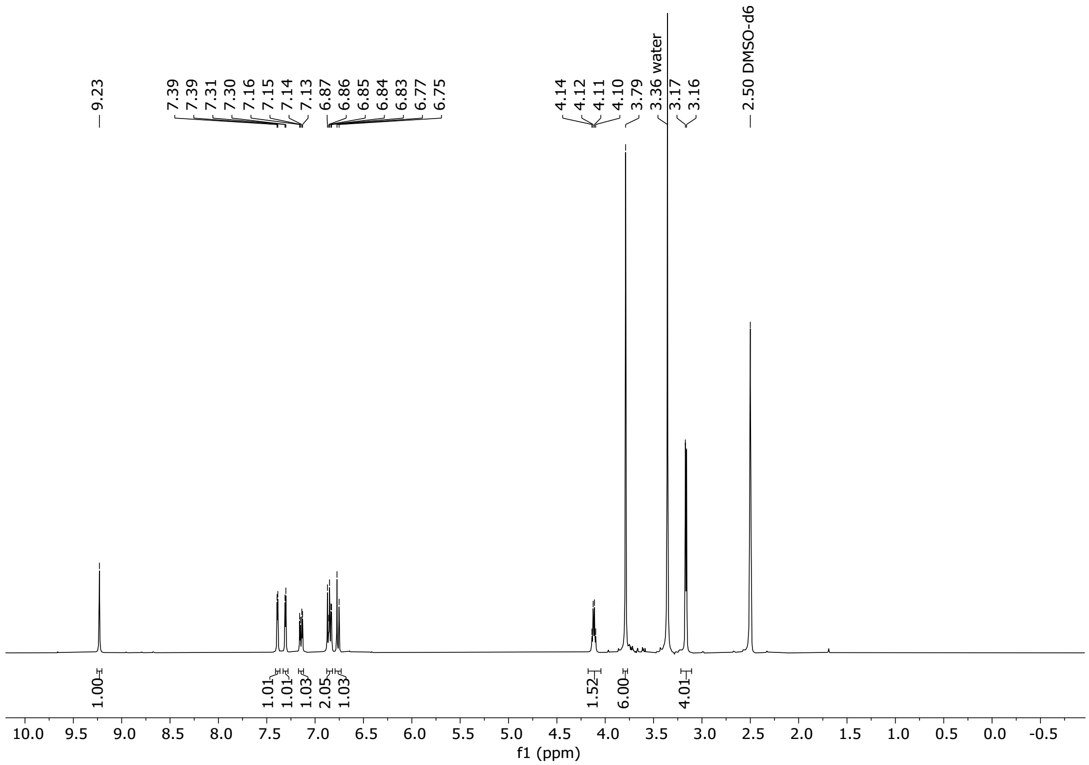

Figure S 27: Complex **6^{OMe}** ^{1}H NMR spectrum (400 MHz, DMSO-d_6, 25 °C).

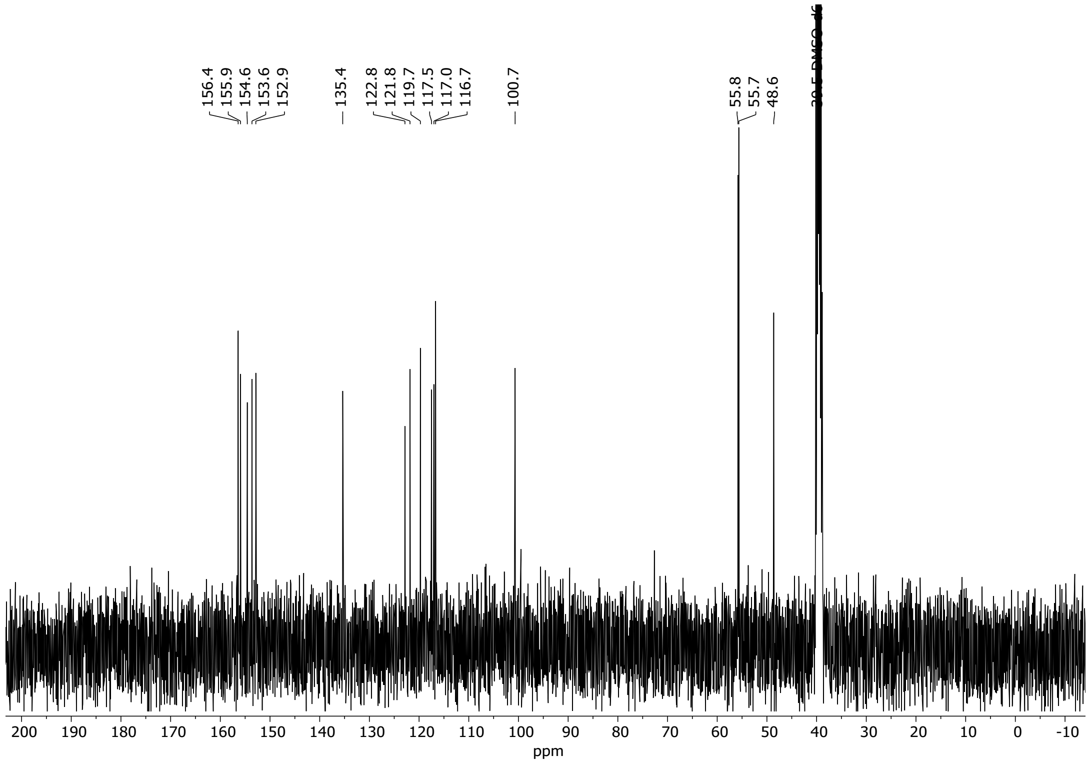

Figure S 28: Complex **6^{OMe}** ^{13}C {^{1}H} NMR spectrum (101 MHz, DMSO-d_6, 25 °C).

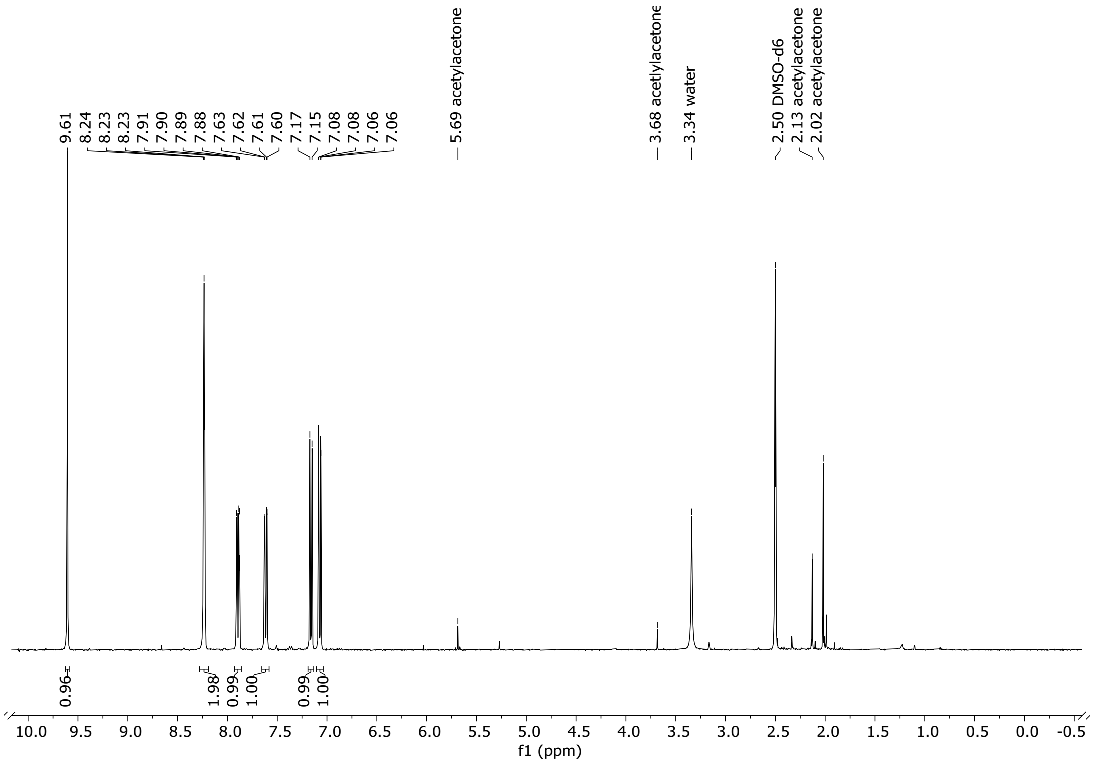

Figure S 29. Complex **7**^{CF3} ^{1}H NMR spectrum (400 MHz, DMSO-d_6, 25 °C).

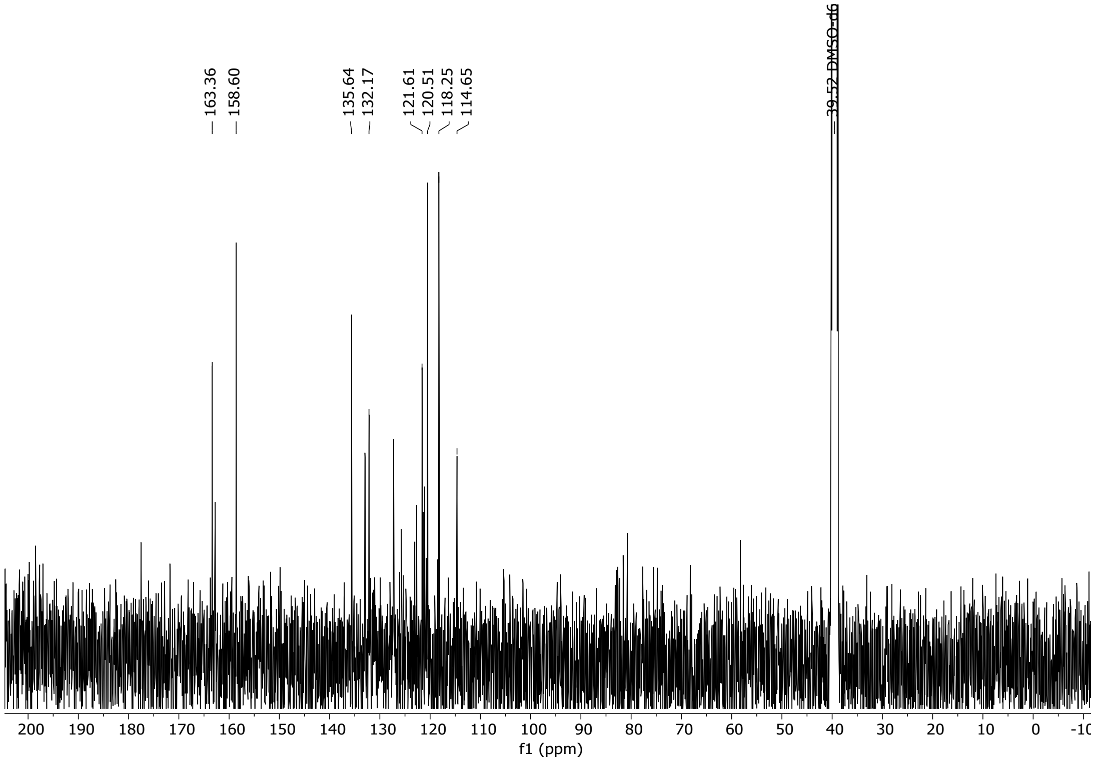

Figure S 30. Complex **7**^{CF3} ^{13}C {^{1}H} NMR spectrum (101 MHz, DMSO-d_6, 25 °C).

UV-visible spectra (in DMSO) of complexes 1^{H} – 7^{CF3}

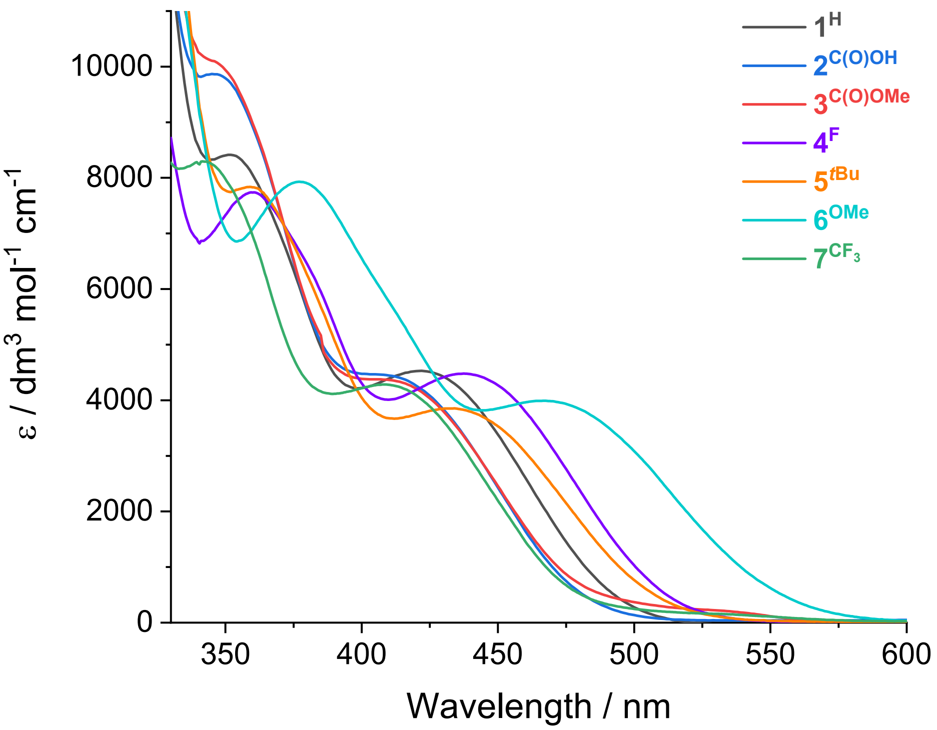

Figure S 31: UV-visible spectra of complexes $\mathbf{1}^{\mathbf{H}}$ (black), $\mathbf{2}^{\mathbf{C(O)OH}}$ (blue), $\mathbf{3}^{\mathbf{C(O)OMe}}$ (red), $\mathbf{4}^{\mathbf{F}}$ (purple), $\mathbf{5}^{\boldsymbol{t}\mathbf{Bu}}$ (orange), and $\mathbf{6}^{\mathbf{OMe}}$ (cyan) and $\mathbf{7}^{\mathbf{CF3}}$ (green).

Crystal structures of complexes 1^{H}_{DMSO}, $3^{C(O)OMe}_{DMSO}$, 4^{F}_{THF}, 6^{OMe}, and 7^{CF3}_{H2O}

Crystals of $\mathbf{1^{H}_{DMSO}}$ were obtained from slow Et_2O vapour diffusion into saturated solution of $\mathbf{1^{H}}$ in DMSO. Crystal parameters are given in Table S1.

Refinement Special Details. The structure exhibited considerable disorder. For the complex moiety all atoms, except oxo oxygens, were disordered and modelled in two positions with refined occupancies of 0.5697:0.4303(19). Pairs of overlapping C-C bond lengths within the aromatic rings were restrained to be equal (e.g.C1-C6 and C8A-C13A). The ADP of disordered pairs of atoms were constrained to be equal (e.g. O3 & O4A, C1 & C13A etc). The ADPs of C1, C6, C8, C13, C1A, C8A & C13A were constrained to be approximately isotropic. The DMSO of crystallisation was also disordered and modelled in two sites with refined occupancies of 0.9220:0.0780(18) and equivalent bond lengths were restrained to be equal as were pairs of The ADP'S (e.g. C16 & C16A).

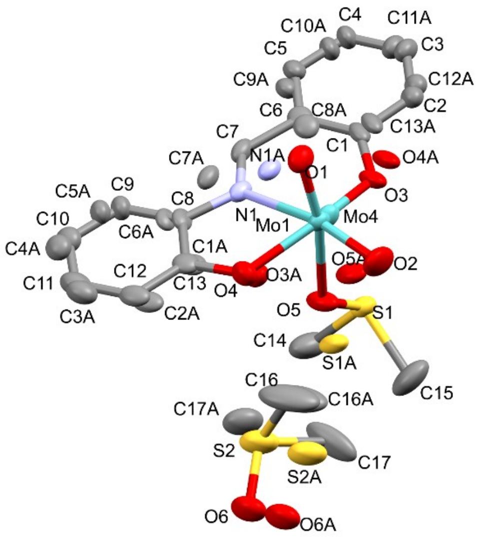

Figure S 32. ORTEP plot (50 % probability) of $\mathbf{1^{H}_{DMSO}}$ (H atoms omitted for clarity; C, grey; N, lavender; O, red; S, yellow; Mo, cyan).

Crystals of $\mathbf{3^{C(O)OMe}_{DMSO}}$ were obtained from slow MeOH vapour diffusion into saturated solution of $\mathbf{3^{C(O)OMe}}$ in DMSO. Crystal parameters are given in Table S1.

Refinement Special Details. The crystal was a non-merohedral twin modelled using two components in a refined ratio of 0.5773:0.4227(8). For both of the complexes present in the asymmetric unit the ligand is disordered. The imine moiety was modelled in two positions in refined ratios of 0.785:0.215(7) and 0.893:0.107(8). The ADPs of disordered pairs of atoms were constrained to be equal (C8 & C8a, N1 & N1a, C27 & C27a, N2 & N2a). Attempts to model the other atoms of the ligand in the minor position proved unsuccessful.

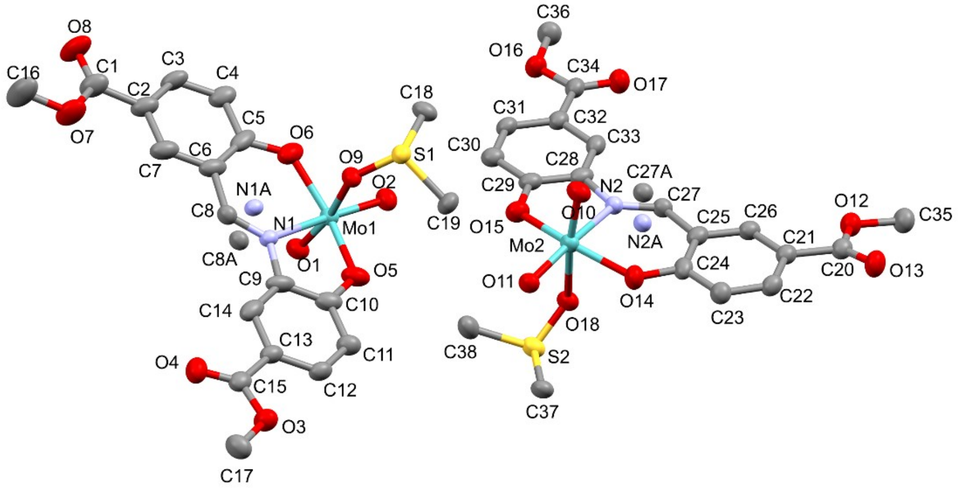

Figure S 33: ORTEP plot (50 % probability) of $\mathbf{3}^{\mathbf{C(O)OMe}}_{\mathbf{DMSO}}$ (H atoms omitted for clarity; C, grey; N, lavender; O, red; S, yellow; Mo, cyan).

Crystals of $\mathbf{4}^{\mathbf{F}}_{\mathbf{THF}}$ were obtained from heptane vapour diffusion into saturated solution of $\mathbf{4}^{\mathbf{F}}$ in THF. Crystal parameters are given in Table S1.

Refinement Special Details. The ligand was disordered with the imine group being modelled in two positions with refined occupancies of 0.532:0.468(17). The ADPs of disordered pairs of atoms were constrained to be equal (C7 & C7A, N1 & N1A). The bound tetrahydrofuran (THF) was also disordered with the carbons modelled in two positions with refined occupancies of 0.520:0.480(6).The asymmetric unit contained half a THF of crystallisation which was disordered about the centre of inversion. C-C bond lengths were restrained to be 1.51 angstroms and the C-O bond-lengths to 1.42 angstroms.

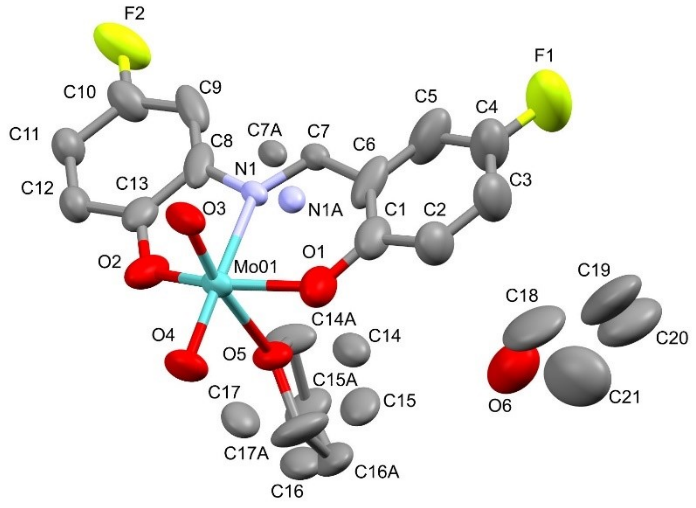

Figure S 34: ORTEP plot (50 % probability) of $\mathbf{4}^{\mathbf{F}}_{\mathbf{THF}}$ (H atoms omitted for clarity; C, grey; N, lavender; O, red; F, chartreuse; Mo, cyan).

Crystals of **6^{OMe}** were obtained from EtOAc/MeOH vapour diffusion into saturated solution of **6^{OMe}** in THF. Crystal parameters are given in Table S1.

Refinement Special Details. The ligand was disordered and modelled in two positions with one methoxyphenyl in a common site but the other and the imine bridge modelled in two sites with refined occupancies of 0.638:0.362(3). The ADP of pairs of proximal atoms were constrained to be equal as follows: C1 & C1A, C2 & C2A C3 & C3A, C4 & C4A, C5 & C5A, C6 & C6A, C7 & C7A, C14 & C14A, O3 & O3A, O4 & O4A, N1 & N1A.

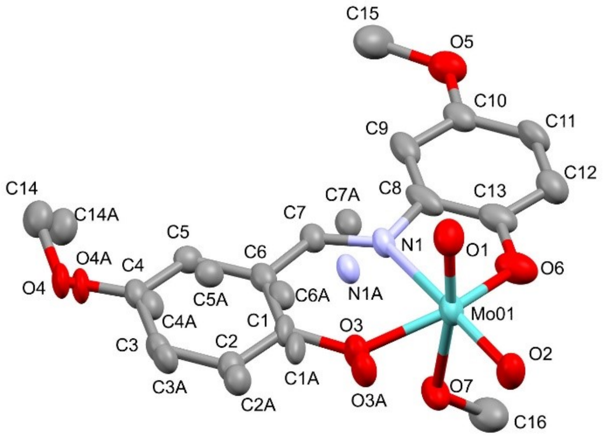

Figure S 35: ORTEP plot (50 % probability) of **6^{OMe}** (H atoms omitted for clarity; C, grey; N, lavender; O, red; Mo, cyan).

Crystals of **7^{CF3}$_{H2O}$** were obtained from heptane vapour diffusion into saturated solution of **7^{CF3}** in THF. Crystal parameters are given in Table S1.

Refinement Special Details. The ligand is disordered and modelled over two sites which required the imine to be modelled in two sites with refined occupancies of 0.547:0.453(4). The ADPs of pairs of disordered atoms were constrained to be equal (N1 & N1a, C7 & C7a).The crystal was also non-merohedrally twinned and modelled in two components with refined occupancies of 0.5076:0.4924(8).

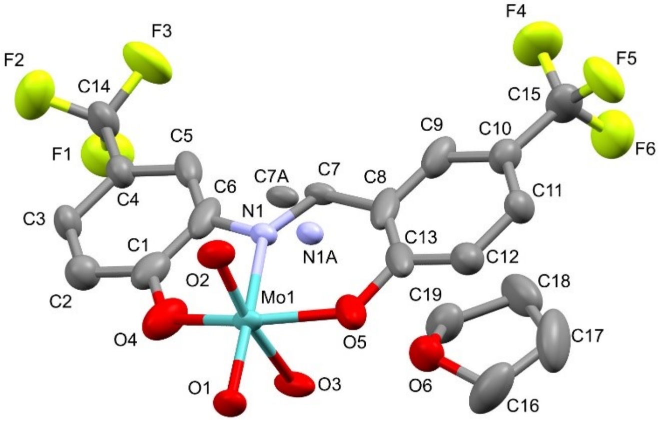

Figure S 36: ORTEP plot (50 % probability) of **7^{CF3} $_{H2O}$** (H atoms omitted for clarity; C, grey; N, lavender; O, red; F, chartreuse; Mo, cyan).

Table S 1. Crystallographic data and structure refinement for **1^{H}_{DMSO}**, **$3^{C(O)OMe}_{DMSO}$**, **4^{F}_{THF}**, **6^{OMe}**, **7^{CF3}_{H2O}**.

Identification code	**1^{H}_{DMSO}**	**$3^{C(O)OMe}_{DMSO}$**	**4^{F}_{THF}**
CCDC deposition number	2312692	2312691	2312695
Empirical formula	$C_{17}H_{21}MoNO_6S_2$	$C_{19}H_{19}NO_9SMo$	$C_{19}H_{19}F_2MoNO_{5.5}$
Formula weight	495.41	533.35	483.29
Temperature/K	109.95 (10)	110.00 (10)	110.00 (10)
Crystal system	Monoclinic	Triclinic	triclinic
Space group	$P2_1/c$	P-1	P-1
a/Å	12.51400 (14)	7.97347 (14)	6.7559 (4)
b/Å	6.72111 (8)	14.0397 (3)	12.1951 (7)
c/Å	23.5861 (3)	18.8868 (4)	12.2098 (7)
α/°	90	89.8034 (16)	71.970 (5)
β/°	93.9945 (11)	87.7720 (16)	80.752 (5)
γ/°	90	74.1423 (16)	77.252 (5)
Volume/$Å^3$	1978.96 (4)	2032.23 (7)	928.32 (10)
Z	4	4	2
ρ_{calc} g/cm^3	1.663	1.743	1.729
μ/mm^{-1}	7.697	6.731	6.283
F(000)	1008.0	1080.0	488.0
Crystal size/mm^3	0.115 × 0.054 × 0.034	0.102 × 0.099 × 0.075	0.2 × 0.19 × 0.05
Radiation	Cu Kα (λ = 1.54184)	Cu Kα (λ = 1.54184)	Cu Kα (λ = 1.54184)
2Θ range for data collection/°	7.082 to 134.142	8.022 to 134.244	7.652 to 134.144
Index ranges	-11 ≤ h ≤ 14,<br>-8 ≤ k ≤ 7,<br>-28 ≤ l ≤ 28	-9 ≤ h ≤ 9,<br>-16 ≤ k ≤ 16,<br>-22 ≤ l ≤ 22	-8 ≤ h ≤ 6,<br>-14 ≤ k ≤ 14,<br>-14 ≤ l ≤ 11
Reflections collected	12118	12797	9376
Independent reflections	3512<br>[R_{int} = 0.0293,<br>R_{sigma} = 0.0289]	12797<br>[R_{int} = ?,<br>R_{sigma} = 0.0315]	3307<br>[R_{int} = 0.0266,<br>R_{sigma} = 0.0287]
Data/restraints/parameters	3512/88/349	12797/0/582	3307/5/336
Goodness-of-fit on F^2	1.039	1.064	1.056
Final R indexes [I>=2σ (I)]	R_1 = 0.0258, wR_2 = 0.0643	R_1 = 0.0440, wR_2 = 0.1166	R_1 = 0.0279, wR_2 = 0.0660
Final R indexes [all data]	R_1 = 0.0287, wR_2 = 0.0658	R_1 = 0.0490, wR_2 = 0.1203	R_1 = 0.0292, wR_2 = 0.0668
Largest diff. peak/hole / e $Å^{-3}$	0.63/-0.36	0.69/-0.80	0.43/-0.50

Table S 1. Continued.

Identification code	**6**^{OMe}	**7**$^{CF3}_{H2O}$
CCDC deposition number	2312693	2312694
Empirical formula	$C_{16}H_{17}NO_7Mo$	$C_{19}H_{17}F_6MoNO_6$
Formula weight	431.24	565.28
Temperature/K	110.00 (10)	110.00 (10)
Crystal system	monoclinic	monoclinic
Space group	$P2_1/c$	$P2_1/c$
a/Å	6.93153 (10)	13.3516 (3)
b/Å	11.74560 (18)	14.8110 (3)
c/Å	20.3314 (3)	11.4468 (3)
α/°	90	90
β/°	94.9309 (13)	112.217 (3)
γ/°	90	90
Volume/Å^3	1649.15 (4)	2095.56 (9)
Z	4	4
ρ_{calc} g/cm^3	1.737	1.792
μ/mm^{-1}	6.876	5.975
F(000)	872.0	1128.0
Crystal size/mm^3	0.21 × 0.139 × 0.102	0.193 × 0.104 × 0.094
Radiation	Cu Kα (λ = 1.54184)	Cu Kα (λ = 1.54184)
2Θ range for data collection/°	8.702 to 134.078	7.152 to 134.068
Index ranges	-8 ≤ h ≤ 6,<br>-14 ≤ k ≤ 9,<br>-24 ≤ l ≤ 24	-15 ≤ h ≤ 15,<br>-17 ≤ k ≤ 17,<br>-11 ≤ l ≤ 13
Reflections collected	9124	6751
Independent reflections	2945<br>[R_{int} = 0.0334,<br>R_{sigma} = 0.0329]	6751<br>[R_{int} = ?,<br>R_{sigma} = 0.0172]
Data/restraints/parameters	2945/0/261	6751/0/307
Goodness-of-fit on F^2	1.066	1.058
Final R indexes [I>=2σ (I)]	R_1 = 0.0283, wR_2 = 0.0665	R_1 = 0.0297, wR_2 = 0.0773
Final R indexes [all data]	R_1 = 0.0303, wR_2 = 0.0676	R_1 = 0.0326, wR_2 = 0.0793
Largest diff. peak/hole / e Å^{-3}	0.46/-0.70	0.82/-0.51

Photocatalytic testing of complexes 1^{H} – 7^{CF3}

^{1}H NMR spectra of photocatalytic reactions

a) b)

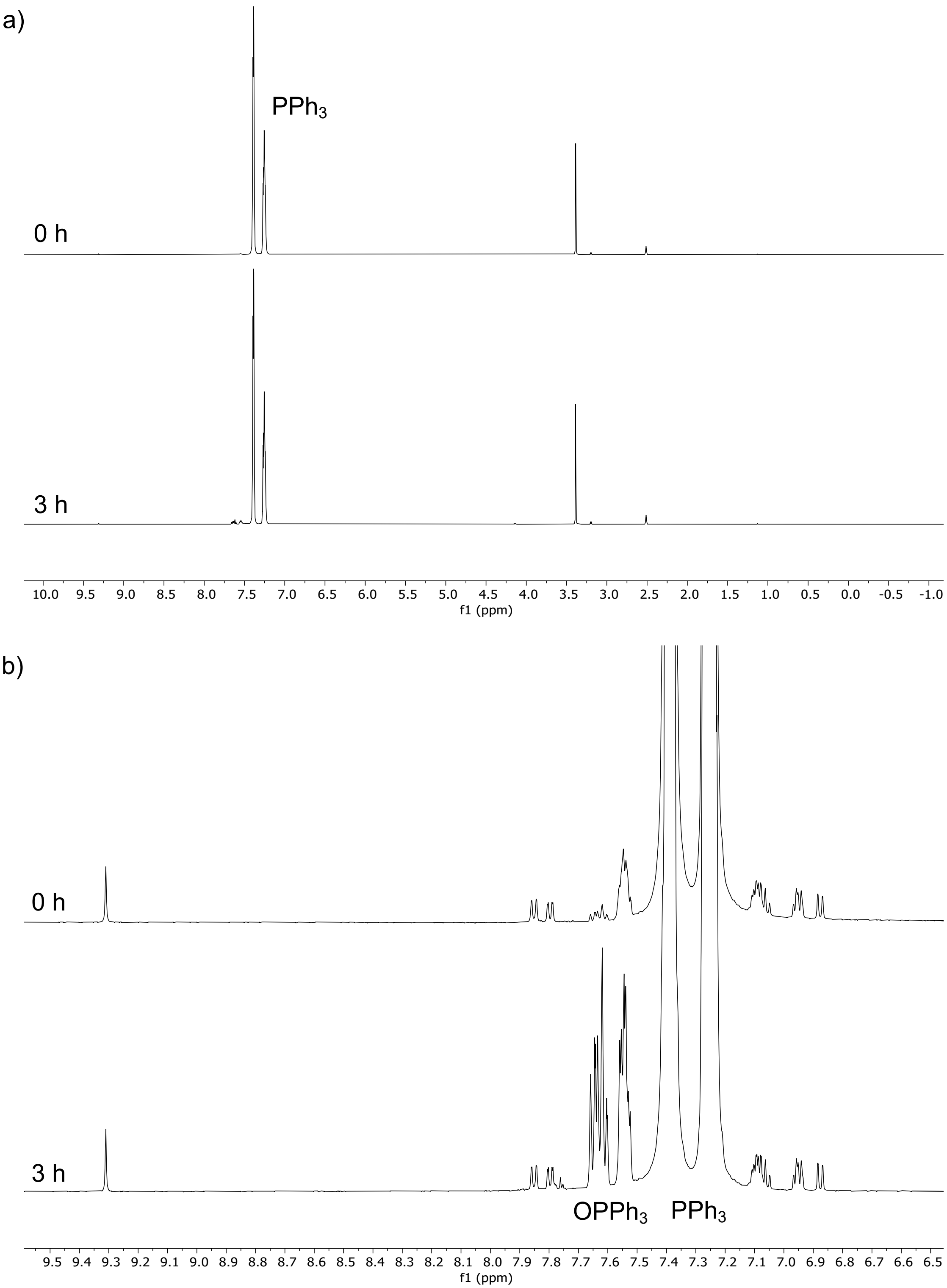

Figure S 37: ^{1}H NMR spectra (500 MHz, DMSO-d_6, 25 °C) of photocatalytic testing with **1^{H}** (1 mmol), PPh_3 (300 mmol) in DMSO-d_6 (0.7 mL) after at 0 h and 3 h of irradiation; a) full spectra; b) aromatic region.

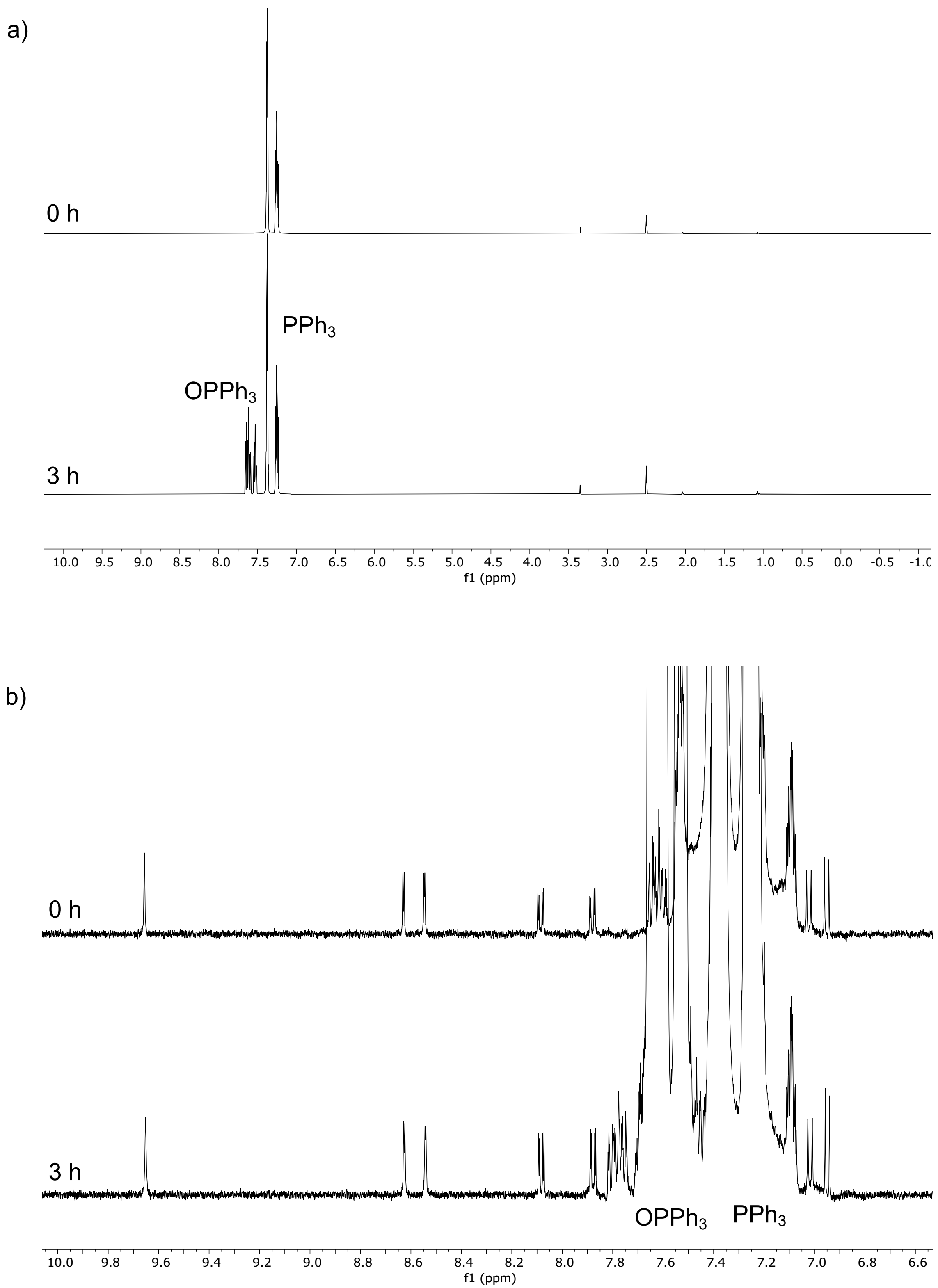

Figure S 38: ^{1}H NMR spectra (500 MHz, DMSO-d_6, 25 °C) of photocatalytic testing with $\mathbf{2}^{\mathbf{C(O)OH}}$ (1 mmol), PPh_3 (300 mmol) in DMSO-d_6 (0.7 mL) after at 0 h and 3 h of irradiation; a) full spectra; b) aromatic region.

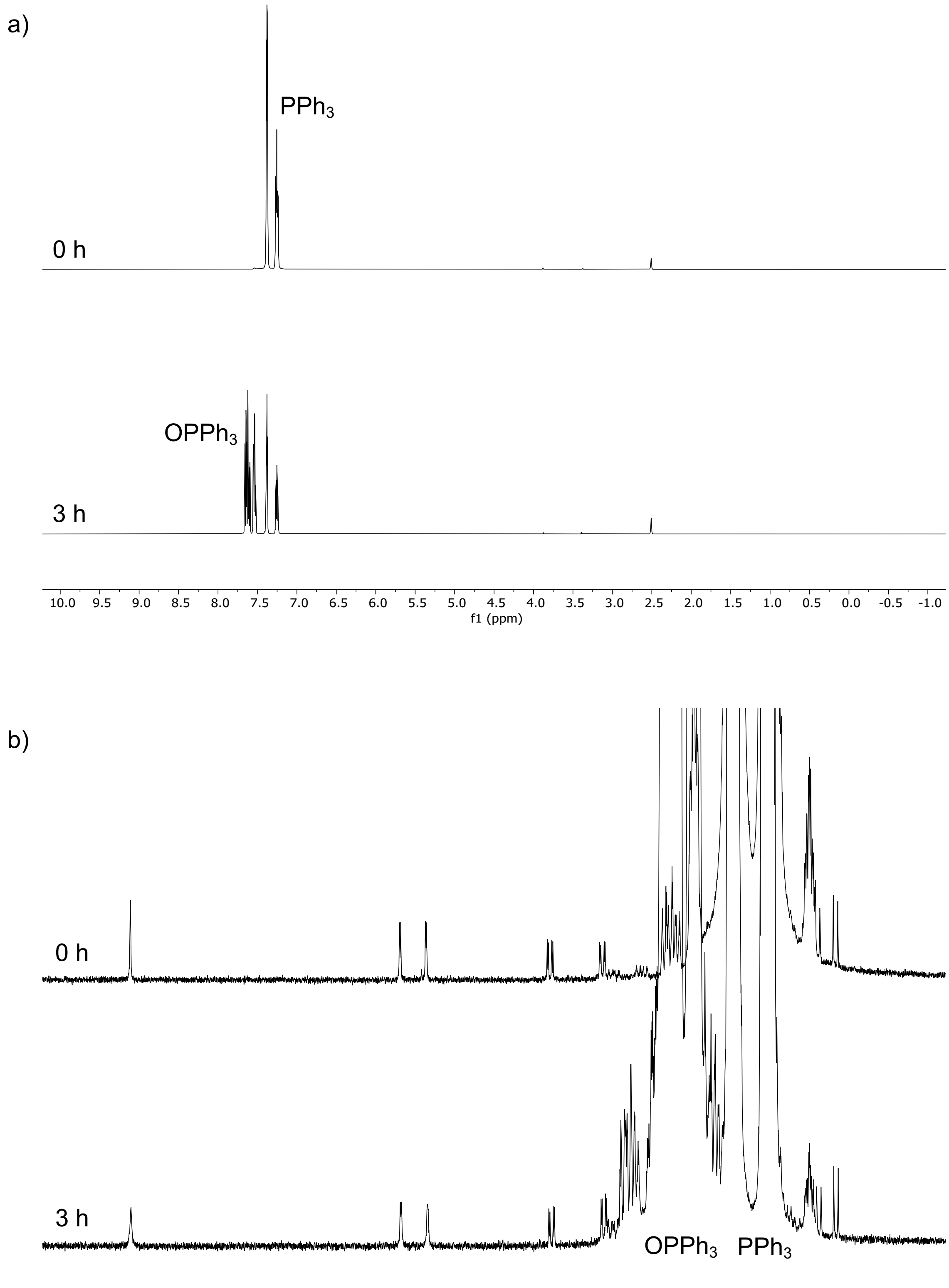

Figure S 39: ^{1}H NMR spectra (500 MHz, DMSO-d_6, 25 °C) of photocatalytic testing with **3**$^{C(O)OMe}$ (1 mmol), PPh_3 (300 mmol) in DMSO-d_6 (0.7 mL) after at 0 h and 3 h of irradiation; a) full spectra; b) aromatic region.

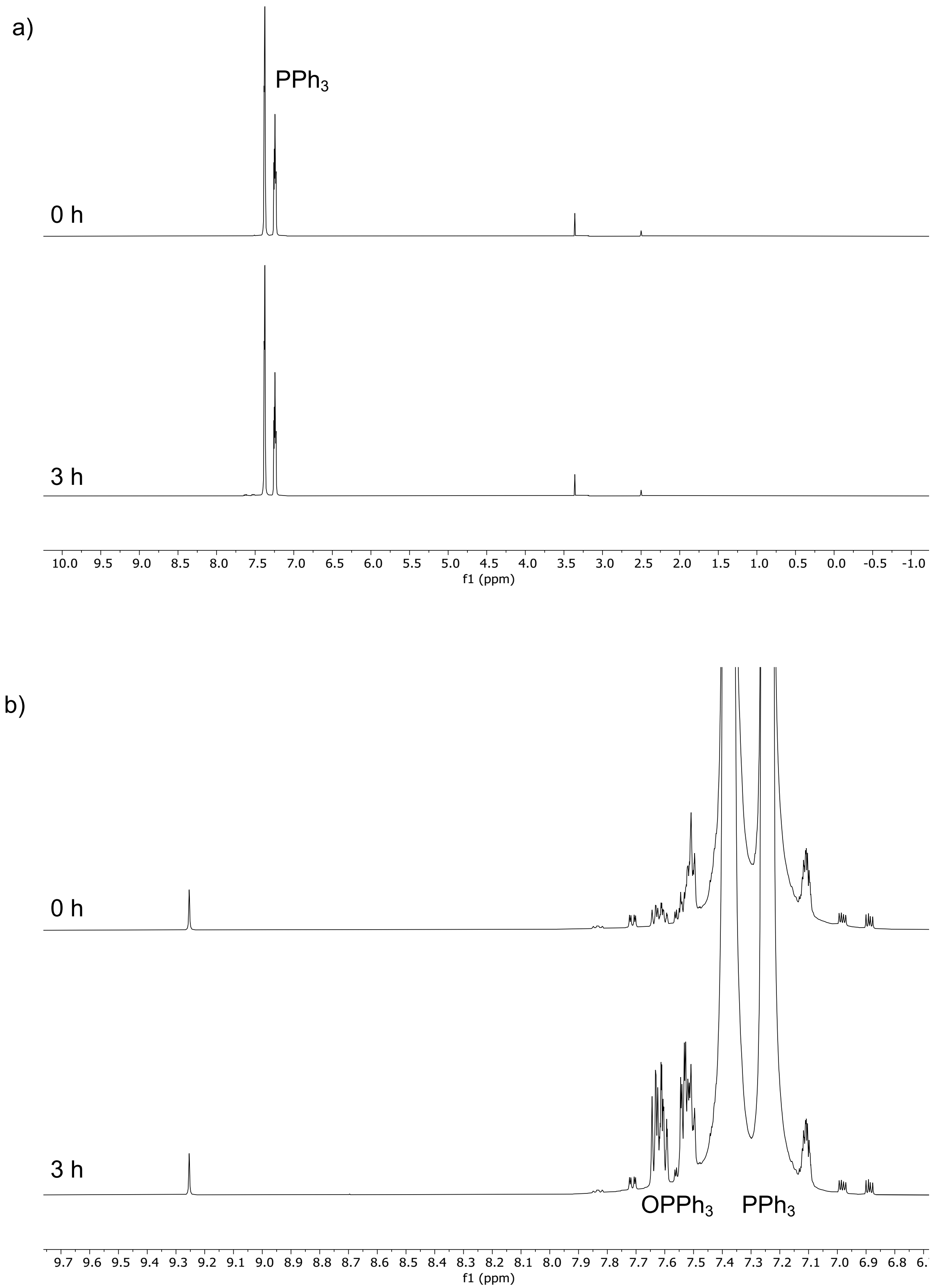

Figure S 40: ^{1}H NMR spectra (500 MHz, DMSO-d$_6$, 25 °C) of photocatalytic testing with **4^{F}** (1 mmol), PPh_3 (300 mmol) in DMSO-d$_6$ (0.7 mL) after at 0 h and 3 h of irradiation; a) full spectra; b) aromatic region..

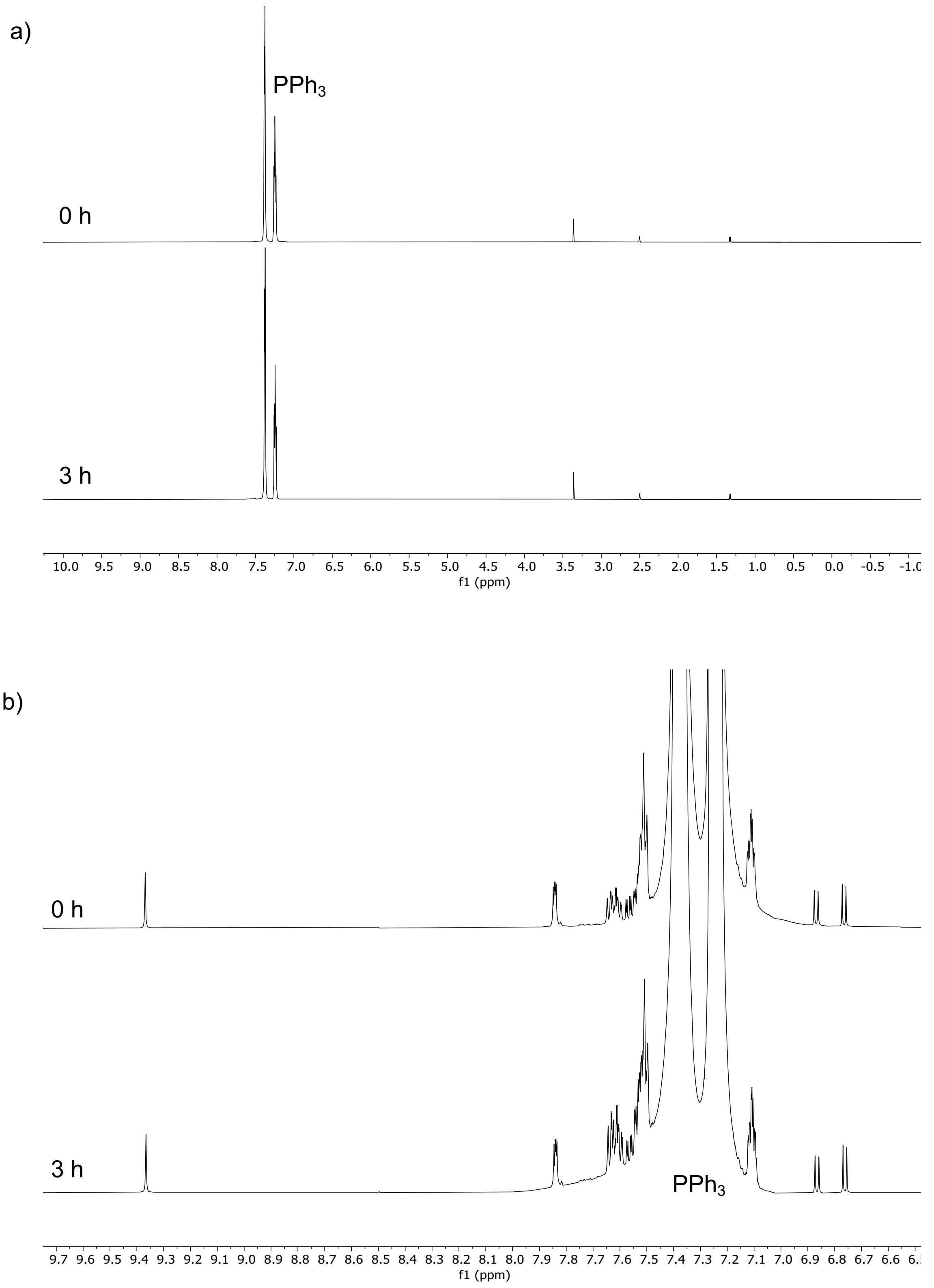

Figure S 41: ^{1}H NMR spectra (500 MHz, DMSO-d$_6$, 25 °C) of photocatalytic testing with **5^{tBu}** (1 mmol), PPh_3 (300 mmol) in DMSO-d$_6$ (0.7 mL) after at 0 h and 3 h of irradiation; a) full spectra; b) aromatic region.

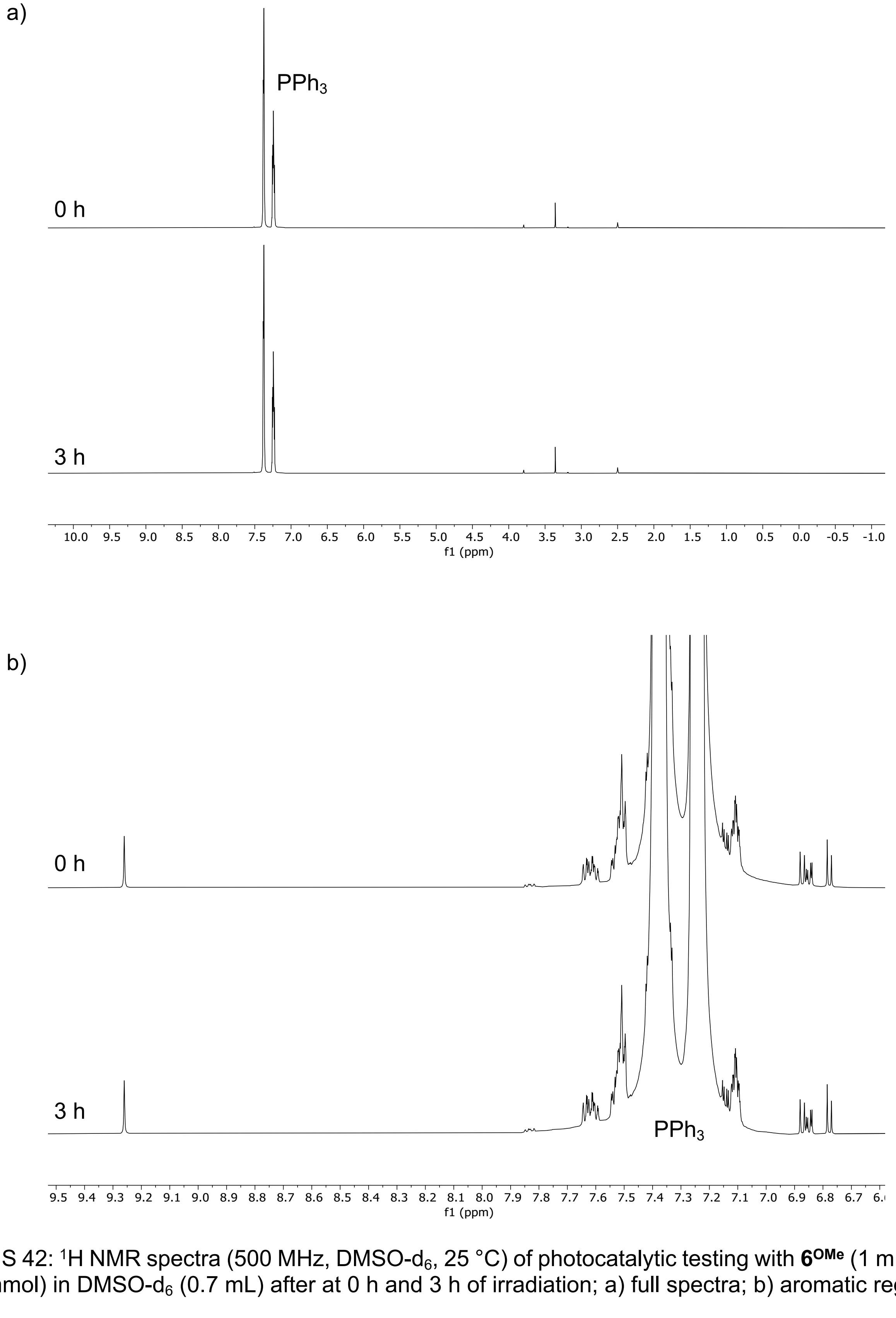

Figure S 42: ^{1}H NMR spectra (500 MHz, DMSO-d_6, 25 °C) of photocatalytic testing with **6^{OMe}** (1 mmol), PPh_3 (300 mmol) in DMSO-d_6 (0.7 mL) after at 0 h and 3 h of irradiation; a) full spectra; b) aromatic region.

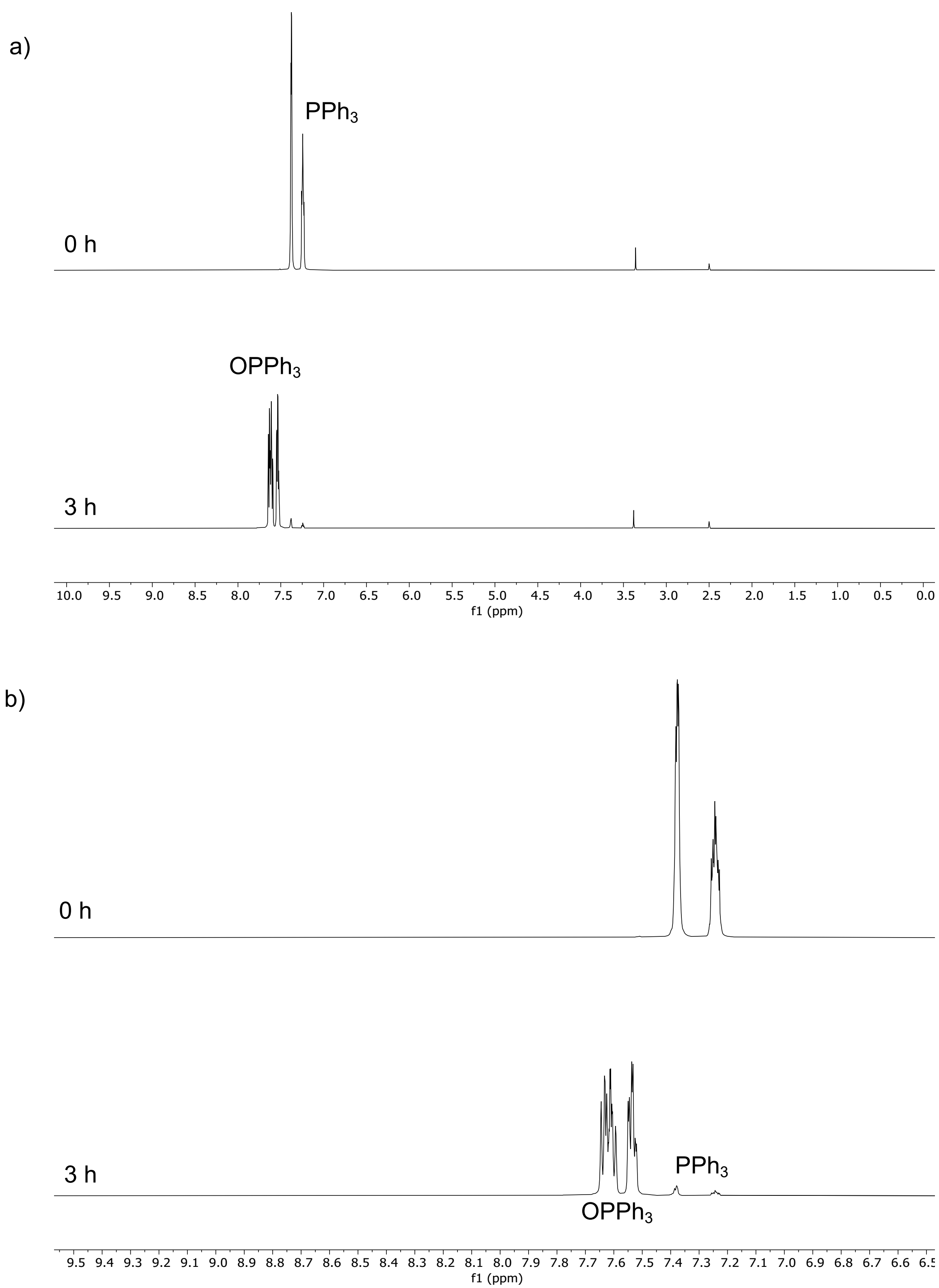

Figure S 43. ^{1}H NMR spectra (500 MHz, DMSO-d_6, 25 °C) of photocatalytic testing with $\mathbf{7^{CF3}}$ (1 mmol), PPh_3 (300 mmol) in DMSO-d_6 (0.7 mL) after at 0 h and 3 h of irradiation; a) full spectra; b) aromatic region.

Photocatalytic testing of complexes 1^{H}, $2^{C(O)OH}$, $3^{C(O)OMe}$, 7^{CF3} as a function of time.

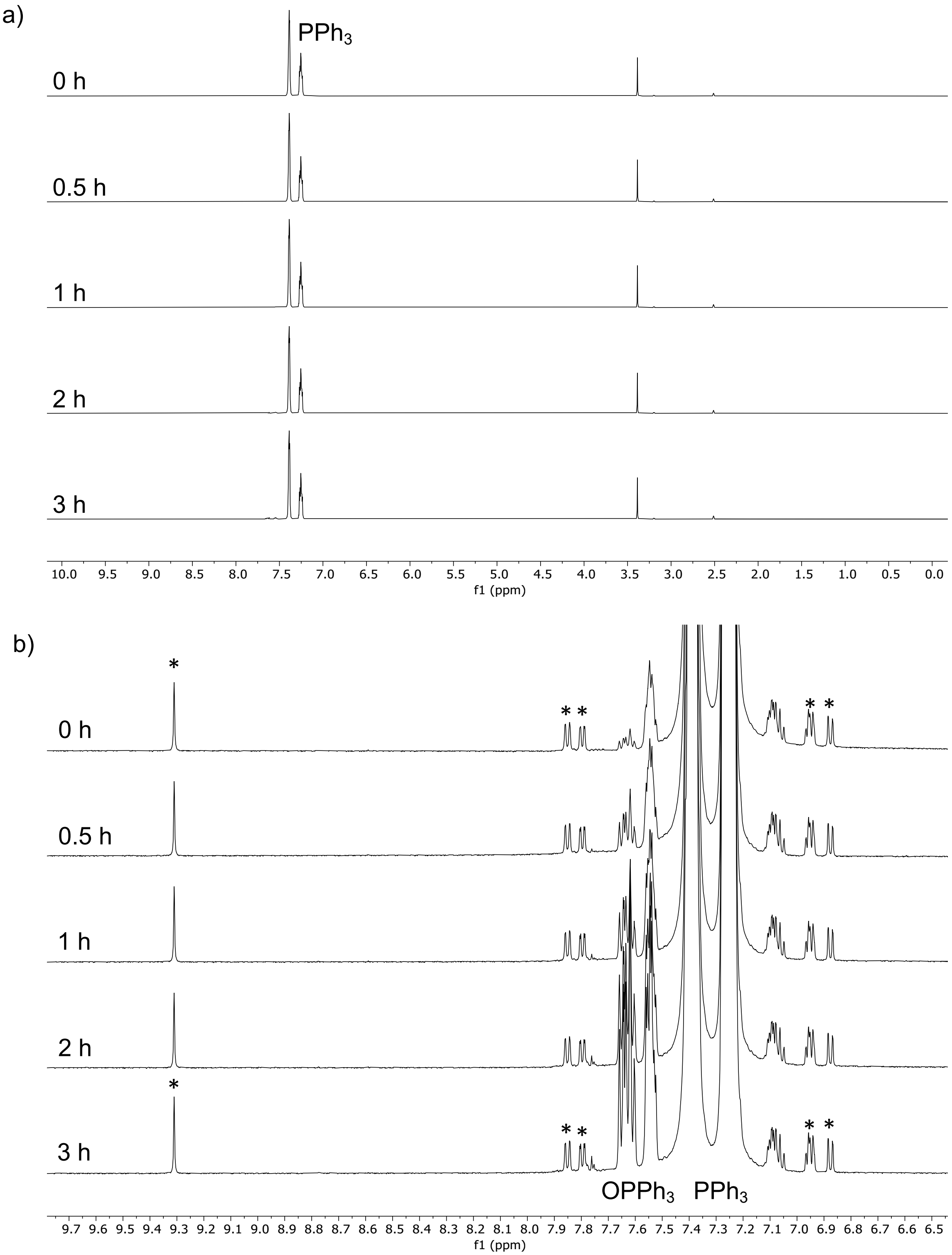

Figure S 44: ^{1}H NMR spectra (500 MHz, DMSO-d_6, 25 °C) of photocatalytic testing with $\mathbf{1^{H}}$ (1 mmol), PPh_3 (300 mmol) in DMSO-d_6 (0.7 mL) at selected timepoints during irradiation; a) full spectra; b) aromatic region (identifiable ^{1}H signals of $\mathbf{1^{H}}$ in the aromatic region are indicated by an asterisk).

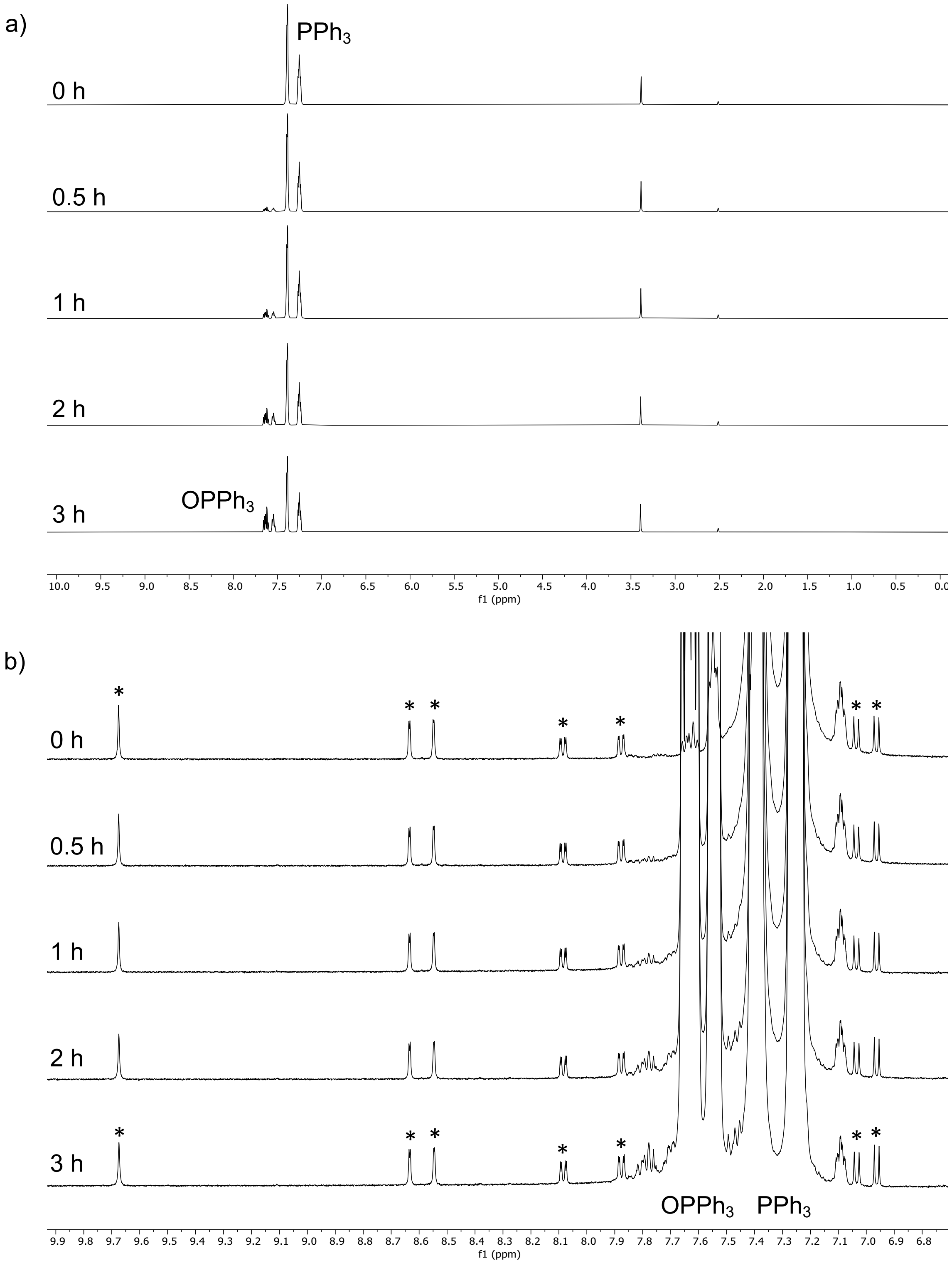

Figure S 45. ^{1}H NMR spectra (500 MHz, DMSO-d_6, 25 °C) of photocatalytic testing with $\mathbf{2}^{C(O)OH}$ (1 mmol), PPh_3 (300 mmol) in DMSO-d_6 (0.7 mL) at selected timepoints during irradiation; a) full spectra; b) aromatic region (identifiable ^{1}H signals of $\mathbf{2}^{C(O)OH}$ in the aromatic region are indicated by an asterisk).

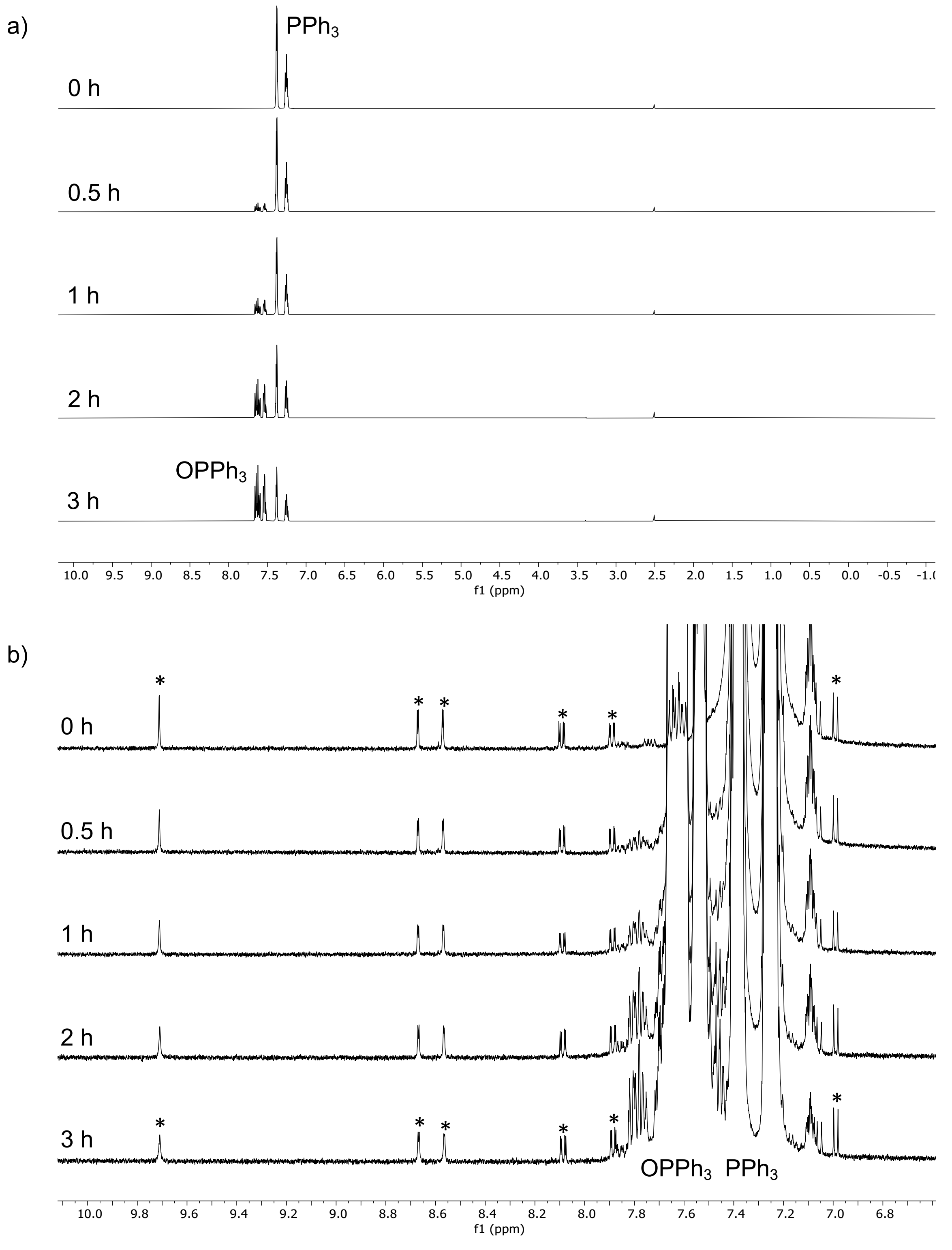

Figure S 46. ^{1}H NMR spectra (500 MHz, DMSO-d$_6$, 25 °C) of photocatalytic testing with **3$^{C(O)OMe}$** (1 mmol), PPh_3 (300 mmol) in DMSO-d$_6$ (0.7 mL) at selected timepoints during irradiation; a) full spectra; b) aromatic region (identifiable ^{1}H signals of **3$^{C(O)OMe}$** in the aromatic region are indicated by an asterisk).

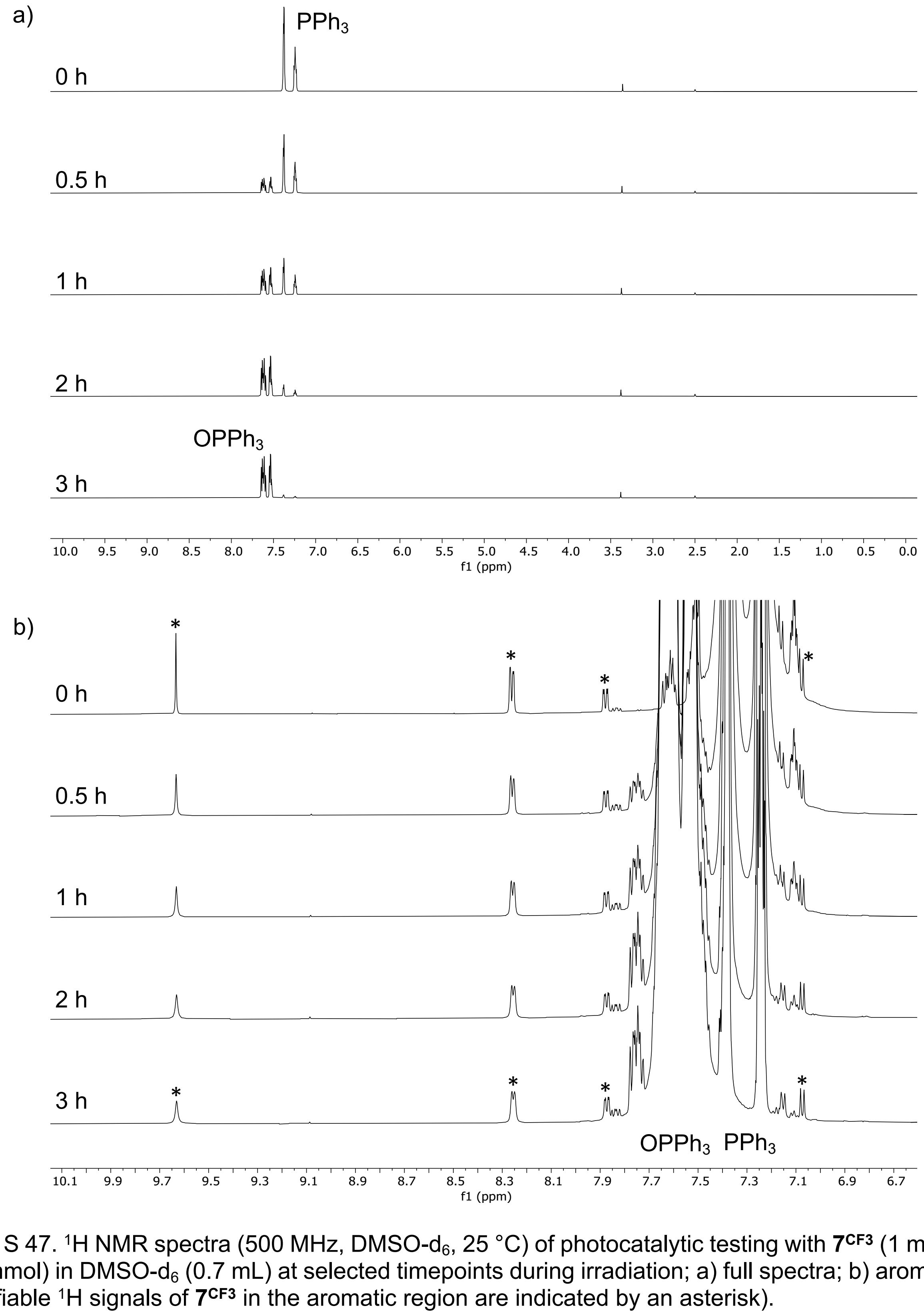

Figure S 47. ^{1}H NMR spectra (500 MHz, DMSO-d$_6$, 25 °C) of photocatalytic testing with **7^{CF3}** (1 mmol), PPh$_3$ (300 mmol) in DMSO-d$_6$ (0.7 mL) at selected timepoints during irradiation; a) full spectra; b) aromatic region (identifiable ^{1}H signals of **7^{CF3}** in the aromatic region are indicated by an asterisk).

Photocatalytic control reactions

Table S 2. Photocatalytic control reactions in DMSO-d_6.

Entry	Catalytic species	[Cat] / mM	[PPh_3] / mM	Irradiation	N_2/air	Time / h	*T* / °C	PPh_3 conversion / %[a]	TON	TOF / h^{-1}
1	$\mathbf{3}^{C(O)OMe}$	1	300	dark	N_2	3	25	0	-	-
2	$\mathbf{3}^{C(O)OMe}$	1	300	dark	N_2	3	45	0	-	-
3	$\mathbf{H_2L1}^{H}$	1	300	410 nm	N_2	3	25	0	-	-
4	$\mathbf{H_2L2}^{C(O)OH}$	1	300	410 nm	N_2	3	25	2	6	2
5	$\mathbf{H_2L3}^{C(O)OMe}$	1	300	410 nm	N_2	3	25	3	9	3
6[b]	$\mathbf{3}^{C(O)OMe}$	1	300	410 nm	N_2	3	25	73	219	73
7	$\mathbf{3}^{C(O)OMe}$	1	300	410 nm	air	3	25	83	249	83
8	None	-	300	dark	N_2	3	25	0	-	-
9	None	-	300	dark	N_2	3	45	0	-	-
10	None	-	300	410 nm	N_2	3	25	0	-	-
11[b]	None	-	300	410 nm	N_2	3	25	2	-	-
12	None	-	300	410 nm	air	3	25	8	-	-

[a]Conversions calculated from ^1H NMR resonances for PPh_3 and $OPPh_3$ species after 3 hours of continuous irradiation (or 3 hours in the dark/at elevated temperature). [b]300 mM of H_2O added to the sample.

Hammett analysis of substituent effects on substrate conversion for complexes $\mathbf{1^{H}}$ – $\mathbf{7^{CF3}}$

Table S 3. OAT conversions of complexes $\mathbf{1^{H}}$ – $\mathbf{7^{CF3}}$ correlated to σ_p parameters.[28]

Catalyst	σ_p[a] (2 × σ_p)	σ_p^{-}[b] (2 × σ_p^{-})	PPh_3 conversion / %
$\mathbf{1^{H}}$	0.00 (0.00)	0.00 (0.00)	3
$\mathbf{2^{C(O)OH}}$	0.45 (0.90)	0.77 (1.54)	35
$\mathbf{3^{C(O)OMe}}$	0.45 (0.90)	0.75 (1.50)	78
$\mathbf{4^{F}}$	0.06 (0.12)	-0.03 (-0.06)	2
$\mathbf{5^{tBu}}$	-0.20 (-0.40)	-0.13 (-0.26)	0
$\mathbf{6^{OMe}}$	-0.27 (-0.54)	-0.26 (-0.52)	1
$\mathbf{7^{CF3}}$	0.54 (1.08)	0.65 (1.30)	97

[a] σ_p and σ_p^{-} parameters are literature values;[28] [b]σ_p^{-} accounts for the negative mesomeric effect induced by some groups, e.g. C(O)OH and C(O)OMe;[28] [c]value in brackets is the summed value accounting for two substituents on the SAP ligand.

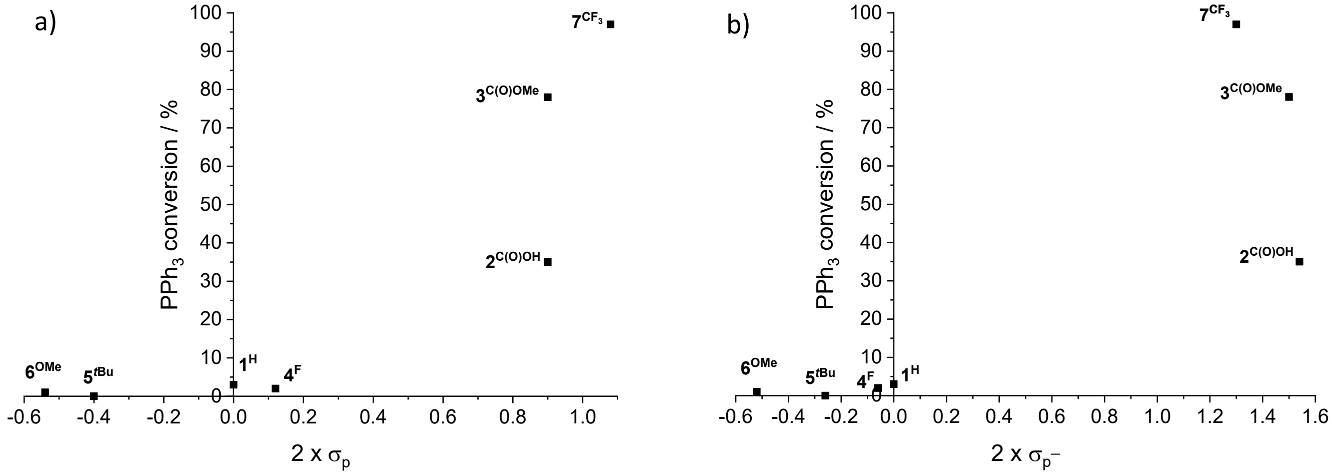

Figure S 48: Hammett plots: a) PPh_3 conversions vs 2 × σ_p; b) PPh_3 conversions vs 2 × σ_p^{-}; for complexes $\mathbf{1^{H}}$ – $\mathbf{7^{CF3}}$ after 3 h irradiation.

Kinetics of reactions between 3$^{C(O)OMe}$ or 7^{CF3} and PPh_3, P(*p*-F-Ph)$_3$, and P(*p*-CF_3-Ph)$_3$ in DMSO-d$_6$

Table S 4: *Pseudo*-first-order rate constants (k_{obs}) for the OAT reaction between PPh_3 , P(*p*-F-Ph)$_3$ and P(*p*-CF_3-Ph)$_3$, respectively, with DMSO-d$_6$ (solvent: DMSO-d$_6$).

	**PPh_3** k_{obs} / h^{-1}	**P(*p*-F-Ph)$_3$** k_{obs} / h^{-1}	**P(*p*-CF_3-Ph)$_3$** k_{obs} / h^{-1}
400 equiv. PR_3:			
**3**^{COOMe}	0.40(1)	0.36(1)	0.17(1)
**7**^{CF3}	0.99(4)	1.16(4)	0.43(1)
200 equiv. PR_3:			
**3**^{COOMe}	0.47 (1)	0.47(1)	0.19(1)
**7**^{CF3}	1.31(1)	1.25(1)	0.54(1)
100 equiv. PR_3:			
**3**^{COOMe}	0.52(5)	0.49(1)	0.19(1)
**7**^{CF3}	1.37(5)	1.47(4)	0.54(1)
**σ_p**	0	0.06	0.53

NB: The decrease in k_{obs} with increasing phosphine equivalents is likely due to the concomitant increase in the viscosity of the reaction medium. σ_p: Hammett constant.[28]

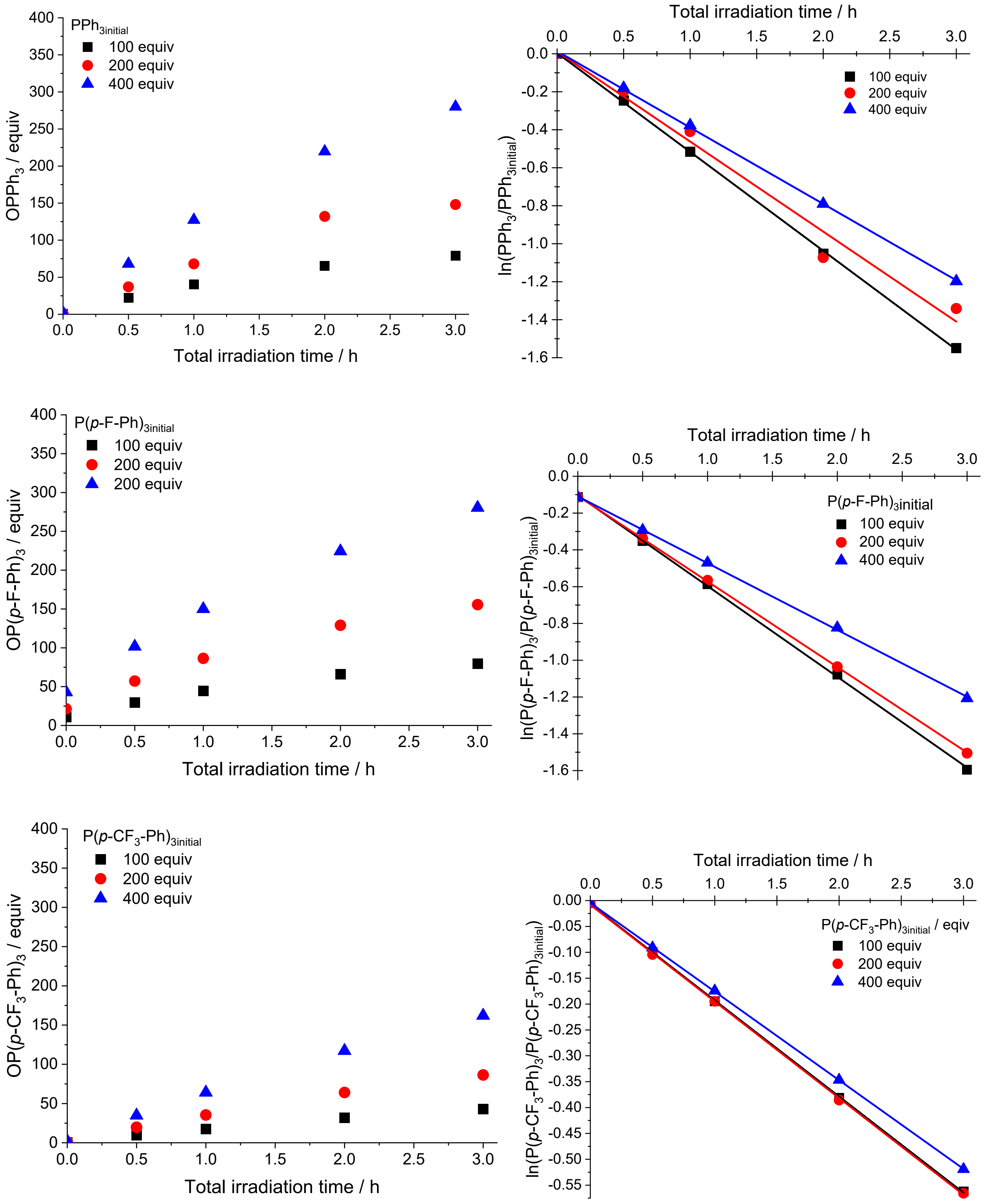

Figure S 49. Kinetics of reactions between $\mathbf{3}^{C(O)OMe}$ and PPh_3, P(*p*-F-Ph)$_3$, and P(*p*-CF_3-Ph)$_3$ in DMSO-d_6, showing pseudo-first order dependence with respect to phosphine concentration.

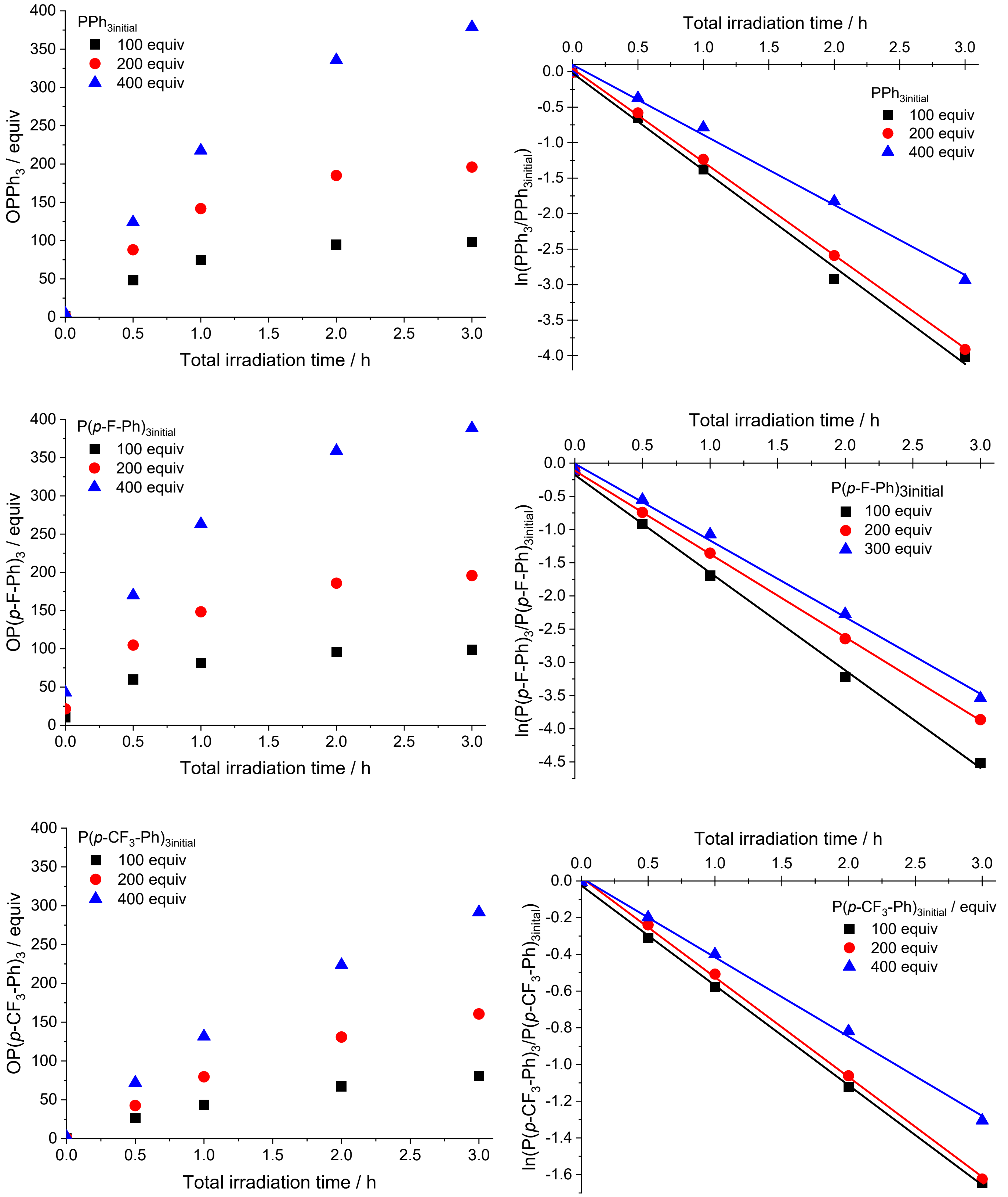

Figure S 50. Kinetics of reactions between $\mathbf{7^{CF3}}$ and PPh_3, P(*p*-F-Ph)$_3$, and P(*p*-CF_3-Ph)$_3$ in DMSO-d_6, showing pseudo-first order dependence with respect to phosphine concentration.

Computational calculations

Geometry optimization of complexes 1^{H} and $3^{C(O)OMe}$

Table S 5. Cartesian coordinates for the optimised geometry of S_0 state of complex $\mathbf{1^{H}}$.

Atom number	Atom type	X	Y	Z
1	C	4.1262420	0.4812440	-0.3183050
2	C	2.7202130	0.3785320	-0.4115770
3	C	1.8882060	1.2662380	0.3382690
4	C	2.4791300	2.2191410	1.1986730
5	C	3.8767130	2.3081080	1.2907540
6	C	4.6951740	1.4440750	0.5299130
7	H	4.7506710	-0.2074410	-0.9101490
8	H	1.8500570	2.8908750	1.8053060
9	H	5.7932520	1.5171910	0.6082120
10	N	0.5151180	1.0625170	0.1084100
11	C	-0.4240030	1.8703700	0.5300710
12	C	-1.8452730	1.6853090	0.3192160
13	C	-2.7219130	2.7285120	0.7350900
14	C	-2.4144570	0.4969650	-0.2549790
15	C	-4.1065850	2.6268850	0.5825050
16	H	-2.2807180	3.6359330	1.1830360
17	C	-3.8204850	0.4047840	-0.3977220
18	C	-4.6528150	1.4551840	0.0118510
19	H	-4.2371120	-0.5164330	-0.8361700
20	H	-5.7450470	1.3593430	-0.1127250
21	O	2.1400280	-0.5495110	-1.1879890
22	O	-1.6739960	-0.5560390	-0.6236210
23	Mo	0.1621320	-0.7127060	-1.3943730
24	O	-0.0963230	0.4623390	-2.6519400
25	H	-4.7641810	3.4503280	0.9052270
26	H	4.3332140	3.0529150	1.9634870
27	O	0.1371830	-2.2609030	-2.1903850
28	O	0.4466840	-1.8431710	0.5828030
29	S	-0.4920510	-1.6390580	1.8308600
30	C	-1.5535070	-3.1222500	1.8502420
31	H	-0.9131090	-4.0265860	1.7798140
32	H	-2.2182690	-3.0354290	0.9664690
33	H	-2.1543410	-3.1177890	2.7850490
34	C	0.6046050	-2.0408470	3.2267130
35	H	0.0036890	-2.0344980	4.1612560
36	H	1.3723470	-1.2406600	3.2616960
37	H	1.0734940	-3.0314970	3.0491880
38	H	-0.1425770	2.7894680	1.0861150

Table S 6. Cartesian coordinates for the optimised geometry of T_1 state of complex $\mathbf{1^H}$.

Atom number	Atom type	X	Y	Z
1	C	3.8105560	0.9361720	-0.5923700
2	C	2.4345700	0.5873000	-0.6315180
3	C	1.4080790	1.5653580	-0.2369920
4	C	1.8317640	2.8790540	0.1503680
5	C	3.1857740	3.1915710	0.1568410
6	C	4.1784390	2.2246090	-0.2089640
7	H	4.5557540	0.1783940	-0.8822160
8	H	1.0936120	3.6464150	0.4285560
9	H	5.2432750	2.5100890	-0.1919500
10	N	0.1441980	1.0699440	-0.2789950
11	C	-0.9338360	1.7884060	0.1727100
12	C	-2.2757420	1.3452390	0.1789860
13	C	-3.2807450	2.2250730	0.7253110
14	C	-2.7285930	0.0363400	-0.2971360
15	C	-4.6134520	1.8553090	0.8147150
16	H	-2.9580250	3.2186390	1.0815900
17	C	-4.0972190	-0.3236520	-0.1695050
18	C	-5.0303130	0.5651890	0.3657660
19	H	-4.3956390	-1.3231490	-0.5258670
20	H	-6.0905510	0.2706040	0.4392640
21	O	2.0288320	-0.6040970	-0.9911270
22	O	-1.9061060	-0.8412810	-0.8455040
23	Mo	0.0191400	-1.0338630	-1.2298250
24	O	-0.0367470	-0.3324940	-2.8434950
25	H	-5.3530930	2.5557100	1.2370750
26	H	3.5063590	4.2062300	0.4467240
27	O	0.2625370	-2.7391390	-1.4954140
28	O	0.0204740	-1.5040350	1.0423170
29	S	1.1152900	-1.0415540	2.0702210
30	C	0.1675510	-0.7393390	3.5949680
31	H	-0.4496470	-1.6342760	3.8210580
32	H	-0.4736960	0.1433920	3.3936660
33	H	0.8806830	-0.5155060	4.4169530
34	C	1.9698900	-2.5813260	2.5447720
35	H	2.6818400	-2.3539320	3.3670760
36	H	2.5219180	-2.9200300	1.6438220
37	H	1.2143840	-3.3342890	2.8531940
38	H	-0.7473410	2.7857830	0.6112110

Table S 7. Cartesian coordinates for the optimised geometry of S_0 state of complex $\mathbf{3}^{\mathbf{C(O)OMe}}$.

Atom number	Atom type	X	Y	Z
1	C	4.1039440	0.5111510	-0.7108640
2	C	2.7816790	0.0066830	-0.7131250
3	C	1.6855440	0.8742880	-0.4107850
4	C	1.9294000	2.2221120	-0.0916430
5	C	3.2493860	2.7206590	-0.0882310
6	C	4.3280870	1.8551000	-0.4012990
7	H	4.9348510	-0.1712260	-0.9503120
8	H	1.1055880	2.9076070	0.1567150
9	H	5.3511500	2.2642580	-0.3916220
10	N	0.4326580	0.2248720	-0.4702190
11	C	-0.7020240	0.8421080	-0.2611220
12	C	-2.0172190	0.2388380	-0.2718070
13	C	-3.1315330	1.0917750	-0.0592670
14	C	-2.2480590	-1.1698670	-0.4593510
15	C	-4.4445240	0.5987000	-0.0337640
16	H	-2.9548070	2.1690890	0.0889580
17	C	-3.5825150	-1.6587840	-0.4268720
18	C	-4.6550710	-0.7929340	-0.2207890
19	H	-3.7399120	-2.7398350	-0.5684380
20	H	-5.6873370	-1.1787280	-0.1973810
21	O	2.5270530	-1.2741590	-0.9814180
22	O	-1.2708230	-2.0417430	-0.6434470
23	C	3.5589440	4.1437880	0.2363460
24	C	-5.6325790	1.4750660	0.1863490
25	O	-5.2893480	2.7764350	0.3488940
26	O	2.4433500	4.8641530	0.5176420
27	O	4.6865050	4.6246370	0.2516760
28	O	-6.7907190	1.0757870	0.2191220
29	Mo	0.6641550	-2.0058960	-1.1086270
30	O	0.4170130	-1.6443460	-2.7916090
31	O	1.0823930	-3.6962350	-1.0265070
32	O	0.8464050	-1.9904920	1.1785760
33	S	0.9102280	-3.3646390	1.9563020
34	C	0.5420780	-2.8612690	3.6651780
35	H	1.2271830	-2.0391740	3.9609500
36	H	0.6565870	-3.7464630	4.3268260
37	H	-0.5129830	-2.5175470	3.6681420
38	C	2.6901360	-3.7293810	2.1263160
39	H	3.2059510	-2.8430950	2.5520920
40	H	3.0558390	-3.9510510	1.1029530
41	H	2.8066450	-4.6230540	2.7767560
42	C	2.6499780	6.2501140	0.8404690
43	H	3.1275280	6.7844580	-0.0096050
44	H	3.2950640	6.3519910	1.7402430
45	H	1.6428390	6.6662620	1.0395600
46	C	-6.3741210	3.6952580	0.5690060
47	H	-7.0767810	3.6863140	-0.2924360
48	H	-5.9058180	4.6935720	0.6738780
49	H	-6.9308660	3.4304020	1.4941440
50	H	-0.6913030	1.9312580	-0.0502990

Table S 8. Cartesian coordinates for the optimised geometry of T_1 state of complex $\mathbf{3}^{\mathbf{C(O)OMe}}$.

Atom number	Atom type	X	Y	Z
1	C	4.26887	0.048578	-0.33605
2	C	2.910359	-0.33387	-0.51458
3	C	1.836754	0.665933	-0.38635
4	C	2.195897	2.021982	-0.11011
5	C	3.53931	2.367355	0.042741
6	C	4.57518	1.375552	-0.06689
7	H	5.050501	-0.72177	-0.42766
8	H	1.430519	2.805497	-0.02369
9	H	5.619212	1.700624	0.064358
10	N	0.589964	0.150044	-0.54237
11	C	-0.5343	0.912917	-0.34991
12	C	-1.86749	0.449513	-0.42076
13	C	-2.92203	1.392305	-0.16527
14	C	-2.26074	-0.93406	-0.69667
15	C	-4.26375	1.019678	-0.1588
16	H	-2.64945	2.440632	0.034709
17	C	-3.63573	-1.29417	-0.65798
18	C	-4.62149	-0.34651	-0.40492
19	H	-3.89234	-2.34762	-0.85461
20	H	-5.68665	-0.626	-0.39261
21	O	2.563973	-1.55829	-0.78251
22	O	-1.37973	-1.87159	-0.98019
23	C	3.966412	3.775646	0.325146
24	C	-5.36532	1.994888	0.102788
25	O	-4.90821	3.252478	0.315397
26	O	2.919529	4.627291	0.399252
27	O	5.132687	4.119964	0.472442
28	O	-6.55485	1.699959	0.124479
29	Mo	0.582579	-2.07914	-1.10038
30	O	0.691067	-1.76572	-2.82764
31	O	0.849586	-3.7947	-0.91986
32	O	0.453453	-1.98488	1.197783
33	S	0.049767	-3.30916	1.962091
34	C	-0.5158	-2.67831	3.572402
35	H	0.264999	-2.01417	3.999246
36	H	-0.7242	-3.54367	4.237703
37	H	-1.45093	-2.11425	3.375784
38	C	1.635815	-4.05295	2.470402
39	H	2.24778	-3.28533	2.989413
40	H	2.125103	-4.39003	1.53387
41	H	1.423988	-4.92244	3.129481
42	C	3.23936	6.005791	0.665932
43	H	3.891161	6.416541	-0.13523
44	H	3.758239	6.10341	1.644019
45	H	2.269446	6.539724	0.687459
46	C	-5.90819	4.253697	0.574813
47	H	-6.60604	4.34312	-0.28599
48	H	-5.35367	5.201282	0.721513
49	H	-6.4895	4.000145	1.487923
50	H	-0.40125	1.974414	-0.07542

Table S 9. Atomic contributions towards frontier molecular orbital composition of S_0 state of complex **1^H**.

Orbital	Atom number	Atom type	Contribution[a]
91 (HOMO)	3	C	13.9%
	2	C	12.6%
	5	C	12.3%
	21	O	10.1%
	6	C	8.1%
	12	C	5.7%
	10	N	5.2%
	14	C	5.0%
92 (LUMO)	23	Mo	24.8%
	11	C	17.2%
	24	O	10.6%
	18	C	7.2%
	10	N	7.1%
	14	C	5.9%

[a] Atomic contributions of less than 5% were ignored.

Table S 10. Atomic contributions towards frontier molecular orbital composition of T_1 state of complex **1^H**.

Orbital	Atom number	Atom type	Contribution[a]
92 ($SOMO_1\alpha$)	11	C	21.8%
	23	Mo	17.3%
	24	O	9.5%
	18	C	8.7%
	14	C	7.9%
	10	N	7.6%
	13	C	5.8%
93 ($SOMO_2\alpha$)	23	Mo	45.7%
	24	O	11.5%
	11	C	6.6%
	22	O	5.9%
90 ($SOMO_M\beta$)	15	C	22.0%
	17	C	14.5%
	22	O	12.0%
	14	C	7.9%
	13	C	7.8%
	12	C	7.1%
	24	O	5.7%
91 ($SOMO_2\beta$)	3	C	14.3%
	2	C	11.7%
	6	C	9.7%
	5	C	9.7%
	10	N	9.5%
	21	O	9.0%
	11	C	6.0%

[a] Atomic contributions of less than 5% were ignored.

Table S 11. Atomic contributions towards frontier molecular orbital composition of S_0 state of complex **3**$^{\mathbf{C(O)OMe}}$.

Orbital	Atom number	Atom type	Contribution[a]
121 (HOMO)	3	C	13.7%
	5	C	11.5%
	2	C	10.6%
	21	O	9.9%
	6	C	6.3%
	12	C	6.3%
	10	N	5.7%
122 (LUMO)	29	Mo	26.0%
	11	C	16.3%
	30	O	11.9%
	18	C	7.7%
	14	C	6.9%
	10	N	6.7%

[a] Atomic contributions of less than 5% were ignored.

Table S 12. Atomic contributions towards frontier molecular orbital composition of T_1 state of complex **3**$^{\mathbf{C(O)OMe}}$.

Orbital	Atom number	Atom type	Contribution[a]
122 ($SOMO_1\alpha$)	11	C	22/5%
	29	Mo	16.1%
	30	O	9.1%
	18	C	9.0%
	14	C	8.3%
	10	N	7.9%
	13	C	5.4%
123 ($SOMO_2\alpha$)	29	Mo	44.1%
	30	O	11.1%
	11	C	6.4%
120 ($SOMO_1\beta$)	15	C	20.0%
	17	C	14.3%
	22	O	12.1%
	12	C	7.0%
	14	C	6.6%
	13	C	5.6%
	30	O	5.5%
121 ($SOMO_2\beta$)	3	C	14.5%
	10	N	10.5%
	2	C	9.7%
	5	C	8.9%
	21	O	8.7%
	6	C	7.9%
	11	C	6.1%

[a] Atomic contributions of less than 5% were ignored.

Time-dependent density functional theory (TD-DFT) calculations of complexes 1^{H} and $3^{C(O)OMe}$.

The simulated UV-Vis spectra are given below for each hybrid functional used; all spectra show features that correspond well with the experimental UV-Vis spectra of complexes $\mathbf{1^{H}}$ and $\mathbf{3^{C(O)OMe}}$. The best match with the experimental spectra was obtained employing PBE0 hybrid functional, which was used for further comparison. The calculated excited state parameters obtained from calculations with PBE0 hybrid functional are summarised in Tables S13 ($\mathbf{1^{H}}$) and S14 ($\mathbf{3^{C(O)OMe}}$).

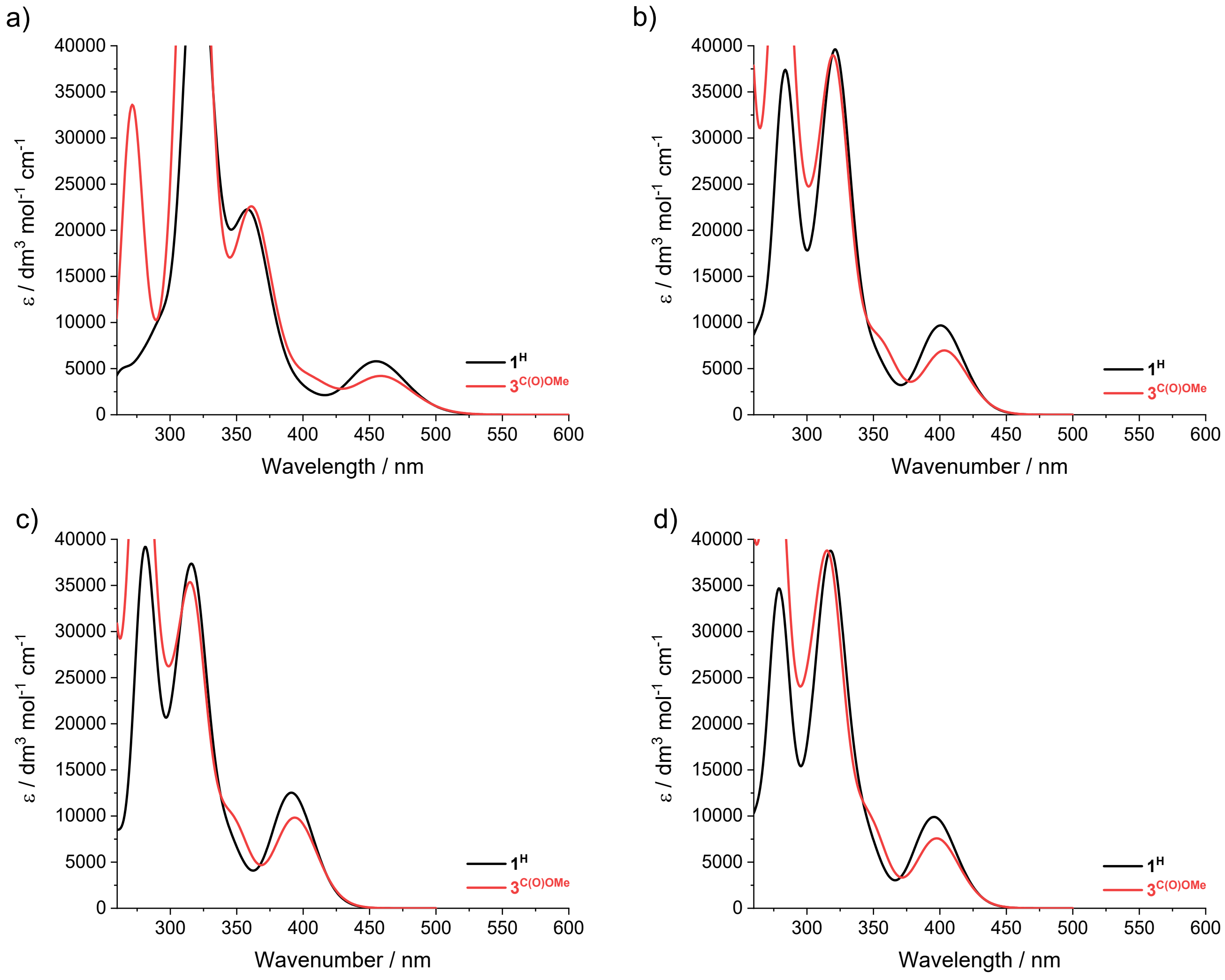

Figure S 51: TD-DFT simulated UV-Vis spectra for $\mathbf{1^{H}}$ (black lines) and $\mathbf{3^{C(O)OMe}}$ (red lines) using DEF2TZVPP/SCRF(DMSO) parameters and functionals: a) PBE0; b) CAM-B3LYP; c) M0962X; d) wB97XD.

Table S 13: TD-DFT calculated singlet excited states of **1^H** (PBE0/DEF2TZVPP/SCRF(DMSO).

Excited state	λ / nm	$E_{excitation}$ / eV	Oscillator strength	Spin state	Orbital transition	Composition[a]
S_1	455.3	2.7232	0.0643	Singlet	HOMO→LUMO	90.4%
					HOMO→$LUMO_{+1}$	4.4%
S_2	398.1	3.1144	0.0251	Singlet	HOMO→$LUMO_{+1}$	68.0%
					HOMO→$LUMO_{+2}$	28.9%
S_3	368.3	3.3663	0.0597	Singlet	$HOMO_{-1}$→LUMO	79.1%
					HOMO→LUMO	2.2%
					HOMO→$LUMO_{+2}$	7.4%
					HOMO→$LUMO_{+3}$	4.9%
S_4	359.1	3.4526	0.1709	Singlet	$HOMO_{-2}$→LUMO	10.6%
					$HOMO_{-1}$→LUMO	4.0%
					HOMO→$LUMO_{+1}$	15.4%
					HOMO→$LUMO_{+2}$	45.9%
					HOMO→$LUMO_{+3}$	18.9%
S_5	342.3	3.6216	0.0602	Singlet	$HOMO_{-2}$→LUMO	7.5%
					$HOMO_{-1}$→LUMO	8.9%
					$HOMO_{-1}$→$LUMO_{+1}$	3.6%
					HOMO→$LUMO_{+1}$	2.2%
					HOMO→$LUMO_{+2}$	4.3%
					HOMO→$LUMO_{+3}$	69.0%
S_6	332.5	3.7286	0.0058	Singlet	$HOMO_{-1}$→$LUMO_{+1}$	76.1%
					$HOMO_{-1}$→$LUMO_{+2}$	16.5%
					HOMO→$LUMO_{+3}$	2.9%
S_7	320.5	3.869	0.5892	Singlet	$HOMO_{-2}$→LUMO	74.0%
					HOMO→$LUMO_{+1}$	5.5%
					HOMO→$LUMO_{+2}$	7.5%
S_8	310.3	3.9958	0.0133	Singlet	$HOMO_{-5}$→LUMO	33.8%
					$HOMO_{-5}$→$LUMO_{+1}$	3.6%
					$HOMO_{-5}$→$LUMO_{+2}$	10.4%
					$HOMO_{-4}$→LUMO	4.3%
					$HOMO_{-1}$→$LUMO_{+1}$	4.6%
					$HOMO_{-1}$→$LUMO_{+2}$	32.3%
S_9	308.8	4.0154	0.0048	Singlet	$HOMO_{-5}$→LUMO	26.5%
					$HOMO_{-5}$→$LUMO_{+1}$	3.6%
					$HOMO_{-5}$→$LUMO_{+2}$	7.4%
					$HOMO_{-1}$→$LUMO_{+1}$	9.6%
					$HOMO_{-1}$→$LUMO_{+2}$	41.5%
S_{10}	298.7	4.1513	0.0096	Singlet	$HOMO_{-4}$→LUMO	4.3%
					$HOMO_{-3}$→LUMO	84.4%
					$HOMO_{-3}$→$LUMO_{+1}$	3.2%
					$HOMO_{-3}$→$LUMO_{+2}$	2.5%

[a] Transition contributions of less than 2% were ignored.

Table S 14: TD-DFT calculated singlet excited states of **3**$^{C(O)OMe}$ (PBE0/DEF2TZVPP/SCRF(DMSO).

Excited state	λ / nm	$E_{excitation}$ / eV	Oscillator strength	Spin state	Orbital transition	Composition[a]
S_1	459.4	2.6986	0.046	Singlet	HOMO→LUMO	92.4%
					HOMO→$LUMO_{+1}$	3.3%
S_2	405	3.0615	0.0403	Singlet	HOMO→$LUMO_{+1}$	87.0%
					HOMO→$LUMO_{+2}$	9.1%
S_3	369.6	3.3543	0.0714	Singlet	$HOMO_{-1}$→LUMO	86.5%
					HOMO→$LUMO_{+3}$	6.3%
S_4	359.6	3.4476	0.1853	Singlet	$HOMO_{-2}$→LUMO	15.5%
					$HOMO_{-1}$→LUMO	2.1%
					HOMO→$LUMO_{+1}$	5.3%
					HOMO→$LUMO_{+2}$	72.6%
					HOMO→$LUMO_{+3}$	3.3%
S_5	342.2	3.6206	0.0014	Singlet	$HOMO_{-1}$→$LUMO_{+1}$	87.4%
					$HOMO_{-1}$→$LUMO_{+2}$	6.3%
S_6	336.1	3.6889	0.0483	Singlet	$HOMO_{-2}$→LUMO	13.7%
					$HOMO_{-1}$→LUMO	3.2%
					HOMO→$LUMO_{+3}$	72.0%
					HOMO→$LUMO_{+4}$	3.3%
S_7	317.8	3.9015	0.7947	Singlet	$HOMO_{-2}$→LUMO	64.1%
					$HOMO_{-1}$→$LUMO_{+2}$	2.1%
					HOMO→$LUMO_{+1}$	2.5%
					HOMO→$LUMO_{+2}$	12.1%
					HOMO→$LUMO_{+3}$	11.9%
S_8	311.8	3.9766	0.0045	Singlet	$HOMO_{-8}$→LUMO	2.4%
					$HOMO_{-7}$→LUMO	37.5%
					$HOMO_{-7}$→$LUMO_{+2}$	8.1%
					$HOMO_{-4}$→LUMO	2.5%
					$HOMO_{-3}$→LUMO	32.5%
S_9	308.8	4.0146	0.0574	Singlet	$HOMO_{-7}$→LUMO	6.6%
					$HOMO_{-7}$→$LUMO_{+2}$	2.1%
					$HOMO_{-5}$→LUMO	3.1%
					$HOMO_{-3}$→LUMO	33.3%
					$HOMO_{-1}$→$LUMO_{+1}$	2.5%
					$HOMO_{-1}$→$LUMO_{+2}$	38.0%
					HOMO→$LUMO_{+4}$	2.7%
S_{10}	304.1	4.0767	0.0464	Singlet	$HOMO_{-7}$→LUMO	12.5%
					$HOMO_{-7}$→$LUMO_{+1}$	2.5%
					$HOMO_{-7}$→ $LUMO_{+2}$	2.6%
					$HOMO_{-3}$→LUMO	24.0%
					$HOMO_{-1}$→$LUMO_{+1}$	3.2%
					$HOMO_{-1}$→$LUMO_{+2}$	37.1%
					$HOMO_{-1}$→$LUMO_{+3}$	3.6%

[a] Transition contributions of less than 2% were ignored.

Table S 15. Single point energy comparison between S_0, T_1, and S_1 states of complexes $\mathbf{1^H}$ and $\mathbf{3^{C(O)OMe}}$.

	Single point energy / Ha			
**State**	$\mathbf{1^H}$		$\mathbf{3^{C(O)OMe}}$	
S_0	-1477.193483		-1932.651778	
T_1	-1477.122089		-1932.578654	
S_1	-1477.093410		-1932.552606	
	**Energy differences between states**			
	**Ha**	**kJ mol^{-1}**	**Ha**	**kJ mol^{-1}**
S_0-T_1	-0.071395	-187	-0.073124	-192
S_0-S_1	-0.100073	-263	-0.099172	-260

Photocatalysis at different wavelengths

Table S 16. PPh_3 conversion after 3 hours of irradiation at specified wavelengths with catalysts **2**$^{C(O)OH}$ and **3**$^{C(O)OMe}$.

Catalyst	PPh_3 conversion / %		
	365 nm	410 nm	460 nm
**2**$^{C(O)OH}$	32	35	32
**3**$^{C(O)OMe}$	69	78	77

Steady state emission spectroscopy

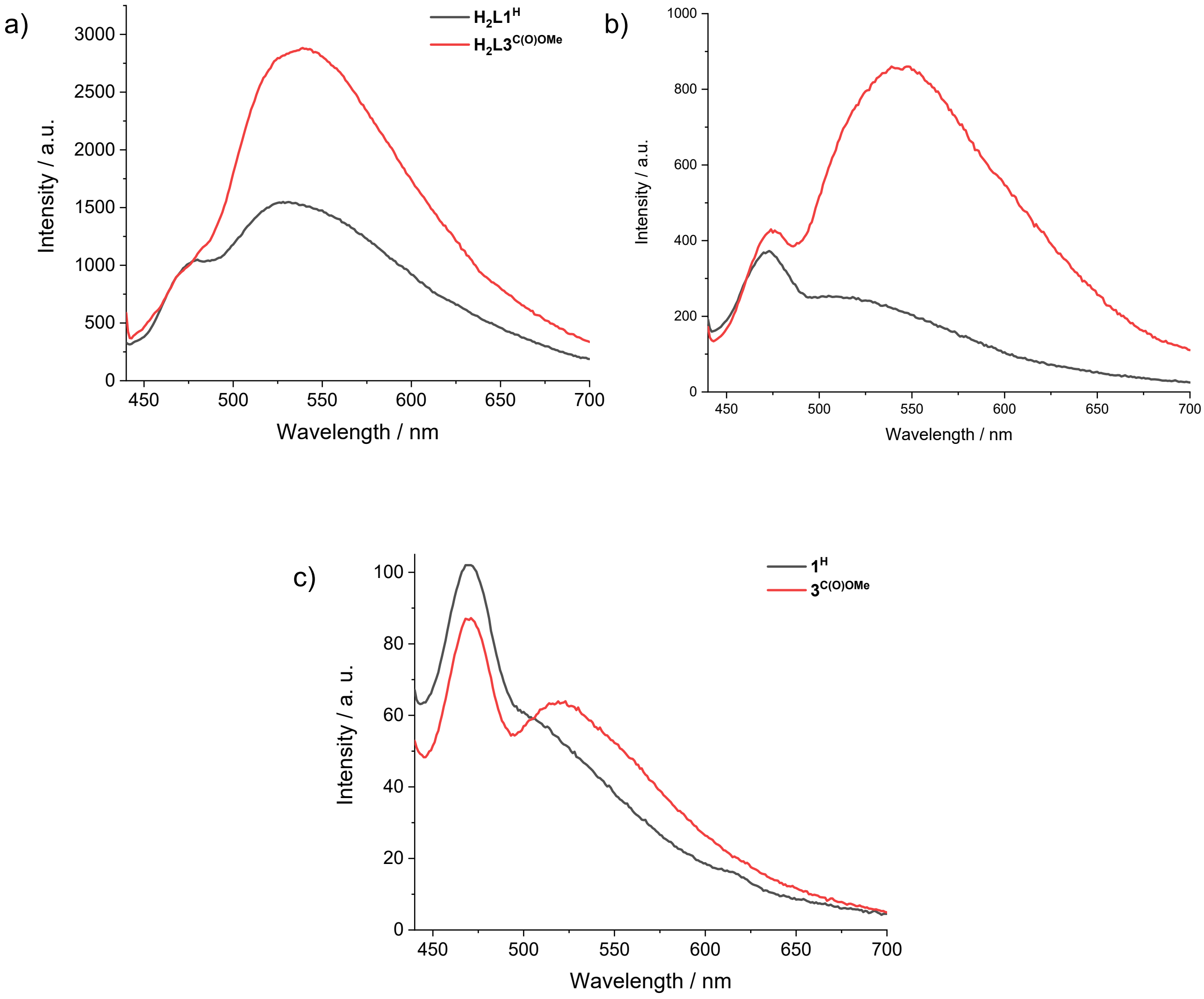

Figure S 52: Steady state emission spectrum of: a) **H_2L1^H** in acetone (0.031 mM) (black line) and **$H_2L3^{C(O)OMe}$** in acetone (0.081 mM) (red line); b) **1**^{H} in acetone (0.020 mM) (black line) and **3**$^{C(O)OMe}$ in acetone (0.018 mM) (red line); c) **1**^{H} in DMSO (0.022 mM) (black line) and **3**$^{C(O)OMe}$ in DMSO (0.015 mM) (red line).

Time-resolved photoluminescence spectroscopy

Concentrations were chosen to give an absorption of 0.1. at the excitation wavelengths λ_{ex} = 473 nm and λ_{em} = 525 nm. Samples were prepared and analysed under N_2 atmosphere. The emission decay profiles were fitted with exponential curves of the form shown below. The linear portion of the profile was identified at ca. 10 to 25 ns and fitting performed on this interval. Double exponential fitting was tested but yielded no improvement.

$$Fit = A + B_1 * \exp\left({}^{-t}/_{\tau_1} \right)$$

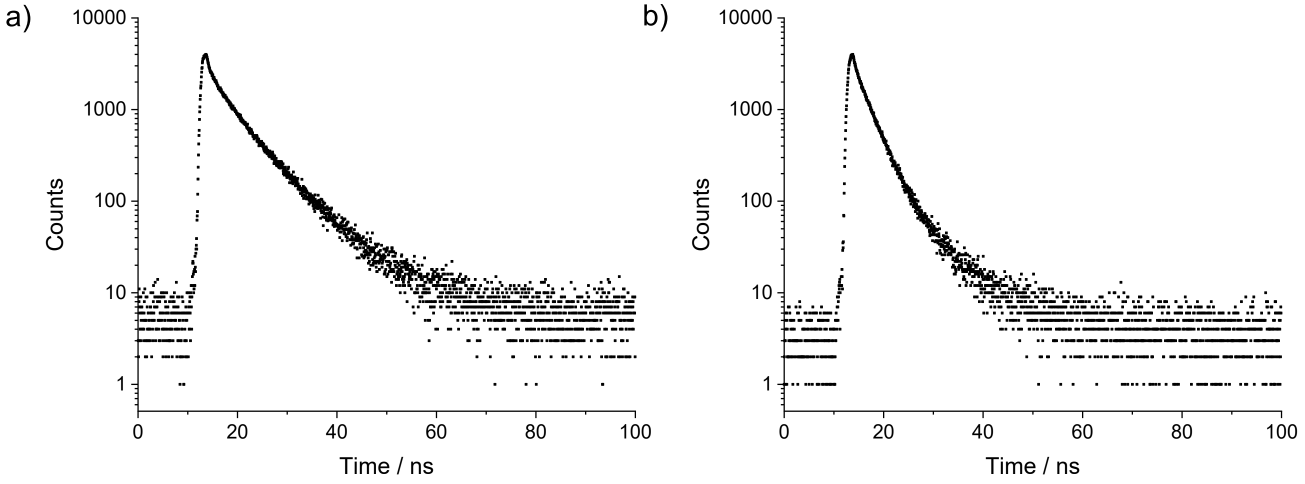

Figure S 53: Emission decay profile of: a) a 0.056 mM solution of **1^H^** in DMSO; b) a 0.013 mM solution of **3^C(O)OMe^** in DMSO.

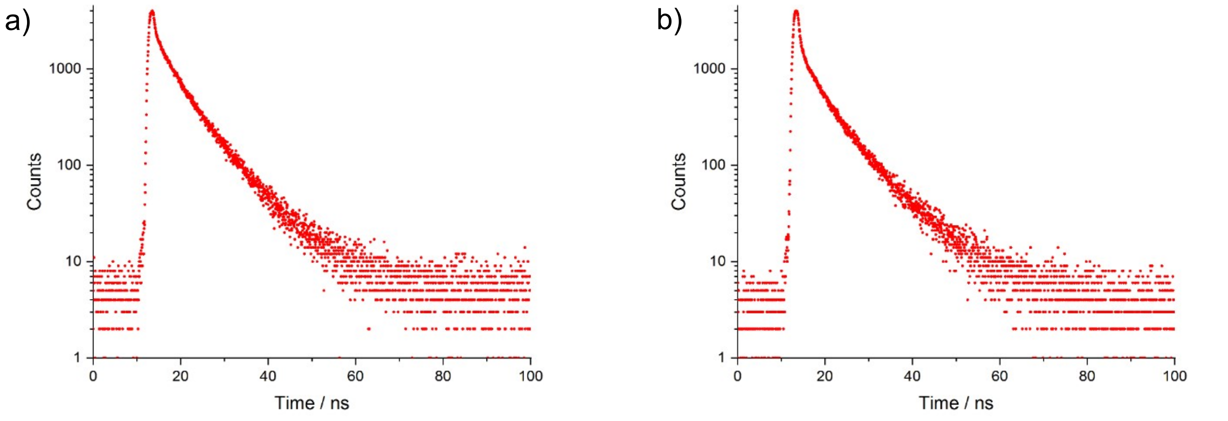

Figure S 54: Emission decay profile of: a) a 0.056 mM solution of **1^H^** in DMSO containing 300 eq of PPh_3; b) of a 0.056 mM solution of **3^C(O)OMe^** in DMSO containing 300 eq of PPh_3.

Table S 17: Excited state lifetimes of complexes **1^{H}** and **3$^{C(O)OMe}$** in DMSO.

Sample components	Excited state lifetime at 525 nm / ns
**1^{H}**	5.3 ± 0.02 (2.6)
**1^{H}** + PPh_3	5.3 ± 0.02 (2.3)
**3$^{C(O)OMe}$**	3.1 ± 0.01 (1.8)
**3$^{C(O)OMe}$** + PPh_3	5.2 ± 0.03 (1.7)

λ_{ex} = 472 nm, in DMSO, under N_2. χ^2 values (goodness of fit) are given in brackets

Ground state interactions between $3^{C(O)OMe}$ or 7^{CF3} and PPh_3 – ^{1}H and ^{31}P NMR spectra

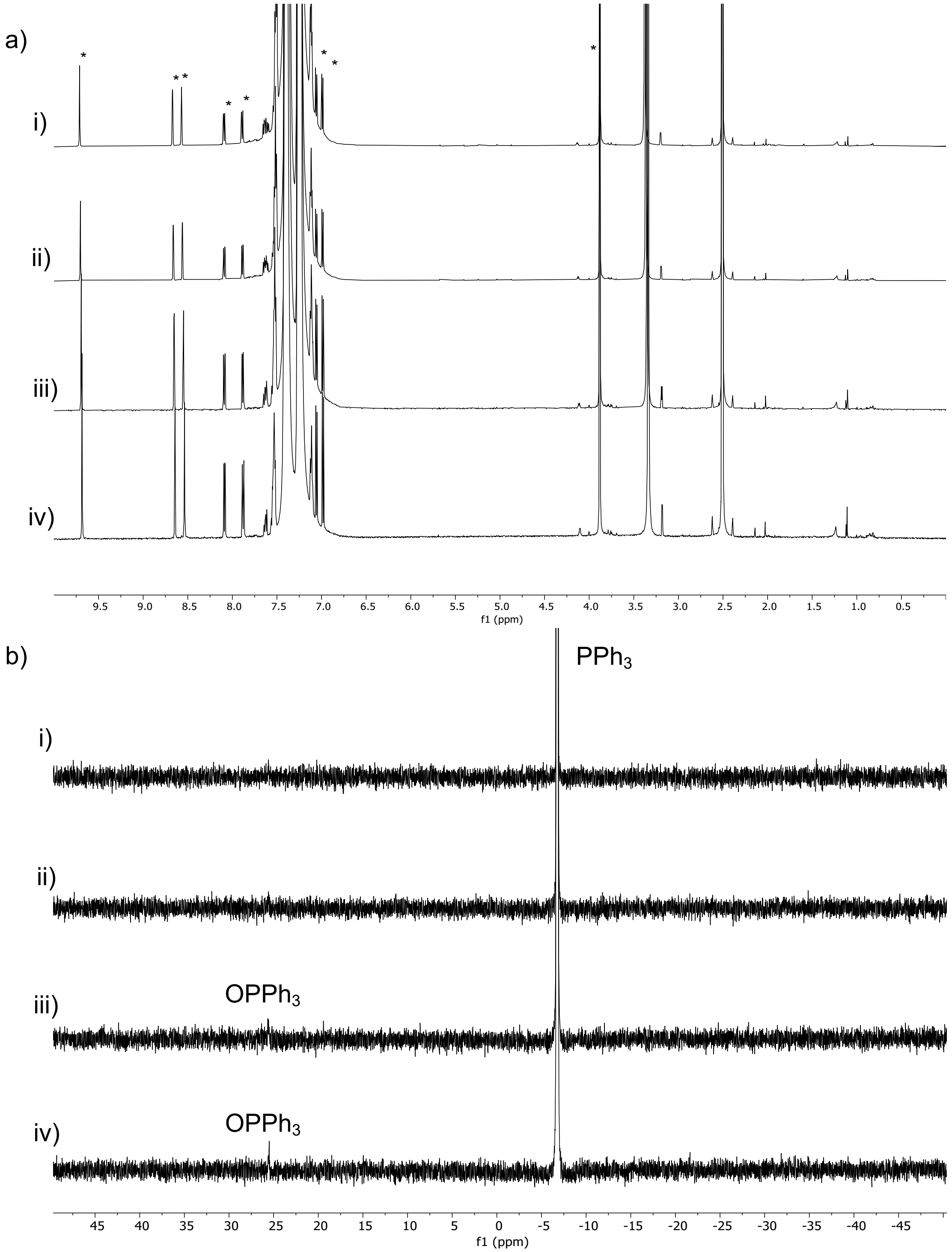

Figure S 55: a) ^{1}H (600 MHz, DMSO-d_6, 25°C) and b) zoomed-in $^{31}P\{^{1}H\}$ NMR spectra (243 MHz, DMSO-d_6, 25°C) of **$3^{C(O)OMe}$** (1 mM in DMSO-d_6) with varying concentrations of PPh_3: i) 100 mM; ii) 200 mM; iii) 300 mM; iv) 400 mM. The samples were not irradiated before recording the spectra. In the ^{1}H NMR spectrum catalyst signals are indicated with an asterix. The additional low intensity signal observed in b) (iii)-(iv) spectra is due to $OPPh_3$ impurity present in the commercial PPh_3 used during sample preparation. No resonances were detected for species resulting from ground state interactions between **$3^{C(O)OMe}$** and PPh_3.

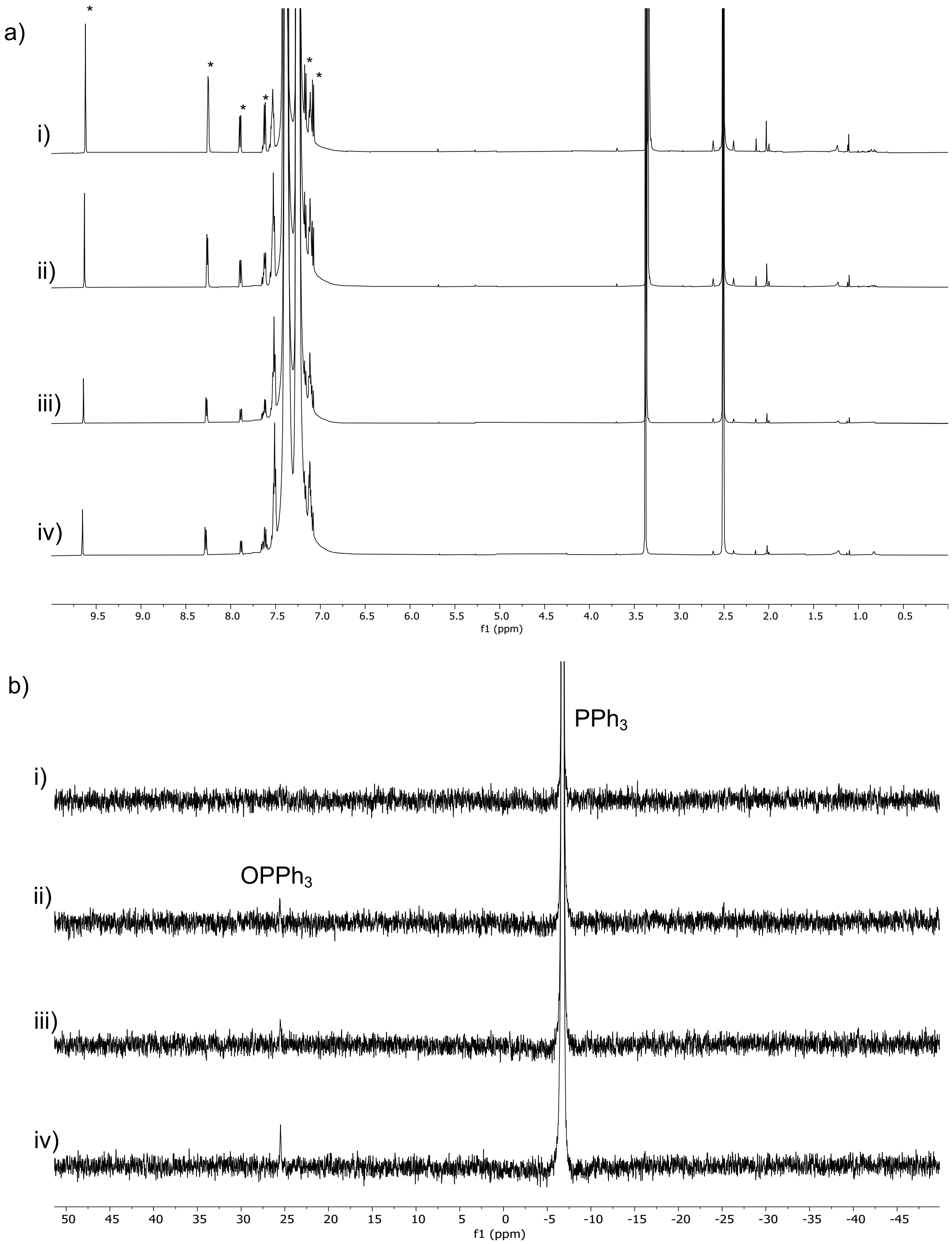

Figure S 56: a) ^{1}H (600 MHz, DMSO-d_6, 25°C) and b) zoomed-in $^{31}P\{^{1}H\}$ NMR spectra (243 MHz, DMSO-d_6, 25°C) of $\mathbf{7^{CF3}}$ (1 mM in DMSO-d_6) with varying concentrations of PPh_3: i) 100 mM; ii) 200 mM; iii) 300 mM; iv) 400 mM. The samples were not irradiated before recording the spectra. In the ^{1}H NMR spectrum catalyst signals are indicated with an asterix. The additional low intensity signal observed in b) (ii)-(iv) spectra is due to $OPPh_3$ impurity present in the commercial PPh_3 used during sample preparation. No resonances were detected for species resulting from ground state interactions between $\mathbf{7^{CF3}}$ and PPh_3

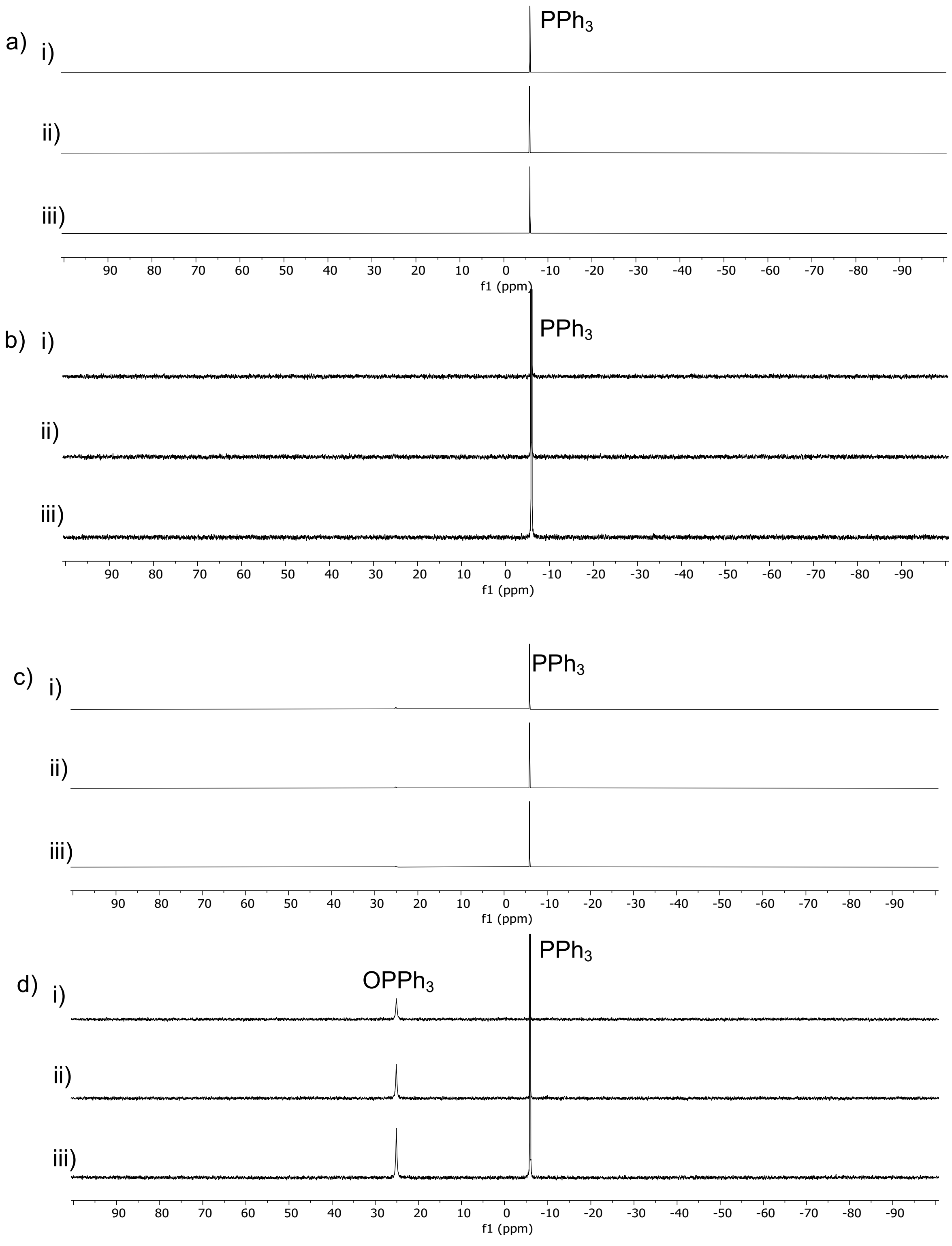

Figure S 57: $^{31}P\{^{1}H\}$ NMR spectra (243 MHz, DMF-d_7, 25°C) of non-catalytic reactions between $\mathbf{3}^{\mathbf{C(O)OMe}}$ (50 mM) and different concentrations of PPh_3 in DMF-d_7 (1 mL); i) 50 mM PPh_3 (1:1 ($\mathbf{3}^{\mathbf{C(O)OMe}}$:$PPh_3$) molar ratio); ii) 100 mM PPh_3 (1:2 ($\mathbf{3}^{\mathbf{C(O)OMe}}$:$PPh_3$) molar ratio); ii) 250 mM PPh_3 (1:5 ($\mathbf{3}^{\mathbf{C(O)OMe}}$:$PPh_3$) molar ratio); spectra a)-b) were recorded before irradiation; spectra c)-d) were recorded after 20 h irradiation at 410 nm. The spectra show prominent peaks for PPh_3 and $OPPh_3$ species; no resonances for catalyst-coordinated PPh_3 or $OPPh_3$ species are observed.

Non-catalytic reactions – Mo(O)-intermediate generation

Detection by ^{1}H NMR spectroscopy

Reactions were prepared in a glove box under N_2 atmosphere. DMF-d_7 was used as received, without further purification. Solutions of the samples in DMF-d_7 were prepared to contain final concentrations of 50 mM of **3**$^{\mathbf{C(O)OMe}}$ and 250 mM of PPh_3, and 1 mL portions were then transferred using a syringe into Youngs NMR tubes. The NMR tubes were inserted into the photoreactor (Fig. S1), and irradiated (λ = 410 nm) for selected time intervals. Colour change from yellow to green (Fig. S54) was apparent after 5 mins irradiation. Samples were monitored by ^{1}H and ^{31}P NMR spectroscopy.

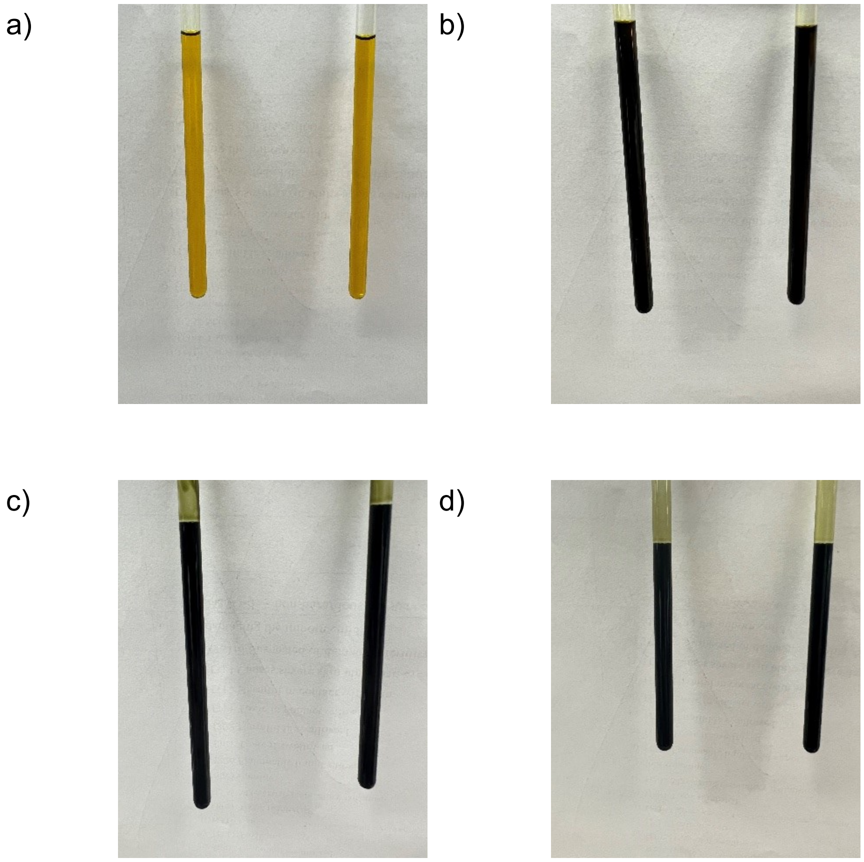

Figure S 58. Images of non-catalytic rections, containing **3**$^{\mathbf{C(O)OMe}}$ (50 mM), PPh_3 (250 mM) in DMF-d_7 (1 mL) under N_2; a) samples as prepared; b) samples after 21 h of continuous irradiation at 410 nm; c) samples after 21 h continuous irradiation and addition of 10 equivalents of DMSO-d_6; d) samples after 21 h continuous irradiation and addition of 10 equivalents of DMSO-d_6, followed by 2 h in the dark at 45°C (sample on the left hand side) or 2 h further irradiation at 410 nm (sample on the right hand side).

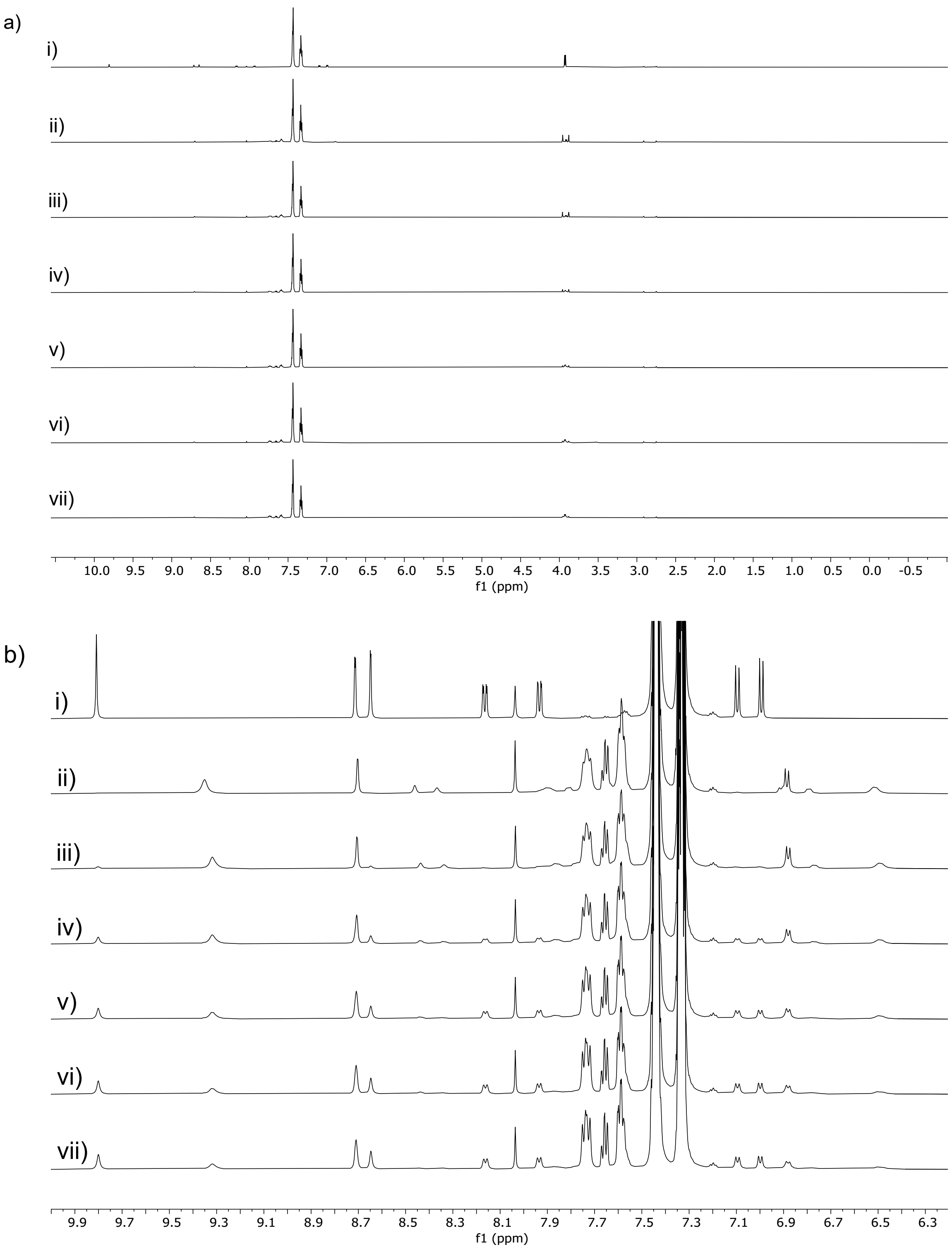

Figure S 59: ^{1}H NMR spectra (600 MHz, DMF-d_7, 25°C) of non-catalytic reactions, i.e. **$3^{C(O)OMe}$** (50 mM), PPh_3 (250 mM), in DMF-d_7 (1 mL) under N_2; a) full spectra; b) aromatic region; i) sample as prepared; ii) sample after 21 h of continuous irradiation at 410 nm; iii) sample after 21 h continuous irradiation and addition of 10 eq. of DMSO-d_6; iv) sample after 21 h continuous irradiation and addition of 10 eq. of DMSO-d_6, followed by 30 min irradiation at 410 nm; v) sample after 21 h continuous irradiation and addition of 10 eq. of DMSO-d_6, followed by 60 min irradiation at 410 nm; vi) sample after 21 h continuous irradiation and addition of 10 eq. of DMSO-d_6, followed by 90 min irradiation at 410 nm; vii) sample after 21 h continuous irradiation and addition of 10 eq. of DMSO-d_6, followed by 120 min irradiation at 410 nm.

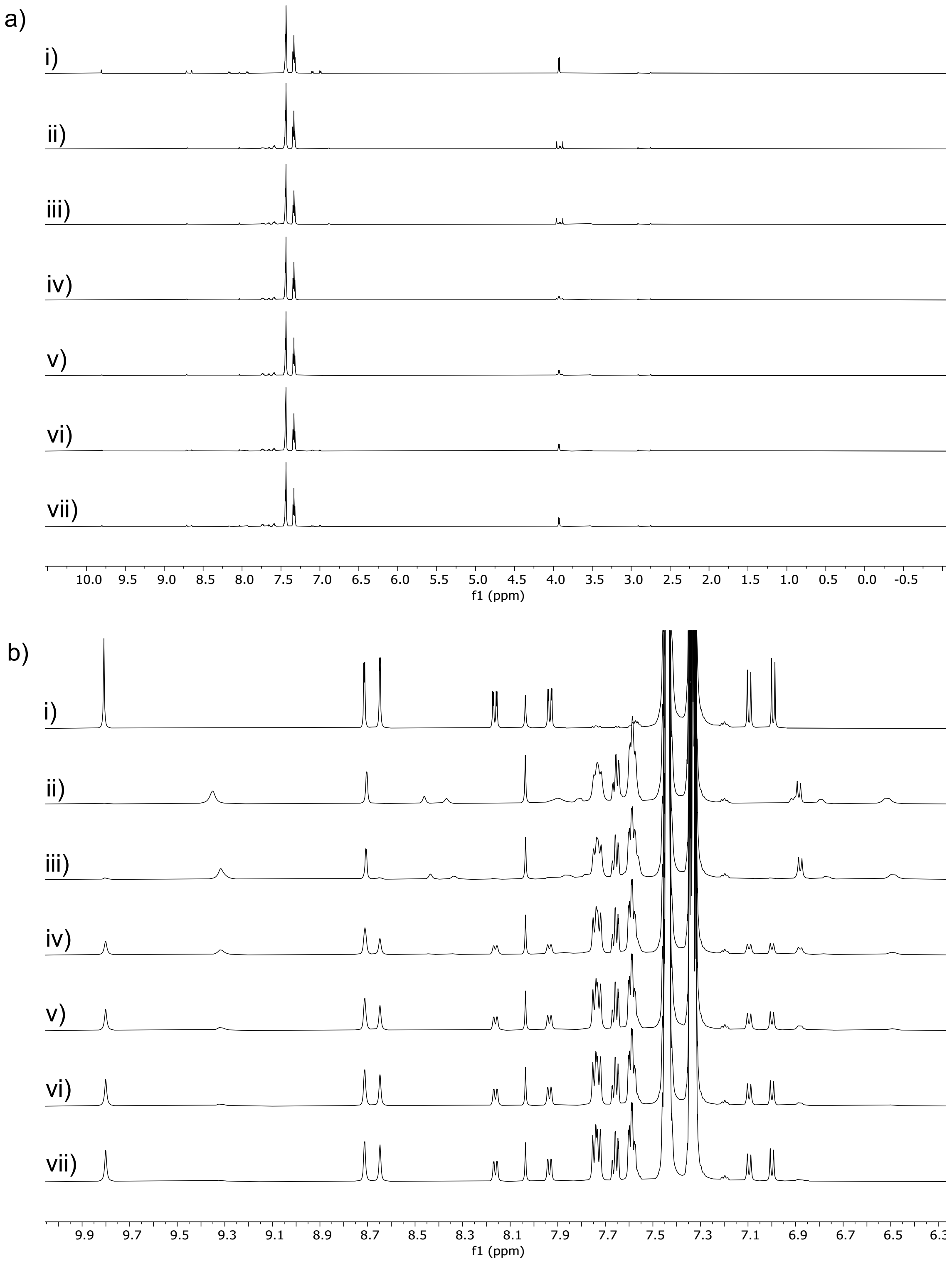

Figure S 60: ^{1}H NMR spectra (600 MHz, DMF-d_7, 25°C) of non-catalytic reactions, i.e. **3**$^{\mathbf{C(O)OMe}}$ (50 mM), PPh_3 (250 mM), in DMF-d_7 (1 mL) under N_2; a) full spectra; b) aromatic region; i) sample as prepared; ii) sample after 21 h of continuous irradiation at 410 nm; iii) sample after 21 h continuous irradiation and addition of 10 eq. of DMSO-d_6; iv) sample after 21 h continuous irradiation, addition of 10 eq. of DMSO-d_6, followed by additional 30 min in the dark at 45°C; v) sample after 21 h continuous irradiation, addition of 10 eq. of DMSO-d_6, followed by additional 60 min in the dark at 45°C; vi) sample after 21 h continuous irradiation, addition of 10 eq. of DMSO-d_6, followed by additional 90 min in the dark at 45°C; vii) sample after 21 h continuous irradiation, addition of 10 eq. of DMSO-d_6, followed by additional 120 min in the dark at 45°C.

In operando solution FTIR spectroscopy.

During 4 hour irradiation at 410 nm, the sample containing complex **3**$^{\mathbf{C(O)OMe}}$ (50 mmol), PPh_3 (500 mmol), and DMSO-d_6 (2.5 M) in DMF (50 µL) showed bleaching of the initial *cis*-$Mo(O)_2$ antisymmetric (911 cm^{-1}) and symmetric (933 cm^{-1}) stretches of **3**$^{\mathbf{C(O)OMe}}$ and growth of a band at 948 cm^{-1} was attributed to monomeric Mo(O) intermediate after OAT to PPh_3 (Fig. S61). Concomitant growth of a $OPPh_3$ band at 1196 cm^{-1} was also observed (Fig. S64). After 4 hour continuous irradiation, the sample was left in the dark and monitored using FTIR for 20 h where regeneration of the initial *cis*-$Mo(O)_2$ bands were observed, accompanied by the bleaching of the Mo(O) intermediate band (Fig. S65). The $OPPh_3$ band did not change intensity significantly (Fig. S66).

Analogous sample containing complex **1**$^{\mathbf{H}}$ did not show the changes observed for complex **3**$^{\mathbf{C(O)OMe}}$ with no *cis*-$Mo(O)_2$ antisymmetric (910 cm^{-1}) and symmetric (932 cm^{-1}) band bleaching (Fig. S67), or $OPPh_3$ band growth observed (Fig. S68).

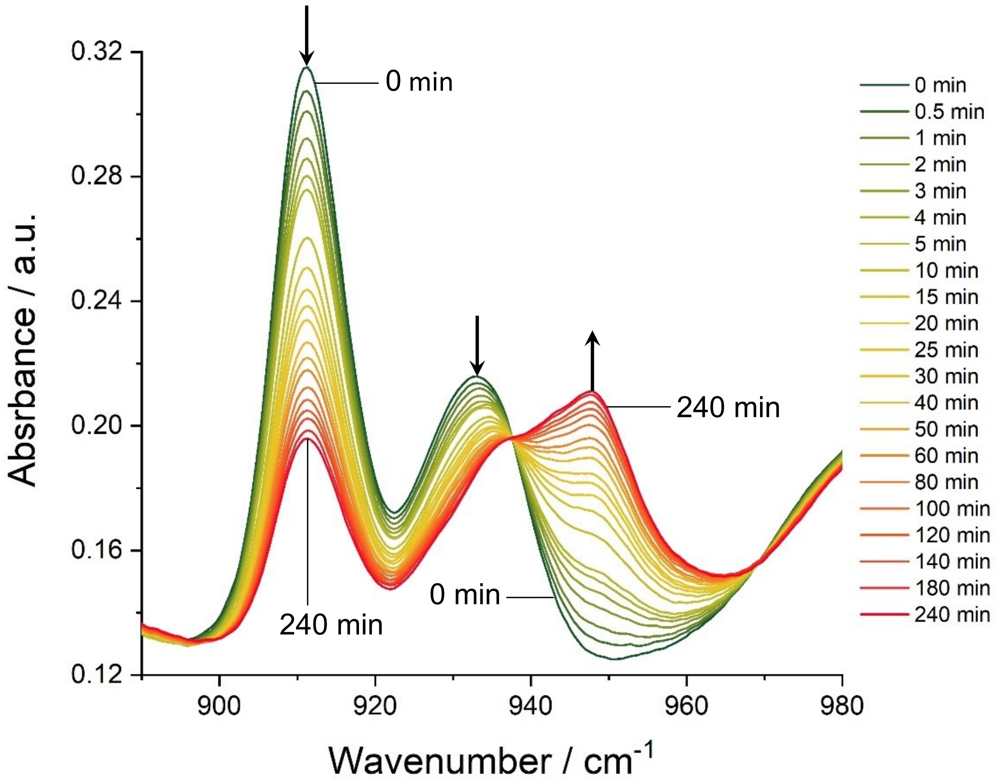

Figure S 61. FTIR spectra (DMF, 25°C, 890-980 cm^{-1} region) of non-catalytic sample of **3**$^{\mathbf{C(O)OMe}}$ (50 mM), PPh_3 (500 mM), and DMSO-d_6 (2.5 M) at selected timepoints during 4 hour irradiation (410 nm).

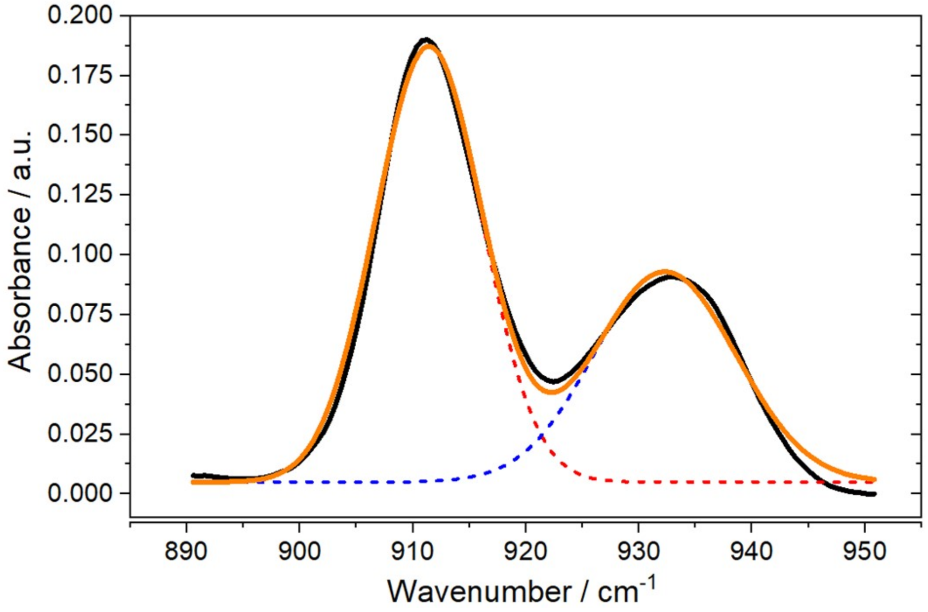

Figure S 62. Gaussian curve fitting for the baseline-corrected FTIR spectrum (DMF, 25°C, 890-950 cm^{-1} region) of non-catalytic sample of **3$^{C(O)OMe}$** (50 mM), PPh_3 (500 mM), and DMSO-d_6 (2.5 M) before irradiation (0 min; black solid line). Red dashed line – single Gaussian curve fitted for the peak at 911 cm^{-1}; blue dashed line – single Gaussian curve fitted for the peak at 933 cm^{-1}; orange solid line – cumulative Gaussian fit for the selected reegion (890-950 cm^{-1}).

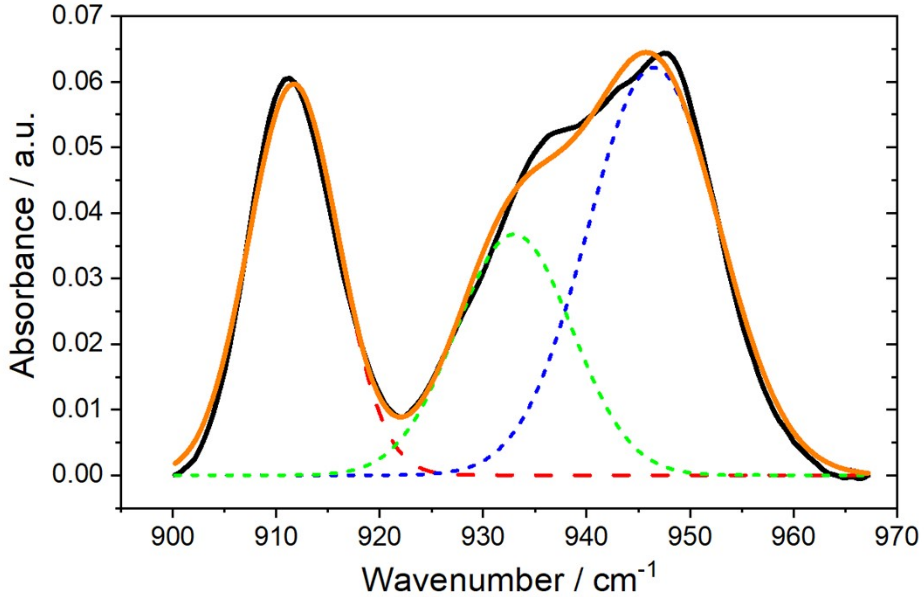

Figure S 63. Gaussian curve fitting for the baseline-corrected FTIR spectrum (DMF, 25°C, 900-968 cm^{-1} region) of non-catalytic sample of **3$^{C(O)OMe}$** (50 mM), PPh_3 (500 mM), and DMSO-d_6 (2.5 M) after 240 min irradiation (410 nm; black solid line). Red dashed line – single Gaussian curve fitted for the peak at 911 cm^{-1}; green dashed line – single Gaussian curve fitted for the peak at 933 cm^{-1}; blue dashed line – single Gaussian curve fitted for the peak at 948 cm^{-1}; orange solid line – cumulative Gaussian fit for the selected region (900-968 cm^{-1}).

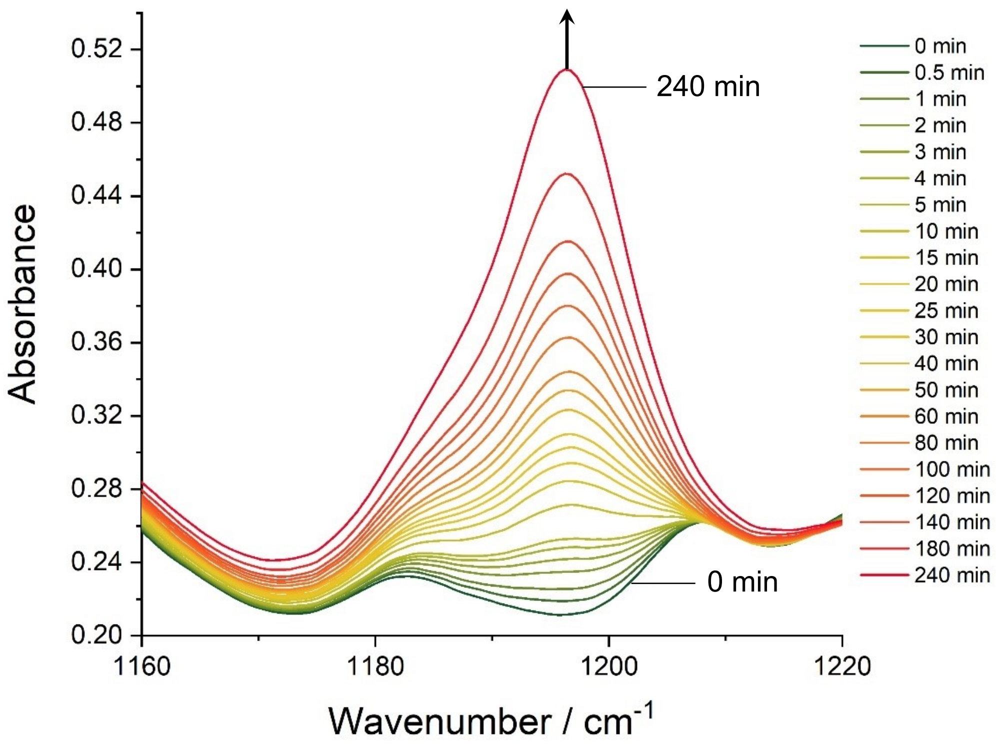

Figure S64. FTIR spectra (DMF, 25°C, 1160-1220 cm^{-1} region) of non-catalytic sample of **3$^{C(O)OMe}$** (50 mM), PPh_3 (500 mM), and DMSO-d$_6$ (2.5 M) at selected timepoints during 4 hour irradiation (410 nm).

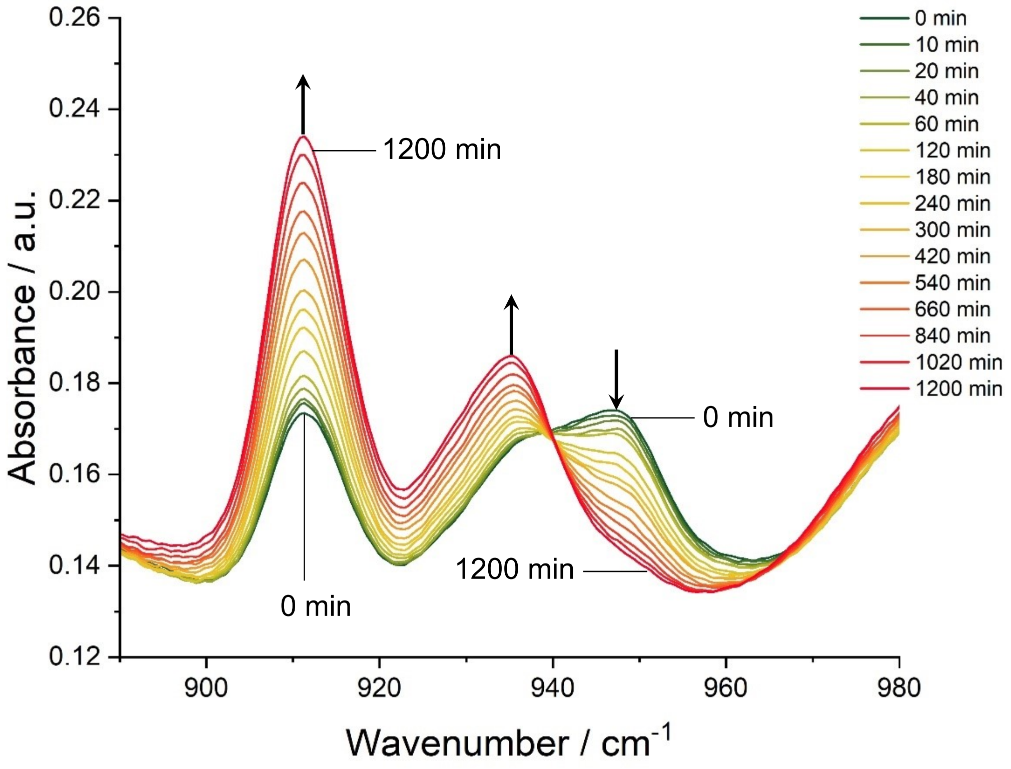

Figure S 65. FTIR spectra (DMF, 25°C, 890-980 cm^{-1} region) of non-catalytic sample of **3$^{C(O)OMe}$** (50 mM), PPh_3 (500 mM), and DMSO-d$_6$ (2.5 M) at selected timepoints during 20 hour period in the dark (after initial 4 hour irradiation).

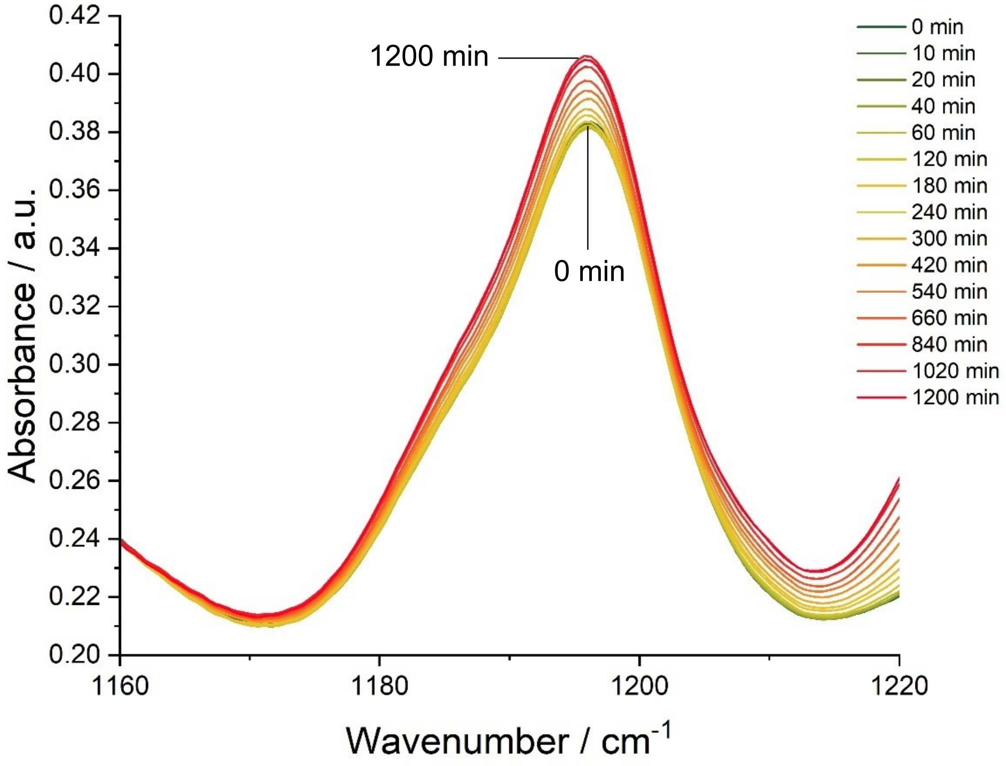

Figure S 66. FTIR spectra (DMF, 25°C, 1160-1220 cm^{-1} region) of non-catalytic sample of $\mathbf{3}^{\mathbf{C(O)OMe}}$ (50 mM), PPh_3 (500 mM), and DMSO-d_6 (2.5 M) at selected timepoints during 20 hour period in the dark (after initial 4 hour irradiation).

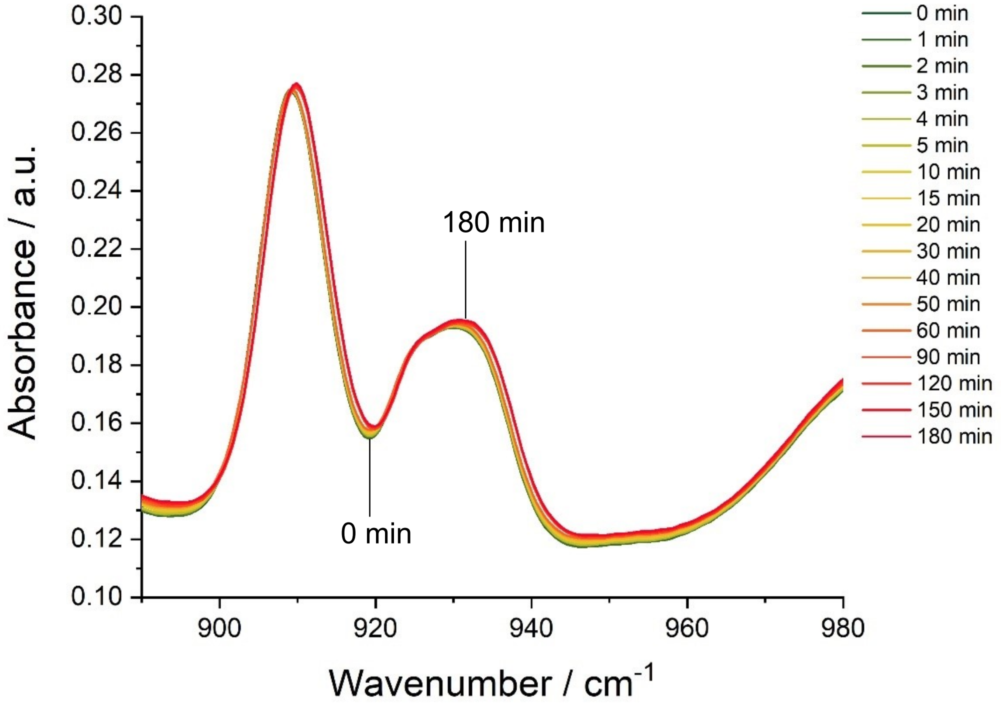

Figure S 67. FTIR spectra (DMF, 25°C, 890-980 cm^{-1} region) of non-catalytic sample of $\mathbf{1}^{\mathbf{H}}$ (50 mM), PPh_3 (500 mM), and DMSO-d_6 (2.5 M) at selected timepoints during 3 hour irradiation (410 nm).

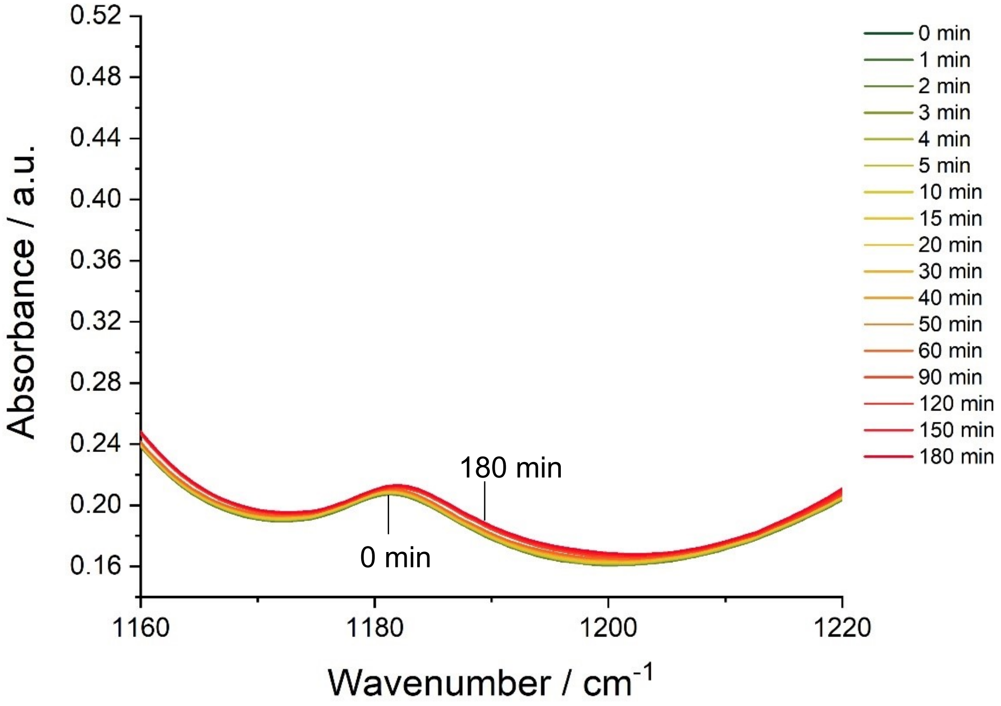

Figure S 68. FTIR spectra (DMF, 25°C, 1160-1220 cm^{-1} region) of non-catalytic sample of $\mathbf{1^{H}}$ (50 mM), PPh_3 (500 mM), and DMSO-d_6 (2.5 M) at selected timepoints during 3 hour irradiation (410 nm).

Phosphine and sulfoxide substrate scope

Reactions were prepared in a glove box under N_2 atmosphere. Acetone-d_6 and liquid substrates were dried over 3 Å molecular sieves and degassed *via* 3 freeze-pump-thaw cycles. Solutions of the samples in acetone-d_6 were prepared containing final concentrations 1 mM of catalyst $\mathbf{3^{C(O)OMe}}$, 300 mM of phosphine, and 300 mM of sulfoxide, and 700 µL portions were then transferred using a syringe into a Youngs NMR tube. The NMR tubes were inserted into the photoreactor (Fig. S1), and irradiated (λ = 410 nm) for 3 hours (or selected time intervals).

Different sulfoxide substrate conversions under photocatalytic conditions were determined using equimolar amounts of PPh_3 (**P1**) as the OAT acceptor (see Table S18); different phosphine substrate conversions under photocatalytic conditions were determined using equimolar amounts of sulfoxides **SO1** and **SO2** as the OAT donor (see Table S19).

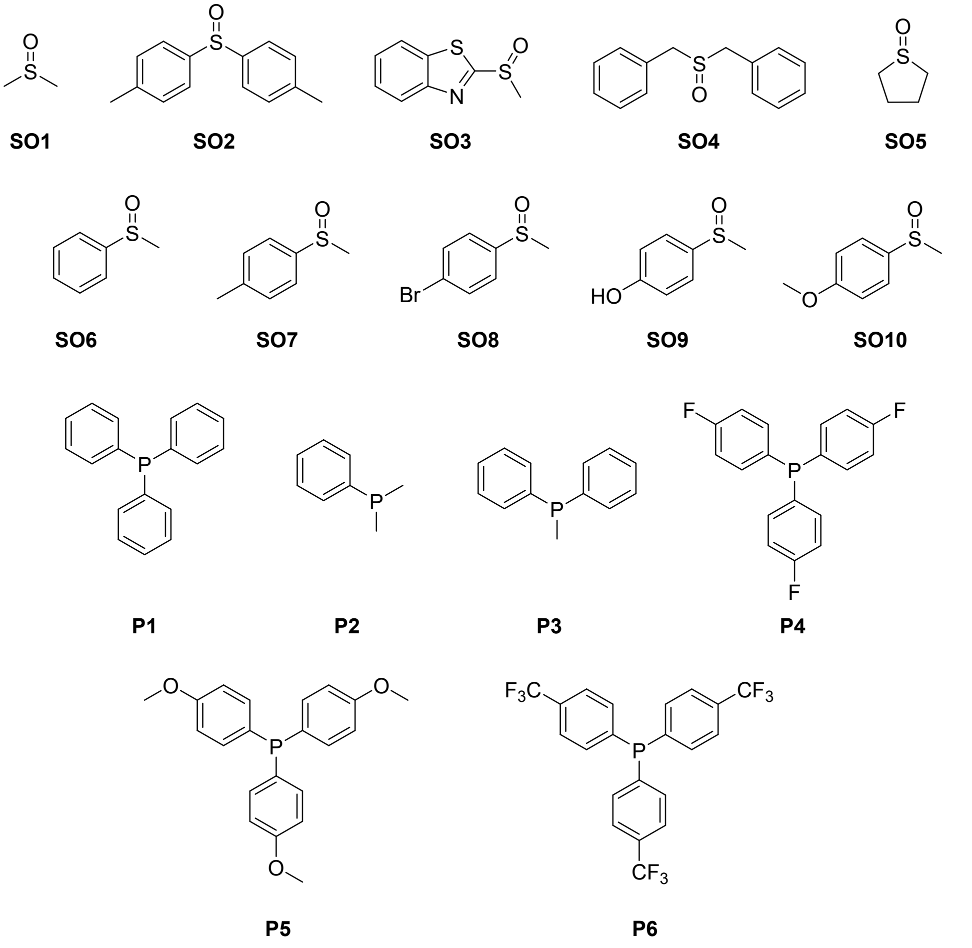

Figure S 69. Structures and numbering of phosphine and sulfoxide substrates.

Table S 18. Sulfoxide conversions (and respective PPh_3 conversions) under photocatalytic conditions using catalyst $\mathbf{3}^{\mathbf{C(O)OMe}}$.

Sulfoxide	Conversion [a] of sulfoxide to sulfide / %	Conversion [a] of PPh_3 to $OPPh_3$ / %
**SO1**	14	20
**SO2**	46	46
**SO3**	28	29
**SO4**	21	[b]
**SO5**	47	54
**SO6**	35	[b]
**SO7**	44	42
**SO8**	36	39
**SO9**	4	2
**SO10**	46	45

Discrepancy between conversions for sulfoxide and PPh_3 substrates are due to volatility of some sulfide products leading to smaller quantities being detected. [a] An uncertainty in the measurement of 2 % is assumed. [b] Determination not possible due to signal overlap.

Table S 19. Phosphine conversions under photocatalytic conditions using catalyst $\mathbf{3}^{\mathbf{C(O)OMe}}$ and sulfoxides **SO1** or **SO2**, and the Tolman and Hammett parameters for the respective phosphines.

.Phosphine	Sulfoxide	Conversion [a] / %
**P1**	**SO1**	18
**P2**	**SO1**	36
**P3**	**SO1**	42
**P1**	**SO2**	45
**P4**	**SO2**	14
**P5**	**SO2**	32
**P6**	**SO2**	40

[a] An uncertainty in the measurement of 2 % is estimated.

a)

b)

c)

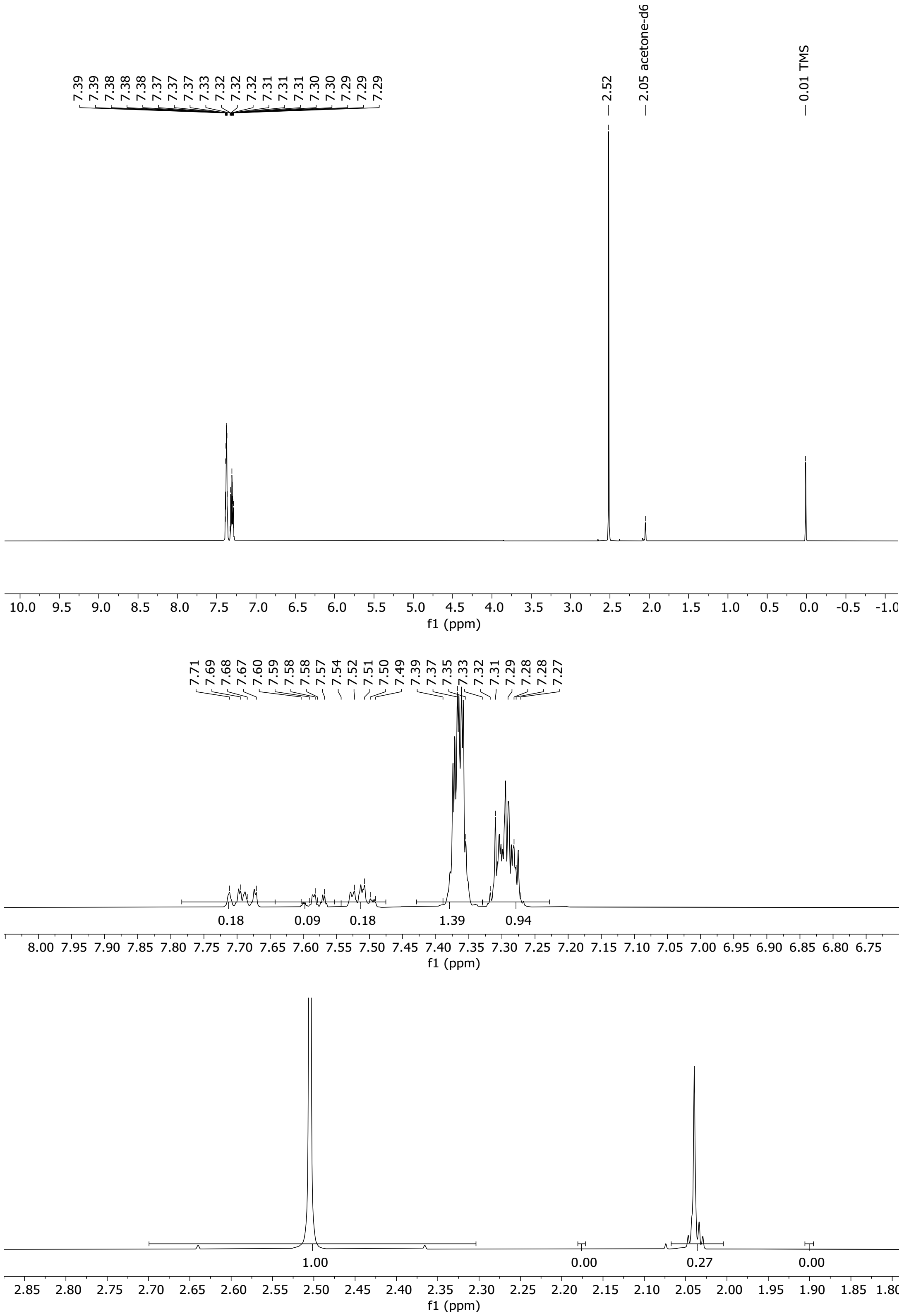

Figure S 70: ^{1}H NMR spectrum (500 MHz, acetone-d_6, 25 °C) of sample containing $\mathbf{3}^{\mathbf{C(O)OMe}}$ (1 mM), **P1** (300 mM), and **SO1** (300 mM) in acetone-d_6 (0.7 mL), after 3 h irradiation; a) full spectrum; b) aromatic region showing integration values for **P1** (and respective product) signals; c) aliphatic region, showing integration values for **SO1** (and respective sulfide product) signals.

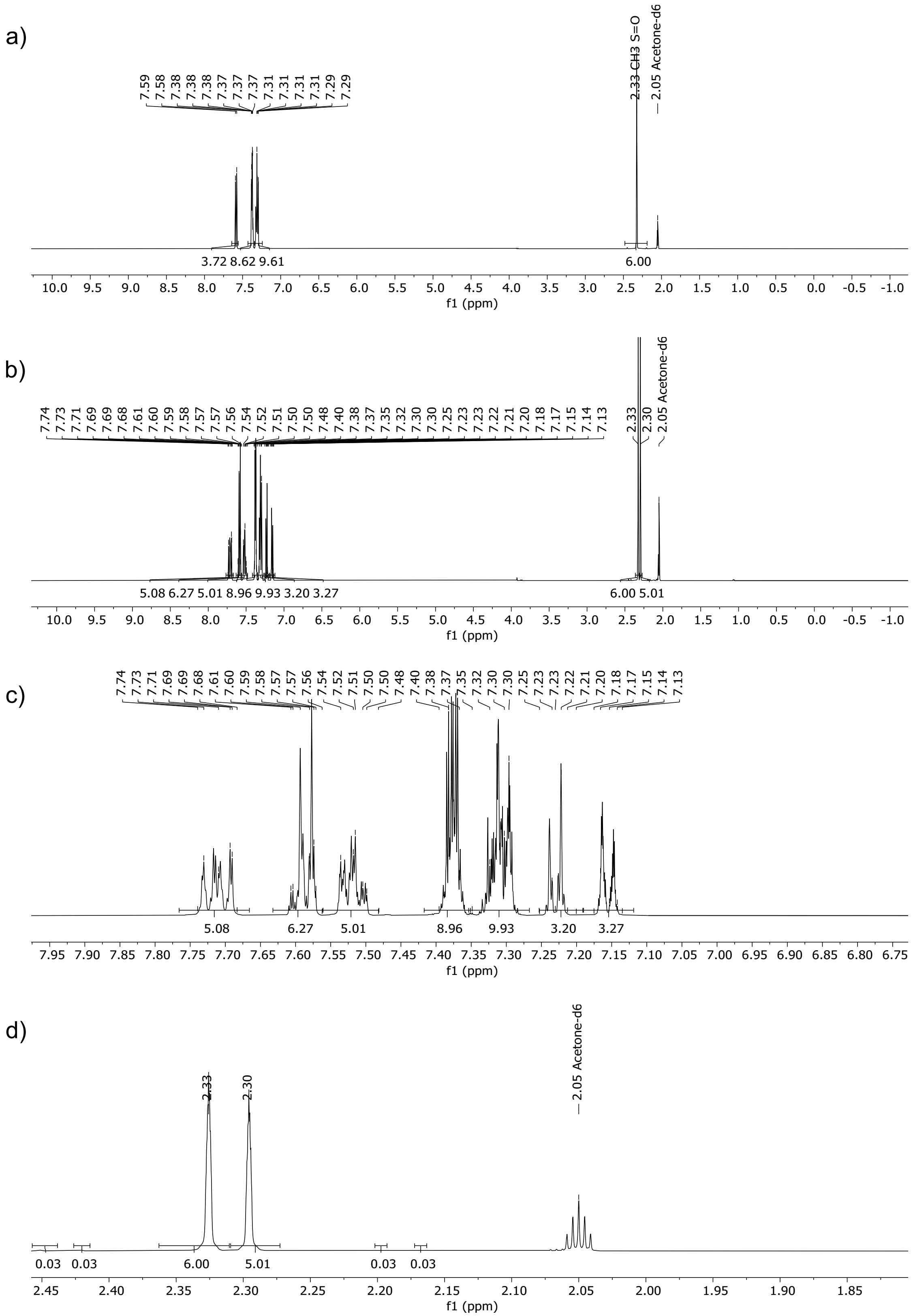

Figure S 71: ^{1}H NMR spectrum (500 MHz, acetone-d_6, 25 °C) of sample containing $\mathbf{3}^{\mathbf{C(O)OMe}}$ (1 mM), **P1** (300 mM), and **SO2** (300 mM) in acetone-d_6 (0.7 mL); a) before irradiation, full spectrum; b) after 3 h irradiation, full spectrum; c) after 3 h irradiation, aromatic region showing integration values for **P1/SO2** (and respective products) signals; d) after 3 h irradiation, aliphatic region showing integration values for **SO2** (and respective sulfide product) signals.

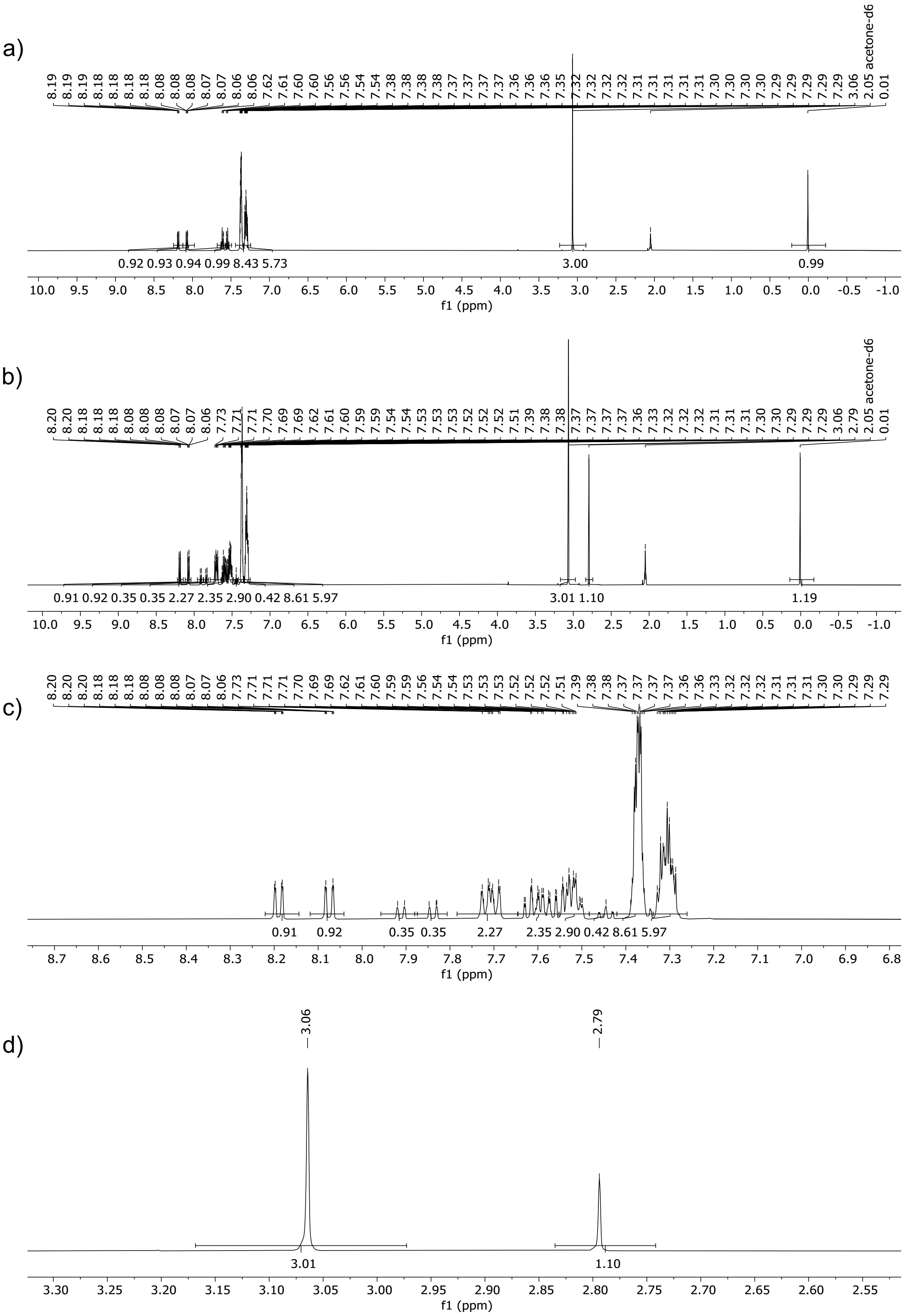

Figure S 72: ^{1}H NMR spectrum (500 MHz, acetone-d_6, 25 °C) of sample containing **3**$^{C(O)OMe}$ (1 mM), **P1** (300 mM), and **SO3** (300 mM) in acetone-d_6 (0.7 mL); a) before irradiation, full spectrum; b) after 3 h irradiation, full spectrum; c) after 3 h irradiation, aromatic region showing integration values for **P1/SO3** (and respective products) signals; d) after 3 h irradiation, aliphatic region showing integration values for **SO3** (and respective sulfide product) signals.

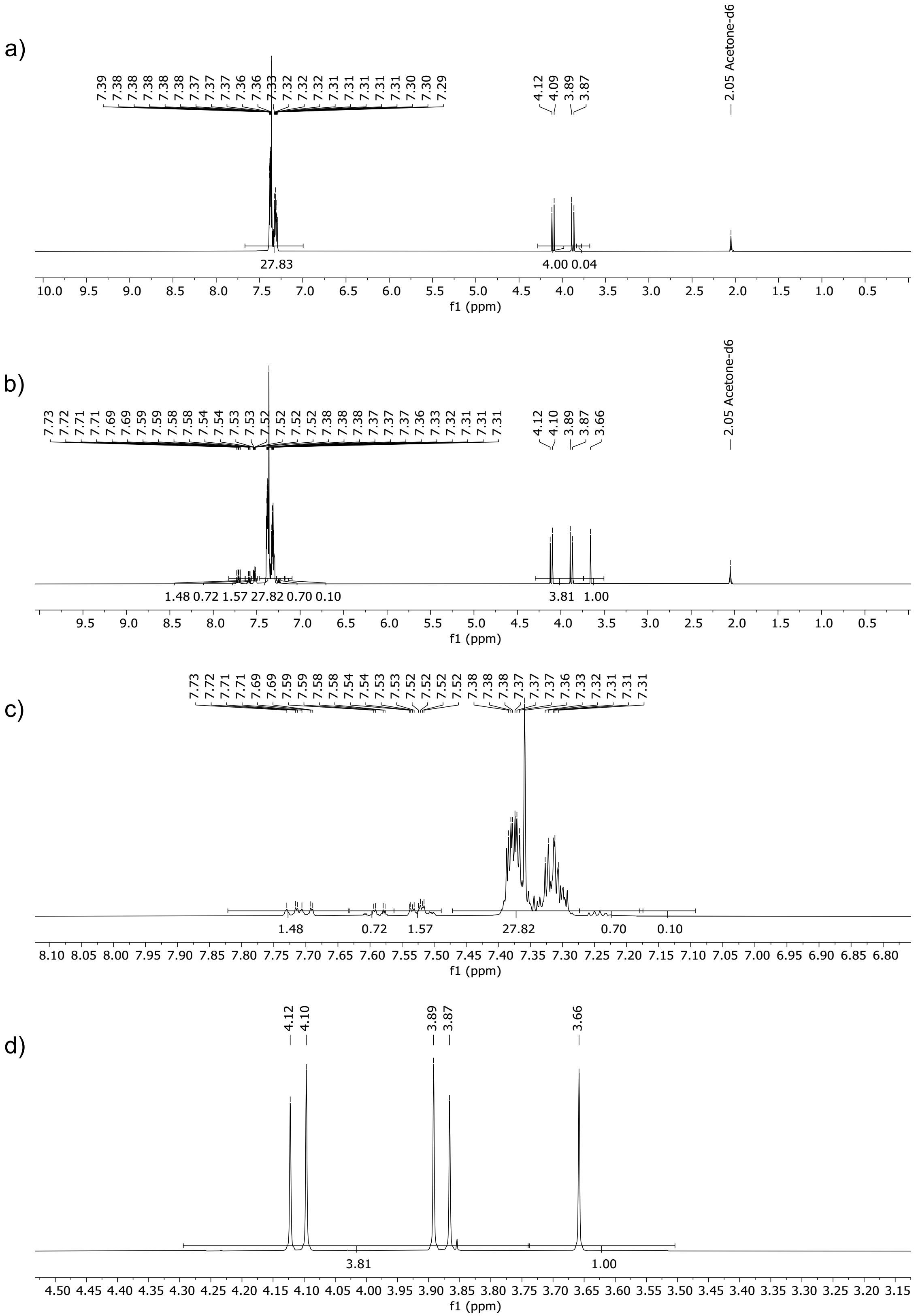

Figure S 73: ^{1}H NMR spectrum (500 MHz, acetone-d_6, 25 °C) of sample containing **3$^{C(O)OMe}$** (1 mM), **P1** (300 mM), and **SO4** (300 mM) in acetone-d_6 (0.7 mL); a) before irradiation, full spectrum; b) after 3 h irradiation, full spectrum; c) after 3 h irradiation, aromatic region showing integration values for **P1/SO4** (and respective products) signals; d) after 3 h irradiation, aliphatic region showing integration values for **SO4** (and respective sulfide product) signals.

a)

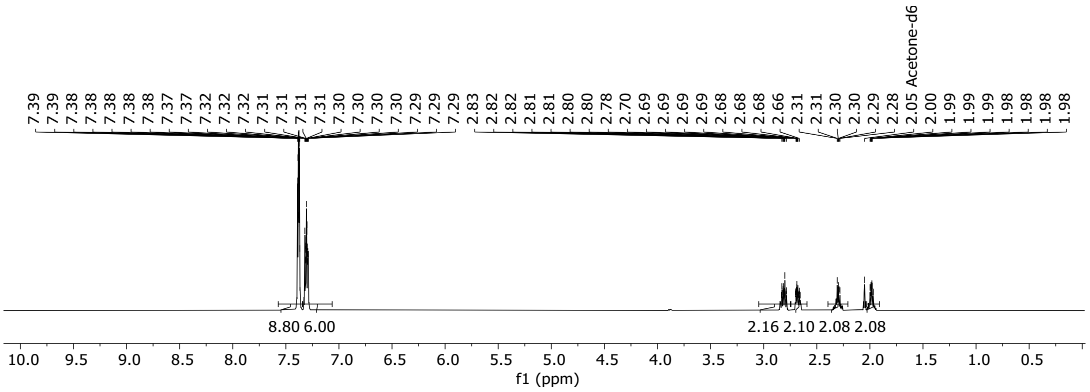

b)

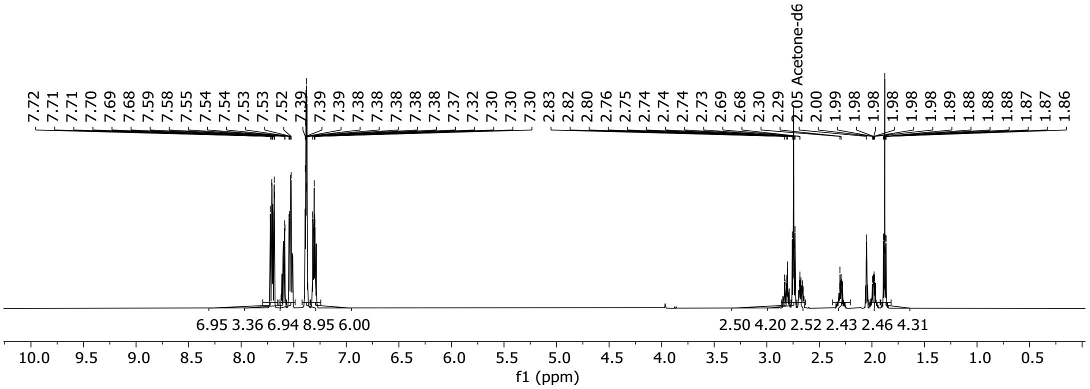

c)

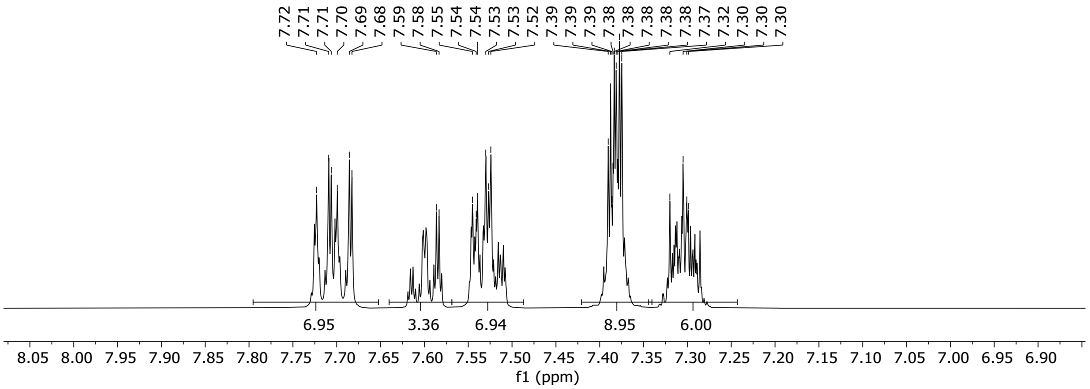

d)

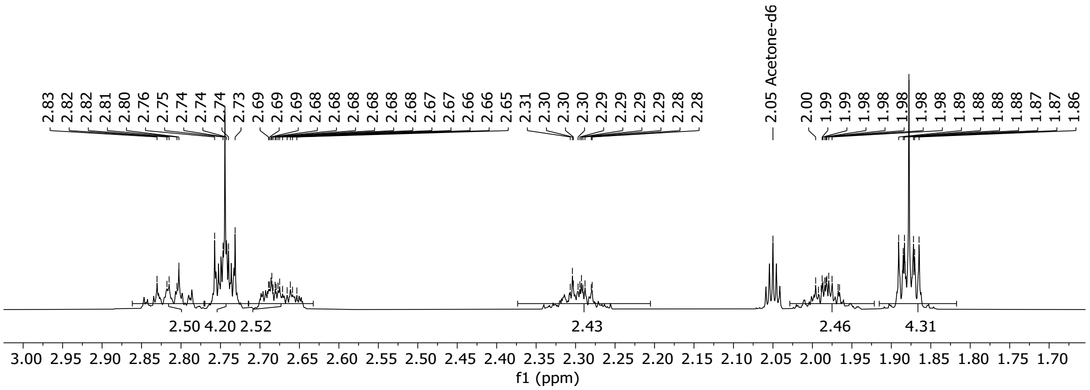

Figure S 74: ^{1}H NMR spectrum (500 MHz, acetone-d_6, 25 °C) of sample containing $\mathbf{3}^{\mathbf{C(O)OMe}}$ (1 mM), **P1** (300 mM), and **SO5** (300 mM) in acetone-d_6 (0.7 mL); a) before irradiation, full spectrum; b) after 3 h irradiation, full spectrum; c) after 3 h irradiation, aromatic region showing integration values for **P1/SO5** (and respective products) signals; d) after 3 h irradiation, aliphatic region showing integration values for **SO5** (and respective sulfide product) signals.

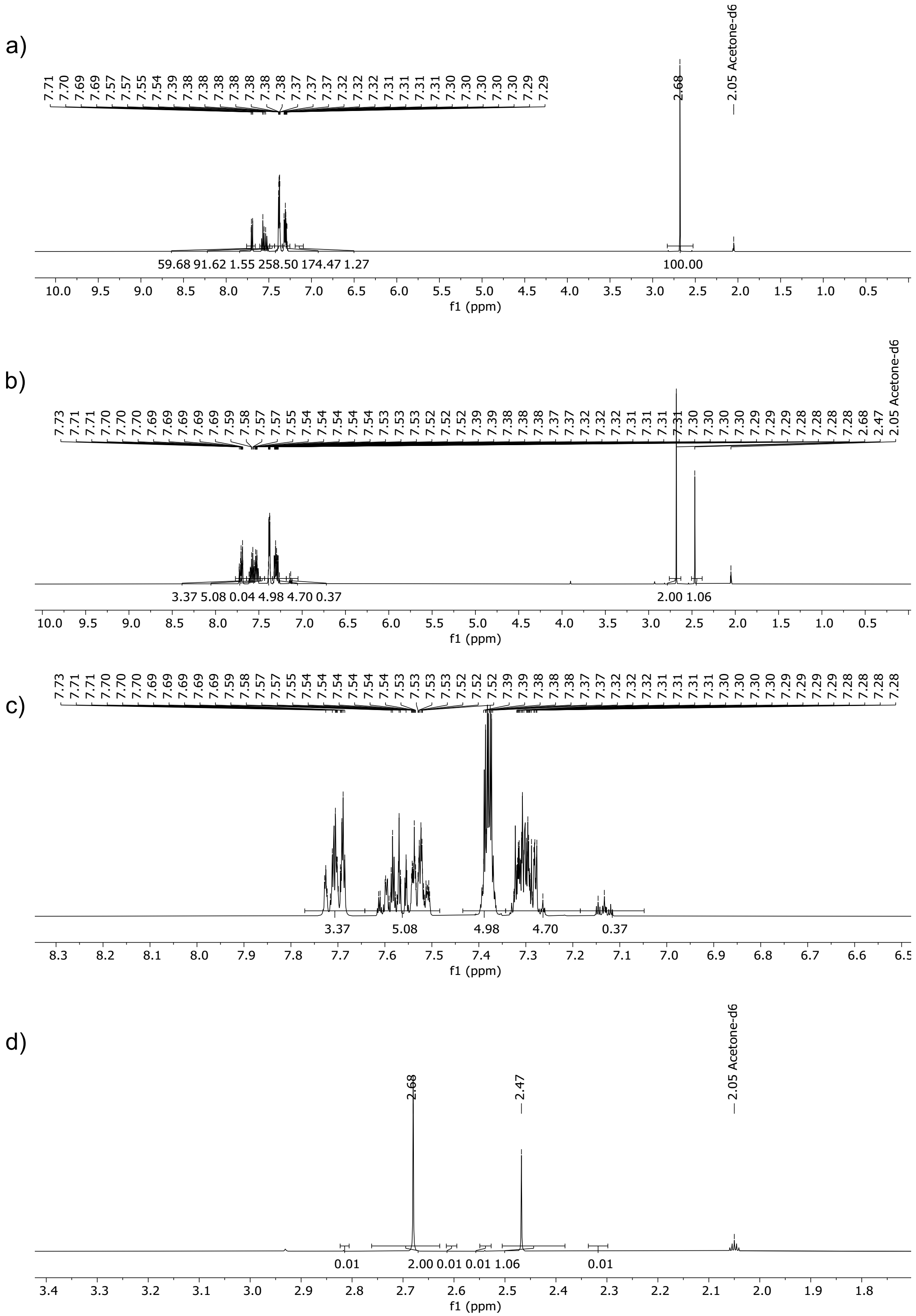

Figure S 75: ^{1}H NMR spectrum (500 MHz, acetone-d$_6$, 25 °C) of sample containing **3**$^{\mathbf{C(O)OMe}}$ (1 mM), **P1** (300 mM), and **SO6** (300 mM) in acetone-d$_6$ (0.7 mL); a) before irradiation, full spectrum; b) after 3 h irradiation, full spectrum; c) after 3 h irradiation, aromatic region showing integration values for **P1/SO6** (and respective products) signals; d) after 3 h irradiation, aliphatic region showing integration values for **SO6** (and respective sulfide product) signals.

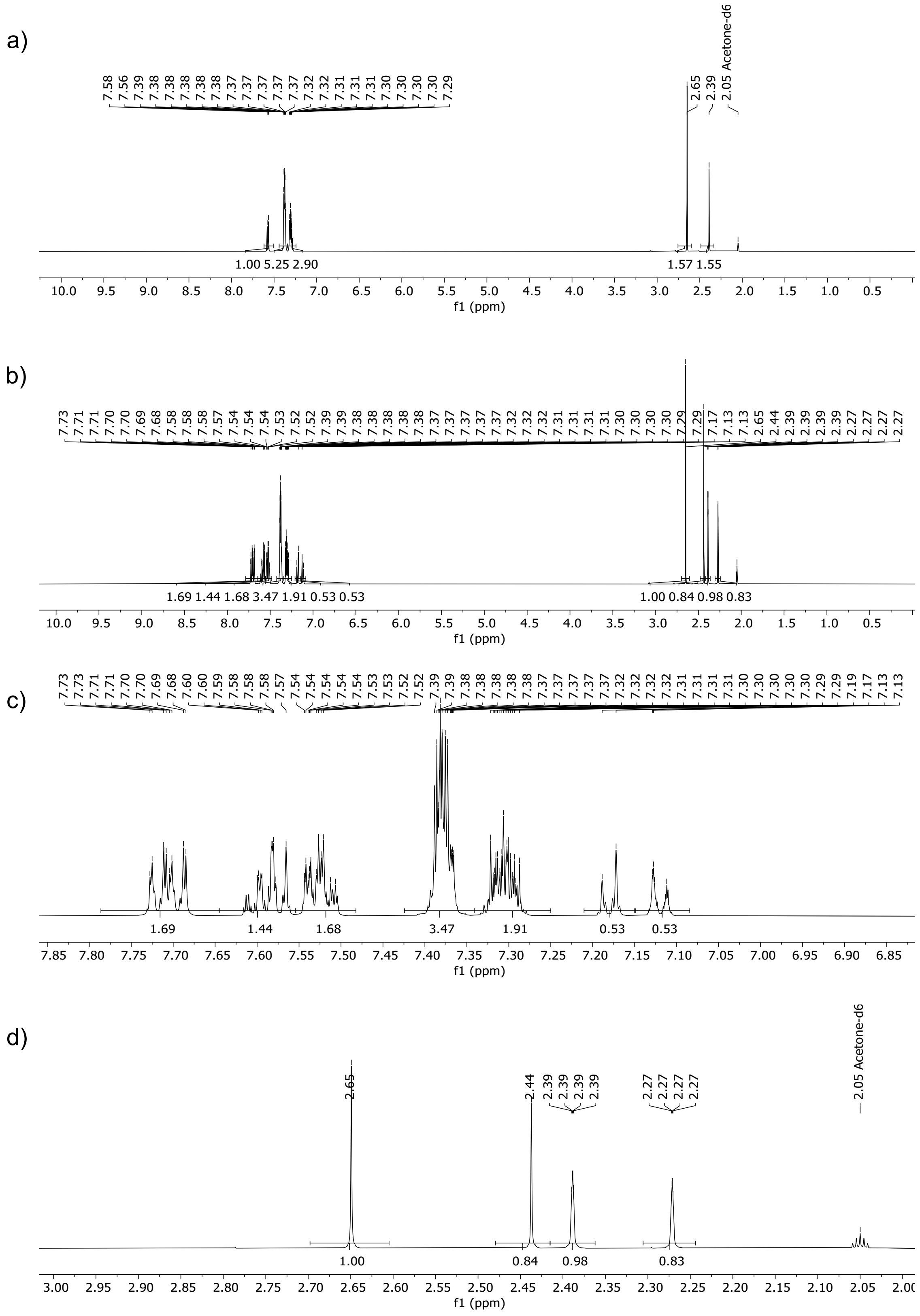

Figure S 76: ^{1}H NMR spectrum (500 MHz, acetone-d$_6$, 25 °C) of sample containing **3$^{C(O)OMe}$** (1 mM), **P1** (300 mM), and **SO7** (300 mM) in acetone-d$_6$ (0.7 mL); a) before irradiation, full spectrum; b) after 3 h irradiation, full spectrum; c) after 3 h irradiation, aromatic region showing integration values for **P1/SO7** (and respective products) signals; d) after 3 h irradiation, aliphatic region showing integration values for **SO7** (and respective sulfide product) signals.

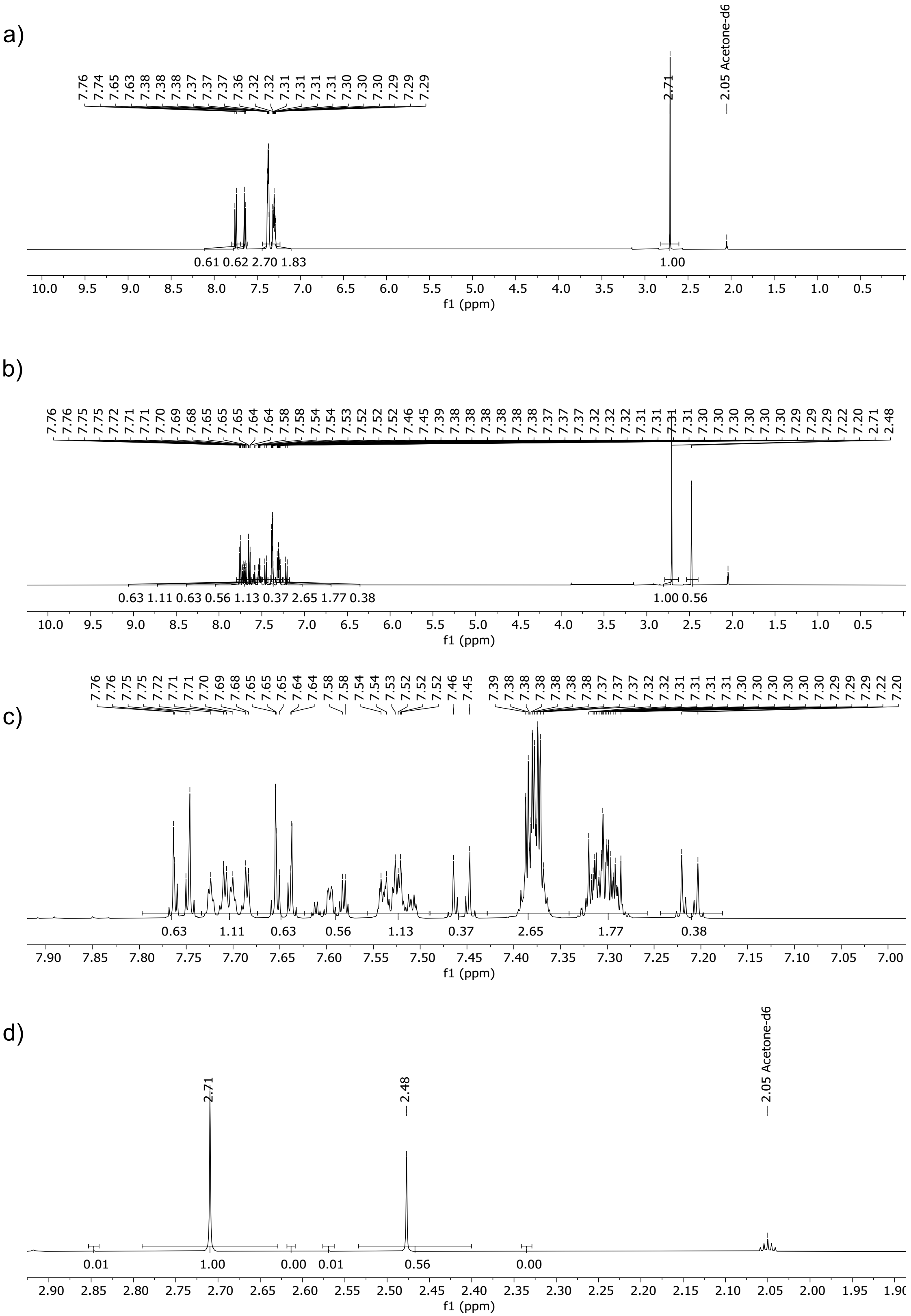

Figure S 77: ^{1}H NMR spectrum (500 MHz, acetone-d$_6$, 25 °C) of sample containing **3$^{C(O)OMe}$** (1 mM), **P1** (300 mM), and **SO8** (300 mM) in acetone-d$_6$ (0.7 mL); a) before irradiation, full spectrum; b) after 3 h irradiation, full spectrum; c) after 3 h irradiation, aromatic region showing integration values for **P1/SO8** (and respective products) signals; d) after 3 h irradiation, aliphatic region showing integration values for **SO8** (and respective sulfide product) signals.

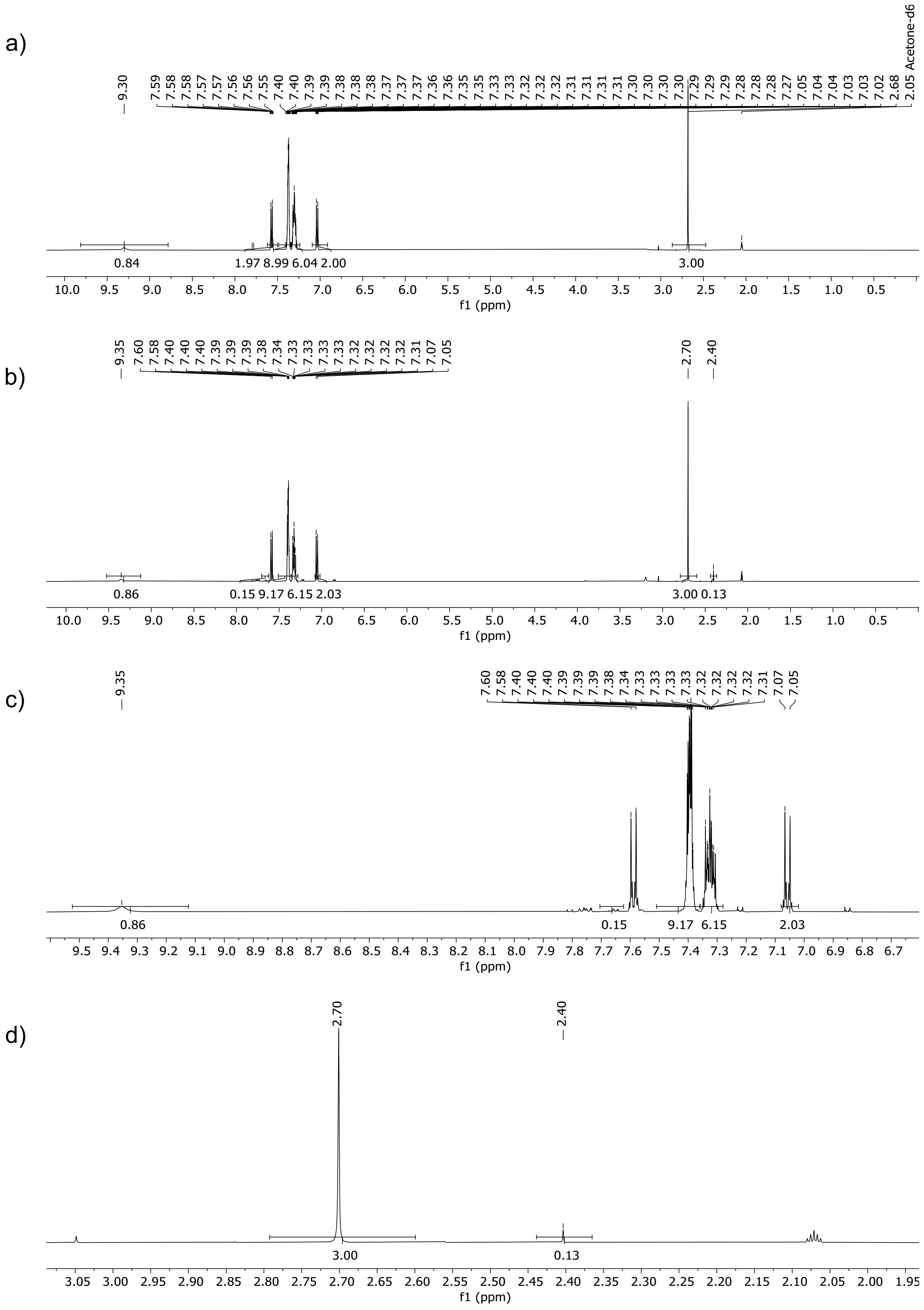

Figure S 78: ^{1}H NMR spectrum (500 MHz, acetone-d_6, 25 °C) of sample containing **3$^{C(O)OMe}$** (1 mM), **P1** (300 mM), and **SO9** (300 mM) in acetone-d_6 (0.7 mL); a) before irradiation, full spectrum; b) after 3 h irradiation, full spectrum; c) after 3 h irradiation, aromatic region showing integration values for **P1/SO9** (and respective products) signals; d) after 3 h irradiation, aliphatic region showing integration values for **SO9** (and respective sulfide product) signals.

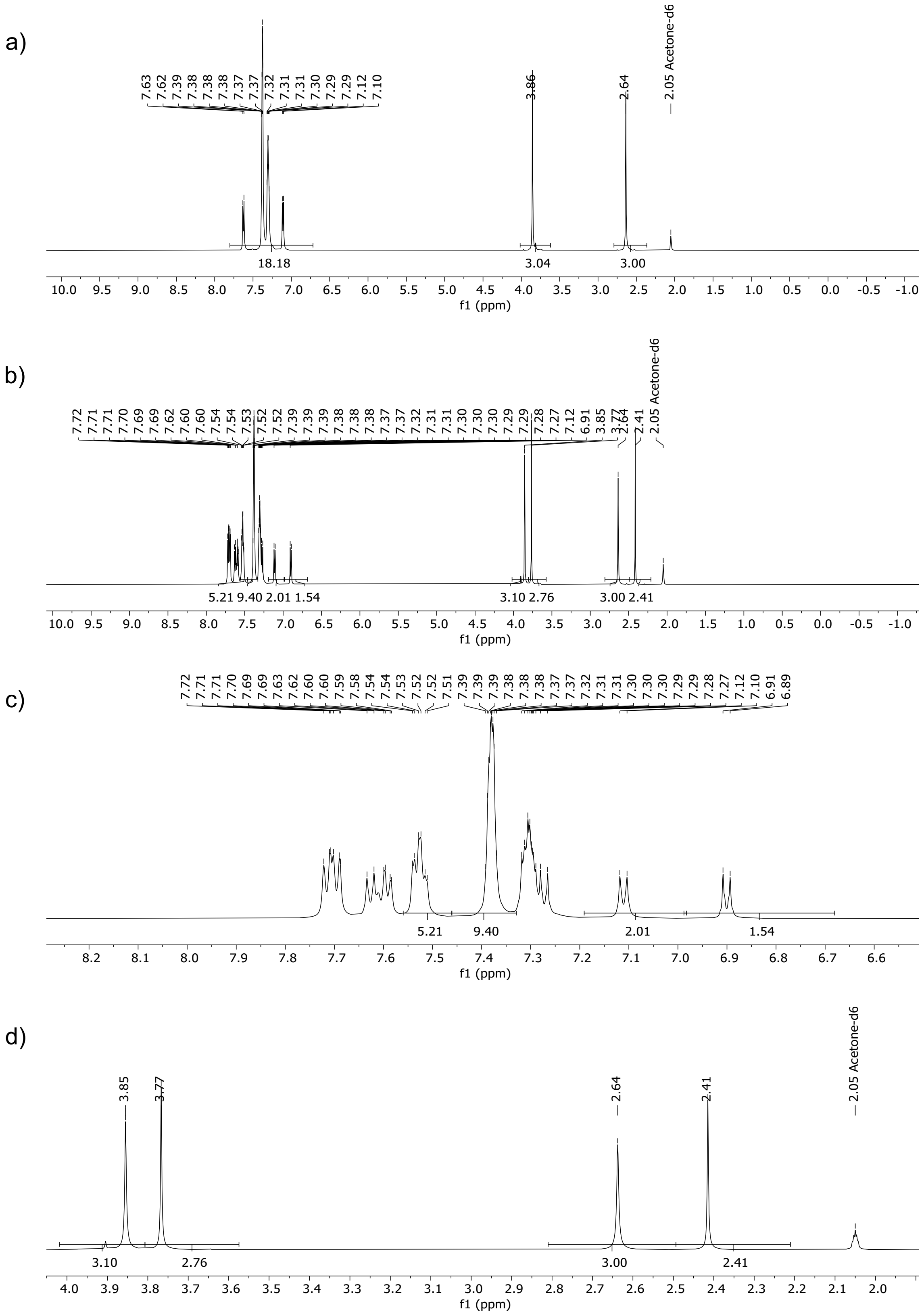

Figure S 79: ^{1}H NMR spectrum (500 MHz, acetone-d_6, 25 °C) of sample containing $\mathbf{3}^{\mathbf{C(O)OMe}}$ (1 mM), **P1** (300 mM), and **SO10** (300 mM) in acetone-d_6 (0.7 mL); a) before irradiation, full spectrum; b) after 3 h irradiation, full spectrum; c) after 3 h irradiation, aromatic region showing integration values for **P1/SO10** (and respective products) signals; d) after 3 h irradiation, aliphatic region showing integration values for **SO10** (and respective sulfide product) signals.

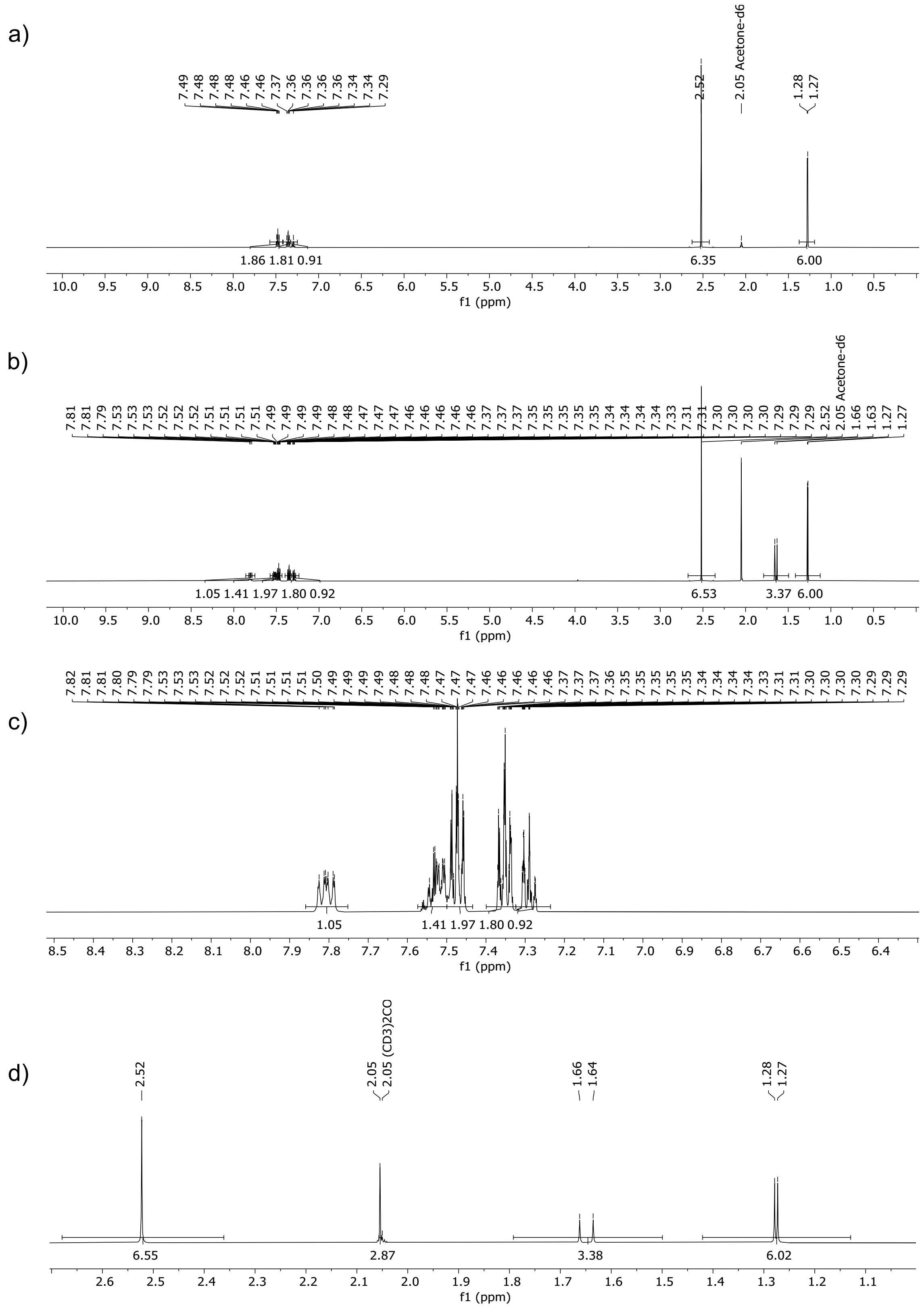

Figure S 80: ^{1}H NMR spectrum (500 MHz, acetone-d_6, 25 °C) of sample containing $\mathbf{3}^{\mathbf{C(O)OMe}}$ (1 mM), **P2** (300 mM), and **SO1** (300 mM) in acetone-d_6 (0.7 mL); a) before irradiation, full spectrum; b) after 3 h irradiation, full spectrum; c) after 3 h irradiation, aromatic region showing integration values for **P2** (and respective product) signals; d) after 3 h irradiation, aliphatic region showing integration values for **P2/SO1** (and respective products) signals.

a)

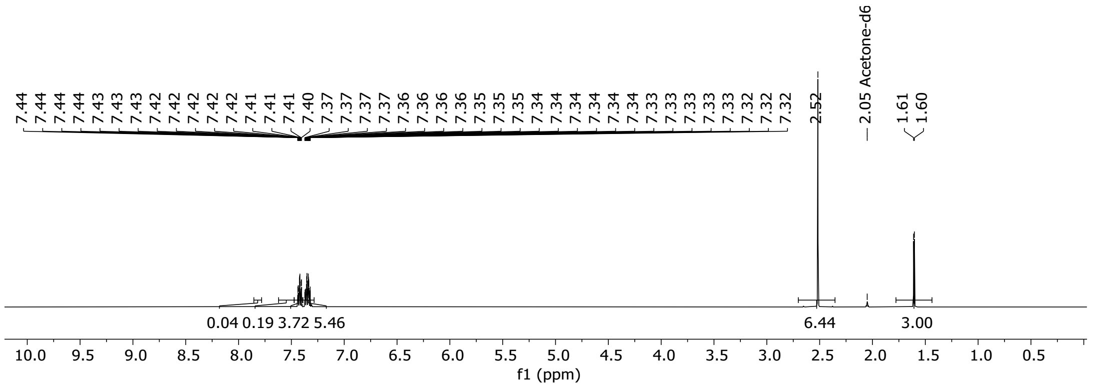

b)

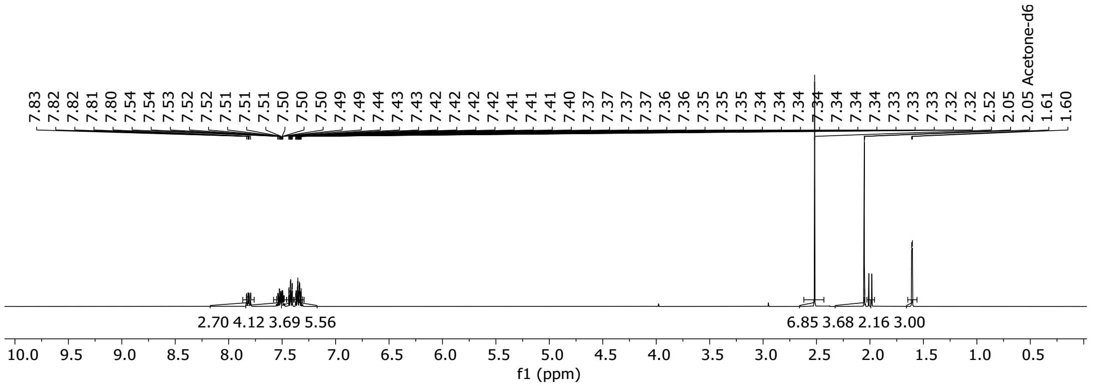

c)

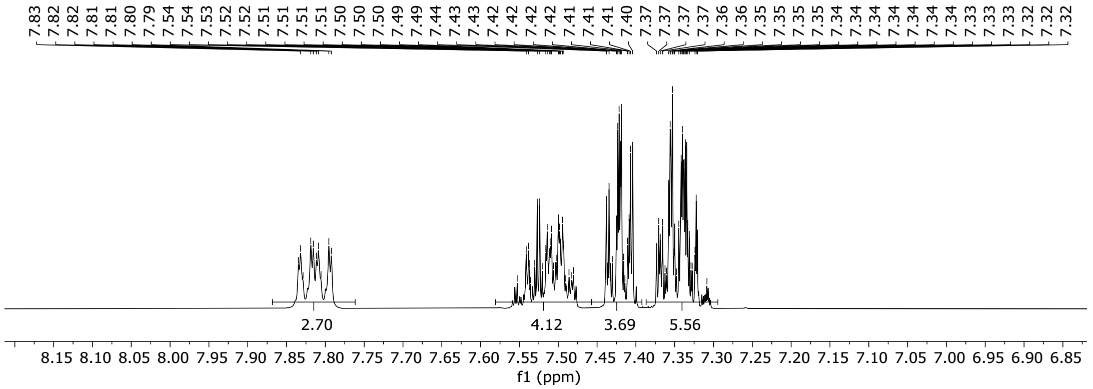

d)

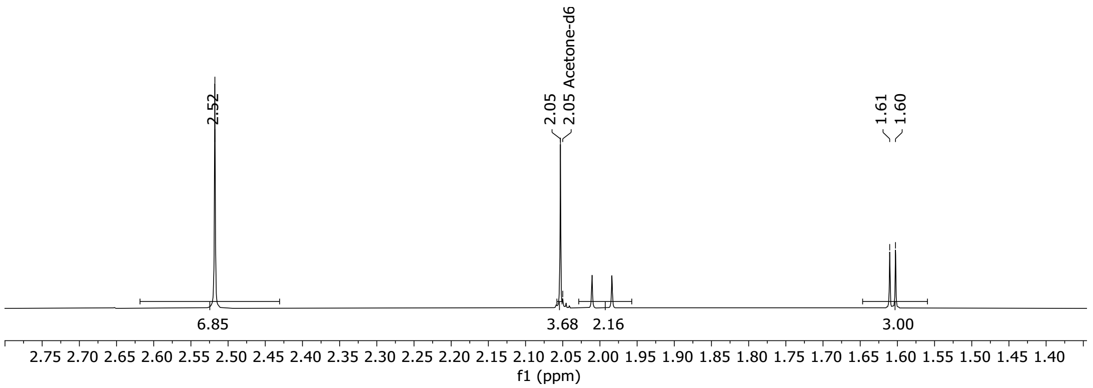

Figure S 81 ^{1}H NMR spectrum (500 MHz, acetone-d_6, 25 °C) of sample containing **3**$^{\mathbf{C(O)OMe}}$ (1 mM), **P3** (300 mM), and **SO1** (300 mM) in acetone-d_6 (0.7 mL); a) before irradiation, full spectrum; b) after 3 h irradiation, full spectrum; c) after 3 h irradiation, aromatic region showing integration values for **P3** (and respective product) signals; d) after 3 h irradiation, aliphatic region showing integration values for **P3/SO1** (and respective products) signals.

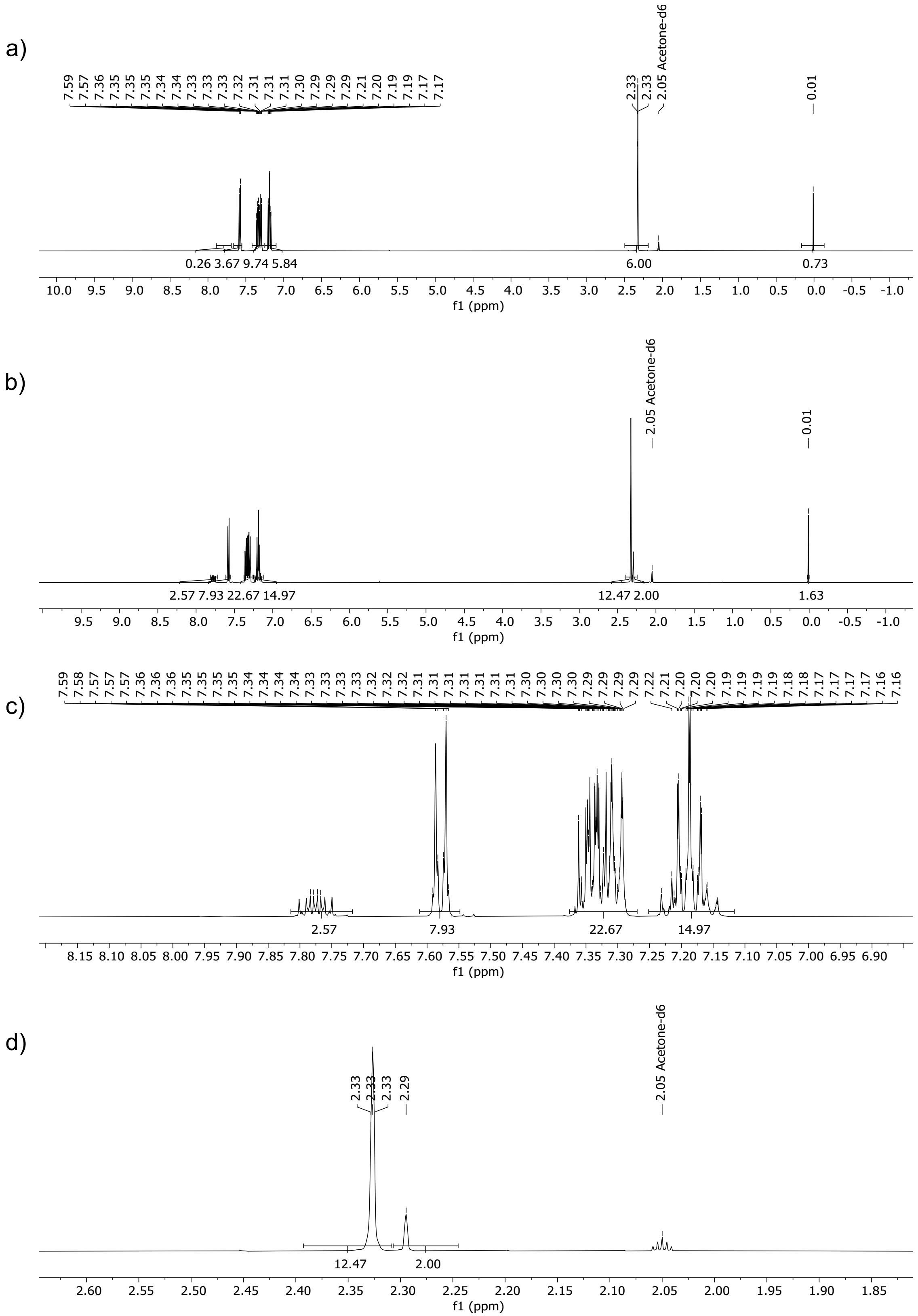

Figure S 82: ^{1}H NMR spectrum (500 MHz, acetone-d_6, 25 °C) of sample containing **3**$^{C(O)OMe}$ (1 mM), **P4** (300 mM), and **SO2** (300 mM) in acetone-d_6 (0.7 mL); a) before irradiation, full spectrum; b) after 3 h irradiation, full spectrum; c) after 3 h irradiation, aromatic region showing integration values for **P4/SO2** (and respective products) signals; d) after 3 h irradiation, aliphatic region showing integration values for **SO2** (and respective product) signals.

a) b) c) d)

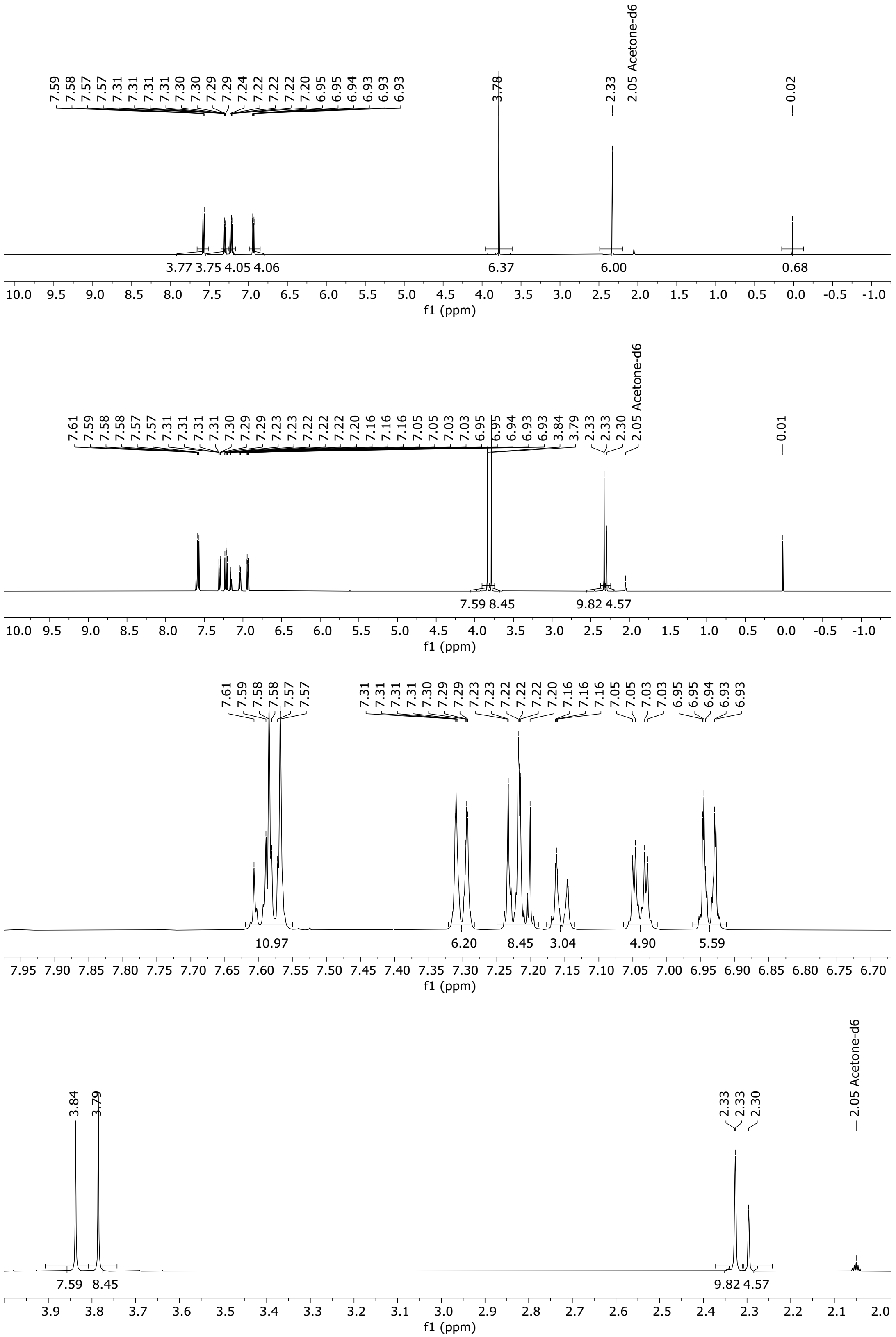

Figure S 83: ^{1}H NMR spectrum (500 MHz, acetone-d_6, 25 °C) of sample containing **$3^{C(O)OMe}$** (1 mM), **P5** (300 mM), and **SO2** (300 mM) in acetone-d_6 (0.7 mL); a) before irradiation, full spectrum; b) after 3 h irradiation, full spectrum; c) after 3 h irradiation, aromatic region showing integration values for **P5/SO2** (and respective products) signals; d) after 3 h irradiation, aliphatic region showing integration values for **SO2** (and respective product) signals.

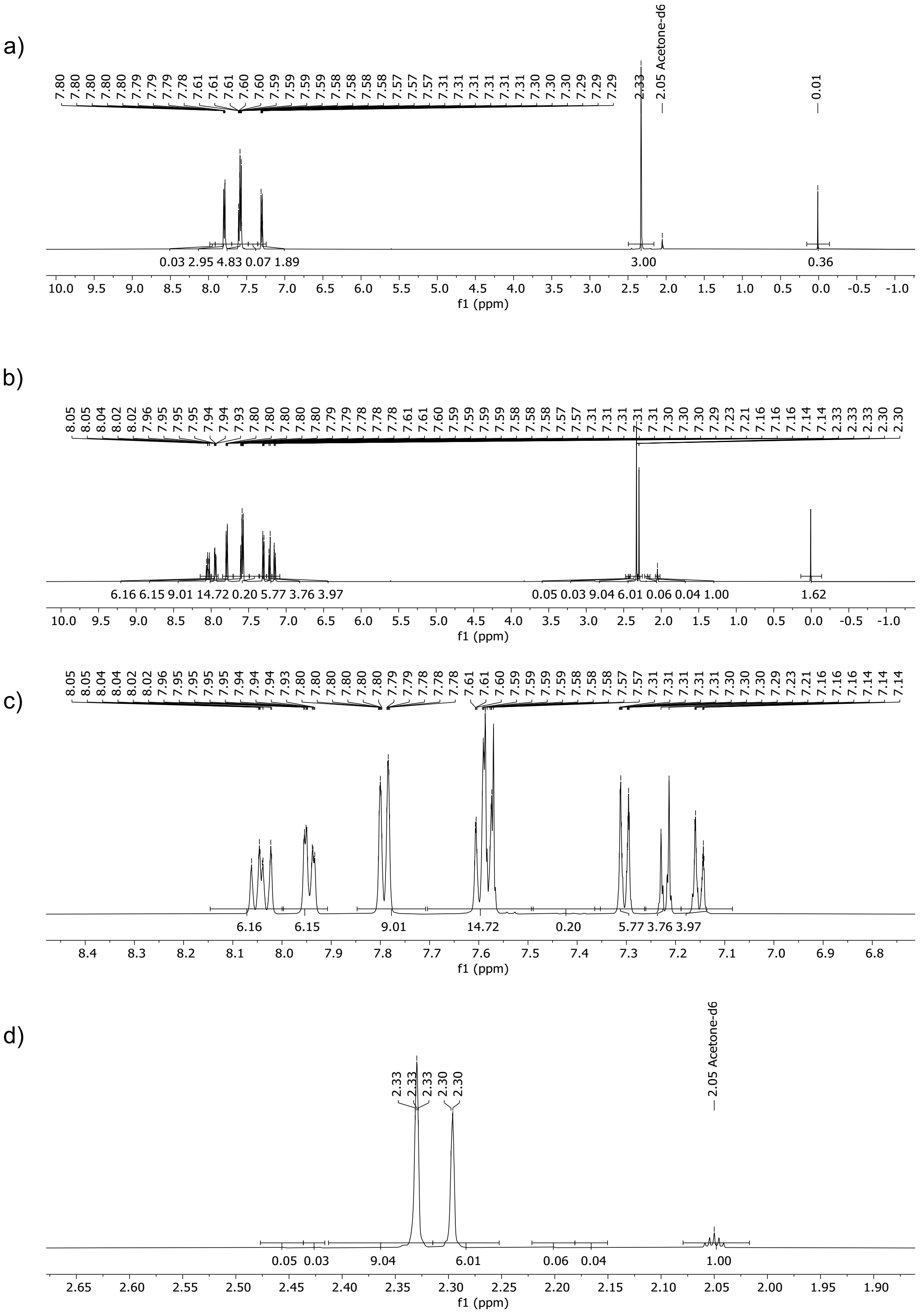

Figure S 84: ^{1}H NMR spectrum (500 MHz, acetone-d_6, 25 °C) of sample containing **$3^{C(O)OMe}$** (1 mM), **P6** (300 mM), and **SO2** (300 mM) in acetone-d_6 (0.7 mL); a) before irradiation, full spectrum; b) after 3 h irradiation, full spectrum; c) after 3 h irradiation, aromatic region showing integration values for **P6/SO2** (and respective products) signals; d) after 3 h irradiation, aliphatic region showing integration values for **P6/SO2** (and respective products) signals.

a)

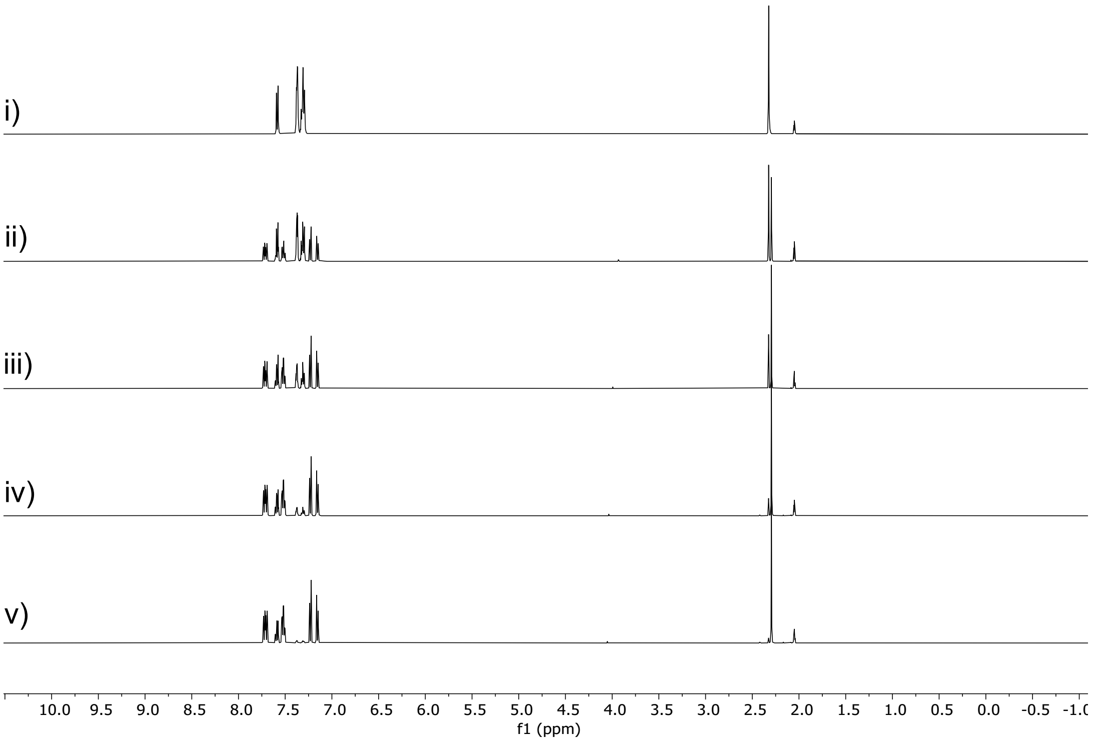

b)

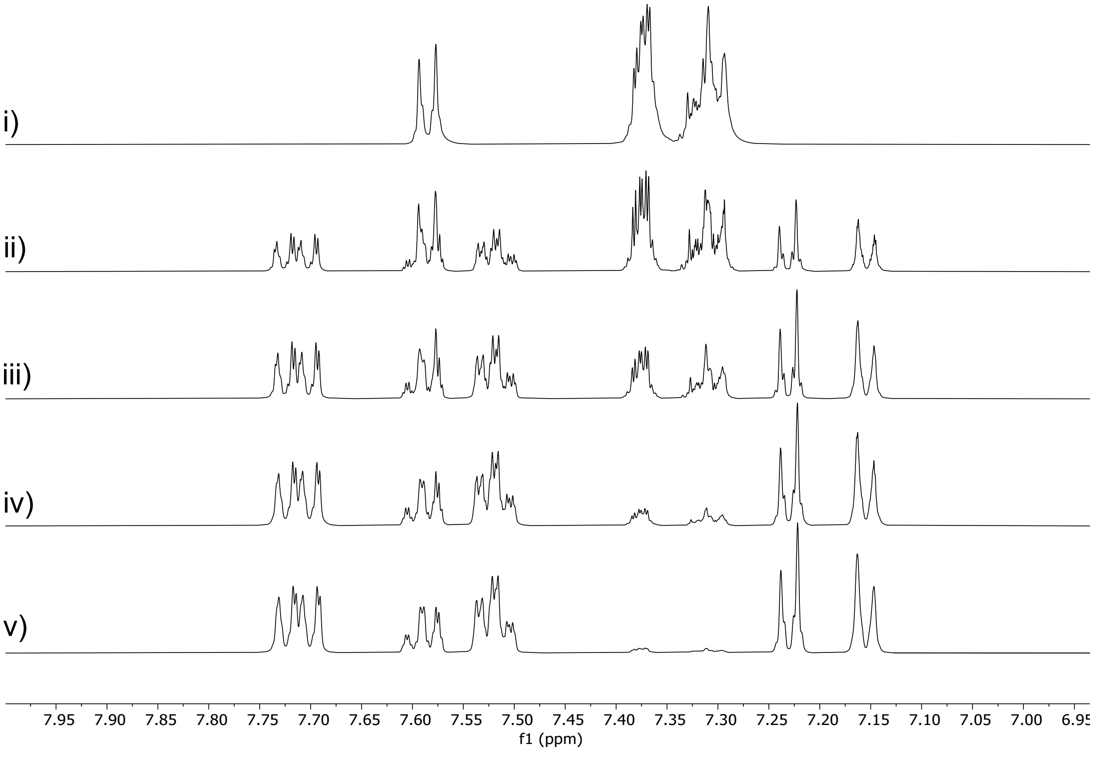

Figure S 85: ^{1}H NMR spectra (500 MHz, acetone-d_6, 25°C) of sample containing $\mathbf{3}^{\mathbf{C(O)OMe}}$ (1 mM), **P1** (300 mM), and **SO2** (300 mM) in acetone-d_6 (0.7 mL); a) full spectrum; b) aromatic region, showing proton signals for **P1/SO2** (and respective products after irradiation; i) before irradiation; ii) after 3 h irradiation; iii) after 9 h irradiation; iv) after 24 h irradiation; v) after 48 h irradiation.

Reactions between $3^{C(O)Me}$ and sacrificial electron donors TEA, DMA and BIH

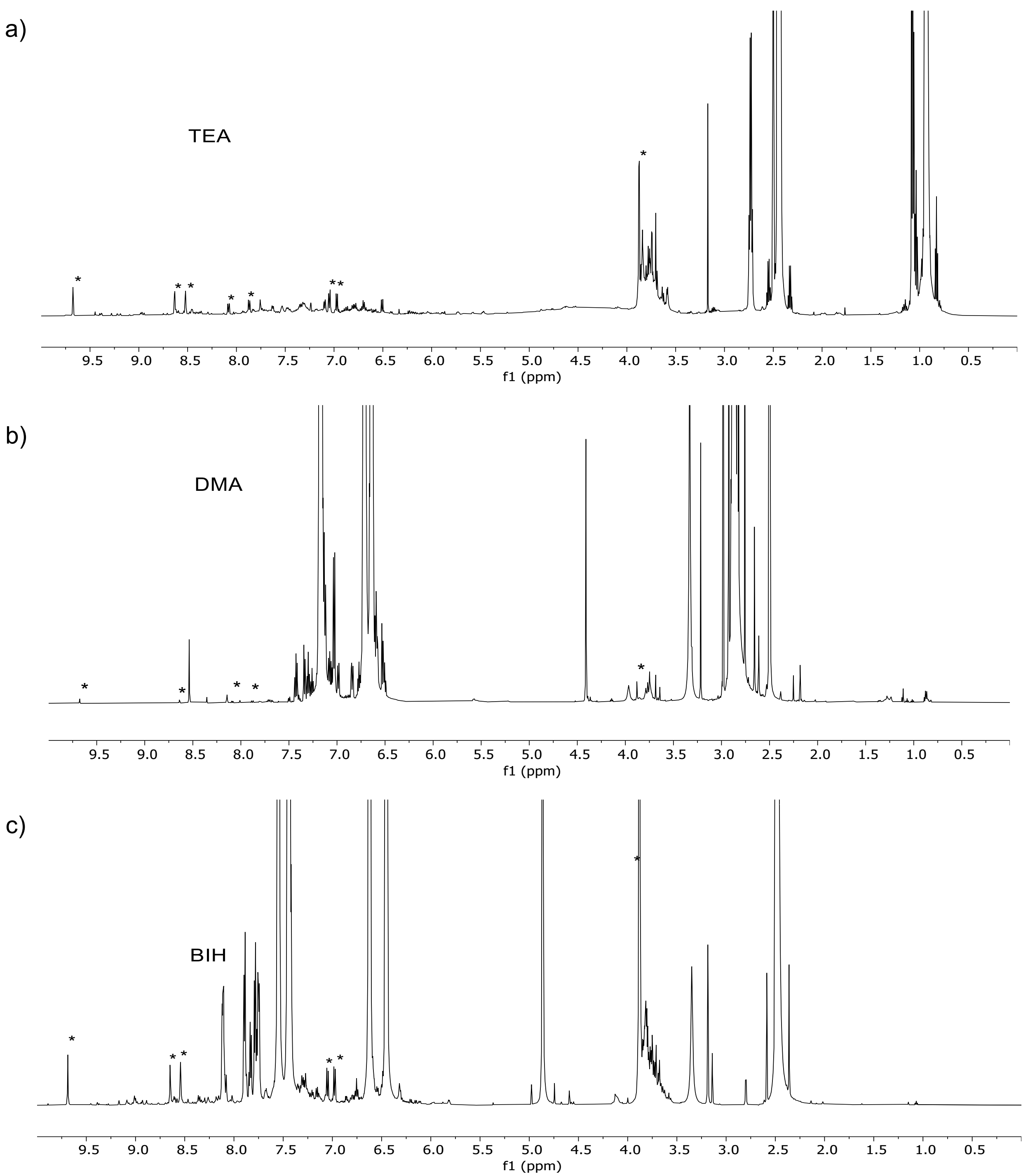

Figure S 86. ^{1}H NMR spectra (600 MHz; DMSO-d_6, 25°C) of reactions between $\mathbf{3}^{\mathbf{C(O)Me}}$ (30 mM) and a) TEA (300 mM); b) DMA (300 mM); and c) BIH (300 mM) in DMSO-d_6 after irradiation at 410 nm for 3 h.

Radical scavenging experiments with TEMPO

Samples were tested in order to see whether PPh_3 oxidation would be diminished in the presence of a radical scavenger, in order to probe the radical-type catalytic mechanism. Appropriate amounts of complex $\mathbf{3}^{\mathbf{C(O)OMe}}$, PPh_3, DMSO-d_6, (2,2,6,6-tetramethylpiperidin-1-yl)oxyl (TEMPO), 2,2,6,6-tetramethyl-piperidine (TMP), and 2,2,6,6-tetramethylpiperidin-1-ol (TEMPOH) were dissolved in 1 mL DMF under N_2 atmosphere to give a sample with final concentrations outlined in Table S20. Upon full dissolution of all components, 50 μL aliquot of each sample was added to the fluid FTIR cell (Harrick flow cell), and the FTIR spectra at 0 h timepoints were recorded. The samples were subsequently irradiated at 410 nm for continuous 3 h period within the liquid IR cell; the FTIR spectra of the samples were recorded again after irradiation.

Table S 20. Solution sample composition for radical scavenger experiments.

Sample number	Final component concentration / mM						Irradiation time / h
	$\mathbf{3}^{\mathbf{C(O)OMe}}$	PPh_3	DMSO-d_6	TEMPO	TMP	TEMPOH	
01	50	500	500	500	-	-	3
02	50	500	500	-	-	-	1
03	50	-	-	500	-	-	0.5
04	50	-	-	-	500	-	0.5
05	50	-	-	-	-	500	0.5

Overall, radical scavenging experiments with TEMPO were unsuccessful mainly due to TEMPO reacting in the catalytic reaction as an oxygen atom donor to the catalyst intermediate (alongside DMSO).[29,30] Samples 04 and 05 showcase that TMP and TEMPOH (likely by-products of TEMPO deoxygenation) readily react with $\mathbf{3}^{\mathbf{C(O)OMe}}$ in solution after irradiation, which complicates the determination of catalytic species formed in any of the samples.

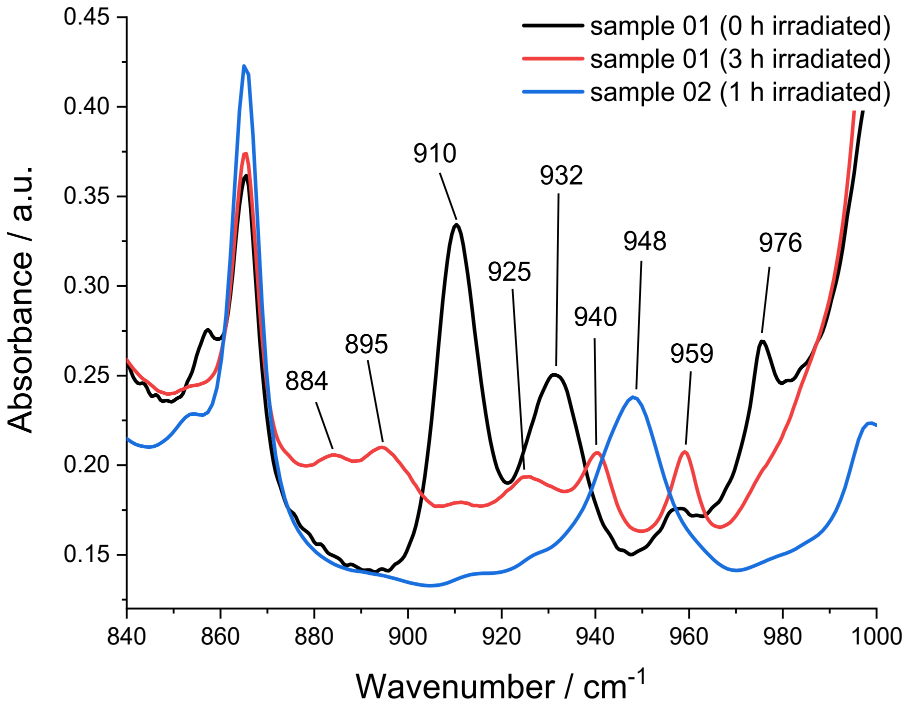

Figure S 87. FTIR spectra (840-1000 cm^{-1} region) of the sample 01 before irradiation (black line), sample 01 after 3 h irradiation (red line), and sample 02 after 1 h irradiation (blue line). For sample composition, see Table S20; samples were irradiated at λ = 410 nm.

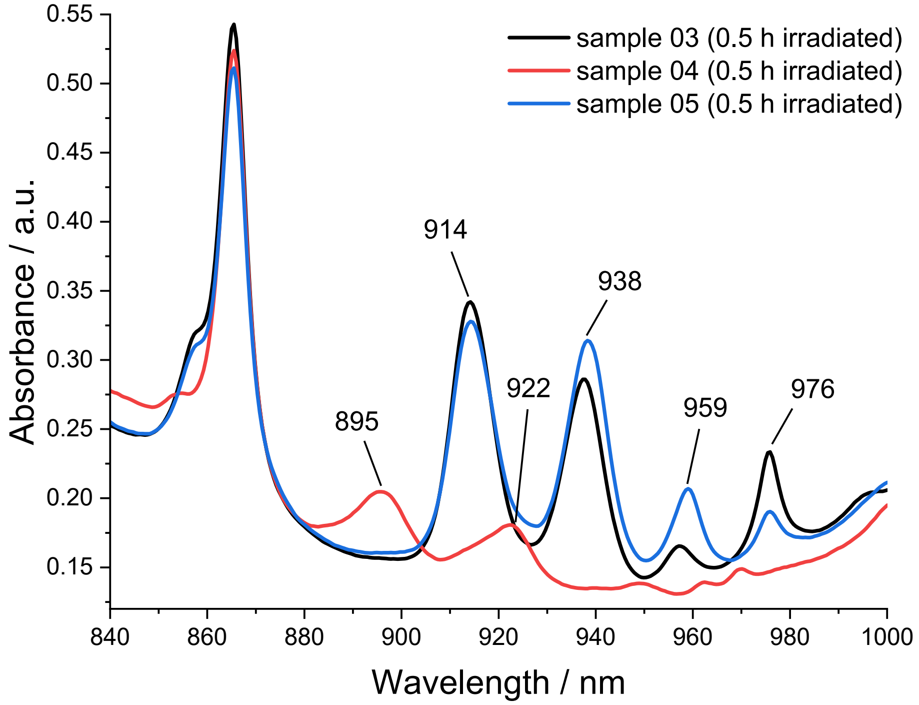

Figure S 88. FTIR spectra (840-1000 cm^{-1} region) of the sample 03 after 0.5 h irradiation (black line), sample 04 after 0.5 h irradiation (red line), and sample 05 after 0.5 h irradiation (blue line). For sample composition, see Table S20; samples were irradiated at λ = 410 nm.

Reactions between PPh_3 and DMSO-d_6 catalysed by $3^{C(O)OMe}$ in the presence of TTBP and $[nBu_4N][PF_6]$

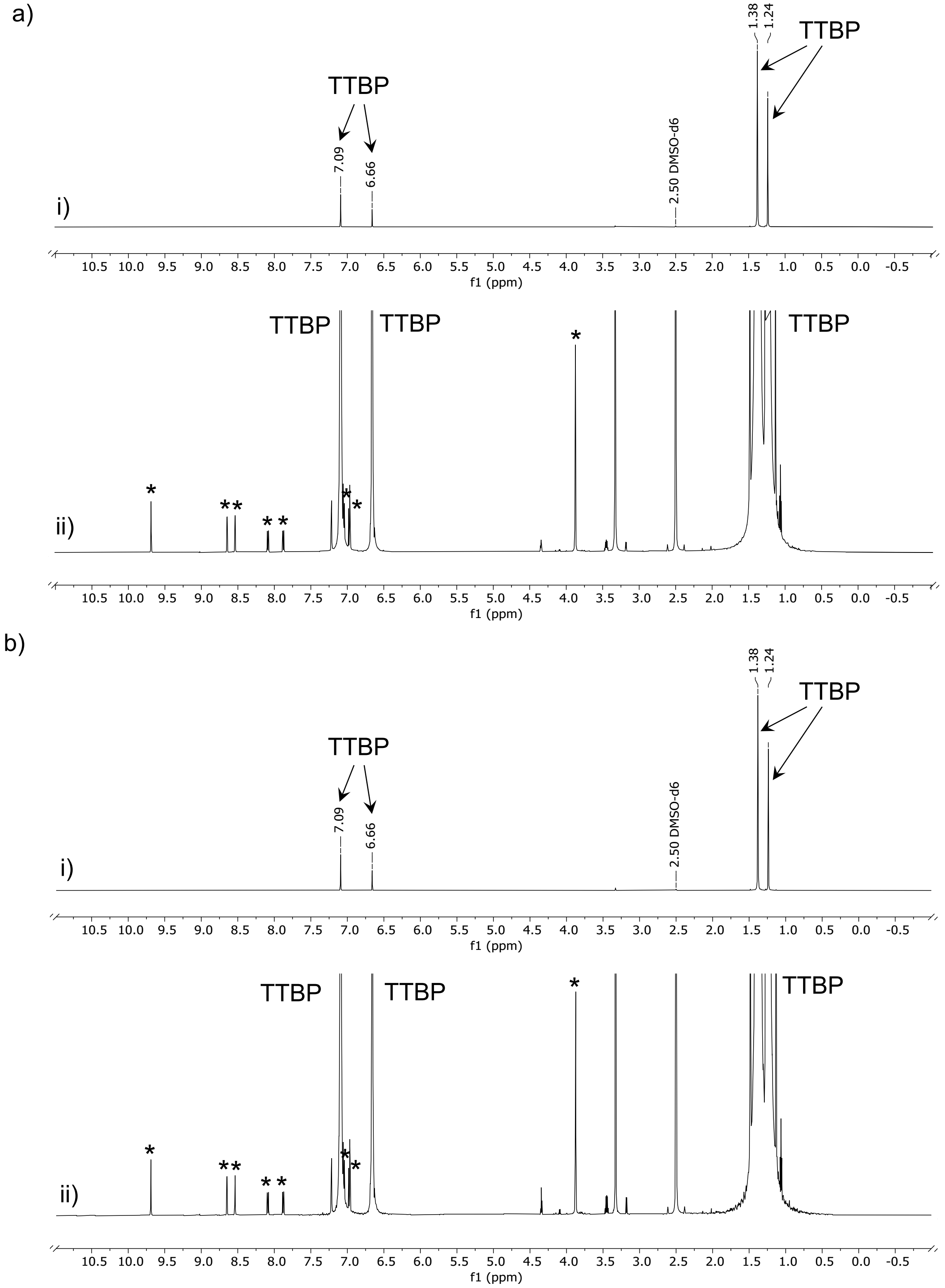

Figure S 89. ^1H NMR spectrum (600 MHz; DMSO-d_6, 25°C) of $\mathbf{3^{C(O)OMe}}$ (1 mM) and TTBP (300 mM) in DMSO-d_6 before (a) and after (b) irradiation at 410 nm for 3 h; i) full spectra; ii) zoomed-in spectra. Catalyst signals are marked with an asterix; catalyst signals do not change, showing catalyst stability in the presence of excess TTBP during irradiation.

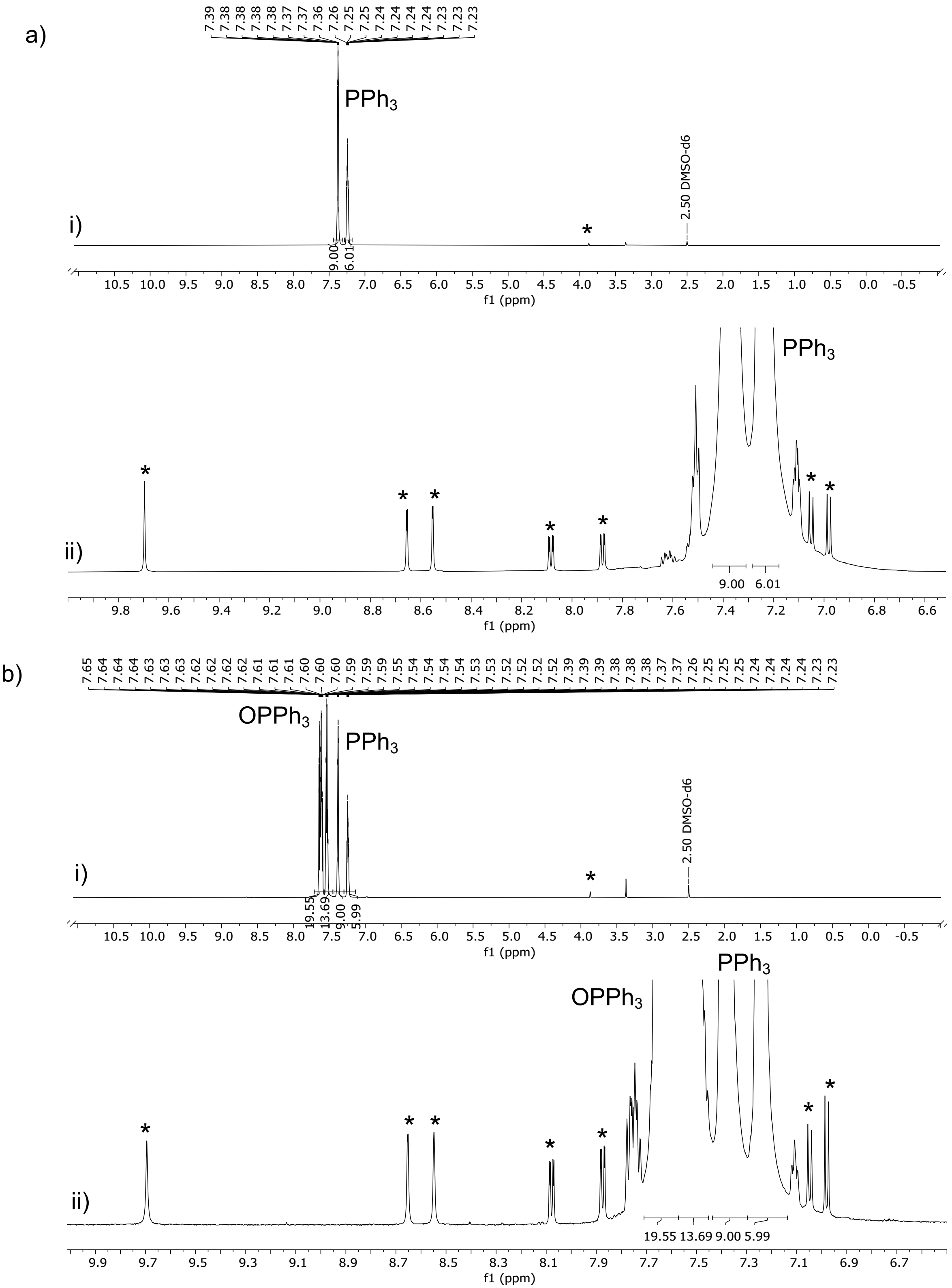

Figure S 90. ^{1}H NMR spectrum (600 MHz; DMSO-d$_6$, 25°C) of **3**$^{\mathbf{C(O)OMe}}$ and PPh_3 (300 equiv) in DMSO-d_6 before (a) and after (b) irradiation at 410 nm for 3 h; i) full spectra; ii) zoomed-in spectra. Catalyst signals are marked with an asterix.

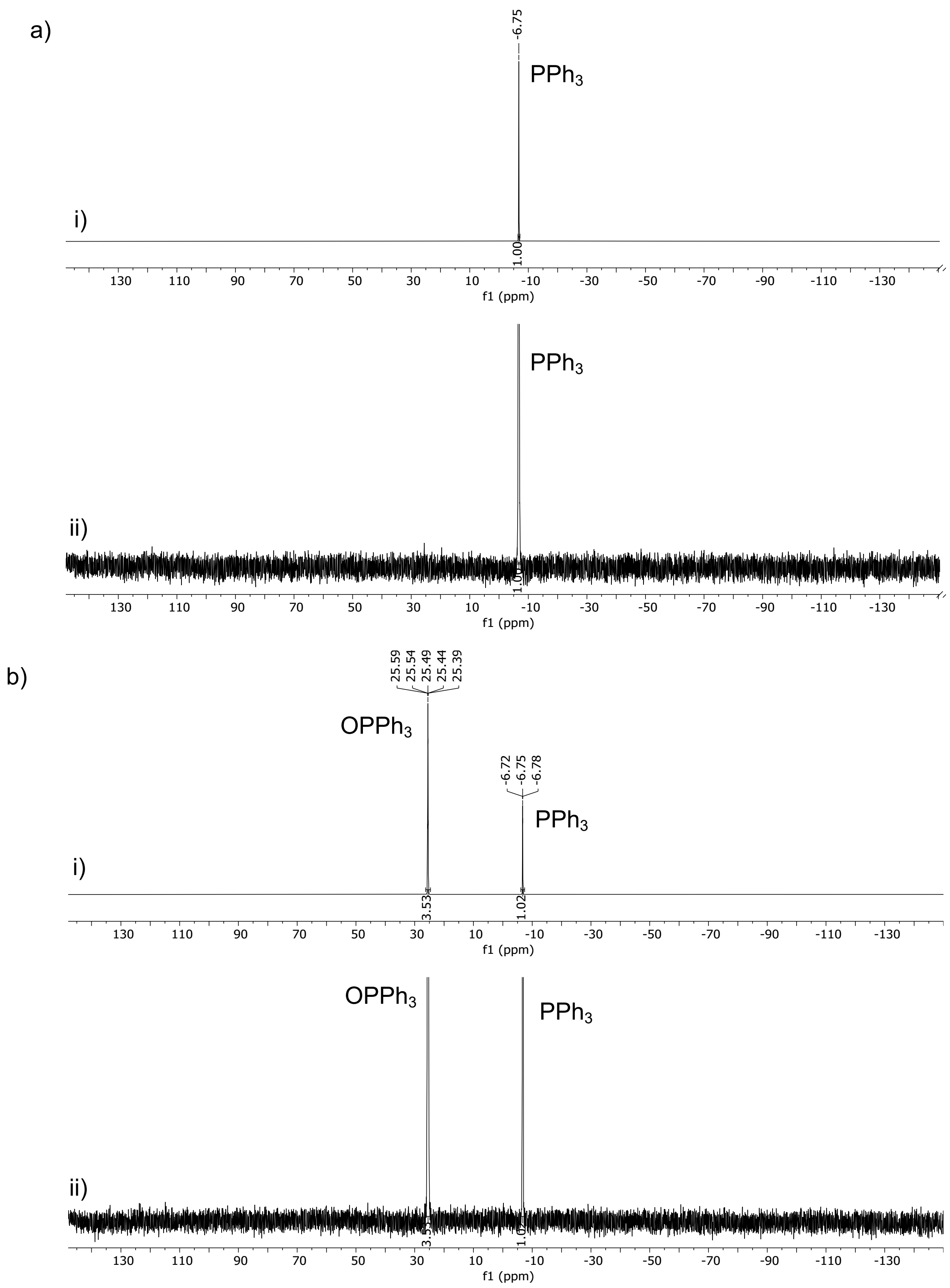

Figure S 91. ^{31}P NMR spectrum (243 MHz; DMSO-d$_6$, 25°C) of $\mathbf{3}^{C(O)OMe}$ and PPh_3 (300 equiv) in DMSO-d_6 before (a) and after (b) irradiation at 410 nm for 3 h; i) full spectra; ii) zoomed-in spectra. No additional phosphine-containing species apart from PPh_3 and $OPPh_3$ are observed after irradiation.

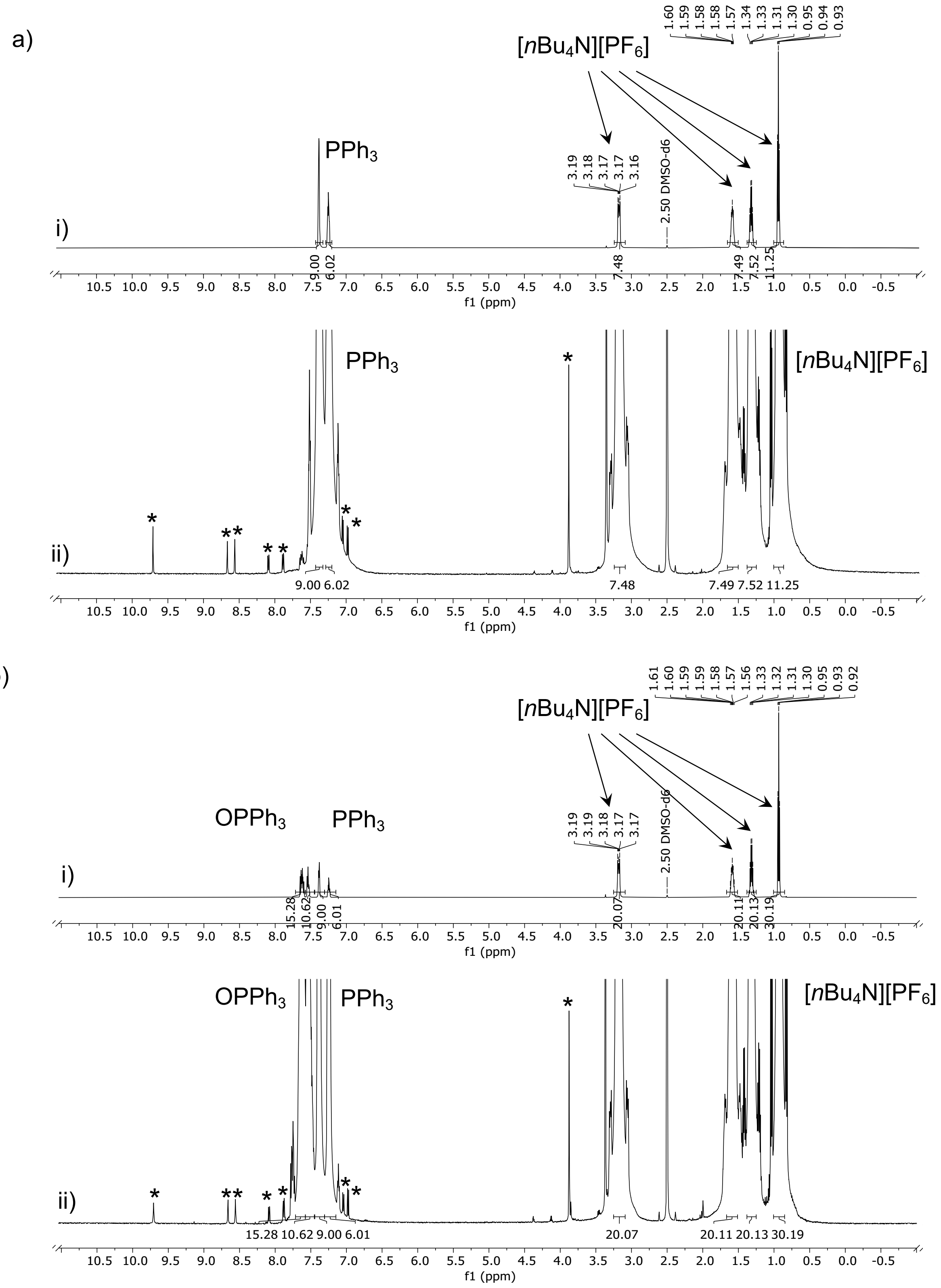

Figure S 92. ^{1}H NMR spectrum (600 MHz; DMSO-d$_6$, 25°C) of $\mathbf{3}^{\mathbf{C(O)OMe}}$, PPh_3 (300 equiv), and [nBu$_4$N][PF$_6$] (250 mM) in DMSO-d_6 before (a) and after (b) irradiation at 410 nm for 3 h; i) full spectra; ii) zoomed-in spectra. Catalyst signals are marked with an asterix; catalyst signals do not change, showing catalyst stability in the presence of excess [nBu$_4$N][PF$_6$] during irradiation.

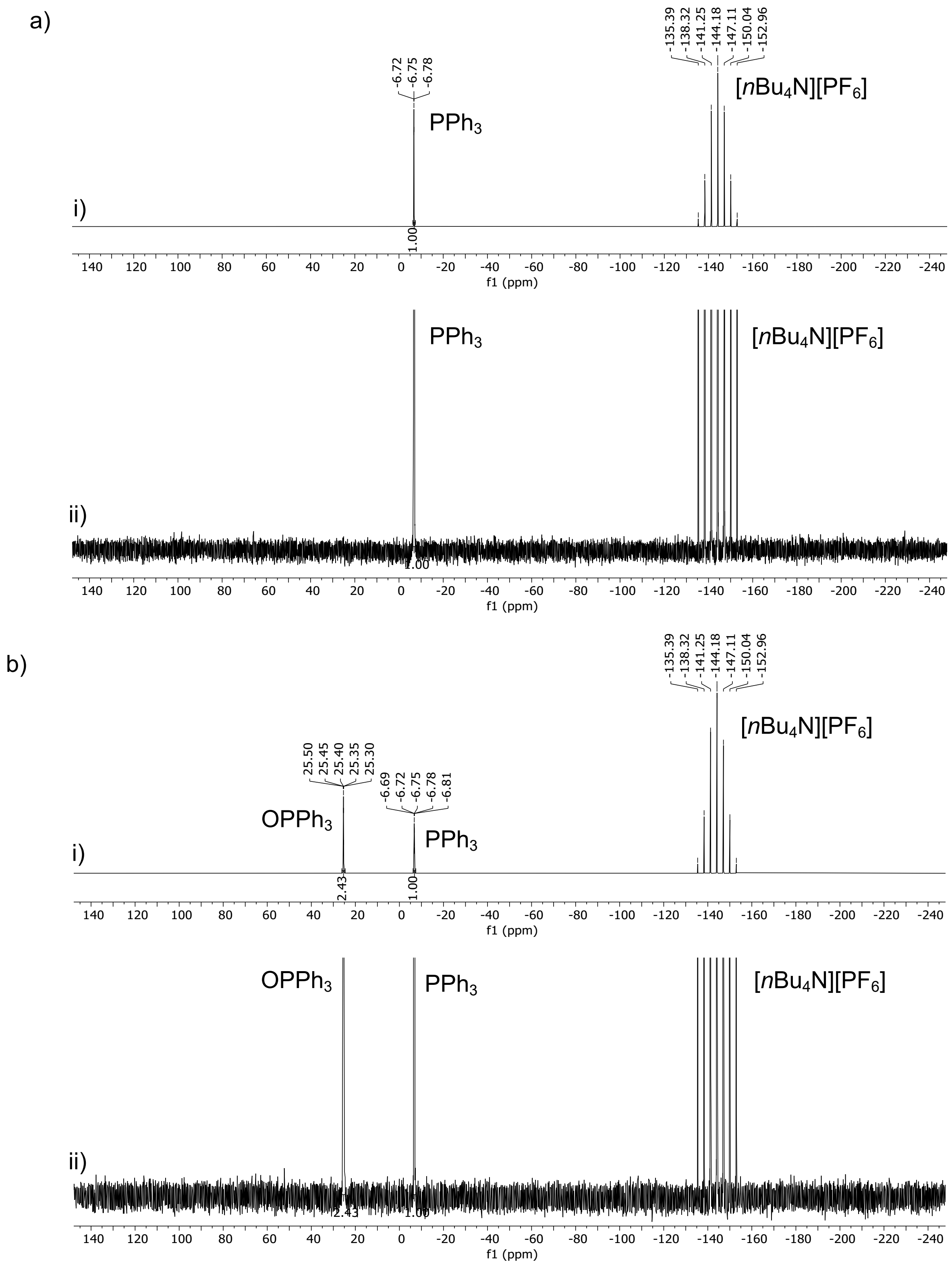

Figure S 93. ^{31}P NMR spectrum (243 MHz; DMSO-d$_6$, 25°C) of $\mathbf{3}^{\mathbf{C(O)OMe}}$, PPh_3 (300 equiv), and [*n*Bu_4N][PF_6] (250 mM) in DMSO-*d*$_6$ before (a) and after (b) irradiation at 410 nm for 3 h; i) full spectra; ii) zoomed-in spectra. No additional phosphine-containing species apart from PPh_3, $OPPh_3$, and [*n*Bu_4N][PF_6] are observed after irradiation.

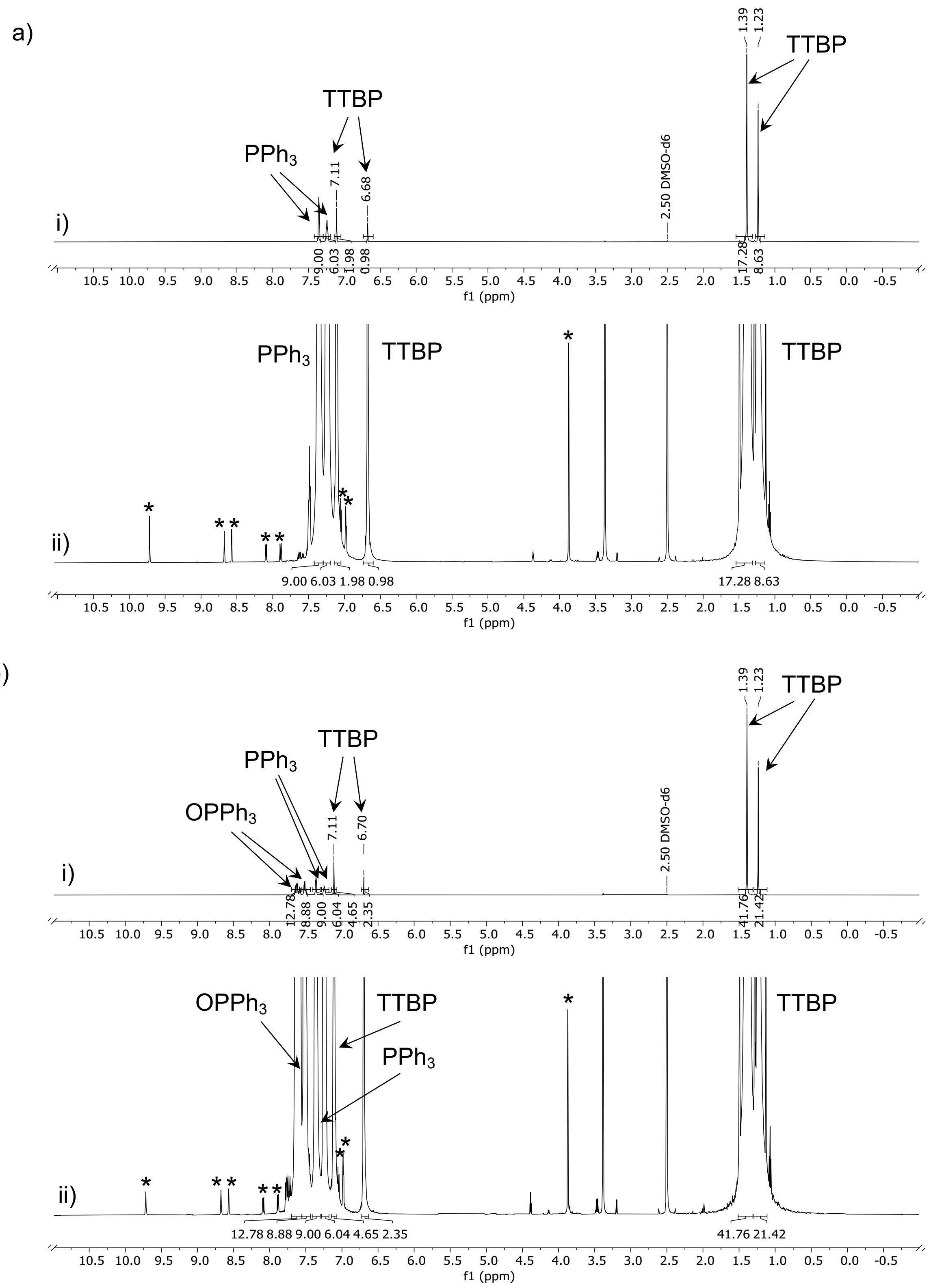

Figure S 94. ^{1}H NMR spectrum (600 MHz; DMSO-d_6, 25°C) of $\mathbf{3}^{C(O)OMe}$, PPh_3 (300 equiv), and TTBP (300 mM) in DMSO-d_6 before (a) and after (b) irradiation at 410 nm for 3 h; i) full spectra; ii) zoomed-in spectra. Catalyst signals are marked with an asterix; catalyst signals do not change, showing catalyst stability in the presence of excess TTBP during irradiation.

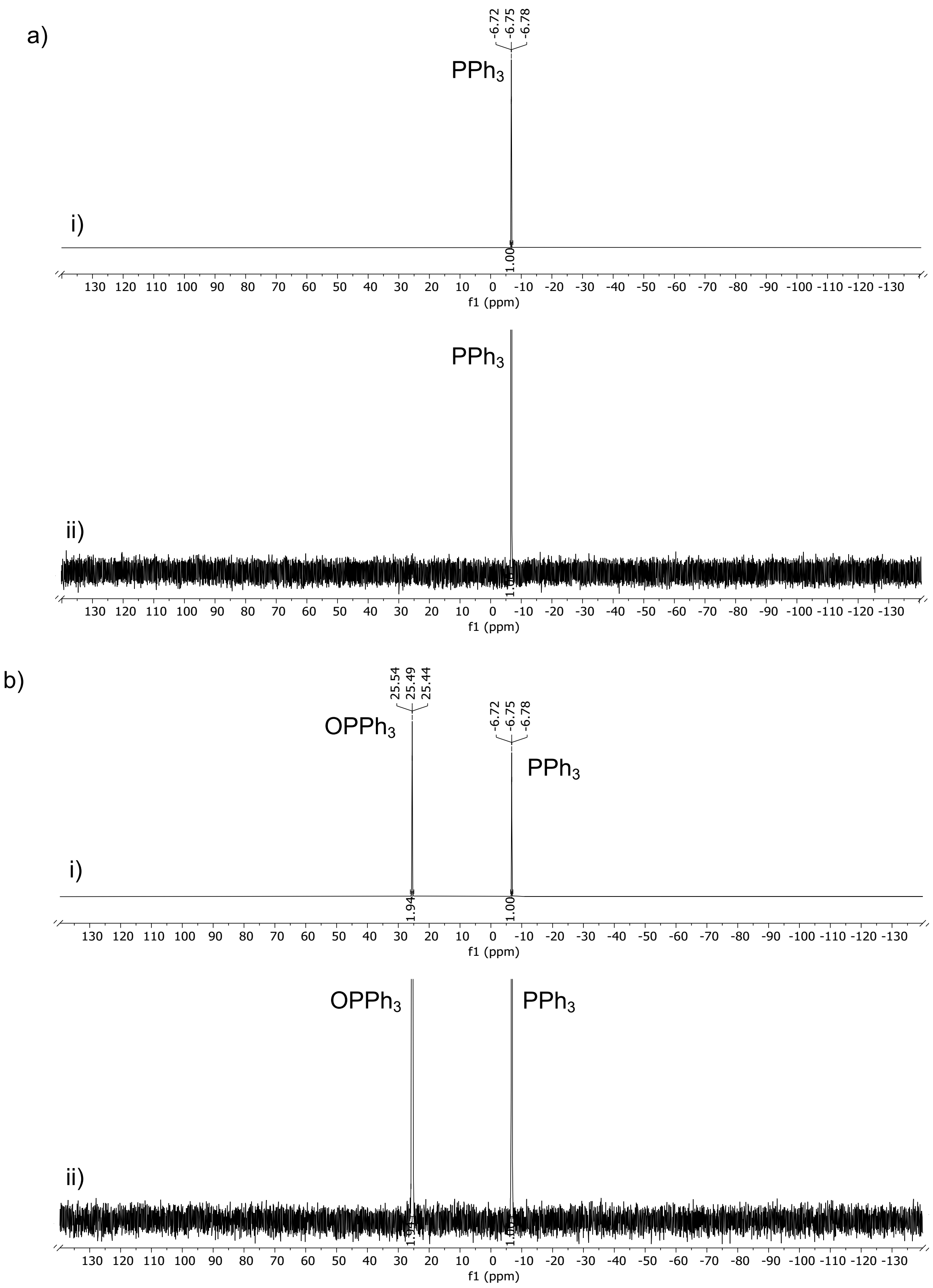

Figure S 95. ^{31}P NMR spectrum (243 MHz; DMSO-d$_6$, 25°C) of $\mathbf{3^{C(O)OMe}}$, PPh_3 (300 equiv), and TTBP (300 mM) in DMSO-*d*$_6$ before (a) and after (b) irradiation at 410 nm for 3 h; i) full spectra; ii) zoomed-in spectra. No additional phosphine-containing species apart from PPh_3 and $OPPh_3$ are observed after irradiation.

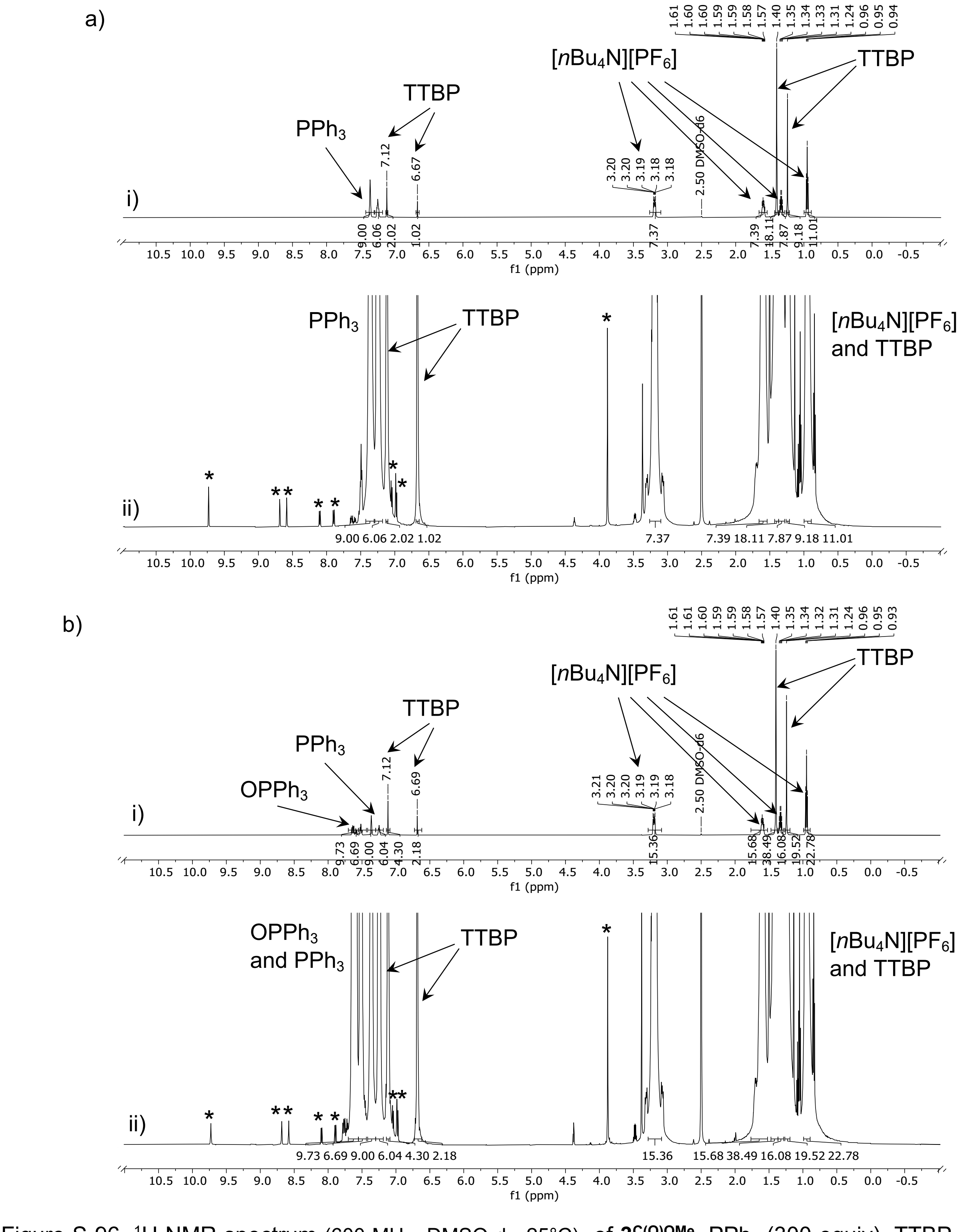

Figure S 96. ^{1}H NMR spectrum (600 MHz; DMSO-d_6, 25°C) of $\mathbf{3}^{\mathbf{C(O)OMe}}$, PPh_3 (300 equiv), TTBP (300 mM), and [nBu$_4$N][PF_6] (250 mM) in DMSO-d_6 before (a) and after (b) irradiation at 410 nm for 3 h; i) full spectra; ii) zoomed-in spectra. Catalyst signals are marked with an asterix; catalyst signals do not change, showing catalyst stability in the presence of excess TTBP and [nBu$_4$N][PF_6] during irradiation.

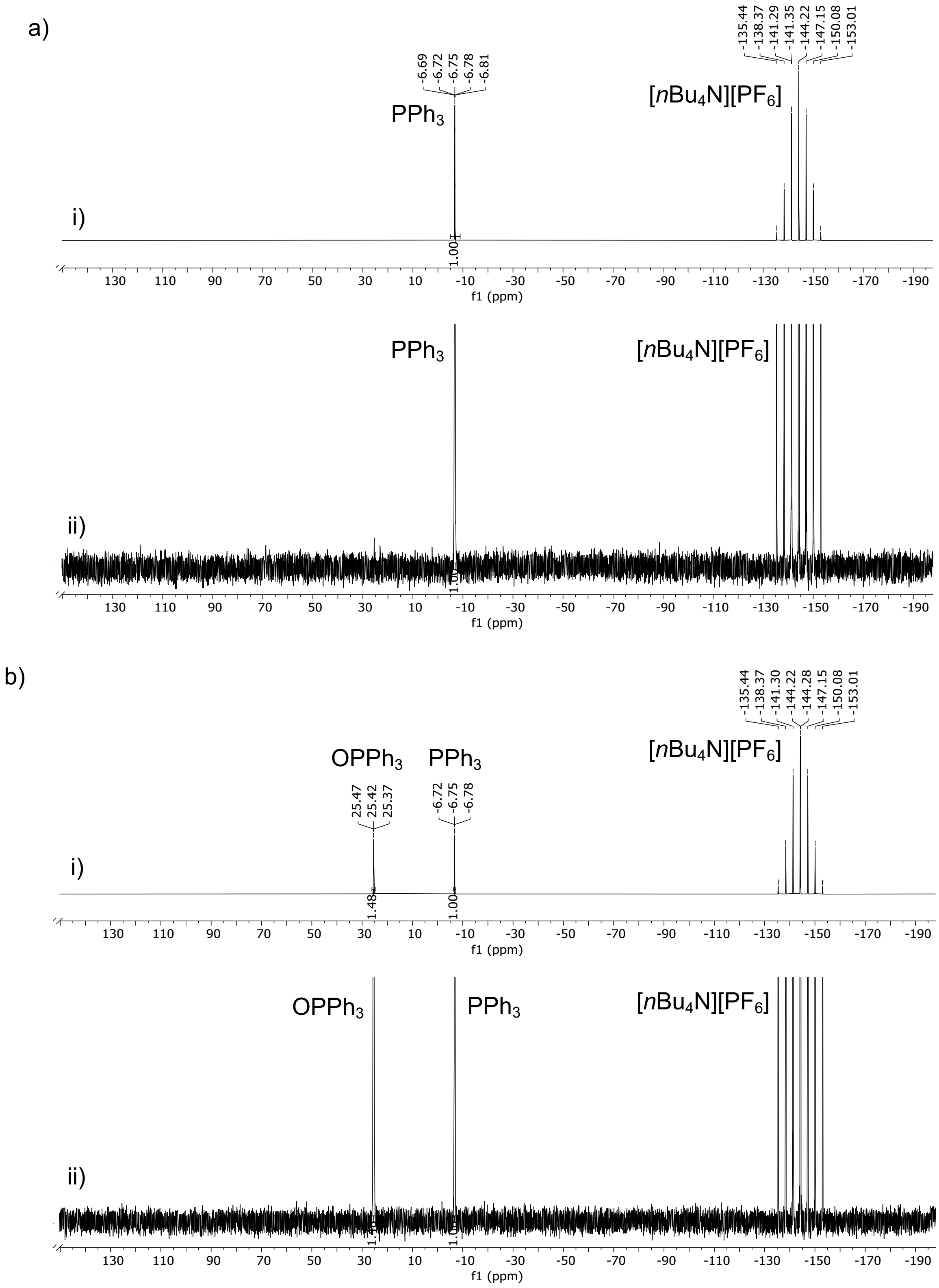

Figure S 97. ^{31}P NMR spectrum (243 MHz; DMSO-d_6, 25°C) of $\mathbf{3}^{C(O)OMe}$, PPh_3 (300 equiv), TTBP (300 mM), and [nBu$_4$N][PF$_6$] (250 mM) in DMSO-d_6 before (a) and after (b) irradiation at 410 nm for 3 h; i) full spectra; ii) zoomed-in spectra. No additional phosphine-containing species apart from PPh_3, $OPPh_3$, and [nBu$_4$N][PF$_6$] are observed after irradiation.

Table S 21. Measured PPh_3 to $OPPh_3$ conversions for reactions between PPh_3 and DMSO-d_6 catalysed by **3**$^{C(O)OMe}$ in the presence of TTBP and [$n$$Bu_4$N][$PF_6$] (after 3 h irradiation at 410 nm); calculated from ^{1}H NMR integration values (i [$OPPh_3$]$_t$) / (i [PPh_3]$_t$ + i [$OPPh_3$]$_t$)).

	Conversion / %			
Sample composition	**3**$^{C(O)OMe}$ 1 mM<br>PPh_3 300 mM<br>DMSO-d_6 0.7 mL	**3**$^{C(O)OMe}$ 1 mM<br>PPh_3 300 mM<br>[$n$$Bu_4$N][$PF_6$] 250 mM<br>DMSO-$d_6$ 0.7 mL	**3**$^{C(O)OMe}$ 1 mM<br>PPh_3 300 mM<br>TTBP 300 mM<br>DMSO-d_6 0.7 mL	**3**$^{C(O)OMe}$ 1 mM<br>PPh_3 300 mM<br>TTBP 300 mM<br>[$n$$Bu_4$N][$PF_6$] 250 mM<br>DMSO-$d_6$ 0.7 mL
Original experiment	68	64	59	52
Repeat 1	69	64	55	54
Repeat 2	71	65	57	52
Repeat 3	68	67	55	53
Mean conversion	69	65	57	53
Conversion range	3	3	4	2
Estimated error	± 2	± 2	± 2	± 1

Reactions between PPh_3 and DMSO-d_6 catalysed by $3^{C(O)OMe}$ in the presence of alkenes

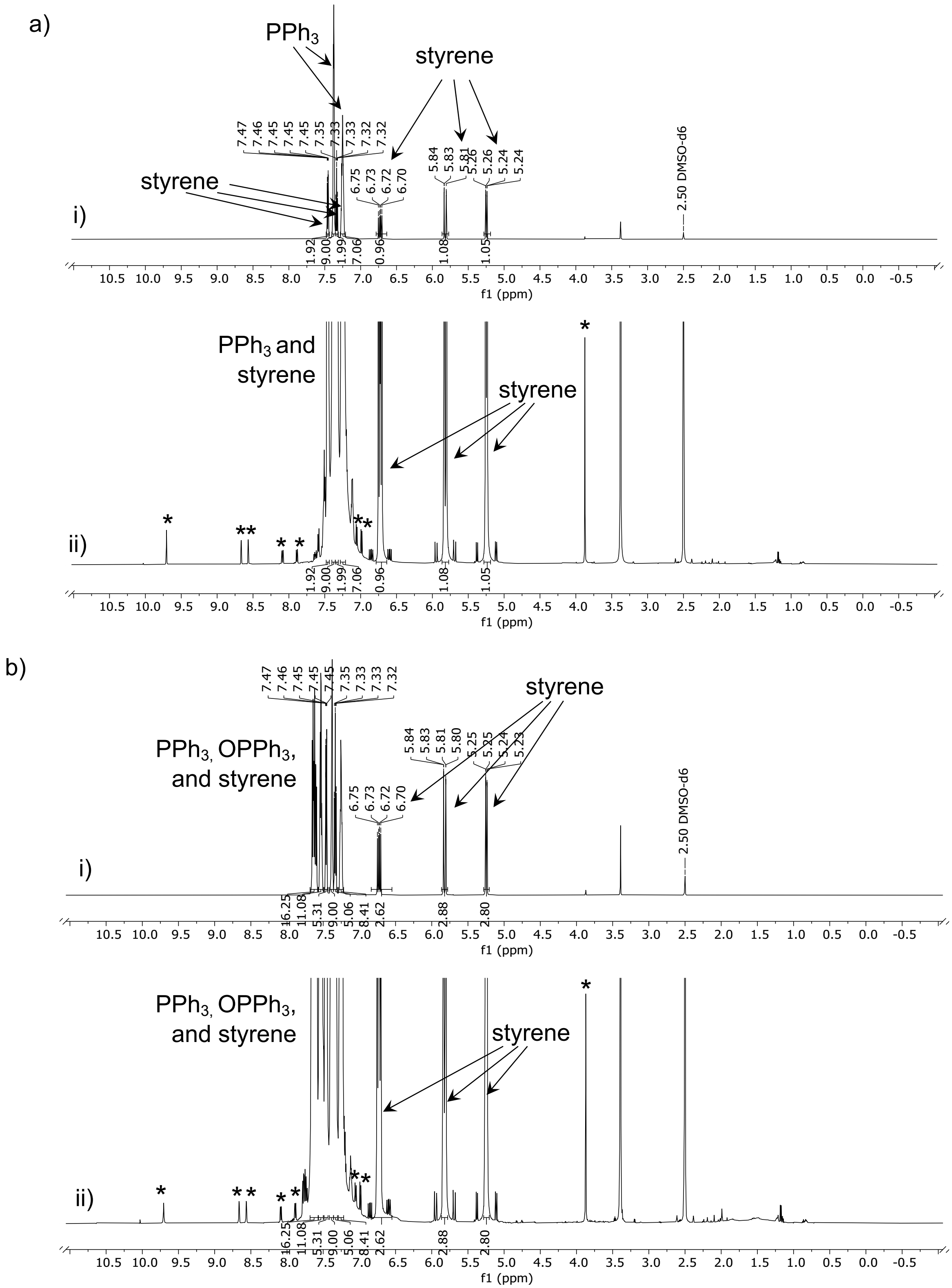

Figure S 98. ^{1}H NMR spectrum (600 MHz; DMSO-d_6, 25°C) of $\mathbf{3}^{C(O)OMe}$, PPh_3 (300 equiv), and styrene (300 equiv) in DMSO-d_6 before (a) and after (b) irradiation at 410 nm for 3 h; i) full spectra; ii) zoomed-in spectra. Catalyst signals are marked with an asterix.

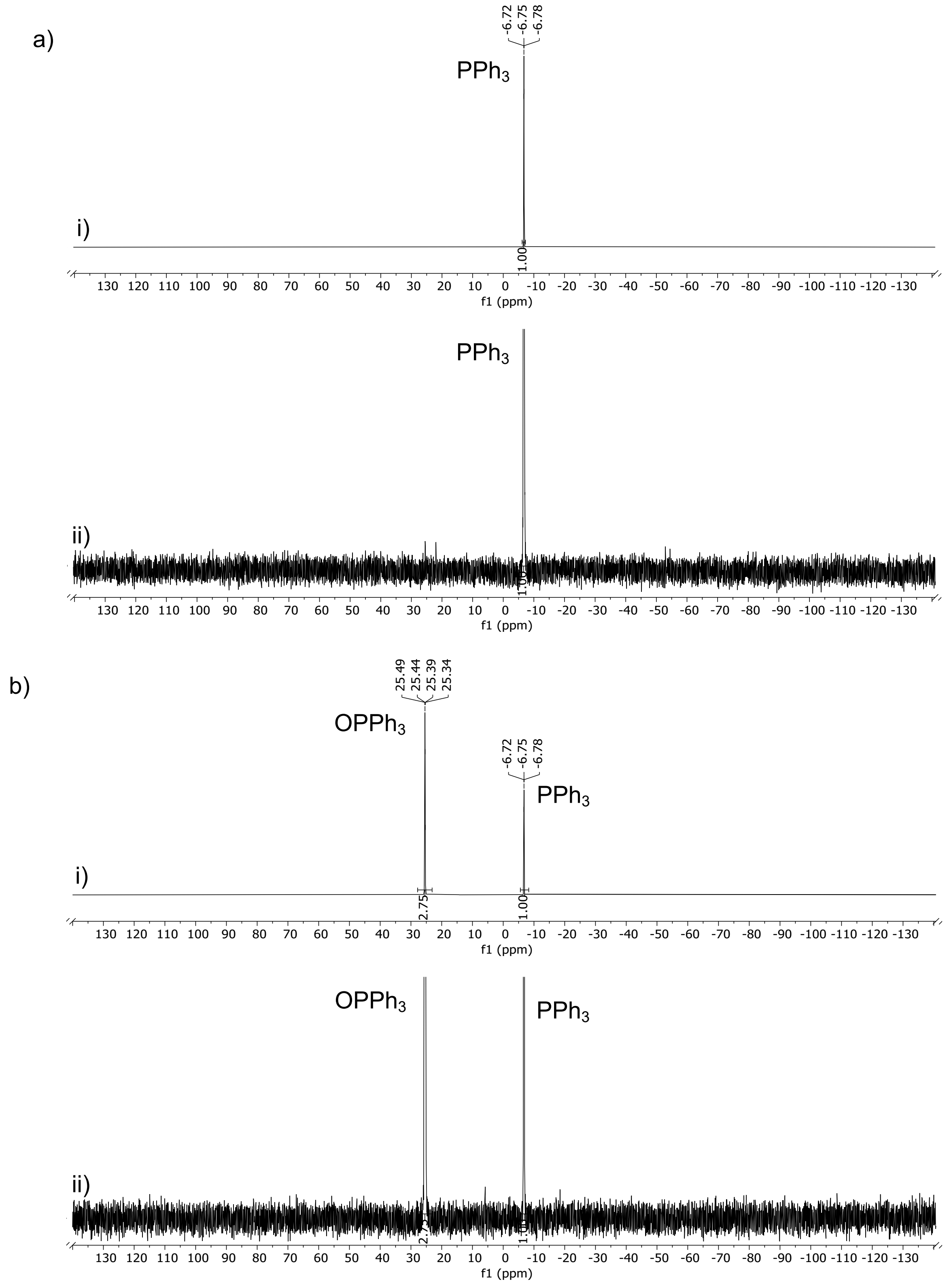

Figure S 99. ^{31}P NMR spectrum (243 MHz; DMSO-d$_6$, 25°C) of **3$^{C(O)OMe}$**, PPh_3 (300 equiv), and styrene (300 equiv) in DMSO-*d*$_6$ before (a) and after (b) irradiation at 410 nm for 3 h; i) full spectra; ii) zoomed-in spectra. No additional phosphine-containing species apart from PPh_3 and $OPPh_3$ are observed after irradiation.

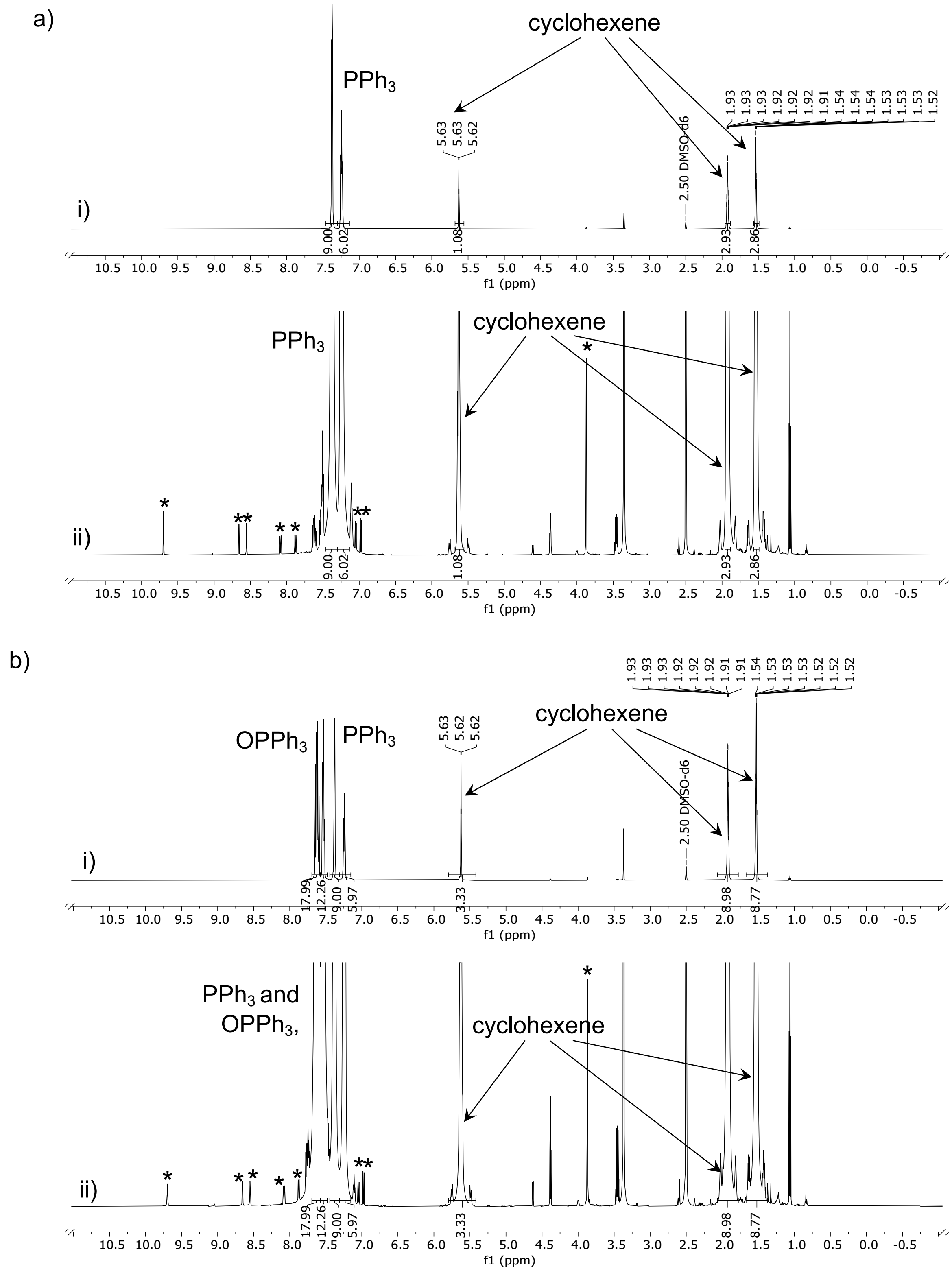

Figure S 100. ^{1}H NMR spectrum (600 MHz; DMSO-d_6, 25°C) of $\mathbf{3}^{\mathbf{C(O)OMe}}$, PPh_3 (300 equiv), and cyclohexene (300 equiv) in DMSO-d_6 before (a) and after (b) irradiation at 410 nm for 3 h; i) full spectra; ii) zoomed-in spectra. Catalyst signals are marked with an asterix.

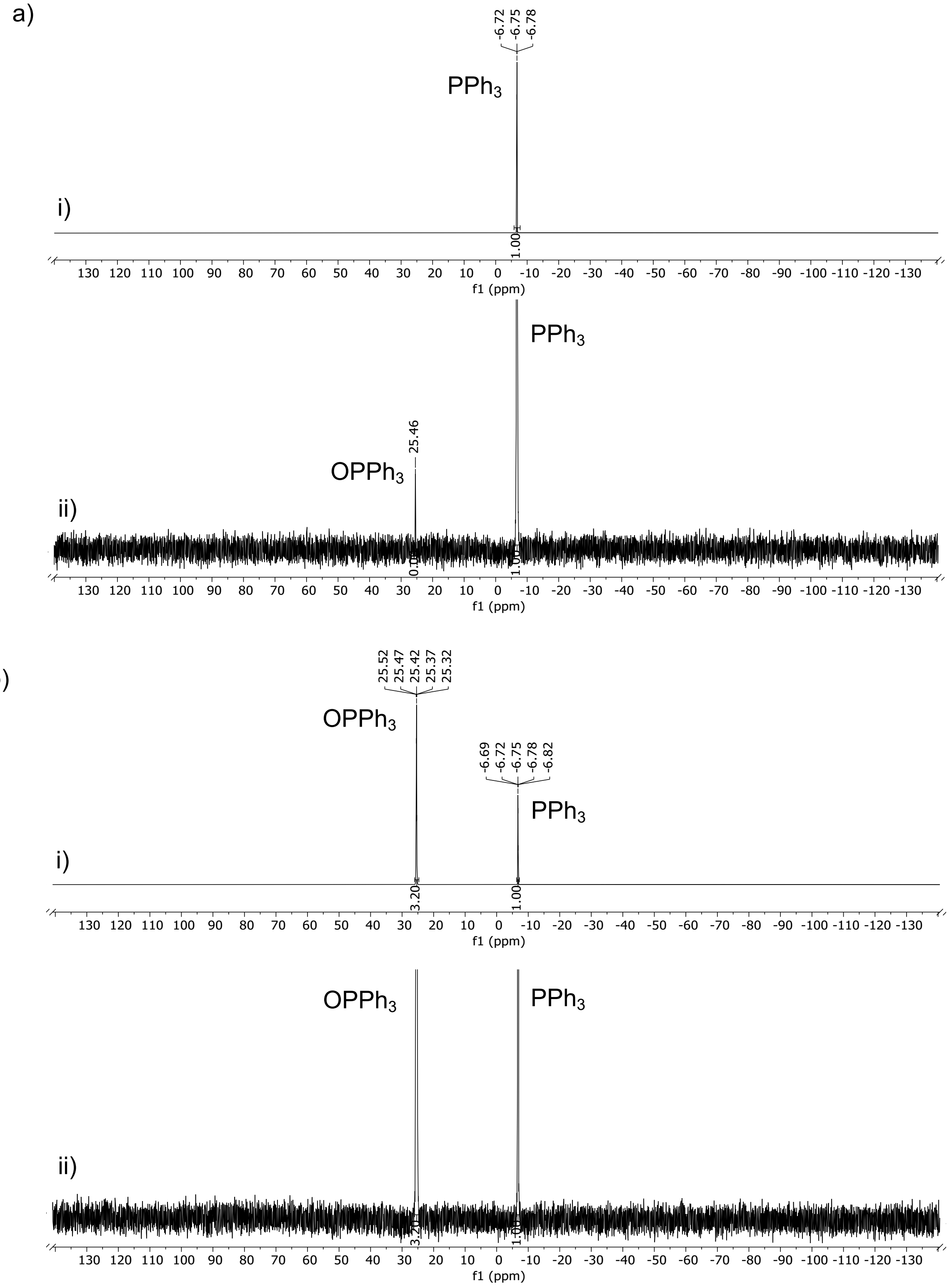

Figure S 101. ^{31}P NMR spectrum (243 MHz; DMSO-d$_6$, 25°C) of **3**$^{\mathbf{C(O)OMe}}$, PPh_3 (300 equiv), and cyclohexene (300 equiv) in DMSO-*d*$_6$ before (a) and after (b) irradiation at 410 nm for 3 h; i) full spectra; ii) zoomed-in spectra. No additional phosphine-containing species apart from PPh_3 and $OPPh_3$ are observed after irradiation.

References

1 CrysAlisPro, Oxford Diffraction Ltd.

2 Empirical absorption correction using spherical harmonics, implemented in SCALE3 ABSPACK scaling algorithm within CrysAlisPro software, Oxford Diffraction Ltd.

3 O. V. Dolomanov, L. J. Bourhis, R. J. Gildea, J. A. K. Howard and H. Puschmann, *J. Appl. Crystallogr.*, 2009, **42**, 339–341.

4 G. M. Sheldrick, *Acta Crystallogr. A*, 2015, **71**, 3–8.

5 G. M. Sheldrick, *Acta Crystallogr. C. Struct. Chem.*, 2015, **71**, 3–8.

6 J. P. Perdew, *Phys. Rev. B,* 1986, **33**, 8822–8824.

7 A. D. Becke, *J. Chem. Phys.*, 1996, **104**, 1040–1046.

8 F. Weigend, *Phys. Chem. Chem. Phys.*, 2006, **8**, 1057–1065.

9 F. Weigend and R. Ahlrichs, *Phys. Chem. Chem. Phys.*, 2005, **7**, 3297–3305.

10 J. Tomasi, B. Mennucci and R. Cammi, *Chem. Rev.*, 2005, **105**, 2999–3093.

11 M. J. Frisch, G. W. Trucks, H. B. Schlegel, G. E. Scuseria, M. A. Robb, J. R. Cheeseman, G. Scalmani, V. Barone, G. A. Petersson, H. Nakatsuji, X. Li, M. Caricato, A. V. Marenich, J. Bloino, B. G. Janesko, R. Gomperts, B. Mennucci, H. P. Hratchian, J. V. Ortiz, A. F. Izmaylov, J. L. Sonnenberg, D. Williams-Young, F. Ding, F. Lipparini, F. Egidi, J. Goings, B. Peng, A. Petrone, T. Henderson, D. Ranasinghe, V. G. Zakrzewski, J. Gao, N. Rega, G. Zheng, W. Liang, M. Hada, M. Ehara, K. Toyota, R. Fukuda, J. Hasegawa, M. Ishida, T. Nakajima, Y. Honda, O. Kitao, H. Nakai, T. Vreven, K. Throssell, J. A. Montgomery, Jr., J. E. Peralta, F. Ogliaro, M. J. Bearpark, J. J. Heyd, E. N. Brothers, K. N. Kudin, V. N. Staroverov, T. A. Keith, R. Kobayashi, J. Normand, K. Raghavachari, A. P. Rendell, J. C. Burant, S. S. Iyengar, J. Tomasi, M. Cossi, J. M. Millam, M. Klene, C. Adamo, R. Cammi, J. W. Ochterski, R. L. Martin, K. Morokuma, O. Farkas, J. B. Foresman, D. J. Fox, Gaussian 16 (Revision C.01), Gaussian, Inc., Wallingford, CT, 2016.

12 C. Adamo and V. Barone, *J. Chem. Phys.*, 1999, **110**, 6158–6170.

13 T. Yanai, D. P. Tew and N. C. Handy, *Chem. Phys. Lett.*, 2004, **393**, 51–57.

14 Y. Zhao and D. G. Truhlar, *Theor. Chem. Acc.*, 2008, **120**, 215–241.

15 J. Da Chai and M. Head-Gordon, *Phys. Chem. Chem. Phys.*, 2008, **10**, 6615–6620.

16 V. Béreau, V. Jubéra, P. Arnaud, A. Kaiba, P. Guionneau and J. P. Sutter, *J. Chem. Soc., Dalton Trans.*, 2010, **39**, 2070–2077.

17 R. M. Irfan, D. Jiang, Z. Sun, D. Lu and P. Du, *Dalton Trans.*, 2016, **45**, 12897–12905.

18 M. Livendahl, J. Jamroskovic, M. Hedenström, T. Görlich, N. Sabouri and E. Chorell, *Org. Biomol. Chem.*, 2017, **15**, 3265–3275.

19 Akanksha and D. Maiti, *Green Chem.*, 2012, **14**, 2314–2320.

20 P. Jaikhan, B. Buranrat, Y. Itoh, J. Chotitumnavee, T. Kurohara and T. Suzuki, *Bioorg. Med. Chem. Lett.*, 2019, **29**, 1173–1176.

21 H. Gehrke and J. Veal, *Inorg. Chim. Acta,* 1969, **3**, 623–627.

22 M. M. Folkendt, B. E. Weiss-Lopez, J. P. Chauvel and N. S. True, *J. Phys. Chem.,* 1985, **89**, 3347–3352.

23 H. Mahmoudi, M. Bagherzadeh, S. Ataie, R. Kia, S. Heydar Moravej, M. Zare, P. R. Raithby, F. Ferlin and L. Vaccaro, *Inorg. Chim. Acta,* 2020, **511**, DOI: 10.1016/j.ica.2020.119775.

24 X. Y. Chen, S. Ozturk and E. J. Sorensen, *Org. Lett.*, 2017, **19**, 6280–6283.

25 A. Guha, R. Sanyal, T. Chattopadhyay, Y. Han, T. K. Mondal and D. Das, *J. Mol. Struct.*, 2013, **1042**, 104–111.

26 S. Kim, J. Y. Noh, K. Y. Kim, J. H. Kim, H. K. Kang, S. W. Nam, S. H. Kim, S. Park, C. Kim and J. Kim, *Inorg. Chem.*, 2012, **51**, 3597–3602.

27 B. Das, A. Chakraborty and S. Chakraborty, *Spectrochim. Acta A Mol. Biomol. Spectrosc.*, 2020, **225**, DOI:10.1016/j.saa.2019.117443.

28 C. Hansch, A. Leo and R. W. Taft, *Chem. Rev.,* 1991, **91**, 165–195.

29 O. E. Brian Schultz, S. F. Gheller, M. C. Muetterties, M. J. Scott and R. H. Holm, *J. Am. Chem. Soc.*, 1993**,** 115, 2714–2722.

30 R. Sanz, J. Escribano, Y. Fernández, R. Aguado, M. R. Pedrosa and F. J. Arnáiz, *Synlett*, 2005, **9**, 1389–1392.
